# Associations between lipids in selected brain regions, plasma miRNA, and behavioral and cognitive measures following ^28^Si ion irradiation

**DOI:** 10.1038/s41598-021-93869-3

**Published:** 2021-07-21

**Authors:** Jessica Minnier, Mark R. Emmett, Ruby Perez, Liang-Hao Ding, Brooke L. Barnette, Rianna E. Larios, Changjin Hong, Tae Hyun Hwang, Yongjia Yu, Christina M. Fallgren, Michael D. Story, Michael M. Weil, Jacob Raber

**Affiliations:** 1 Oregon Health & Science University-Portland State University School of Public Health, Knight Cancer Institute Biostatistics Shared Resource, and the Knight Cardiovascular Institute, OR Health & Science University, Portland, OR 97239 USA; 2 Department of Biochemistry and Molecular Biology; Radiation Oncology, Pharmacology and Toxicology, Mitchell Center for Neurodegenerative Diseases, University of Texas Medical Branch Cancer Center, Galveston, TX 77555 USA; 3 Department of Behavioral Neuroscience, L470, Oregon Health & Science University, 3181SW Sam Jackson Park Road, Portland, OR 97239 USA; 4 Department of Radiation Oncology, University of Texas Southwestern Medical Center, Dallas, TX 75390 USA; 5 Lerner Research Institute, Cleveland Clinic Lerner College of Medicine US, Cleveland, OH 44195 USA; 6 Department of Molecular Medicine, School of Medicine, GU Malignancies Program, Case Comprehensive Cancer Center, Genomic Medicine Institute, Case Western Reserve University US., Cleveland, OH 10900 USA; 7 Department of Environmental and Radiological Health Sciences, Colorado State University, Fort Collins, CO 80523 USA; 8 Simmons Comprehensive Cancer Center, University of Texas Southwestern Medical Center, Dallas, TX USA; 9 Division of Neuroscience ONPRC, Departments of Neurology, Psychiatry, and Radiation Medicine, Oregon Health & Science University, Portland, OR 97239 USA

**Keywords:** Neuroscience, Environmental sciences

## Abstract

The space radiation environment consists of multiple species of charged particles, including ^28^Si ions, that may impact brain function during and following missions. To develop biomarkers of the space radiation response, BALB/c and C3H female and male mice and their F2 hybrid progeny were irradiated with ^28^Si ions (350 MeV/n, 0.2 Gy) and tested for behavioral and cognitive performance 1, 6, and 12 months following irradiation. The plasma of the mice was collected for analysis of miRNA levels. Select pertinent brain regions were dissected for lipidomic analyses and analyses of levels of select biomarkers shown to be sensitive to effects of space radiation in previous studies. There were associations between lipids in select brain regions, plasma miRNA, and cognitive measures and behavioral following ^28^Si ion irradiation. Different but overlapping sets of miRNAs in plasma were found to be associated with cognitive measures and behavioral in sham and irradiated mice at the three time points. The radiation condition revealed pathways involved in neurodegenerative conditions and cancers. Levels of the dendritic marker MAP2 in the cortex were higher in irradiated than sham-irradiated mice at middle age, which might be part of a compensatory response. Relationships were also revealed with CD68 in miRNAs in an anatomical distinct fashion, suggesting that distinct miRNAs modulate neuroinflammation in different brain regions. The associations between lipids in selected brain regions, plasma miRNA, and behavioral and cognitive measures following ^28^Si ion irradiation could be used for the development of biomarker of the space radiation response.

## Introduction

The space radiation environment may pose a hazard to behavioral and cognitive performance of space flight crews during and following the mission(s) due to the presence of galactic cosmic rays (GCR) and solar particle events (SPE). GCR involves fully ionized atomic nuclei while SPE includes low to medium energy protons with a small heavy ion component. The heavy ion component includes ^28^Si ions. ^28^Si ion irradiation was shown to affect fear memory in C57BL/6J^1^ and C57Bl6/J 3 DBA2/J F1 hybrid mice^2^ in a strain-dependent fashion. An important question is whether there are biomarkers in plasma or in the brain of the mice that are associated with behavioral and cognitive measures.

Brain enriched micro RNAs (miRNAs) in plasma have been shown to be biomarkers of neurodevelopmental disorders in humans and animal models^3^. miRNA-based diagnostics are also being developed for the detection and differentiation of neurodegenerative conditions like Alzheimer’s disease, Parkinson’s disease, frontotemporal dementia, and amyotrophic lateral sclerosis^4^. Altered expression of miRNA-223 was seen in patients with first-episode schizophrenia^5^, of miRNA 4428 and 4480 in patients with advanced breast cancer and brain metastasis^6^, and reduced plasma levels of circulating brain miRNA-134 and miRNA-2392 expression were observed in interventional cardiologists exposed professionally to ionizing irradiation compared with controls^7^. Select miRNAs in plasma have also been identified as biomarkers of environmental challenges such as traumatic brain injury^8^ and to predict the response to radiotherapy in cancer patients^9^.

The hypothalamus and amygdala are brain regions important in the stress response^10–12^. The amygdala is also important in emotional learning and memory^13–15^. Changes in the lipidome in rats were seen with aging^16^. Differences in hypothalamic lipid profiles in young and aged rats with intact and impaired cognitive abilities have been reported^17^.

In the present study, to develop biomarkers of the space radiation response, BALB/c and C3H female and male mice and their F2 hybrid progeny were irradiated with ^28^Si ions (350 MeV/n, 0.2 Gy) and tested for behavioral and cognitive performance 1, 6, and 12 months following irradiation. The plasma of the mice was collected for analysis of miRNA levels and the hypothalamus and amygdala were dissected for lipidomics analyses and analyses of levels of select biomarkers shown to be sensitive to effects of space radiation in previous studies to identify the associations between sets of lipids in the hypothalamus and amygdala, plasma miRNA, and behavioral and cognitive measures.

## Materials and methods

### Mice and study design

BALB/c and C3H female and male mice and their F2 hybrid progeny were irradiated with ^28^Si ions (350 MeV/n, 0.2 Gy) at the NASA Space Radiation Laboratory (NSRL) at Brookhaven National Laboratory (BNL, Long Island, NY) on October 4, 2016. As this study was part of a cancer-focused larger project, the rationale for using the BALB/c and C3H strains was based on their differential susceptibility to radiation-induced tumors. One week after irradiation or sham-irradiation, the mice were shipped to Colorado State University (Fort Collins, CO) and tested there for behavioral and cognitive performance 1, 6, and 12 months following irradiation or sham-irradiation. The number of mice in the experimental groups were: (1) 48 BALB/c [24 sham-irradiated and 24 irradiated; 8 of each treatment and sex were euthanized and tissues dissected at each time point (TP)]; (2) 48 C3H (24 sham-irradiated and 24 irradiated; 8 of each treatment and sex were euthanized and tissues dissected at each TP); (3) 32 ^28^Si ion irradiated F2 mice tested (behaviorally naive F2 mice tested at each TP; none of the F2 mice were killed following behavioral testing. The F1 mice, [BALB/c × C3H]F1, were generated from matings of BALB/c females and C3H males. All of the mice, parental and F2, were irradiated at 10 to 12 weeks of age. Breeding mice were not irradiated, unmated BALB/c and C3H were. Because the parental strains are inbred, we could irradiate age matched parental and F2 mice at the same time. For tracking purposes, no white mice were included with the F2 mice. The plasma of the mice was collected for analysis of miRNA levels. The hypothalamus and amygdala were dissected for lipidomics analyses and analyses of levels of select biomarkers shown to be sensitive to effects of space radiation in previous studies. All procedures and methods were performed in accordance with the relevant guidelines and regulations, followed the ARRIVE guidelines, and were approved by the IACUC of Colorado State University.

### Behavioral and cognitive testing

All behavioral and cognitive testing was conducted at CSU by experimenters who were blinded to radiation dose. Each mouse was tested separately over 6 consecutive testing days. Starting on day 1, exploratory activity and measures of anxiety were assessed in the open field for 2 days. The mice were tested for novel object recognition during the two subsequent days. The following 2 days, mice were tested contextual and cued fear learning and memory. Body weights were recorded the following week.

### Open field and novel object recognition

The open field was used to assess measures of anxiety, locomotor, and exploratory behavior. Mice were singly placed in a lit (average: 4.2 lx) white plastic arena (40.64 cm × 40.64 cm) (TAP Plastics, Portland, Oregon) for five-minute trials, once each day for two consecutive days. Arenas were cleaned with 0.5% acetic acid between each trial. Movement of the mice and durations spent in the center of the arena (center 20 cm square area) were recorded and analyzed using Viewer video tracking software (Biobserve GmbH, Bonn, Germany). After habituation to the arena for two days, which requires hippocampal function^18^, novel object recognition was assessed by placing two identical objects 10 cm apart in the arena on the third day, then replacing one object with a novel object on the fourth day. Trials on the third and fourth days were fifteen minutes each. Videos recorded on day 4 were viewed by experimenters who hand scored durations of time spent exploring each object. Time spent exploring the novel object versus the familiar object on day 4, expressed as a percentage of the total object exploration time in the trial, was used to determine object recognition memory.

### Fear conditioning

Contextual and cued fear conditioning were used to assess hippocampus-dependent contextual associative memory and hippocampus-independent cued associative memory^19^ using near-infrared (NIR) video and automated analysis, and Video Freeze automated scoring software (Med Associates Inc., St. Albans, VT, USA). In the fear conditioning tests, mice learn to associate an environmental context or cue (tone, conditioned stimulus, CS) with a mild foot shock (unconditioned stimulus, US). Upon re-exposure to the training context, or a new environment in which the mice are exposed to a tone that was present during training, associative learning is assessed based on freezing behavior, characterized by absence of all movement besides respiration. On the training day, the mice were individually placed inside a white LED lit (100 lx) fear conditioning chamber with a metal grid floor and allowed to habituate for a 90 s baseline period. This was followed by a 80 dB, 2800 Hz tone (conditioned stimulus (CS) or cue) lasting for 30 s and co-terminating with a 2 s, 0.7 mA foot shock (unconditioned stimulus or US) at 120 s. Five tone-shock pairings were used, with an inter-shock interval (ISI) of 90 s. Measurements of average motion (cm/s) and percentage of time freezing were analyzed during the baseline period (prior to the first tone), and during each subsequent ISI and CS (tone/cue) to assess acquisition of fear memory. Chambers were cleaned between trials with a 0.5% acetic acid solution. The next day, the mice were placed back into the same context as used on the training day, for a single 5-min trial, and freezing behavior was measured in the absence of either tones or shocks to assess contextual associative memory. Three hours later, the mice were placed into a novel context, containing a smooth white plastic covering the wire grid floor, a “tented” black plastic ceiling, and scented with hidden vanilla extract-soaked nestlets. The chambers were cleaned between trials with a 10% isopropanol solution. Each trial consisted of a 90-s baseline, then a 180-s 80 dB, 2800 Hz tone and freezing behavior was analyzed as an indicator of cued associative memory.

### MAP-2, CD68, and BDNF ELISAs

For assessments of cortical MAP-2, CD68, and BDNF levels, the mice were euthanized by cervical dislocation following CO_2_ anesthesia and hippocampal and cortical or hypothalamic and amygdaloid, regions of their brains dissected for separate analyses. For TP1, BDNF levels were analyzed in the hypothalamus, amygdala, and cortex, CD68 levels were analyzed in the hypothalamus, and MAP-2 levels in the cortex. For TP2, BDNF and CD68 levels were analyzed in the hypothalamus and amygdala. For TP3, BDNF and MAP-2 levels were analyzed in the cortex. The brain tissues were homogenized and a protein assay was performed using a BCA kit (Fisher Scientific, Chicago, IL, United States), as described. MyBioSource CD68 (Catalog number MBS2601301) and MAP-2 (Catalog number MBS725632, San Diego, CA, United States) ELISAs and a VWR mouse BDNF (Catalog number 10205-700, Radnor, PA, United States) ELISA were used to determine CD68, MAP-2, and BDNF tissue levels according to the manufacturer instructions. The standard curve was run in duplicate and the samples as single samples. Based on the optical density values read using a SpectraMax iD5 (Molecular Devices, San Jose, CA, United States), the MAP-2, CD68, and BDNF levels in the samples were calculated using GraphPad Prism software (San Diego, CA, United States).

### Lipid isolation

Mouse hypothalamus and amygdala tissue samples were extracted for polar lipid analysis. The entire tissue sample was homogenized in 400 µL of 155 mM ammonium acetate^20^ solution using a Polytron equipped with a micro-generator (10 s × 2, at 15,000 rpm) in 4 mL glass tubes. A 2 µL volume was removed and diluted in 155 mM ammonium acetate for BCA total protein determination. Protein concentrations were standardized to the amount of homogenate to perform the lipid extraction on because the amount of tissue provided varied greatly. A volume corresponding to 200 µg of total protein was transferred to a 2 mL screw cap (Teflon lined) glass vial and 1:1 MeOH:CHCl_3_ (400 µL of each solvent) was added. The MeOH solution contained 2 mM butylated hydroxy toluene (BHT) to prevent lipid oxidation^21^. Samples were then capped and wrapped in parafilm and placed in a sonicating water bath for 30 min. Samples were then transferred to a heating block shaker at 48 °C overnight (parafilm was removed before placing in heating block).

The next day, the sample caps were re-wrapped in parafilm and placed in sonicating water bath for 10 min. After sonication, the parafilm was removed and the vial was centrifuged at 5000*g* for 15 min at room temperature. The supernatant was transferred to a 30 mL glass Corex tube, capped with a piece of aluminum foil and saved for later at room temp. Subsequently, 1:1 MeOH:CHCl_3_ (400 µL of each solvent) was added to the pellet in the vial, vortexed and the vial was centrifuged at 5000*g* for 15 min at room temperature. Supernatant was combined with the previous aliquot in the 30 mL Corex tube from above. The remaining pellet was re-extracted with 1:1 MeOH:CHCl_3_ (400 µL of each solvent). The solvents were added to the pellet in the vial, vortexed and the vial was centrifuged at 5000*g* for 15 min at room temperature. Supernatant was combined with the previous aliquots in the 30 mL Corex tube from above. To the combined supernatants in the Corex tube, 3.3 mL of H_2_O and 1.2 mL of CHCl_3_ was added. The mixture was vortexed and mixed well with aid of a glass pipet. The Corex tube with the combined aliquots was centrifuged at 5000*g* for 20 min at room temperature to produce 2 phases with clear separation. The polar lipids are in the aqueous layer (top layer). This layer was transferred to a 2 mL screw cap glass vial and samples were dried in a SpeedVac. Once dried, sample was taken up into 100 µL of 80% MeOH, 20% H2O with 10 mM NH_4_OAc. A 50 µL aliquot of each sample was transferred an autosampler vial (with insert) for mass spectrometry analysis^22^.

### Mass spectrometry analysis

Samples were analyzed on a Bruker Solarix 12T FT-ICR MS by nanoLC-micro-ESI MS/MS at a flow rate of 400 nL/min. Each sample was on-line separated with an Eksigent nanoLC 2D system over an in-house packed 75 micron i.d. nano-LC column packed with 8 cm of Phenyl hexal resin; 5 µL of sample was injected onto the column. The sample was loaded onto the column and washed for 5 min with 20%/80% A/B solvent. The sample was eluted with a gradient starting at 20%/80% A/B solvent and ramping to 1%/99% A/B solvent over 10 min. 1%/99% A/B solvent was held for 5 min to elute everything from the column. The solvent was then stepped down immediately to 20%/80% A/B solvent and held there for 10 min to re-equilibrate the column for the next sample. The solvent compositions were: Solvent A: 98% H2O, 2% MeOH, with 10 mM NH_4_OAc; Solvent B: 98% MeOH, 2% H_2_O with 10 mM NH_4_OAc)^22^.

### Lipid data analysis and identification

Lipidomics data was processed via Bruker’s Data Analysis 4.0. The SNAP algorithm was implemented for peak picking and charge state determination. Neutral mass peaklists were generated for all ions with a s/n of 5 or greater. Lipid identifications were then assigned to specific *m/z* ions based on accurate mass using database searches with LIPID Maps and Swiss Lipids databases. All lipid identifications for hypothalamus and amygdala samples were performed in a blinded method, i.e. sample i.d. or treatment was unknown during assignment of lipid identifications.

### Sequencing of circulating miRNA from mouse plasma

Circulating miRNA was isolated using 50 µl of mouse plasma. The isolation was carried out using miRCURY RNA Isolation Kit—Biofluids from Exiqon. Synthetic miRNA mimics, UniSP2, UniSP4 and UniSP6 were added before isolation into each sample for QC of sequencing library setup. QIAseq miRNA Library Kit was used to build molecular-barcoding-enabled sequencing libraries. Small RNA sequencing was performed at Qiagen Genomics Service Core using an Illumina NextSeq500 sequencer. The read length was 76 nt, single-end read. The sequence reads were aligned with references genome GRCm38. MiRNAs were annotated according to mirbase_20. The aligned reads were de-barcoded to produce counts of Unique Molecular Index (UMI) corresponding to miRNAs. The UMI counts from each sample were normalized using DEseq2 package from Bioconductor. The data were transformed with vst algorithm and were used for all subsequent analyses.

### Statistical analyses

**Figure 1:**
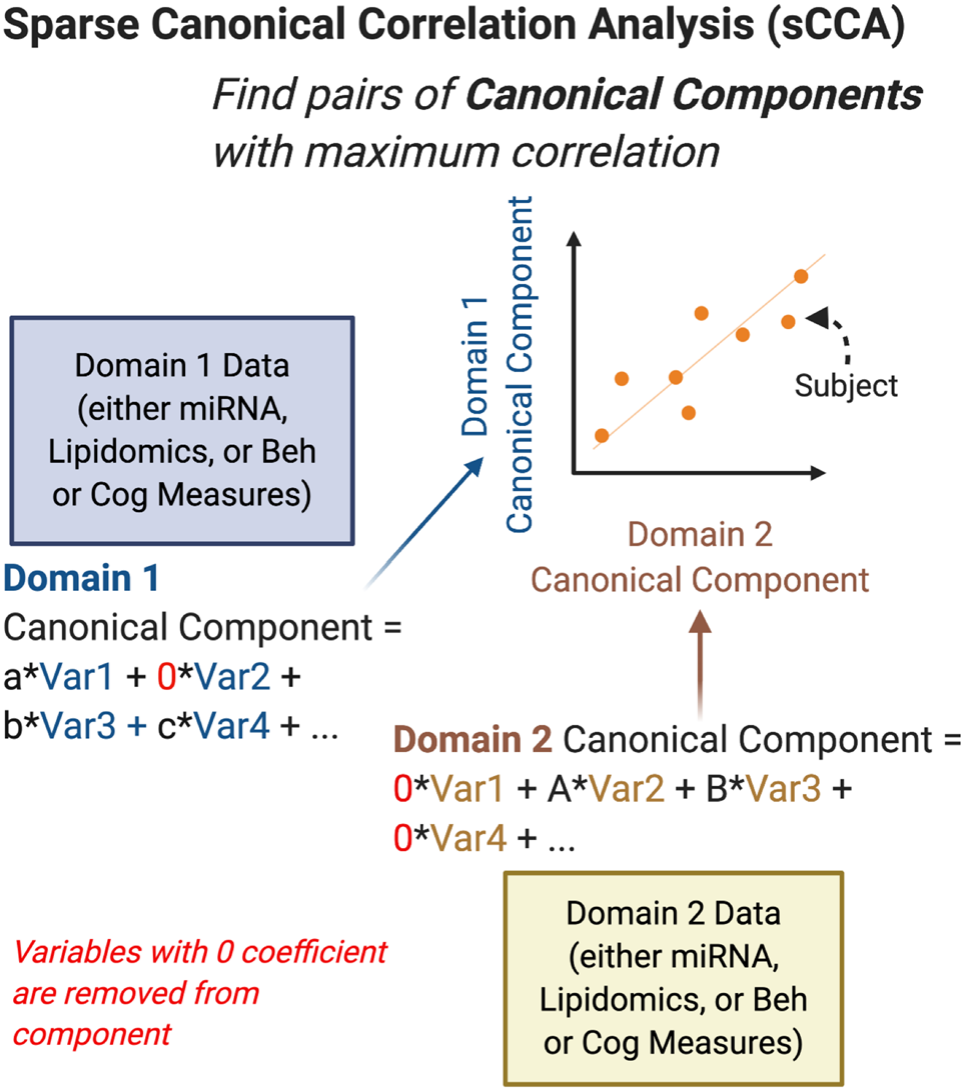
Schematic illustration of the sparse Canonical Correlation Analysis.

To identify the strongest joint associations between sets of lipids, miRNA, and behavioral and cognitive measures, sparse canonical correlation analysis (sCCA)^23^ was performed within irradiation and generation groups (Fig. 1). The sCCA method examines two data matrices (**X** and **Z**, i.e. **X** = miRNA and **Z** = behavioral outcomes) and uses penalized matrix decomposition to find a linear combination within each data set that have the strongest correlation with each other. Sparse CCA is a useful method for evaluating and describing relationships across multiple types of data sets from different modalities. Sparse CCA has been used in many big data applications, including several cognitive neuroscience studies^24^ Sparse CCA was implemented with the PMA package in R^25^ and the lasso (L1) penalty was used to select a subset of variables within each data set to include in the linear combinations. Sparse CCA was performed for each pair of data sets (lipidomics vs miRNA, behavioral, or cognitive, and miRNA vs behavioral or cognitive) within each radiation and generation subgroup at each time point separately. The number of pairs of combinations (canonical vectors) was chosen to be 3 and the Pearson correlations of each of the 3 pairs of linear components (denoted K1, K2, K3) are presented in the figures. In the case of missing data in behavioral or cognitive data (2 mice for time point 3 behavioral data; 9 mice at time point 2 for cognitive data; 4 mice for time point 3 for cognitive data), only complete cases were included in the analyses. For missingness (non-detection) in lipidomics data, lipid datasets were restricted to sets of lipids with < 20% missingness within each subgroup, and complete case data was used. Lipids were considered missing if the signal to noise ratio was less than 5. Due to the skew and non-normality of many of the cognitive and behavioral measures, normalizing transformation was performed for the sCCA analyses to improve robustness to outliers. For each variable, the optimal normalizing transformation from a set of normalizations (Yeo-Johnson transformation, the Box Cox transformation (if the data is positive), the log_10(x + a) transformation, the square-root (x + a) transformation, and the arcsinh transformation) was selected based on the Pearson P test statistic for normality using the bestNormalize R package (version 1.6.0^26^) under default settings.

In addition to the analyses described above, a Lasso regression analysis^27^ was performed. The input dependent variables were the biological condition of the mice (age, strain, sex, and radiation condition) and the miRNA expression or lipid abundance. The independent predictors or response variables were either the behavioral and cognitive performance measures or the brain lipid level. The subject biological conditions were encoded into a binary matrix. Any missing value in the continuous variables (miRNA or lipidomics level) was inferred with the mean value across the samples and then scaled to have 0 means. Separate lasso regression models were also fit within irradiation and sham groups to assess possible effect modification of irradiation on other factors. Lasso regression coefficients were estimated using the cv.glmnet() function in the glmnet package in R^28^ with 100-fold cross-validation for lasso penalty (alpha = 1). Lasso and sparse CCA methods were implemented in these data sets due to the high dimensionality of the measures and outcomes compared to the sample size. Neither method results in p-values that need to be corrected for multiple testing and instead we focused on the strongest correlations and associations observed in the data after variable selection to remove noise and noninformative predictors.

miRNA enrichment (over-representation) analyses for both the lasso regression and sCCA results were performed with the miRNA Enrichment Analysis and Annotation Tool (miEAA 2.0^29^ with Benjamini–Hochberg False Discovery Rate threshold 5% within each enrichment analysis and within each pathway category. Databases queried for enrichment included Gene Ontology (GO) terms, KEGG pathways, miRWalk pathway, target genes, and published diseases.

## Results

### Effects of ^28^Si ion irradiation on behavioral and cognitive measures, BDNF levels in the hypothalamus and amygdala at TP1, and CD-68 levels in the amygdala at TP2

While this is a biomarker study and the study was not designed to assess effects of ^28^Si ion irradiation on behavioral and cognitive measures, to appreciate what behavioral and cognitive changes are caused by ^28^Si ion irradiation, Supplementary Fig. 1 illustrates significant effects and trends towards significant effects of ^28^Si ion irradiation on behavioral and cognitive measures of the parental strains at the three time points. Supplementary Fig. 2 illustrates significant effects of ^28^Si ion irradiation on BDNF levels in the hypothalamus and amygdala of C3H mice at the first time point (TP1) and the significant effects of ^28^Si ion irradiation on CD-68 levels in the amygdala, but not hypothalamus of C3H mice at the second time point (TP2).

### Associations between plasma miRNAs and cognitive measures

**Figure 2:**
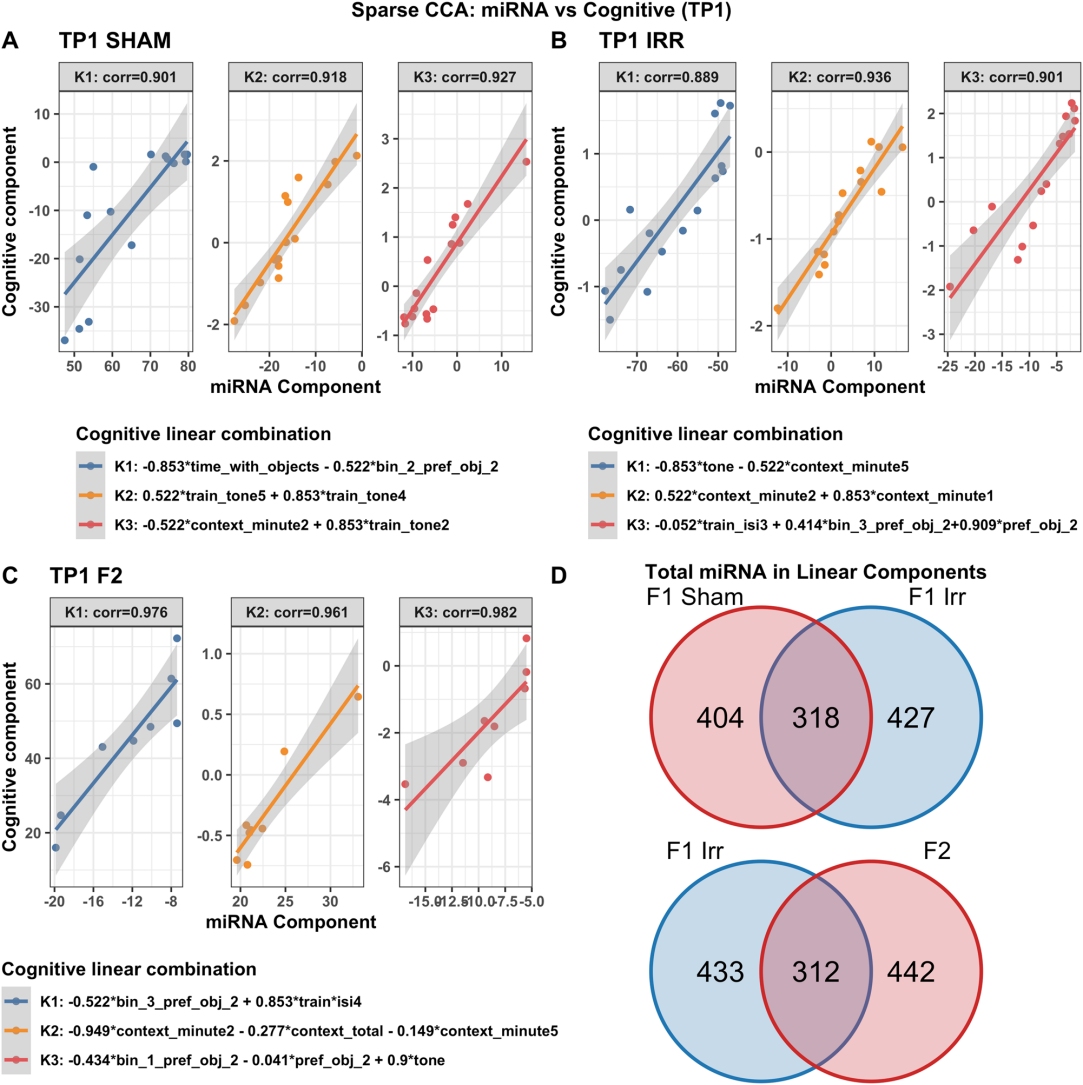
Correlations between plasma miRNAs and cognitive measures at TP1. Results from sCCA analyses of plasma miRNAs and cognitive measures at time point 1. Three analyses are shown: sham mice (**A**), F1 irradiated mice (**B**), F2 irradiated mice (**C**). The top 3 canonical vector components (linear combinations within each data set denoted K1, K2, K3) with the strongest correlations are detected and presented in the three panels of each analyses. For each component, the explicit linear combinations of cognitive measures are shown in the legend. Pearson’s correlation for each component is shown above each panel. The scatter plots show the linear combination for miRNA on the x-axis and the linear combination of cognitive component in the Y axis, along with the best fit linear line and confidence band (grey shaded area). Venn diagrams show the number and overlap of miRNAs represented collectively across the three canonical components within each irradiation/generation analysis.

Table 1 illustrates the most important cognitive and behavioral measures used for the integrated analysis. Different but overlapping sets of miRNAs in plasma were found to be correlated with cognitive measures in sham and irradiated mice at the first (TP1, Fig. 2), second (TP2, Supplementary Fig. 3), and third (TP3, Supplementary Fig. 4) time points. At TP1, the radiation condition revealed pathways involved in Rett syndrome, multiple sclerosis, Alzheimer’s disease, and medulla blastoma (Supplementary Fig. 5). In addition, different but overlapping sets of miRNAs in plasma were found to be correlated with cognitive measures in irradiated mice of the parental strains and F2 mice at the first (TP1, Fig. 2), second (TP2, Supplementary Fig. 3), and third (TP3, Supplementary Fig. 4) time points. At TP1, the F2 irradiated group revealed pathways involved in mitochondria function and exosomes (Supplementary Fig. 5). At TP2, the radiation condition revealed pathways involved in Huntington disease and Down syndrome (Supplementary Fig. 6) while the F2 irradiated group revealed pathways involves in Down syndrome and synapse. At TP3, the radiation condition revealed pathways involved in Rett syndrome and Alzheimer’s disease (Supplementary Fig. 7).

**Table 1:** Cognitive and behavioral measures used for the integrated analyses.

**Cognitive measures**
1. *preference_obj_2*, *bin_1-3_preference_obj_2*: percent time spent exploring the novel object, either over the total 15 min of the test or in any of the 3 5-min bins
2. *time_with_objects*: total time spent exploring both objects on day 2 of the object recognition test
3. *context_total*, *context_minute1-5*: percent time freezing in the contextual memory test, either as the total over the 5 min test or in individual minutes. This is considered a memory measure
4. *tone*: percent time spent freezing during the tone in the cued memory test
5. *train_isi1-4*: increase in percent time spent freezing between the tone-shock pairing during training of the fear conditioning test. This is considered a learning measure
6. *train_tone1-5*: increase in percent time freezing during the tones
activity decrease in the open field on day 2 as compared to day 1 (repeated measures or delta). These data are not in the current excel file yet and will be added later
**Behavioral measures**
1. *ofd1_fecal, ofd2_fecal, nod1_fecal, nod2_fecal*: fecal boli on day 1 and 2 of the open field and day 1 and day 2 of the object recognition test
2. *fc_cued_percent_time_frz_baseline*: percent freezing during the baseline in the cued memory test

### Associations between plasma miRNAs and behavioral measures

**Figure 3:**
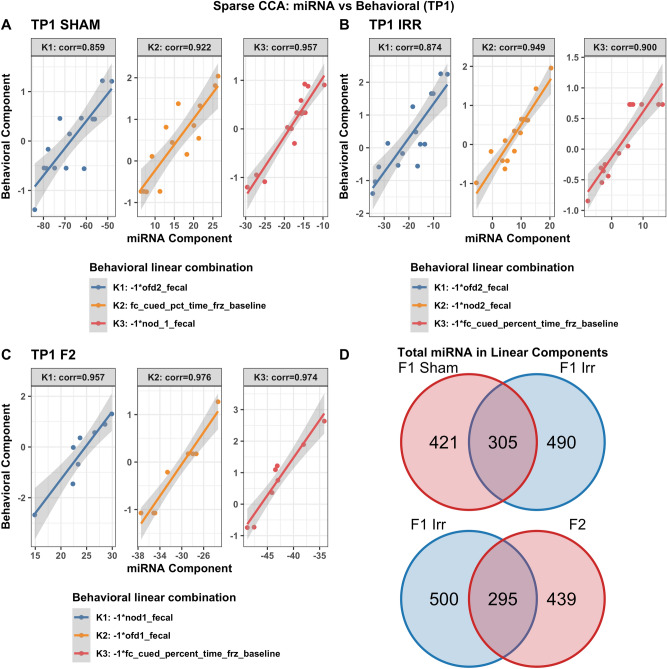
Correlations between plasma miRNAs and behavioral measures at TP1. Results from sCCA analyses of plasma miRNAs and behavioral measures at time point 1. Three analyses are shown: sham mice (**A**), F1 irradiated mice (**B**), F2 irradiated mice (**C**). The top 3 canonical vector components (linear combinations within each data set denoted K1, K2, K3) with the strongest correlations are detected and presented in the three panels of each analyses. For each component, the explicit linear combinations of behavioral measures are shown in the legend. Pearson’s correlation for each component is shown above each panel. The scatter plots show the linear combination for miRNA on the x-axis and the linear combination of behavioral component in the Y axis, along with the best fit linear line and confidence band (grey shaded area). Venn diagrams show the number and overlap of miRNAs represented collectively across the three canonical components within each irradiation/generation analysis.

A similar pattern was seen for relationships with behavioral measures. Different but overlapping sets of miRNAs in plasma were found to be correlated with behavioral measures in sham and irradiated mice at the first (TP1, Fig. 3), second (TP2, Supplementary Fig. 8), and third (TP3, Supplementary Fig. 9) time points. At TP1 (Supplementary Fig. 10) and TP3 (Supplementary Fig. 12), the radiation condition revealed pathways involved in Alzheimer’s disease, peripheral nerve injury, multiple sclerosis and an animal model of multiple sclerosis (EAE), and medulla blastoma. In contrast, at TP2, the radiation condition did not reveal any of those pathways (Supplementary Fig. 11).

### Associations between plasma miRNAs and lipids in the hypothalamus and amygdala

**Figure 4:**
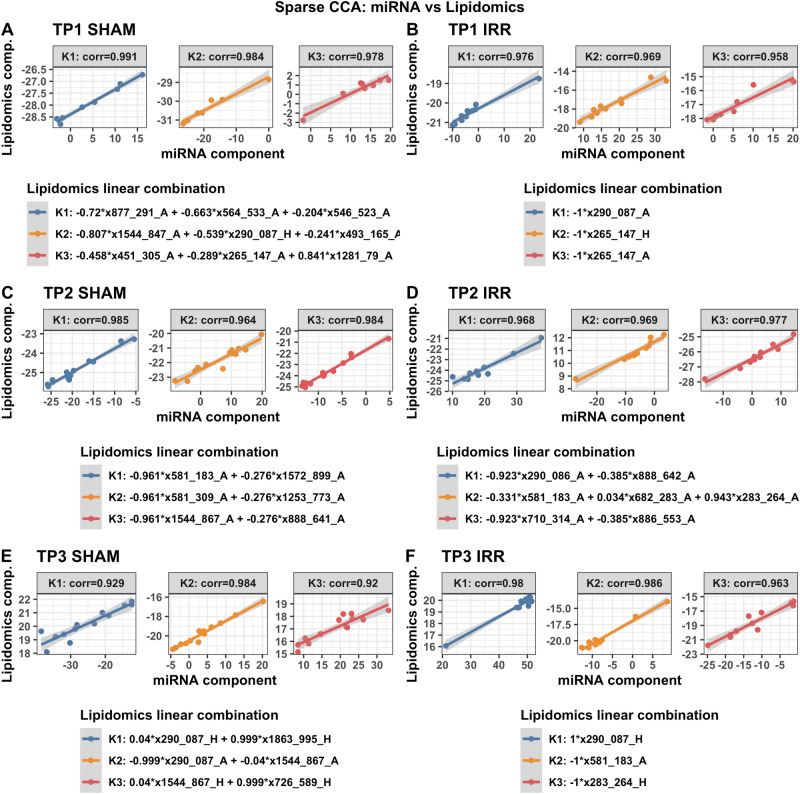
Correlations between plasma miRNAs and lipids in select brain regions. Results from sCCA analyses of plasma miRNAs and lipidomics at time points 1, 2 and 3. Two analyses for each time point are shown: F1 irradiated mice (top row) and F1 sham mice (bottom row). The top 3 canonical vector components (linear combinations within each data set denoted K1, K2, K3) with the strongest correlations are detected and presented in the three panels of each analyses. For each component, the explicit linear combinations of lipidomics measures are shown in the legend. Pearson’s correlation for each component is shown above each panel. The scatter plots show the linear combination for miRNA on the x-axis and the linear combination of lipidomics component in the Y axis, along with the best fit linear line and confidence band (grey shaded area).

We next assessed relationships between plasma miRNAs and lipids in the hypothalamus and amygdala at the three time points. Different but overlapping sets of miRNAs in plasma were found to be correlated with lipids in sham and irradiated mice at the first (TP1, Fig. 4A,B), second (TP2, Fig. 4C,D), and third (TP3, Fig. 4D,E) time points. At TP1, plasma miRNAs in irradiated mice that were correlated with lipids in the hypothalamus and amygdala were enriched in pathways related to longevity and cancer that were not seen in sham-irradiated mice (Supplementary Fig. 13). At TP2, plasma miRNAs in irradiated mice that were correlated with lipids in the hypothalamus and amygdala were also enriched in pathways related cancer, including pancreatic carcinoma, lung neoplasm, neoplasm metastasis, and adenocarcinoma that were not seen in sham-irradiated mice (Supplementary Fig. 14). At TP3, plasma miRNAs in sham-irradiated mice that were correlated with lipids in the hypothalamus and amygdala were enriched in pathways related to Rett syndrome, seizures, Alzheimer’s disease, peripheral nerve injuries, and medulloblastoma (Supplementary Fig. 15). The lipids involved in these plasma miRNA-lipids relationships in sham-irradiated and irradiated mice at the three time points are illustrated in Table 2. Many of these lipids are involved in inflammatory responses, mitochondrial function, neuronal function and the insulin response.

**Table 2:** Lipids correlated with miRNAs in sCCA analyses. Lipids found to be correlated with miRNAs in all sCCA analyses relating lipidomics with miRNA data, with corresponding set of miRNAs found to be correlated with each individual lipid. Lipid masses are specified with “_A” denoting amygdala tissue and “_H” denoting hippocampus tissue.

Mass	Lipid_name	miRNAs correlated in sCCA analyses
265.147	x265_147_A	mmu_let_7a_2_3p,mmu_let_7b_3p,mmu_let_7c_1_3p,mmu_let_7d_5p,mmu_let_7e_5p,mmu_let_7f_1_3p,mmu_let_7g_3p,mmu_let_7i_5p,mmu_let_7j,mmu_let_7k,mmu_mi_r_100_3p,mmu_mi_r_103_1_5p,mmu_mi_r_106a_3p,mmu_mi_r_106a_5p,mmu_mi_r_106b_3p,mmu_mi_r_106b_5p,mmu_mi_r_1188_3p,mmu_mi_r_1191a,mmu_mi_r_1195,mmu_mi_r_1198_5p,mmu_mi_r_1199_3p,mmu_mi_r_1224_5p,mmu_mi_r_124_5p,mmu_mi_r_1247_5p,mmu_mi_r_1258_3p,mmu_mi_r_125b_2_3p,mmu_mi_r_127_3p,mmu_mi_r_129_1_3p,mmu_mi_r_129_5p,mmu_mi_r_1306_3p,mmu_mi_r_130a_3p,mmu_mi_r_130b_5p,mmu_mi_r_133a_3p,mmu_mi_r_133b_3p,mmu_mi_r_133c,mmu_mi_r_135a_2_3p,mmu_mi_r_135a_5p,mmu_mi_r_137_5p,mmu_mi_r_138_5p,mmu_mi_r_140_3p,mmu_mi_r_140_5p,mmu_mi_r_141_5p,mmu_mi_r_144_3p,mmu_mi_r_145b,mmu_mi_r_146a_3p,mmu_mi_r_148a_3p,mmu_mi_r_149_3p,mmu_mi_r_150_3p,mmu_mi_r_150_5p,mmu_mi_r_151_5p,mmu_mi_r_152_3p,mmu_mi_r_154_5p,mmu_mi_r_15a_3p,mmu_mi_r_15b_3p,mmu_mi_r_15b_5p,mmu_mi_r_16_1_3p,mmu_mi_r_16_2_3p,mmu_mi_r_16_5p,mmu_mi_r_17_3p,mmu_mi_r_17_5p,mmu_mi_r_181b_1_3p,mmu_mi_r_181b_2_3p,mmu_mi_r_181b_5p,mmu_mi_r_181c_5p,mmu_mi_r_181d_5p,mmu_mi_r_1839_5p,mmu_mi_r_1843a_5p,mmu_mi_r_1843b_5p,mmu_mi_r_185_3p,mmu_mi_r_186_5p,mmu_mi_r_187_3p,mmu_mi_r_1894_5p,mmu_mi_r_1897_5p,mmu_mi_r_1898,mmu_mi_r_1899,mmu_mi_r_18a_3p,mmu_mi_r_18b_5p,mmu_mi_r_1905,mmu_mi_r_1907,mmu_mi_r_190a_3p,mmu_mi_r_1928,mmu_mi_r_1930_3p,mmu_mi_r_1931,mmu_mi_r_1936,mmu_mi_r_1938,mmu_mi_r_1940,mmu_mi_r_1941_5p,mmu_mi_r_1942,mmu_mi_r_1946b,mmu_mi_r_1947_3p,mmu_mi_r_1948_3p,mmu_mi_r_1952,mmu_mi_r_1953,mmu_mi_r_1955_3p,mmu_mi_r_1956,mmu_mi_r_1958,mmu_mi_r_195a_5p,mmu_mi_r_1960,mmu_mi_r_1961,mmu_mi_r_1962,mmu_mi_r_1964_3p,mmu_mi_r_1966_5p,mmu_mi_r_1968_5p,mmu_mi_r_196a_5p,mmu_mi_r_196b_5p,mmu_mi_r_1971,mmu_mi_r_1982_3p,mmu_mi_r_199a_5p,mmu_mi_r_199b_5p,mmu_mi_r_19b_1_5p,mmu_mi_r_1b_3p,mmu_mi_r_200a_5p,mmu_mi_r_202_3p,mmu_mi_r_203_3p,mmu_mi_r_204_5p,mmu_mi_r_206_5p,mmu_mi_r_207,mmu_mi_r_208a_3p,mmu_mi_r_20a_5p,mmu_mi_r_20b_5p,mmu_mi_r_210_5p,mmu_mi_r_211_5p,mmu_mi_r_212_5p,mmu_mi_r_2137,mmu_mi_r_2139,mmu_mi_r_214_5p,mmu_mi_r_216b_3p,mmu_mi_r_217_3p,mmu_mi_r_2183,mmu_mi_r_219a_2_3p,mmu_mi_r_219c_3p,mmu_mi_r_22_5p,mmu_mi_r_221_3p,mmu_mi_r_222_3p,mmu_mi_r_222_5p,mmu_mi_r_223_3p,mmu_mi_r_223_5p,mmu_mi_r_224_3p,mmu_mi_r_25_3p,mmu_mi_r_26a_1_3p,mmu_mi_r_26b_3p,mmu_mi_r_27a_5p,mmu_mi_r_27b_5p,mmu_mi_r_2861,mmu_mi_r_28b,mmu_mi_r_28c,mmu_mi_r_290a_3p,mmu_mi_r_290b_3p,mmu_mi_r_291a_3p,mmu_mi_r_293_5p,mmu_mi_r_294_5p,mmu_mi_r_295_3p,mmu_mi_r_297a_3p_mmu_mi_r_297b_3p_mmu_mi_r_297c_3p,mmu_mi_r_298_3p,mmu_mi_r_299b_3p,mmu_mi_r_29b_1_5p,mmu_mi_r_300_5p,mmu_mi_r_301a_5p,mmu_mi_r_301b_3p,mmu_mi_r_302a_5p,mmu_mi_r_3057_3p,mmu_mi_r_3057_5p,mmu_mi_r_3058_5p,mmu_mi_r_3059_3p,mmu_mi_r_3059_5p,mmu_mi_r_3061_5p,mmu_mi_r_3064_5p,mmu_mi_r_3067_3p,mmu_mi_r_3070_3p,mmu_mi_r_3071_3p,mmu_mi_r_3072_3p,mmu_mi_r_3073a_3p,mmu_mi_r_3076_5p,mmu_mi_r_3077_3p,mmu_mi_r_3078_5p,mmu_mi_r_3080_3p,mmu_mi_r_3082_5p,mmu_mi_r_3084_3p,mmu_mi_r_3085_3p,mmu_mi_r_3085_5p,mmu_mi_r_3087_3p,mmu_mi_r_3087_5p,mmu_mi_r_3088_3p,mmu_mi_r_3089_5p,mmu_mi_r_3090_3p,mmu_mi_r_3092_3p,mmu_mi_r_3097_3p,mmu_mi_r_3098_3p,mmu_mi_r_3098_5p,mmu_mi_r_30f.,mmu_mi_r_3100_3p,mmu_mi_r_3100_5p,mmu_mi_r_3102_5p,mmu_mi_r_3103_3p,mmu_mi_r_3103_5p,mmu_mi_r_3104_3p,mmu_mi_r_3104_5p,mmu_mi_r_3105_3p,mmu_mi_r_3105_5p,mmu_mi_r_3109_5p,mmu_mi_r_3154,mmu_mi_r_324_3p,mmu_mi_r_324_5p,mmu_mi_r_325_5p,mmu_mi_r_328_3p,mmu_mi_r_329_5p,mmu_mi_r_331_3p,mmu_mi_r_331_5p,mmu_mi_r_338_5p,mmu_mi_r_340_5p,mmu_mi_r_341_3p,mmu_mi_r_342_3p,mmu_mi_r_344_3p,mmu_mi_r_344b_3p,mmu_mi_r_344c_5p,mmu_mi_r_344d_1_5p,mmu_mi_r_344d_3_5p,mmu_mi_r_344f_3p,mmu_mi_r_344f_5p,mmu_mi_r_344i,mmu_mi_r_346_5p,mmu_mi_r_3472,mmu_mi_r_3474,mmu_mi_r_3475_3p,mmu_mi_r_3475_5p,mmu_mi_r_34a_3p,mmu_mi_r_34c_3p,mmu_mi_r_3544_3p,mmu_mi_r_3547_3p,mmu_mi_r_3552,mmu_mi_r_3572_3p,mmu_mi_r_361_3p,mmu_mi_r_3618_3p,mmu_mi_r_374c_5p,mmu_mi_r_376a_5p,mmu_mi_r_376c_5p,mmu_mi_r_377_3p,mmu_mi_r_378a_3p,mmu_mi_r_378a_5p,mmu_mi_r_378b,mmu_mi_r_378c,mmu_mi_r_378d,mmu_mi_r_379_5p,mmu_mi_r_380_3p,mmu_mi_r_381_3p,mmu_mi_r_381_5p,mmu_mi_r_382_3p,mmu_mi_r_3964,mmu_mi_r_3966,mmu_mi_r_3968,mmu_mi_r_3969,mmu_mi_r_3970,mmu_mi_r_409_5p,mmu_mi_r_410_5p,mmu_mi_r_411_3p,mmu_mi_r_412_3p,mmu_mi_r_421_3p,mmu_mi_r_421_5p,mmu_mi_r_423_3p,mmu_mi_r_423_5p,mmu_mi_r_425_5p,mmu_mi_r_433_5p,mmu_mi_r_434_3p,mmu_mi_r_449a_3p,mmu_mi_r_449a_5p,mmu_mi_r_450a_5p,mmu_mi_r_451a,mmu_mi_r_452_3p,mmu_mi_r_453,mmu_mi_r_465c_5p,mmu_mi_r_465d_3p,mmu_mi_r_465d_5p,mmu_mi_r_466a_3p_mmu_mi_r_466e_3p,mmu_mi_r_466b_3p_mmu_mi_r_466c_3p_mmu_mi_r_466p_3p,mmu_mi_r_466d_3p,mmu_mi_r_466h_3p,mmu_mi_r_466j,mmu_mi_r_466k,mmu_mi_r_466l_3p,mmu_mi_r_466q,mmu_mi_r_467b_3p,mmu_mi_r_467f.,mmu_mi_r_467g,mmu_mi_r_470_3p,mmu_mi_r_471_5p,mmu_mi_r_483_3p,mmu_mi_r_483_5p,mmu_mi_r_485_3p,mmu_mi_r_485_5p,mmu_mi_r_486a_3p,mmu_mi_r_486a_5p,mmu_mi_r_486b_3p,mmu_mi_r_486b_5p,mmu_mi_r_487b_3p,mmu_mi_r_489_5p,mmu_mi_r_490_3p,mmu_mi_r_491_5p,mmu_mi_r_495_5p,mmu_mi_r_496a_3p,mmu_mi_r_499_3p,mmu_mi_r_499_5p,mmu_mi_r_5100,mmu_mi_r_5106,mmu_mi_r_5107_3p,mmu_mi_r_5108,mmu_mi_r_511_3p,mmu_mi_r_5119,mmu_mi_r_5120,mmu_mi_r_5122,mmu_mi_r_5124b,mmu_mi_r_5128,mmu_mi_r_5130,mmu_mi_r_5131,mmu_mi_r_541_5p,mmu_mi_r_544_3p,mmu_mi_r_544_5p,mmu_mi_r_551b_3p,mmu_mi_r_5617_3p,mmu_mi_r_5624_3p,mmu_mi_r_5625_3p,mmu_mi_r_5625_5p,mmu_mi_r_5709_3p,mmu_mi_r_590_3p,mmu_mi_r_598_5p,mmu_mi_r_615_3p,mmu_mi_r_6237,mmu_mi_r_6238,mmu_mi_r_6239,mmu_mi_r_6336,mmu_mi_r_6337,mmu_mi_r_6340,mmu_mi_r_6344,mmu_mi_r_6345,mmu_mi_r_6348,mmu_mi_r_6349,mmu_mi_r_6350,mmu_mi_r_6351,mmu_mi_r_6354,mmu_mi_r_6358,mmu_mi_r_6360,mmu_mi_r_6364,mmu_mi_r_6365,mmu_mi_r_6369,mmu_mi_r_6371,mmu_mi_r_6372,mmu_mi_r_6378,mmu_mi_r_6386,mmu_mi_r_6387,mmu_mi_r_6390,mmu_mi_r_6392_5p,mmu_mi_r_6394,mmu_mi_r_6396,mmu_mi_r_6398,mmu_mi_r_6400,mmu_mi_r_6403,mmu_mi_r_6404,mmu_mi_r_6406,mmu_mi_r_6408,mmu_mi_r_6409,mmu_mi_r_6410,mmu_mi_r_6411,mmu_mi_r_6412,mmu_mi_r_6414,mmu_mi_r_6415,mmu_mi_r_6416_5p,mmu_mi_r_6417,mmu_mi_r_6418_3p,mmu_mi_r_6419,mmu_mi_r_652_3p,mmu_mi_r_652_5p,mmu_mi_r_6537_3p,mmu_mi_r_6540_3p,mmu_mi_r_6540_5p,mmu_mi_r_665_3p,mmu_mi_r_668_3p,mmu_mi_r_669b_3p,mmu_mi_r_669c_3p,mmu_mi_r_669c_5p,mmu_mi_r_669e_3p,mmu_mi_r_669e_5p,mmu_mi_r_669g,mmu_mi_r_669h_3p,mmu_mi_r_670_3p,mmu_mi_r_6715_3p,mmu_mi_r_672_3p,mmu_mi_r_673_5p,mmu_mi_r_674_5p,mmu_mi_r_675_5p,mmu_mi_r_676_3p,mmu_mi_r_676_5p,mmu_mi_r_6769b_3p,mmu_mi_r_679_3p,mmu_mi_r_679_5p,mmu_mi_r_680,mmu_mi_r_686,mmu_mi_r_687,mmu_mi_r_6896_5p,mmu_mi_r_6904_5p,mmu_mi_r_6905_3p,mmu_mi_r_6906_3p,mmu_mi_r_6908_5p,mmu_mi_r_6909_5p,mmu_mi_r_6910_3p,mmu_mi_r_6914_5p,mmu_mi_r_6916_3p,mmu_mi_r_6917_3p,mmu_mi_r_6919_3p,mmu_mi_r_6919_5p,mmu_mi_r_6921_3p,mmu_mi_r_6922_3p,mmu_mi_r_6924_5p,mmu_mi_r_6925_3p,mmu_mi_r_6925_5p,mmu_mi_r_6928_5p,mmu_mi_r_693_3p,mmu_mi_r_6930_3p,mmu_mi_r_6930_5p,mmu_mi_r_6932_3p,mmu_mi_r_6933_3p,mmu_mi_r_6934_3p,mmu_mi_r_6934_5p,mmu_mi_r_6936_5p,mmu_mi_r_6937_3p,mmu_mi_r_6938_5p,mmu_mi_r_6940_3p,mmu_mi_r_6941_3p,mmu_mi_r_6941_5p,mmu_mi_r_6942_5p,mmu_mi_r_6943_5p,mmu_mi_r_6948_5p,mmu_mi_r_6949_5p,mmu_mi_r_695,mmu_mi_r_6952_3p,mmu_mi_r_6953_3p,mmu_mi_r_6954_3p,mmu_mi_r_6954_5p,mmu_mi_r_6955_3p,mmu_mi_r_6959_3p,mmu_mi_r_6959_5p,mmu_mi_r_6960_3p,mmu_mi_r_6961_5p,mmu_mi_r_6962_3p,mmu_mi_r_6962_5p,mmu_mi_r_6964_5p,mmu_mi_r_6966_3p,mmu_mi_r_6970_3p,mmu_mi_r_6970_5p,mmu_mi_r_6973a_5p,mmu_mi_r_6976_3p,mmu_mi_r_6977_3p,mmu_mi_r_6977_5p,mmu_mi_r_6979_3p,mmu_mi_r_6979_5p,mmu_mi_r_698_3p,mmu_mi_r_698_5p,mmu_mi_r_6980_3p,mmu_mi_r_6980_5p,mmu_mi_r_6982_5p,mmu_mi_r_6983_3p,mmu_mi_r_6983_5p,mmu_mi_r_6984_5p,mmu_mi_r_6986_3p,mmu_mi_r_6986_5p,mmu_mi_r_6991_5p,mmu_mi_r_6992_3p,mmu_mi_r_6992_5p,mmu_mi_r_6994_3p,mmu_mi_r_6996_3p,mmu_mi_r_6997_3p,mmu_mi_r_700_5p,mmu_mi_r_7000_3p,mmu_mi_r_7000_5p,mmu_mi_r_7004_5p,mmu_mi_r_7005_5p,mmu_mi_r_7006_5p,mmu_mi_r_7009_3p,mmu_mi_r_7012_3p,mmu_mi_r_7016_3p,mmu_mi_r_7016_5p,mmu_mi_r_7017_3p,mmu_mi_r_7018_3p,mmu_mi_r_7019_5p,mmu_mi_r_702_5p,mmu_mi_r_7021_5p,mmu_mi_r_7023_3p,mmu_mi_r_7024_3p,mmu_mi_r_7025_3p,mmu_mi_r_7025_5p,mmu_mi_r_7029_5p,mmu_mi_r_7031_3p,mmu_mi_r_7033_3p,mmu_mi_r_7034_3p,mmu_mi_r_7035_3p,mmu_mi_r_7036a_3p,mmu_mi_r_7036a_5p,mmu_mi_r_7038_5p,mmu_mi_r_7041_5p,mmu_mi_r_7042_5p,mmu_mi_r_7043_3p,mmu_mi_r_7044_5p,mmu_mi_r_7046_5p,mmu_mi_r_7049_3p,mmu_mi_r_7050_5p,mmu_mi_r_7051_5p,mmu_mi_r_7054_5p,mmu_mi_r_7055_3p,mmu_mi_r_7057_5p,mmu_mi_r_7059_3p,mmu_mi_r_7059_5p,mmu_mi_r_7060_5p,mmu_mi_r_7062_3p,mmu_mi_r_7063_5p,mmu_mi_r_7064_3p,mmu_mi_r_7064_5p,mmu_mi_r_7065_3p,mmu_mi_r_7065_5p,mmu_mi_r_7067_3p,mmu_mi_r_7068_5p,mmu_mi_r_7069_3p,mmu_mi_r_7070_3p,mmu_mi_r_7073_5p,mmu_mi_r_7074_3p,mmu_mi_r_7075_5p,mmu_mi_r_7076_5p,mmu_mi_r_7077_3p,mmu_mi_r_7077_5p,mmu_mi_r_7079_5p,mmu_mi_r_708_3p,mmu_mi_r_7080_5p,mmu_mi_r_7082_3p,mmu_mi_r_7082_5p,mmu_mi_r_7085_5p,mmu_mi_r_7087_3p,mmu_mi_r_7088_5p,mmu_mi_r_7091_5p,mmu_mi_r_7093_5p,mmu_mi_r_7094_3p,mmu_mi_r_7094b_2_5p,mmu_mi_r_7115_5p,mmu_mi_r_7117_5p,mmu_mi_r_7118_3p,mmu_mi_r_712_5p,mmu_mi_r_717,mmu_mi_r_718,mmu_mi_r_721,mmu_mi_r_7211_3p,mmu_mi_r_7212_5p,mmu_mi_r_7215_5p,mmu_mi_r_7218_3p,mmu_mi_r_7221_3p,mmu_mi_r_7222_3p,mmu_mi_r_7223_5p,mmu_mi_r_7228_5p,mmu_mi_r_7230_3p,mmu_mi_r_7232_3p,mmu_mi_r_7235_5p,mmu_mi_r_7236_3p,mmu_mi_r_7237_5p,mmu_mi_r_7239_3p,mmu_mi_r_7239_5p,mmu_mi_r_7240_3p,mmu_mi_r_7241_3p,mmu_mi_r_7242_3p,mmu_mi_r_7242_5p,mmu_mi_r_743a_5p,mmu_mi_r_744_3p,mmu_mi_r_744_5p,mmu_mi_r_760_3p,mmu_mi_r_7646_5p,mmu_mi_r_7647_3p,mmu_mi_r_7648_3p,mmu_mi_r_7651_3p,mmu_mi_r_7651_5p,mmu_mi_r_7652_3p,mmu_mi_r_7655_3p,mmu_mi_r_7656_3p,mmu_mi_r_7656_5p,mmu_mi_r_7657_5p,mmu_mi_r_7658_5p,mmu_mi_r_7663_3p,mmu_mi_r_7663_5p,mmu_mi_r_7664_3p,mmu_mi_r_7666_5p,mmu_mi_r_7667_3p,mmu_mi_r_7670_5p,mmu_mi_r_7676_3p,mmu_mi_r_7677_3p,mmu_mi_r_7678_3p,mmu_mi_r_7679_5p,mmu_mi_r_7680_5p,mmu_mi_r_7683_3p,mmu_mi_r_7684_3p,mmu_mi_r_7684_5p,mmu_mi_r_7687_3p,mmu_mi_r_7687_5p,mmu_mi_r_7688_5p,mmu_mi_r_770_3p,mmu_mi_r_7b_3p,mmu_mi_r_8092,mmu_mi_r_8096,mmu_mi_r_8097,mmu_mi_r_8100,mmu_mi_r_8101,mmu_mi_r_8102,mmu_mi_r_8104,mmu_mi_r_8106,mmu_mi_r_8107,mmu_mi_r_8108,mmu_mi_r_8110,mmu_mi_r_8119,mmu_mi_r_871_3p,mmu_mi_r_871_5p,mmu_mi_r_873a_3p,mmu_mi_r_873b,mmu_mi_r_874_5p,mmu_mi_r_879_3p,mmu_mi_r_879_5p,mmu_mi_r_881_3p,mmu_mi_r_883a_3p,mmu_mi_r_883a_5p,mmu_mi_r_92a_3p,mmu_mi_r_92b_3p,mmu_mi_r_93_3p,mmu_mi_r_93_5p,mmu_mi_r_9768_5p,mmu_mi_r_9769_3p,mmu_mi_r_98_3 p,mmu_mi_r_98_5p,mmu_pi_r_000578_gb_dq540853_mus_musculus_17_39456112_39456137_plus,mmu_pi_r_000619_gb_dq540976_mus_musculus_17_39454691_39454717_plus,mmu_pi_r_000622_gb_dq540988_mus_musculus_18_54824707_54824734_minus,mmu_pi_r_000622_gb_dq540988_mus_musculus_2_5296560_5296587_minus,mmu_pi_r_000622_gb_dq540988_mus_musculus_3_5843428_5843455_plus,mmu_pi_r_000691_gb_dq541218_mus_musculus_8_126484247_126484272_minus,mmu_pi_r_000691_gb_dq541218_mus_musculus_8_126485950_126485975_minus,mmu_pi_r_000935_gb_dq541777_mus_musculus_6_47717737_47717766_minus,mmu_pi_r_001570_gb_dq543701_mus_musculus_2_5296574_5296603_minus,mmu_pi_r_001570_gb_dq543701_mus_musculus_3_5843412_5843441_plus,mmu_pi_r_002728_gb_dq547492_mus_musculus_2_150846558_150846586_plus,mmu_pi_r_003399_gb_dq549760_mus_musculus_5_108144856_108144884_plus,mmu_pi_r_004086_gb_dq551625_mus_musculus_2_129969457_129969485_plus,mmu_pi_r_005109_gb_dq555094_mus_musculus_5_146565258_146565289_plus,mmu_pi_r_017405_gb_dq696996_mus_musculus_11_65550994_65551015_minus,mmu_pi_r_020492_gb_dq701563_mus_musculus_11_108827972_108827997_minus,mmu_pi_r_023189_gb_dq705481_mus_musculus_16_18197850_18197871_minus,mmu_pi_r_023366_gb_dq705744_mus_musculus_7_73687678_73687707_minus,mmu_pi_r_025576_gb_dq708952_mus_musculus_x_6405415_6405436_minus,mmu_pi_r_028975_gb_dq713872_mus_musculus_x_6405378_6405399_minus,mmu_pi_r_032865_gb_dq719430_mus_musculus_2_116876592_116876614_plus,mmu_pi_r_032974_gb_dq719597_mus_musculus_4_130021751_130021778_plus,mmu_pi_r_038328_gb_pi_rna_t47_mus_musculus_17_39455055_39455084_plus,mmu_pi_r_038328_gb_pi_rna_t47_mus_musculus_19_13121850_13121879_plus,mmu_pi_r_038328_gb_pi_rna_t47_mus_musculus_x_23165477_23165506_plus,mmu_pi_r_038649_gb_pi_rna_1701_mus_musculus_17_27070232_27070255_plus
265.147	x265_147_H	mmu_let_7b_5p,mmu_let_7c_5p,mmu_let_7d_3p,mmu_let_7d_5p,mmu_let_7f_2_3p,mmu_let_7g_5p,mmu_let_7k,mmu_mi_r_106b_3p,mmu_mi_r_1187,mmu_mi_r_1188_3p,mmu_mi_r_1188_5p,mmu_mi_r_1193_5p,mmu_mi_r_1198_5p,mmu_mi_r_1199_5p,mmu_mi_r_125a_5p,mmu_mi_r_125b_5p,mmu_mi_r_126b_3p,mmu_mi_r_126b_5p,mmu_mi_r_127_5p,mmu_mi_r_1291,mmu_mi_r_1298_3p,mmu_mi_r_1306_3p,mmu_mi_r_1306_5p,mmu_mi_r_130a_5p,mmu_mi_r_130b_3p,mmu_mi_r_132_3p,mmu_mi_r_133c,mmu_mi_r_135b_3p,mmu_mi_r_136_5p,mmu_mi_r_148b_5p,mmu_mi_r_151_3p,mmu_mi_r_153_5p,mmu_mi_r_181d_3p,mmu_mi_r_1839_3p,mmu_mi_r_1843b_5p,mmu_mi_r_185_3p,mmu_mi_r_186_3p,mmu_mi_r_188_5p,mmu_mi_r_1894_3p,mmu_mi_r_1894_5p,mmu_mi_r_1899,mmu_mi_r_1904,mmu_mi_r_190a_5p,mmu_mi_r_191_5p,mmu_mi_r_1932,mmu_mi_r_1933_3p,mmu_mi_r_1933_5p,mmu_mi_r_1934_3p,mmu_mi_r_1949,mmu_mi_r_1955_5p,mmu_mi_r_1956,mmu_mi_r_1957b,mmu_mi_r_1958,mmu_mi_r_195b,mmu_mi_r_1961,mmu_mi_r_1964_5p,mmu_mi_r_1966_3p,mmu_mi_r_1968_3p,mmu_mi_r_1969,mmu_mi_r_19b_2_5p,mmu_mi_r_19b_3p,mmu_mi_r_200a_5p,mmu_mi_r_204_5p,mmu_mi_r_208a_5p,mmu_mi_r_208b_5p,mmu_mi_r_20a_3p,mmu_mi_r_218_1_3p,mmu_mi_r_21a_5p,mmu_mi_r_221_3p,mmu_mi_r_224_3p,mmu_mi_r_23b_5p,mmu_mi_r_25_3p,mmu_mi_r_26a_1_3p,mmu_mi_r_27b_3p,mmu_mi_r_27b_5p,mmu_mi_r_290a_5p,mmu_mi_r_291a_5p,mmu_mi_r_293_3p,mmu_mi_r_293_5p,mmu_mi_r_294_5p,mmu_mi_r_296_5p,mmu_mi_r_298_5p,mmu_mi_r_301b_5p,mmu_mi_r_302a_3p,mmu_mi_r_302c_5p,mmu_mi_r_302d_3p,mmu_mi_r_3058_3p,mmu_mi_r_3060_5p,mmu_mi_r_3064_5p,mmu_mi_r_3072_3p,mmu_mi_r_3072_5p,mmu_mi_r_3075_5p,mmu_mi_r_3076_3p,mmu_mi_r_3079_5p,mmu_mi_r_3081_3p,mmu_mi_r_3083_3p,mmu_mi_r_3085_3p,mmu_mi_r_30a_3p,mmu_mi_r_30a_5p,mmu_mi_r_30b_5p,mmu_mi_r_30c_5p,mmu_mi_r_30d_5p,mmu_mi_r_3102_3p_2_3p,mmu_mi_r_3108_3p,mmu_mi_r_3109_3p,mmu_mi_r_320_3p,mmu_mi_r_328_3p,mmu_mi_r_328_5p,mmu_mi_r_330_3p,mmu_mi_r_338_5p,mmu_mi_r_339_3p,mmu_mi_r_339_5p,mmu_mi_r_342_5p,mmu_mi_r_344_3p,mmu_mi_r_344b_3p,mmu_mi_r_344d_2_5p,mmu_mi_r_3473b,mmu_mi_r_3473c,mmu_mi_r_350_3p,mmu_mi_r_3535,mmu_mi_r_361_3p,mmu_mi_r_361_5p,mmu_mi_r_363_3p,mmu_mi_r_363_5p,mmu_mi_r_367_3p,mmu_mi_r_370_5p,mmu_mi_r_374b_5p,mmu_mi_r_374c_3p,mmu_mi_r_376c_3p,mmu_mi_r_378a_5p,mmu_mi_r_381_3p,mmu_mi_r_3961,mmu_mi_r_3968,mmu_mi_r_421_3p,mmu_mi_r_425_3p,mmu_mi_r_433_3p,mmu_mi_r_453,mmu_mi_r_465a_5p,mmu_mi_r_466d_5p,mmu_mi_r_466j,mmu_mi_r_466l_3p,mmu_mi_r_467b_5p,mmu_mi_r_468_5p,mmu_mi_r_470_3p,mmu_mi_r_484,mmu_mi_r_486a_3p,mmu_mi_r_486a_5p,mmu_mi_r_486b_5p,mmu_mi_r_494_5p,mmu_mi_r_501_3p,mmu_mi_r_501_5p,mmu_mi_r_504_5p,mmu_mi_r_5046,mmu_mi_r_505_5p,mmu_mi_r_5098,mmu_mi_r_5110,mmu_mi_r_5121,mmu_mi_r_5126,mmu_mi_r_5127,mmu_mi_r_5134_3p,mmu_mi_r_532_5p,mmu_mi_r_542_5p,mmu_mi_r_543_5p,mmu_mi_r_5616_3p,mmu_mi_r_5617_5p,mmu_mi_r_5619_3p,mmu_mi_r_5623_5p,mmu_mi_r_5627_3p,mmu_mi_r_5709_3p,mmu_mi_r_5709_5p,mmu_mi_r_590_3p,mmu_mi_r_6238,mmu_mi_r_6354,mmu_mi_r_6385,mmu_mi_r_6387,mmu_mi_r_6395,mmu_mi_r_652_3p,mmu_mi_r_652_5p,mmu_mi_r_6537_5p,mmu_mi_r_664_5p,mmu_mi_r_669l_3p,mmu_mi_r_669m_3p,mmu_mi_r_669p_3p,mmu_mi_r_676_3p,mmu_mi_r_679_5p,mmu_mi_r_6896_3p,mmu_mi_r_6899_5p,mmu_mi_r_6904_5p,mmu_mi_r_6905_5p,mmu_mi_r_6907_3p,mmu_mi_r_6911_3p,mmu_mi_r_6914_3p,mmu_mi_r_6921_5p,mmu_mi_r_6924_5p,mmu_mi_r_6932_3p,mmu_mi_r_6943_5p,mmu_mi_r_6946_3p,mmu_mi_r_6950_3p,mmu_mi_r_6954_5p,mmu_mi_r_6961_3p,mmu_mi_r_6966_5p,mmu_mi_r_6973a_3p,mmu_mi_r_6981_3p,mmu_mi_r_6982_3p,mmu_mi_r_6982_5p,mmu_mi_r_6983_3p,mmu_mi_r_6984_3p,mmu_mi_r_6984_5p,mmu_mi_r_6987_5p,mmu_mi_r_6988_3p,mmu_mi_r_6996_3p,mmu_mi_r_7000_3p,mmu_mi_r_7008_5p,mmu_mi_r_7013_3p,mmu_mi_r_7018_5p,mmu_mi_r_7032_5p,mmu_mi_r_7034_3p,mmu_mi_r_7038_3p,mmu_mi_r_7044_5p,mmu_mi_r_7054_3p,mmu_mi_r_706,mmu_mi_r_7065_3p,mmu_mi_r_7067_5p,mmu_mi_r_7069_3p,mmu_mi_r_7071_3p,mmu_mi_r_7071_5p,mmu_mi_r_7073_3p,mmu_mi_r_708_3p,mmu_mi_r_7080_3p,mmu_mi_r_7080_5p,mmu_mi_r_7081_3p,mmu_mi_r_7088_5p,mmu_mi_r_7091_3p,mmu_mi_r_7092_3p,mmu_mi_r_7118_5p,mmu_mi_r_7119_3p,mmu_mi_r_712_5p,mmu_mi_r_717,mmu_mi_r_7229_5p,mmu_mi_r_7231_3p,mmu_mi_r_7232_5p,mmu_mi_r_7241_5p,mmu_mi_r_741_3p,mmu_mi_r_741_5p,mmu_mi_r_742_3p,mmu_mi_r_743b_3p,mmu_mi_r_743b_5p,mmu_mi_r_758_3p,mmu_mi_r_763,mmu_mi_r_7647_3p,mmu_mi_r_7650_3p,mmu_mi_r_7650_5p,mmu_mi_r_7653_3p,mmu_mi_r_7654_5p,mmu_mi_r_7660_5p,mmu_mi_r_7662_5p,mmu_mi_r_7674_3p,mmu_mi_r_7674_5p,mmu_mi_r_7677_5p,mmu_mi_r_7682_5p,mmu_mi_r_7685_3p,mmu_mi_r_7688_3p,mmu_mi_r_770_3p,mmu_mi_r_7a_5p,mmu_mi_r_802_3p,mmu_mi_r_8095,mmu_mi_r_8102,mmu_mi_r_8112,mmu_mi_r_8114,mmu_mi_r_8120,mmu_mi_r_874_3p,mmu_mi_r_875_3p,mmu_mi_r_877_3p,mmu_mi_r_883a_3p,mmu_mi_r_92a_3p,mmu_mi_r_93_3p,mmu_mi_r_93_5p,mmu_pi_r_000219_gb_dq540058_mus_musculus_17_39455665_39455691_plus,mmu_pi_r_000622_gb_dq540988_mus_musculus_2_5296560_5296587_minus,mmu_pi_r_013859_gb_dq691760_mus_musculus_9_54046065_54046096_minus,mmu_pi_r_020492_gb_dq701563_mus_musculus_11_108827972_108827997_minus,mmu_pi_r_025576_gb_dq708952_mus_musculus_x_6405415_6405436_minus,mmu_pi_r_030152_gb_dq715561_mus_musculus_18_67167521_67167549_minus,mmu_pi_r_038323_gb_pi_rna_t34_mus_musculus_13_44880547_44880577_minus,mmu_pi_r_038328_gb_pi_rna_t47_mus_musculus_6_3151091_3151120_plus,mmu_pi_r_038649_gb_pi_rna_1701_mus_musculus_17_27070232_27070255_plus
283.264	x283_264_H	mmu_let_7a_2_3p,mmu_let_7e_5p,mmu_let_7f_1_3p,mmu_let_7j,mmu_let_7k,mmu_mi_r_101a_5p,mmu_mi_r_101c,mmu_mi_r_1199_3p,mmu_mi_r_122_3p,mmu_mi_r_122_5p,mmu_mi_r_1249_3p,mmu_mi_r_125a_3p,mmu_mi_r_125a_5p,mmu_mi_r_125b_1_3p,mmu_mi_r_125b_2_3p,mmu_mi_r_125b_5p,mmu_mi_r_126a_3p,mmu_mi_r_126a_5p,mmu_mi_r_126b_3p,mmu_mi_r_126b_5p,mmu_mi_r_127_5p,mmu_mi_r_128_1_5p,mmu_mi_r_128_2_5p,mmu_mi_r_1298_3p,mmu_mi_r_129b_5p,mmu_mi_r_132_5p,mmu_mi_r_133a_3p,mmu_mi_r_133a_5p,mmu_mi_r_133b_3p,mmu_mi_r_133c,mmu_mi_r_135a_5p,mmu_mi_r_141_5p,mmu_mi_r_143_5p,mmu_mi_r_149_5p,mmu_mi_r_154_5p,mmu_mi_r_16_1_3p,mmu_mi_r_187_3p,mmu_mi_r_187_5p,mmu_mi_r_188_3p,mmu_mi_r_1898,mmu_mi_r_1901,mmu_mi_r_1902,mmu_mi_r_190a_3p,mmu_mi_r_190a_5p,mmu_mi_r_190b_3p,mmu_mi_r_192_3p,mmu_mi_r_192_5p,mmu_mi_r_1929_5p,mmu_mi_r_1930_5p,mmu_mi_r_1934_3p,mmu_mi_r_194_1_3p,mmu_mi_r_194_2_3p,mmu_mi_r_194_5p,mmu_mi_r_1941_3p,mmu_mi_r_1951,mmu_mi_r_1953,mmu_mi_r_1955_3p,mmu_mi_r_195a_3p,mmu_mi_r_199a_5p,mmu_mi_r_199b_5p,mmu_mi_r_19b_1_5p,mmu_mi_r_1a_1_5p,mmu_mi_r_1a_3p,mmu_mi_r_1b_5p,mmu_mi_r_200b_3p,mmu_mi_r_200b_5p,mmu_mi_r_203_3p,mmu_mi_r_204_5p,mmu_mi_r_208a_3p,mmu_mi_r_208b_3p,mmu_mi_r_20a_3p,mmu_mi_r_20b_3p,mmu_mi_r_211_5p,mmu_mi_r_212_3p,mmu_mi_r_215_3p,mmu_mi_r_215_5p,mmu_mi_r_216b_3p,mmu_mi_r_2183,mmu_mi_r_219c_3p,mmu_mi_r_21a_3p,mmu_mi_r_221_5p,mmu_mi_r_223_5p,mmu_mi_r_224_3p,mmu_mi_r_224_5p,mmu_mi_r_26a_1_3p,mmu_mi_r_26a_5p,mmu_mi_r_26b_3p,mmu_mi_r_295_5p,mmu_mi_r_299a_5p,mmu_mi_r_29b_1_5p,mmu_mi_r_29b_2_5p,mmu_mi_r_29c_5p,mmu_mi_r_300_3p,mmu_mi_r_302a_3p,mmu_mi_r_302a_5p,mmu_mi_r_302b_3p,mmu_mi_r_3058_5p,mmu_mi_r_3060_5p,mmu_mi_r_3070_2_3p,mmu_mi_r_3071_3p,mmu_mi_r_3071_5p,mmu_mi_r_3072_3p,mmu_mi_r_3075_3p,mmu_mi_r_3075_5p,mmu_mi_r_3084_5p,mmu_mi_r_3089_5p,mmu_mi_r_3098_5p,mmu_mi_r_3099_3p,mmu_mi_r_3099_5p,mmu_mi_r_30a_3p,mmu_mi_r_30b_5p,mmu_mi_r_30c_5p,mmu_mi_r_30d_3p,mmu_mi_r_30e_3p,mmu_mi_r_3100_5p,mmu_mi_r_3106_5p,mmu_mi_r_3109_3p,mmu_mi_r_330_3p,mmu_mi_r_339_3p,mmu_mi_r_342_5p,mmu_mi_r_344_5p,mmu_mi_r_344b_5p,mmu_mi_r_344e_3p,mmu_mi_r_344f_3p,mmu_mi_r_344g_5p,mmu_mi_r_3473e,mmu_mi_r_34c_5p,mmu_mi_r_350_3p,mmu_mi_r_3544_3p,mmu_mi_r_362_5p,mmu_mi_r_365_2_5p,mmu_mi_r_369_5p,mmu_mi_r_374c_3p,mmu_mi_r_376b_5p,mmu_mi_r_377_5p,mmu_mi_r_378a_5p,mmu_mi_r_378d,mmu_mi_r_379_3p,mmu_mi_r_381_3p,mmu_mi_r_381_5p,mmu_mi_r_382_5p,mmu_mi_r_3966,mmu_mi_r_3967,mmu_mi_r_411_5p,mmu_mi_r_431_3p,mmu_mi_r_433_5p,mmu_mi_r_449a_3p,mmu_mi_r_449a_5p,mmu_mi_r_450a_1_3p,mmu_mi_r_450b_3p,mmu_mi_r_463_5p,mmu_mi_r_467c_5p,mmu_mi_r_467d_5p,mmu_mi_r_494_3p,mmu_mi_r_497a_5p,mmu_mi_r_499_3p,mmu_mi_r_499_5p,mmu_mi_r_501_5p,mmu_mi_r_505_3p,mmu_mi_r_509_5p,mmu_mi_r_5107_5p,mmu_mi_r_511_3p,mmu_mi_r_511_5p,mmu_mi_r_5129_5p,mmu_mi_r_5133,mmu_mi_r_547_3p,mmu_mi_r_5624_3p,mmu_mi_r_5626_5p,mmu_mi_r_574_5p,mmu_mi_r_615_3p,mmu_mi_r_6344,mmu_mi_r_6367,mmu_mi_r_6374,mmu_mi_r_6378,mmu_mi_r_6389,mmu_mi_r_6392_5p,mmu_mi_r_6408,mmu_mi_r_6418_3p,mmu_mi_r_654_5p,mmu_mi_r_6546_5p,mmu_mi_r_664_5p,mmu_mi_r_669j,mmu_mi_r_669m_3p,mmu_mi_r_6715_3p,mmu_mi_r_672_3p,mmu_mi_r_672_5p,mmu_mi_r_673_3p,mmu_mi_r_6769b_5p,mmu_mi_r_687,mmu_mi_r_6903_5p,mmu_mi_r_6904_5p,mmu_mi_r_6907_3p,mmu_mi_r_6908_5p,mmu_mi_r_6918_5p,mmu_mi_r_6925_5p,mmu_mi_r_693_5p,mmu_mi_r_6930_5p,mmu_mi_r_6932_3p,mmu_mi_r_6937_3p,mmu_mi_r_6951_3p,mmu_mi_r_6953_5p,mmu_mi_r_6954_3p,mmu_mi_r_6956_3p,mmu_mi_r_696,mmu_mi_r_6962_3p,mmu_mi_r_6964_3p,mmu_mi_r_6967_5p,mmu_mi_r_6979_3p,mmu_mi_r_6980_3p,mmu_mi_r_6981_3p,mmu_mi_r_6983_3p,mmu_mi_r_6984_3p,mmu_mi_r_6988_3p,mmu_mi_r_6989_3p,mmu_mi_r_6989_5p,mmu_mi_r_6994_3p,mmu_mi_r_6999_3p,mmu_mi_r_6999_5p,mmu_mi_r_7001_3p,mmu_mi_r_7005_3p,mmu_mi_r_7005_5p,mmu_mi_r_7006_3p,mmu_mi_r_701_3p,mmu_mi_r_7010_3p,mmu_mi_r_7015_3p,mmu_mi_r_7017_3p,mmu_mi_r_7017_5p,mmu_mi_r_7019_3p,mmu_mi_r_702_3p,mmu_mi_r_7024_3p,mmu_mi_r_7025_3p,mmu_mi_r_7027_5p,mmu_mi_r_7029_3p,mmu_mi_r_7032_3p,mmu_mi_r_7040_3p,mmu_mi_r_7041_3p,mmu_mi_r_7042_3p,mmu_mi_r_7048_3p,mmu_mi_r_7051_5p,mmu_mi_r_7052_3p,mmu_mi_r_7059_3p,mmu_mi_r_7067_3p,mmu_mi_r_7070_5p,mmu_mi_r_7074_3p,mmu_mi_r_7076_3p,mmu_mi_r_7079_5p,mmu_mi_r_708_5p,mmu_mi_r_7085_3p,mmu_mi_r_7089_3p,mmu_mi_r_7092_3p,mmu_mi_r_7092_5p,mmu_mi_r_7093_3p,mmu_mi_r_7094_3p,mmu_mi_r_710,mmu_mi_r_7115_5p,mmu_mi_r_7116_5p,mmu_mi_r_7119_5p,mmu_mi_r_719,mmu_mi_r_7212_5p,mmu_mi_r_7231_3p,mmu_mi_r_7232_5p,mmu_mi_r_7242_3p,mmu_mi_r_7242_5p,mmu_mi_r_763,mmu_mi_r_7649_3p,mmu_mi_r_7662_3p,mmu_mi_r_7664_5p,mmu_mi_r_7667_3p,mmu_mi_r_7671_3p,mmu_mi_r_7686_3p,mmu_mi_r_7689_5p,mmu_mi_r_802_5p,mmu_mi_r_804,mmu_mi_r_8097,mmu_mi_r_8098,mmu_mi_r_8107,mmu_mi_r_873a_3p,mmu_mi_r_877_3p,mmu_mi_r_883b_3p,mmu_mi_r_9_3p,mmu_mi_r_9768_3p,mmu_mi_r_9768_5p,mmu_mi_r_98_3p,mmu_mi_r_99a_3p,mmu_pi_r_000616_gb_dq540965_mus_musculus_3_5843413_5843444_plus,mmu_pi_r_000691_gb_dq541218_mus_musculus_8_126428267_126428292_minus,mmu_pi_r_000691_gb_dq541218_mus_musculus_8_126455417_126455442_minus,mmu_pi_r_000691_gb_dq541218_mus_musculus_8_126457090_126457115_minus,mmu_pi_r_000691_gb_dq541218_mus_musculus_8_126462165_126462190_minus,mmu_pi_r_000691_gb_dq541218_mus_musculus_8_126492702_126492727_minus,mmu_pi_r_013377_gb_dq691037_mus_musculus_8_95079800_95079829_plus,mmu_pi_r_037443_gb_dq726144_mus_musculus_2_92364888_92364918_plus,mmu_pi_r_038323_gb_pi_rna_t34_mus_musculus_14_18071278_18071308_plus,mmu_pi_r_038328_gb_pi_rna_t47_mus_musculus_19_13121850_13121879_plus
283.264	x283_264_A	mmu_let_7e_3p,mmu_let_7g_3p,mmu_let_7j,mmu_mi_r_101b_5p,mmu_mi_r_103_1_5p,mmu_mi_r_105,mmu_mi_r_10a_3p,mmu_mi_r_1224_3p,mmu_mi_r_1251_5p,mmu_mi_r_129_1_3p,mmu_mi_r_129_5p,mmu_mi_r_129b_5p,mmu_mi_r_130a_5p,mmu_mi_r_133a_5p,mmu_mi_r_135a_5p,mmu_mi_r_138_2_3p,mmu_mi_r_141_5p,mmu_mi_r_146a_5p,mmu_mi_r_146b_3p,mmu_mi_r_146b_5p,mmu_mi_r_147_3p,mmu_mi_r_147_5p,mmu_mi_r_148a_5p,mmu_mi_r_16_2_3p,mmu_mi_r_188_5p,mmu_mi_r_1895,mmu_mi_r_1898,mmu_mi_r_1906,mmu_mi_r_190a_3p,mmu_mi_r_191_3p,mmu_mi_r_1912_5p,mmu_mi_r_1929_5p,mmu_mi_r_1932,mmu_mi_r_1934_3p,mmu_mi_r_1934_5p,mmu_mi_r_1938,mmu_mi_r_193b_3p,mmu_mi_r_1940,mmu_mi_r_1943_3p,mmu_mi_r_1945,mmu_mi_r_1946a,mmu_mi_r_1946b,mmu_mi_r_1947_3p,mmu_mi_r_1949,mmu_mi_r_1950,mmu_mi_r_195a_3p,mmu_mi_r_1961,mmu_mi_r_1963,mmu_mi_r_1964_3p,mmu_mi_r_196a_5p,mmu_mi_r_1970,mmu_mi_r_1971,mmu_mi_r_19a_5p,mmu_mi_r_19b_1_5p,mmu_mi_r_1b_3p,mmu_mi_r_205_3p,mmu_mi_r_208a_5p,mmu_mi_r_20a_3p,mmu_mi_r_20b_3p,mmu_mi_r_212_3p,mmu_mi_r_218_5p,mmu_mi_r_219a_1_3p,mmu_mi_r_21a_3p,mmu_mi_r_223_5p,mmu_mi_r_24_2_5p,mmu_mi_r_28a_5p,mmu_mi_r_302c_5p,mmu_mi_r_3057_5p,mmu_mi_r_3058_3p,mmu_mi_r_3058_5p,mmu_mi_r_3060_3p,mmu_mi_r_3062_3p,mmu_mi_r_3064_5p,mmu_mi_r_3066_5p,mmu_mi_r_3067_5p,mmu_mi_r_3069_3p,mmu_mi_r_3074_1_3p,mmu_mi_r_3079_5p,mmu_mi_r_3085_3p,mmu_mi_r_3088_3p,mmu_mi_r_3094_3p,mmu_mi_r_3097_3p,mmu_mi_r_3098_5p,mmu_mi_r_30c_2_3p,mmu_mi_r_3102_5p_2_5p,mmu_mi_r_3108_5p,mmu_mi_r_3112_3p,mmu_mi_r_327,mmu_mi_r_33_3p,mmu_mi_r_33_5p,mmu_mi_r_331_5p,mmu_mi_r_335_3p,mmu_mi_r_338_5p,mmu_mi_r_344b_3p,mmu_mi_r_344c_3p,mmu_mi_r_3470b,mmu_mi_r_3473f.,mmu_mi_r_3473g,mmu_mi_r_34b_3p,mmu_mi_r_34c_5p,mmu_mi_r_350_5p,mmu_mi_r_3535,mmu_mi_r_3544_3p,mmu_mi_r_3569_3p,mmu_mi_r_3572_5p,mmu_mi_r_3620_5p,mmu_mi_r_374c_5p,mmu_mi_r_383_3p,mmu_mi_r_384_3p,mmu_mi_r_3966,mmu_mi_r_411_3p,mmu_mi_r_429_5p,mmu_mi_r_451b,mmu_mi_r_455_3p,mmu_mi_r_466a_3p_mmu_mi_r_466e_3p,mmu_mi_r_467g,mmu_mi_r_487b_3p,mmu_mi_r_490_3p,mmu_mi_r_490_5p,mmu_mi_r_494_5p,mmu_mi_r_495_3p,mmu_mi_r_496a_5p,mmu_mi_r_497a_3p,mmu_mi_r_497b,mmu_mi_r_504_5p,mmu_mi_r_505_3p,mmu_mi_r_5103,mmu_mi_r_5107_3p,mmu_mi_r_511_5p,mmu_mi_r_5114,mmu_mi_r_5119,mmu_mi_r_5120,mmu_mi_r_5124b,mmu_mi_r_5125,mmu_mi_r_5129_3p,mmu_mi_r_5129_5p,mmu_mi_r_5134_5p,mmu_mi_r_532_3p,mmu_mi_r_543_3p,mmu_mi_r_544_5p,mmu_mi_r_547_3p,mmu_mi_r_551b_5p,mmu_mi_r_5709_5p,mmu_mi_r_5710,mmu_mi_r_582_5p,mmu_mi_r_590_5p,mmu_mi_r_592_5p,mmu_mi_r_598_5p,mmu_mi_r_615_5p,mmu_mi_r_6237,mmu_mi_r_6355,mmu_mi_r_6357,mmu_mi_r_6378,mmu_mi_r_6389,mmu_mi_r_6390,mmu_mi_r_6395,mmu_mi_r_6419,mmu_mi_r_653_5p,mmu_mi_r_6546_3p,mmu_mi_r_664_5p,mmu_mi_r_669i,mmu_mi_r_669p_3p,mmu_mi_r_670_5p,mmu_mi_r_673_3p,mmu_mi_r_675_3p,mmu_mi_r_675_5p,mmu_mi_r_681,mmu_mi_r_6900_3p,mmu_mi_r_6908_3p,mmu_mi_r_691,mmu_mi_r_6912_5p,mmu_mi_r_6914_3p,mmu_mi_r_6914_5p,mmu_mi_r_6915_3p,mmu_mi_r_6917_5p,mmu_mi_r_6919_3p,mmu_mi_r_6921_3p,mmu_mi_r_693_3p,mmu_mi_r_6930_5p,mmu_mi_r_6932_5p,mmu_mi_r_6933_3p,mmu_mi_r_6937_5p,mmu_mi_r_6945_5p,mmu_mi_r_6947_5p,mmu_mi_r_6949_3p,mmu_mi_r_6949_5p,mmu_mi_r_6951_5p,mmu_mi_r_6952_3p,mmu_mi_r_6953_3p,mmu_mi_r_6953_5p,mmu_mi_r_6956_3p,mmu_mi_r_6958_5p,mmu_mi_r_6960_5p,mmu_mi_r_6973b_3p,mmu_mi_r_6974_5p,mmu_mi_r_698_5p,mmu_mi_r_6983_5p,mmu_mi_r_6986_5p,mmu_mi_r_6991_3p,mmu_mi_r_6994_3p,mmu_mi_r_701_3p,mmu_mi_r_7012_5p,mmu_mi_r_7015_3p,mmu_mi_r_7015_5p,mmu_mi_r_7024_5p,mmu_mi_r_7027_3p,mmu_mi_r_7027_5p,mmu_mi_r_7029_3p,mmu_mi_r_7029_5p,mmu_mi_r_7034_5p,mmu_mi_r_7052_5p,mmu_mi_r_7058_3p,mmu_mi_r_7061_5p,mmu_mi_r_7062_5p,mmu_mi_r_7063_5p,mmu_mi_r_7065_3p,mmu_mi_r_7070_5p,mmu_mi_r_7081_3p,mmu_mi_r_7083_3p,mmu_mi_r_7088_3p,mmu_mi_r_709,mmu_mi_r_7091_3p,mmu_mi_r_7091_5p,mmu_mi_r_7093_3p,mmu_mi_r_7093_5p,mmu_mi_r_7115_5p,mmu_mi_r_7116_5p,mmu_mi_r_7117_5p,mmu_mi_r_719,mmu_mi_r_7212_5p,mmu_mi_r_7219_5p,mmu_mi_r_7223_5p,mmu_mi_r_7228_3p,mmu_mi_r_7235_5p,mmu_mi_r_7239_3p,mmu_mi_r_742_3p,mmu_mi_r_7646_5p,mmu_mi_r_7648_5p,mmu_mi_r_7651_3p,mmu_mi_r_7653_5p,mmu_mi_r_7654_5p,mmu_mi_r_7655_5p,mmu_mi_r_7656_3p,mmu_mi_r_7658_5p,mmu_mi_r_7665_5p,mmu_mi_r_7667_3p,mmu_mi_r_7667_5p,mmu_mi_r_7671_5p,mmu_mi_r_7675_3p,mmu_mi_r_7675_5p,mmu_mi_r_7687_5p,mmu_mi_r_7a_2_3p,mmu_mi_r_8091,mmu_mi_r_8092,mmu_mi_r_8097,mmu_mi_r_8105,mmu_mi_r_8109,mmu_mi_r_8116,mmu_mi_r_873a_5p,mmu_mi_r_877_3p,mmu_mi_r_877_5p,mmu_mi_r_92a_1_5p,mmu_pi_r_000159_gb_dq539904_mus_musculus_9_118523651_118523678_minus,mmu_pi_r_000219_gb_dq540058_mus_musculus_17_39455665_39455691_plus,mmu_pi_r_004086_gb_dq551625_mus_musculus_2_129969457_129969485_plus,mmu_pi_r_020692_gb_dq701869_mus_musculus_5_115840031_115840052_plus,mmu_pi_r_022545_gb_dq704578_mus_musculus_9_67495705_67495734_minus,mmu_pi_r_023189_gb_dq705481_mus_musculus_16_18197850_18197871_minus,mmu_pi_r_025576_gb_dq708952_mus_musculus_x_6405415_6405436_minus,mmu_pi_r_028241_gb_dq712821_mus_musculus_x_56741084_56741107_minus,mmu_pi_r_028252_gb_dq712837_mus_musculus_7_81403433_81403455_plus,mmu_pi_r_028975_gb_dq713872_mus_musculus_x_6405378_6405399_minus,mmu_pi_r_032015_gb_dq718174_mus_musculus_2_10427172_10427193_plus,mmu_pi_r_032974_gb_dq719597_mus_musculus_4_130021751_130021778_plus,mmu_pi_r_038323_gb_pi_rna_t34_mus_musculus_14_18071278_18071308_plus
290.086	x290_086_A	mmu_let_7a_2_3p,mmu_let_7e_3p,mmu_let_7e_5p,mmu_let_7f_2_3p,mmu_let_7g_3p,mmu_mi_r_100_5p,mmu_mi_r_103_1_5p,mmu_mi_r_103_2_5p,mmu_mi_r_105,mmu_mi_r_10b_3p,mmu_mi_r_1188_5p,mmu_mi_r_1199_3p,mmu_mi_r_1231_3p,mmu_mi_r_124_3p,mmu_mi_r_1249_3p,mmu_mi_r_1249_5p,mmu_mi_r_125a_3p,mmu_mi_r_125a_5p,mmu_mi_r_125b_1_3p,mmu_mi_r_125b_2_3p,mmu_mi_r_125b_5p,mmu_mi_r_1264_3p,mmu_mi_r_126a_3p,mmu_mi_r_126a_5p,mmu_mi_r_126b_3p,mmu_mi_r_1298_3p,mmu_mi_r_130a_3p,mmu_mi_r_133a_3p,mmu_mi_r_133a_5p,mmu_mi_r_133b_3p,mmu_mi_r_135a_5p,mmu_mi_r_135b_3p,mmu_mi_r_136_3p,mmu_mi_r_138_2_3p,mmu_mi_r_141_5p,mmu_mi_r_143_3p,mmu_mi_r_145a_3p,mmu_mi_r_145a_5p,mmu_mi_r_149_3p,mmu_mi_r_149_5p,mmu_mi_r_152_3p,mmu_mi_r_154_5p,mmu_mi_r_181a_1_3p,mmu_mi_r_181a_5p,mmu_mi_r_181b_2_3p,mmu_mi_r_181b_5p,mmu_mi_r_181d_3p,mmu_mi_r_183_3p,mmu_mi_r_184_3p,mmu_mi_r_1843b_5p,mmu_mi_r_187_5p,mmu_mi_r_1895,mmu_mi_r_1897_5p,mmu_mi_r_1907,mmu_mi_r_190b_3p,mmu_mi_r_1933_5p,mmu_mi_r_193b_3p,mmu_mi_r_1941_5p,mmu_mi_r_1946a,mmu_mi_r_1947_3p,mmu_mi_r_1949,mmu_mi_r_1950,mmu_mi_r_1951,mmu_mi_r_195a_3p,mmu_mi_r_195a_5p,mmu_mi_r_1962,mmu_mi_r_1963,mmu_mi_r_1966_3p,mmu_mi_r_196a_1_3p,mmu_mi_r_196a_2_3p,mmu_mi_r_199a_3p_mmu_mi_r_199b_3p,mmu_mi_r_199a_5p,mmu_mi_r_199b_5p,mmu_mi_r_1a_1_5p,mmu_mi_r_1a_2_5p,mmu_mi_r_1a_3p,mmu_mi_r_1b_5p,mmu_mi_r_200b_3p,mmu_mi_r_203_5p,mmu_mi_r_204_5p,mmu_mi_r_208a_3p,mmu_mi_r_208a_5p,mmu_mi_r_208b_3p,mmu_mi_r_208b_5p,mmu_mi_r_20a_3p,mmu_mi_r_211_3p,mmu_mi_r_2136,mmu_mi_r_214_3p,mmu_mi_r_214_5p,mmu_mi_r_216b_3p,mmu_mi_r_216c_3p,mmu_mi_r_217_3p,mmu_mi_r_218_5p,mmu_mi_r_21a_3p,mmu_mi_r_224_3p,mmu_mi_r_224_5p,mmu_mi_r_23a_3p,mmu_mi_r_23a_5p,mmu_mi_r_23b_3p,mmu_mi_r_23b_5p,mmu_mi_r_24_3p,mmu_mi_r_26a_1_3p,mmu_mi_r_26a_5p,mmu_mi_r_27a_5p,mmu_mi_r_27b_3p,mmu_mi_r_28a_3p,mmu_mi_r_291a_5p,mmu_mi_r_292a_5p,mmu_mi_r_293_5p,mmu_mi_r_294_3p,mmu_mi_r_295_5p,mmu_mi_r_297a_3p_mmu_mi_r_297b_3p_mmu_mi_r_297c_3p,mmu_mi_r_299a_3p,mmu_mi_r_299a_5p,mmu_mi_r_299b_3p,mmu_mi_r_29b_2_5p,mmu_mi_r_29c_5p,mmu_mi_r_302a_3p,mmu_mi_r_302b_3p,mmu_mi_r_3057_3p,mmu_mi_r_3057_5p,mmu_mi_r_3060_5p,mmu_mi_r_3063_3p,mmu_mi_r_3066_3p,mmu_mi_r_3067_3p,mmu_mi_r_3067_5p,mmu_mi_r_3072_5p,mmu_mi_r_3078_3p,mmu_mi_r_3078_5p,mmu_mi_r_3081_3p,mmu_mi_r_3084_5p,mmu_mi_r_3085_3p,mmu_mi_r_3087_5p,mmu_mi_r_3091_5p,mmu_mi_r_3092_5p,mmu_mi_r_3094_5p,mmu_mi_r_30c_2_3p,mmu_mi_r_3100_3p,mmu_mi_r_3101_3p,mmu_mi_r_3102_5p,mmu_mi_r_3102_5p_2_5p,mmu_mi_r_3105_3p,mmu_mi_r_3109_3p,mmu_mi_r_3110_5p,mmu_mi_r_320_5p,mmu_mi_r_322_3p,mmu_mi_r_327,mmu_mi_r_328_5p,mmu_mi_r_329_5p,mmu_mi_r_33_3p,mmu_mi_r_335_3p,mmu_mi_r_335_5p,mmu_mi_r_343,mmu_mi_r_344b_5p,mmu_mi_r_344f_3p,mmu_mi_r_344g_3p,mmu_mi_r_344h_3p,mmu_mi_r_3473a,mmu_mi_r_3473e,mmu_mi_r_3473f.,mmu_mi_r_3475_5p,mmu_mi_r_34b_3p,mmu_mi_r_34c_3p,mmu_mi_r_34c_5p,mmu_mi_r_351_5p,mmu_mi_r_3535,mmu_mi_r_3544_3p,mmu_mi_r_3544_5p,mmu_mi_r_3547_5p,mmu_mi_r_3618_5p,mmu_mi_r_3620_5p,mmu_mi_r_365_2_5p,mmu_mi_r_365_3p,mmu_mi_r_369_3p,mmu_mi_r_376b_3p,mmu_mi_r_376b_5p,mmu_mi_r_376c_3p,mmu_mi_r_376c_5p,mmu_mi_r_377_3p,mmu_mi_r_378a_5p,mmu_mi_r_378c,mmu_mi_r_379_3p,mmu_mi_r_379_5p,mmu_mi_r_381_5p,mmu_mi_r_382_3p,mmu_mi_r_3964,mmu_mi_r_410_3p,mmu_mi_r_411_5p,mmu_mi_r_412_5p,mmu_mi_r_429_3p,mmu_mi_r_429_5p,mmu_mi_r_431_3p,mmu_mi_r_432,mmu_mi_r_434_3p,mmu_mi_r_449a_3p,mmu_mi_r_450a_5p,mmu_mi_r_451b,mmu_mi_r_452_3p,mmu_mi_r_452_5p,mmu_mi_r_455_3p,mmu_mi_r_455_5p,mmu_mi_r_465d_5p,mmu_mi_r_466h_3p,mmu_mi_r_466l_3p,mmu_mi_r_466q,mmu_mi_r_467c_3p,mmu_mi_r_467c_5p,mmu_mi_r_467f.,mmu_mi_r_483_5p,mmu_mi_r_485_3p,mmu_mi_r_487b_3p,mmu_mi_r_489_3p,mmu_mi_r_490_3p,mmu_mi_r_490_5p,mmu_mi_r_493_3p,mmu_mi_r_494_5p,mmu_mi_r_497a_3p,mmu_mi_r_497a_5p,mmu_mi_r_499_3p,mmu_mi_r_499_5p,mmu_mi_r_501_5p,mmu_mi_r_504_3p,mmu_mi_r_5101,mmu_mi_r_5103,mmu_mi_r_5106,mmu_mi_r_5114,mmu_mi_r_5118,mmu_mi_r_5122,mmu_mi_r_5130,mmu_mi_r_5132_3p,mmu_mi_r_5135,mmu_mi_r_540_5p,mmu_mi_r_541_3p,mmu_mi_r_541_5p,mmu_mi_r_543_3p,mmu_mi_r_547_3p,mmu_mi_r_5616_3p,mmu_mi_r_5621_5p,mmu_mi_r_5623_5p,mmu_mi_r_582_3p,mmu_mi_r_592_3p,mmu_mi_r_592_5p,mmu_mi_r_615_3p,mmu_mi_r_6339,mmu_mi_r_6340,mmu_mi_r_6342,mmu_mi_r_6343,mmu_mi_r_6351,mmu_mi_r_6353,mmu_mi_r_6356,mmu_mi_r_6362,mmu_mi_r_6364,mmu_mi_r_6367,mmu_mi_r_6370,mmu_mi_r_6372,mmu_mi_r_6382,mmu_mi_r_6384,mmu_mi_r_6390,mmu_mi_r_6394,mmu_mi_r_6400,mmu_mi_r_6401,mmu_mi_r_6411,mmu_mi_r_6420,mmu_mi_r_6516_5p,mmu_mi_r_653_5p,mmu_mi_r_6538,mmu_mi_r_6546_5p,mmu_mi_r_665_3p,mmu_mi_r_665_5p,mmu_mi_r_666_3p,mmu_mi_r_666_5p,mmu_mi_r_669a_3_3p,mmu_mi_r_669a_3p_mmu_mi_r_669o_3p,mmu_mi_r_669b_5p,mmu_mi_r_669c_5p,mmu_mi_r_669h_5p,mmu_mi_r_669j,mmu_mi_r_673_5p,mmu_mi_r_675_3p,mmu_mi_r_6769b_3p,mmu_mi_r_681,mmu_mi_r_682,mmu_mi_r_684,mmu_mi_r_687,mmu_mi_r_6896_3p,mmu_mi_r_6896_5p,mmu_mi_r_6903_5p,mmu_mi_r_6908_5p,mmu_mi_r_691,mmu_mi_r_6910_5p,mmu_mi_r_6912_3p,mmu_mi_r_6912_5p,mmu_mi_r_6913_5p,mmu_mi_r_6915_3p,mmu_mi_r_6916_3p,mmu_mi_r_6919_3p,mmu_mi_r_6920_3p,mmu_mi_r_6921_5p,mmu_mi_r_6922_3p,mmu_mi_r_6923_5p,mmu_mi_r_6926_5p,mmu_mi_r_6928_5p,mmu_mi_r_6929_3p,mmu_mi_r_6930_5p,mmu_mi_r_6932_3p,mmu_mi_r_6932_5p,mmu_mi_r_6933_3p,mmu_mi_r_6935_3p,mmu_mi_r_6937_5p,mmu_mi_r_6938_3p,mmu_mi_r_6942_5p,mmu_mi_r_6944_5p,mmu_mi_r_6948_5p,mmu_mi_r_6949_5p,mmu_mi_r_695,mmu_mi_r_6953_3p,mmu_mi_r_696,mmu_mi_r_6960_5p,mmu_mi_r_6963_5p,mmu_mi_r_6964_3p,mmu_mi_r_6964_5p,mmu_mi_r_6973b_3p,mmu_mi_r_6973b_5p,mmu_mi_r_6975_3p,mmu_mi_r_6975_5p,mmu_mi_r_6977_3p,mmu_mi_r_6979_5p,mmu_mi_r_698_3p,mmu_mi_r_698_5p,mmu_mi_r_6983_5p,mmu_mi_r_6986_3p,mmu_mi_r_6987_5p,mmu_mi_r_6989_3p,mmu_mi_r_6990_5p,mmu_mi_r_6993_5p,mmu_mi_r_6994_3p,mmu_mi_r_6994_5p,mmu_mi_r_6995_3p,mmu_mi_r_6996_5p,mmu_mi_r_7000_5p,mmu_mi_r_7002_3p,mmu_mi_r_7008_3p,mmu_mi_r_7008_5p,mmu_mi_r_7009_5p,mmu_mi_r_701_3p,mmu_mi_r_7010_3p,mmu_mi_r_7010_5p,mmu_mi_r_7012_3p,mmu_mi_r_7012_5p,mmu_mi_r_7013_3p,mmu_mi_r_7015_3p,mmu_mi_r_7016_5p,mmu_mi_r_7018_5p,mmu_mi_r_7019_5p,mmu_mi_r_702_3p,mmu_mi_r_7023_3p,mmu_mi_r_7024_5p,mmu_mi_r_7027_3p,mmu_mi_r_7028_3p,mmu_mi_r_703,mmu_mi_r_7036a_3p,mmu_mi_r_7037_3p,mmu_mi_r_7037_5p,mmu_mi_r_7038_3p,mmu_mi_r_7038_5p,mmu_mi_r_7040_5p,mmu_mi_r_7042_3p,mmu_mi_r_7044_5p,mmu_mi_r_7045_5p,mmu_mi_r_7048_3p,mmu_mi_r_7048_5p,mmu_mi_r_7054_3p,mmu_mi_r_7055_5p,mmu_mi_r_706,mmu_mi_r_7061_3p,mmu_mi_r_7062_5p,mmu_mi_r_7064_5p,mmu_mi_r_7068_3p,mmu_mi_r_7068_5p,mmu_mi_r_7069_3p,mmu_mi_r_707,mmu_mi_r_7070_5p,mmu_mi_r_7074_5p,mmu_mi_r_7076_3p,mmu_mi_r_7076_5p,mmu_mi_r_7079_3p,mmu_mi_r_708_5p,mmu_mi_r_7080_5p,mmu_mi_r_7082_5p,mmu_mi_r_7085_3p,mmu_mi_r_7088_3p,mmu_mi_r_7088_5p,mmu_mi_r_709,mmu_mi_r_7092_3p,mmu_mi_r_7094_3p,mmu_mi_r_7119_5p,mmu_mi_r_714,mmu_mi_r_7211_3p,mmu_mi_r_7212_3p,mmu_mi_r_7212_5p,mmu_mi_r_7214_5p,mmu_mi_r_7215_3p,mmu_mi_r_7216_5p,mmu_mi_r_7220_5p,mmu_mi_r_7223_5p,mmu_mi_r_7224_3p,mmu_mi_r_7225_5p,mmu_mi_r_7226_3p,mmu_mi_r_7227_3p,mmu_mi_r_7228_3p,mmu_mi_r_7233_3p,mmu_mi_r_7234_3p,mmu_mi_r_7237_5p,mmu_mi_r_7239_5p,mmu_mi_r_7240_3p,mmu_mi_r_7240_5p,mmu_mi_r_741_3p,mmu_mi_r_742_3p,mmu_mi_r_744_3p,mmu_mi_r_758_5p,mmu_mi_r_759,mmu_mi_r_762,mmu_mi_r_7647_5p,mmu_mi_r_7648_5p,mmu_mi_r_7649_3p,mmu_mi_r_7649_5p,mmu_mi_r_7651_3p,mmu_mi_r_7652_5p,mmu_mi_r_7653_3p,mmu_mi_r_7654_5p,mmu_mi_r_7655_3p,mmu_mi_r_7656_5p,mmu_mi_r_7661_3p,mmu_mi_r_7661_5p,mmu_mi_r_7662_5p,mmu_mi_r_7664_3p,mmu_mi_r_7664_5p,mmu_mi_r_7665_5p,mmu_mi_r_7666_5p,mmu_mi_r_7667_3p,mmu_mi_r_7671_5p,mmu_mi_r_7672_5p,mmu_mi_r_7673_5p,mmu_mi_r_7674_3p,mmu_mi_r_7675_3p,mmu_mi_r_7675_5p,mmu_mi_r_7677_3p,mmu_mi_r_7678_5p,mmu_mi_r_7679_3p,mmu_mi_r_7679_5p,mmu_mi_r_7681_5p,mmu_mi_r_7686_5p,mmu_mi_r_7687_3p,mmu_mi_r_7b_5p,mmu_mi_r_8093,mmu_mi_r_8094,mmu_mi_r_8099,mmu_mi_r_8100,mmu_mi_r_8105,mmu_mi_r_8107,mmu_mi_r_8111,mmu_mi_r_8113,mmu_mi_r_8117,mmu_mi_r_8119,mmu_mi_r_873b,mmu_mi_r_876_5p,mmu_mi_r_879_5p,mmu_mi_r_881_5p,mmu_mi_r_883a_3p,mmu_mi_r_92a_2_5p,mmu_mi_r_96_3p,mmu_mi_r_9769_5p,mmu_mi_r_99a_3p,mmu_mi_r_99a_5p,mmu_mi_r_99b_3p,mmu_mi_r_99b_5p,mmu_pi_r_000219_gb_dq540058_mus_musculus_17_39455665_39455691_plus,mmu_pi_r_000622_gb_dq540988_mus_musculus_18_54824707_54824734_minus,mmu_pi_r_000622_gb_dq540988_mus_musculus_3_5843428_5843455_plus,mmu_pi_r_001662_gb_dq544105_mus_musculus_2_151104333_151104361_minus,mmu_pi_r_003848_gb_dq551015_mus_musculus_10_18489292_18489321_minus,mmu_pi_r_007729_gb_dq564913_mus_musculus_9_123310826_123310855_plus,mmu_pi_r_022956_gb_dq705141_mus_musculus_5_115112809_115112837_minus,mmu_pi_r_024744_gb_dq707762_mus_musculus_17_66099819_66099850_minus,mmu_pi_r_035342_gb_dq723105_mus_musculus_15_59084194_59084224_minus,mmu_pi_r_037443_gb_dq726144_mus_musculus_2_92364888_92364918_plus,mmu_pi_r_038323_gb_pi_rna_t34_mus_musculus_9_3258895_3258925_minus,mmu_pi_r_038328_gb_pi_rna_t47_mus_musculus_17_39455055_39455084_plus
290.087	x290_087_H	mmu_let_7a_1_3p_mmu_let_7c_2_3p,mmu_let_7a_5p,mmu_let_7b_3p,mmu_let_7b_5p,mmu_let_7c_1_3p,mmu_let_7c_5p,mmu_let_7d_3p,mmu_let_7d_5p,mmu_let_7e_5p,mmu_let_7f_1_3p,mmu_let_7f_2_3p,mmu_let_7f_5p,mmu_let_7g_3p,mmu_let_7g_5p,mmu_let_7i_3p,mmu_let_7i_5p,mmu_let_7j,mmu_let_7k,mmu_mi_r_100_3p,mmu_mi_r_101a_3p,mmu_mi_r_101a_5p,mmu_mi_r_101b_3p,mmu_mi_r_101c,mmu_mi_r_103_1_5p,mmu_mi_r_103_2_5p,mmu_mi_r_103_3p,mmu_mi_r_105,mmu_mi_r_106a_3p,mmu_mi_r_106b_3p,mmu_mi_r_106b_5p,mmu_mi_r_107_3p,mmu_mi_r_10a_3p,mmu_mi_r_10a_5p,mmu_mi_r_10b_3p,mmu_mi_r_10b_5p,mmu_mi_r_1190,mmu_mi_r_1191a,mmu_mi_r_1191b_3p,mmu_mi_r_1192,mmu_mi_r_1193_3p,mmu_mi_r_1193_5p,mmu_mi_r_1194,mmu_mi_r_1197_5p,mmu_mi_r_1198_5p,mmu_mi_r_1199_5p,mmu_mi_r_122_3p,mmu_mi_r_122_5p,mmu_mi_r_1224_5p,mmu_mi_r_124_3p,mmu_mi_r_1247_5p,mmu_mi_r_1249_3p,mmu_mi_r_1258_5p,mmu_mi_r_125a_3p,mmu_mi_r_125b_2_3p,mmu_mi_r_125b_5p,mmu_mi_r_126a_3p,mmu_mi_r_126a_5p,mmu_mi_r_126b_5p,mmu_mi_r_127_3p,mmu_mi_r_128_1_5p,mmu_mi_r_128_3p,mmu_mi_r_129_1_3p,mmu_mi_r_129_5p,mmu_mi_r_1298_5p,mmu_mi_r_129b_5p,mmu_mi_r_130a_5p,mmu_mi_r_130c,mmu_mi_r_132_5p,mmu_mi_r_133b_5p,mmu_mi_r_133c,mmu_mi_r_134_3p,mmu_mi_r_134_5p,mmu_mi_r_136_3p,mmu_mi_r_137_3p,mmu_mi_r_137_5p,mmu_mi_r_138_1_3p,mmu_mi_r_138_2_3p,mmu_mi_r_139_3p,mmu_mi_r_140_3p,mmu_mi_r_140_5p,mmu_mi_r_141_3p,mmu_mi_r_142a_3p,mmu_mi_r_142a_5p,mmu_mi_r_142b,mmu_mi_r_144_3p,mmu_mi_r_144_5p,mmu_mi_r_145b,mmu_mi_r_146a_3p,mmu_mi_r_146a_5p,mmu_mi_r_146b_3p,mmu_mi_r_146b_5p,mmu_mi_r_147_3p,mmu_mi_r_148b_3p,mmu_mi_r_149_3p,mmu_mi_r_150_3p,mmu_mi_r_150_5p,mmu_mi_r_151_5p,mmu_mi_r_152_5p,mmu_mi_r_153_3p,mmu_mi_r_153_5p,mmu_mi_r_154_5p,mmu_mi_r_155_3p,mmu_mi_r_155_5p,mmu_mi_r_15a_3p,mmu_mi_r_15a_5p,mmu_mi_r_15b_3p,mmu_mi_r_15b_5p,mmu_mi_r_16_1_3p,mmu_mi_r_16_2_3p,mmu_mi_r_16_5p,mmu_mi_r_17_3p,mmu_mi_r_17_5p,mmu_mi_r_181a_2_3p,mmu_mi_r_181a_5p,mmu_mi_r_181b_1_3p,mmu_mi_r_182_5p,mmu_mi_r_183_5p,mmu_mi_r_1839_3p,mmu_mi_r_1839_5p,mmu_mi_r_184_3p,mmu_mi_r_1843a_3p,mmu_mi_r_185_3p,mmu_mi_r_185_5p,mmu_mi_r_186_3p,mmu_mi_r_186_5p,mmu_mi_r_187_3p,mmu_mi_r_187_5p,mmu_mi_r_1894_3p,mmu_mi_r_1894_5p,mmu_mi_r_1895,mmu_mi_r_1896,mmu_mi_r_1897_5p,mmu_mi_r_1898,mmu_mi_r_18a_5p,mmu_mi_r_18b_5p,mmu_mi_r_1900,mmu_mi_r_1901,mmu_mi_r_1903,mmu_mi_r_1905,mmu_mi_r_1906,mmu_mi_r_1907,mmu_mi_r_190a_3p,mmu_mi_r_190a_5p,mmu_mi_r_190b_5p,mmu_mi_r_191_3p,mmu_mi_r_191_5p,mmu_mi_r_1912_5p,mmu_mi_r_192_3p,mmu_mi_r_192_5p,mmu_mi_r_1928,mmu_mi_r_1930_3p,mmu_mi_r_1933_3p,mmu_mi_r_1933_5p,mmu_mi_r_1936,mmu_mi_r_193a_3p,mmu_mi_r_193a_5p,mmu_mi_r_194_1_3p,mmu_mi_r_194_5p,mmu_mi_r_1941_3p,mmu_mi_r_1941_5p,mmu_mi_r_1942,mmu_mi_r_1943_5p,mmu_mi_r_1948_5p,mmu_mi_r_1950,mmu_mi_r_1951,mmu_mi_r_1952,mmu_mi_r_1953,mmu_mi_r_1954,mmu_mi_r_195a_5p,mmu_mi_r_1964_3p,mmu_mi_r_1966_5p,mmu_mi_r_1968_5p,mmu_mi_r_196b_5p,mmu_mi_r_1982_3p,mmu_mi_r_19a_3p,mmu_mi_r_19a_5p,mmu_mi_r_19b_3p,mmu_mi_r_200a_3p,mmu_mi_r_200b_3p,mmu_mi_r_200b_5p,mmu_mi_r_200c_3p,mmu_mi_r_202_3p,mmu_mi_r_202_5p,mmu_mi_r_203_3p,mmu_mi_r_205_3p,mmu_mi_r_205_5p,mmu_mi_r_206_5p,mmu_mi_r_208a_5p,mmu_mi_r_20a_3p,mmu_mi_r_20a_5p,mmu_mi_r_20b_5p,mmu_mi_r_210_3p,mmu_mi_r_210_5p,mmu_mi_r_211_3p,mmu_mi_r_211_5p,mmu_mi_r_212_3p,mmu_mi_r_212_5p,mmu_mi_r_2136,mmu_mi_r_2139,mmu_mi_r_214_3p,mmu_mi_r_215_3p,mmu_mi_r_215_5p,mmu_mi_r_217_3p,mmu_mi_r_217_5p,mmu_mi_r_218_1_3p,mmu_mi_r_218_5p,mmu_mi_r_2183,mmu_mi_r_219a_1_3p,mmu_mi_r_219c_3p,mmu_mi_r_219c_5p,mmu_mi_r_21a_3p,mmu_mi_r_21a_5p,mmu_mi_r_21b,mmu_mi_r_21c,mmu_mi_r_22_5p,mmu_mi_r_221_5p,mmu_mi_r_223_5p,mmu_mi_r_224_3p,mmu_mi_r_23a_3p,mmu_mi_r_23b_3p,mmu_mi_r_24_1_5p,mmu_mi_r_24_3p,mmu_mi_r_25_3p,mmu_mi_r_25_5p,mmu_mi_r_26a_1_3p,mmu_mi_r_26a_2_3p,mmu_mi_r_26a_5p,mmu_mi_r_26b_5p,mmu_mi_r_27a_3p,mmu_mi_r_27b_3p,mmu_mi_r_28a_3p,mmu_mi_r_28c,mmu_mi_r_290a_3p,mmu_mi_r_290b_3p,mmu_mi_r_291b_3p,mmu_mi_r_291b_5p,mmu_mi_r_292a_3p,mmu_mi_r_292a_5p,mmu_mi_r_295_3p,mmu_mi_r_296_3p,mmu_mi_r_299b_3p,mmu_mi_r_299b_5p,mmu_mi_r_29a_3p,mmu_mi_r_29b_1_5p,mmu_mi_r_29b_3p,mmu_mi_r_29c_3p,mmu_mi_r_29c_5p,mmu_mi_r_300_3p,mmu_mi_r_300_5p,mmu_mi_r_301a_5p,mmu_mi_r_301b_3p,mmu_mi_r_302a_3p,mmu_mi_r_302b_5p,mmu_mi_r_302c_3p,mmu_mi_r_302d_3p,mmu_mi_r_3058_5p,mmu_mi_r_3060_3p,mmu_mi_r_3061_5p,mmu_mi_r_3062_3p,mmu_mi_r_3063_3p,mmu_mi_r_3065_3p,mmu_mi_r_3066_5p,mmu_mi_r_3067_5p,mmu_mi_r_3068_5p,mmu_mi_r_3069_5p,mmu_mi_r_3070_5p,mmu_mi_r_3071_3p,mmu_mi_r_3073a_3p,mmu_mi_r_3073b_3p,mmu_mi_r_3074_2_3p,mmu_mi_r_3074_5p,mmu_mi_r_3075_3p,mmu_mi_r_3075_5p,mmu_mi_r_3076_3p,mmu_mi_r_3076_5p,mmu_mi_r_3078_3p,mmu_mi_r_3081_3p,mmu_mi_r_3081_5p,mmu_mi_r_3082_3p,mmu_mi_r_3083_5p,mmu_mi_r_3084_5p,mmu_mi_r_3085_5p,mmu_mi_r_3086_3p,mmu_mi_r_3087_3p,mmu_mi_r_3087_5p,mmu_mi_r_3088_5p,mmu_mi_r_3089_3p,mmu_mi_r_3089_5p,mmu_mi_r_3091_3p,mmu_mi_r_3091_5p,mmu_mi_r_3092_3p,mmu_mi_r_3093_3p,mmu_mi_r_3094_3p,mmu_mi_r_3094_5p,mmu_mi_r_3095_3p,mmu_mi_r_3098_3p,mmu_mi_r_3098_5p,mmu_mi_r_3099_3p,mmu_mi_r_3099_5p,mmu_mi_r_30a_3p,mmu_mi_r_30a_5p,mmu_mi_r_30b_3p,mmu_mi_r_30c_5p,mmu_mi_r_30d_3p,mmu_mi_r_30d_5p,mmu_mi_r_30e_3p,mmu_mi_r_30e_5p,mmu_mi_r_31_5p,mmu_mi_r_3102_3p_2_3p,mmu_mi_r_3102_5p,mmu_mi_r_3104_3p,mmu_mi_r_3104_5p,mmu_mi_r_3105_5p,mmu_mi_r_3106_5p,mmu_mi_r_3108_3p,mmu_mi_r_3108_5p,mmu_mi_r_3113_5p,mmu_mi_r_32_5p,mmu_mi_r_320_5p,mmu_mi_r_322_5p,mmu_mi_r_323_3p,mmu_mi_r_324_5p,mmu_mi_r_326_3p,mmu_mi_r_327,mmu_mi_r_328_3p,mmu_mi_r_329_3p,mmu_mi_r_329_5p,mmu_mi_r_33_5p,mmu_mi_r_330_3p,mmu_mi_r_331_3p,mmu_mi_r_331_5p,mmu_mi_r_335_5p,mmu_mi_r_337_3p,mmu_mi_r_338_3p,mmu_mi_r_339_5p,mmu_mi_r_340_5p,mmu_mi_r_341_5p,mmu_mi_r_342_3p,mmu_mi_r_344b_3p,mmu_mi_r_344e_3p,mmu_mi_r_344f_5p,mmu_mi_r_344g_5p,mmu_mi_r_344h_3p,mmu_mi_r_345_3p,mmu_mi_r_345_5p,mmu_mi_r_3470b,mmu_mi_r_3473b,mmu_mi_r_3473c,mmu_mi_r_3473f.,mmu_mi_r_3473g,mmu_mi_r_3474,mmu_mi_r_3475_3p,mmu_mi_r_34a_3p,mmu_mi_r_34a_5p,mmu_mi_r_34b_3p,mmu_mi_r_34b_5p,mmu_mi_r_34c_3p,mmu_mi_r_34c_5p,mmu_mi_r_351_3p,mmu_mi_r_351_5p,mmu_mi_r_3535,mmu_mi_r_3547_3p,mmu_mi_r_3547_5p,mmu_mi_r_3572_3p,mmu_mi_r_361_3p,mmu_mi_r_3618_3p,mmu_mi_r_3618_5p,mmu_mi_r_362_5p,mmu_mi_r_3620_3p,mmu_mi_r_365_2_5p,mmu_mi_r_365_3p,mmu_mi_r_367_3p,mmu_mi_r_367_5p,mmu_mi_r_369_3p,mmu_mi_r_370_3p,mmu_mi_r_370_5p,mmu_mi_r_374b_5p,mmu_mi_r_374c_3p,mmu_mi_r_375_3p,mmu_mi_r_376a_3p,mmu_mi_r_376b_3p,mmu_mi_r_376b_5p,mmu_mi_r_376c_3p,mmu_mi_r_377_5p,mmu_mi_r_380_5p,mmu_mi_r_382_3p,mmu_mi_r_383_5p,mmu_mi_r_384_3p,mmu_mi_r_384_5p,mmu_mi_r_3961,mmu_mi_r_3962,mmu_mi_r_3964,mmu_mi_r_3965,mmu_mi_r_3967,mmu_mi_r_3968,mmu_mi_r_3970,mmu_mi_r_409_3p,mmu_mi_r_410_3p,mmu_mi_r_411_5p,mmu_mi_r_412_5p,mmu_mi_r_421_3p,mmu_mi_r_421_5p,mmu_mi_r_423_3p,mmu_mi_r_423_5p,mmu_mi_r_425_3p,mmu_mi_r_429_3p,mmu_mi_r_429_5p,mmu_mi_r_431_3p,mmu_mi_r_431_5p,mmu_mi_r_432,mmu_mi_r_433_5p,mmu_mi_r_434_3p,mmu_mi_r_448_3p,mmu_mi_r_449a_3p,mmu_mi_r_449a_5p,mmu_mi_r_449b,mmu_mi_r_450a_2_3p,mmu_mi_r_450b_3p,mmu_mi_r_451a,mmu_mi_r_452_3p,mmu_mi_r_452_5p,mmu_mi_r_465d_3p,mmu_mi_r_465d_5p,mmu_mi_r_466a_3p_mmu_mi_r_466e_3p,mmu_mi_r_466b_3p_mmu_mi_r_466c_3p_mmu_mi_r_466p_3p,mmu_mi_r_466j,mmu_mi_r_466l_3p,mmu_mi_r_466m_3p,mmu_mi_r_466q,mmu_mi_r_467d_5p,mmu_mi_r_467f.,mmu_mi_r_470_5p,mmu_mi_r_471_5p,mmu_mi_r_485_5p,mmu_mi_r_486a_3p,mmu_mi_r_486b_3p,mmu_mi_r_486b_5p,mmu_mi_r_488_3p,mmu_mi_r_488_5p,mmu_mi_r_489_3p,mmu_mi_r_489_5p,mmu_mi_r_490_3p,mmu_mi_r_490_5p,mmu_mi_r_493_3p,mmu_mi_r_493_5p,mmu_mi_r_494_5p,mmu_mi_r_496a_3p,mmu_mi_r_496a_5p,mmu_mi_r_497a_5p,mmu_mi_r_500_3p,mmu_mi_r_500_5p,mmu_mi_r_501_5p,mmu_mi_r_503_3p,mmu_mi_r_503_5p,mmu_mi_r_5046,mmu_mi_r_505_3p,mmu_mi_r_505_5p,mmu_mi_r_509_3p,mmu_mi_r_509_5p,mmu_mi_r_5098,mmu_mi_r_5100,mmu_mi_r_5101,mmu_mi_r_5103,mmu_mi_r_5104,mmu_mi_r_5106,mmu_mi_r_5107_3p,mmu_mi_r_5107_5p,mmu_mi_r_511_3p,mmu_mi_r_511_5p,mmu_mi_r_5113,mmu_mi_r_5116,mmu_mi_r_5118,mmu_mi_r_5119,mmu_mi_r_5120,mmu_mi_r_5122,mmu_mi_r_5124a,mmu_mi_r_5124b,mmu_mi_r_5125,mmu_mi_r_5127,mmu_mi_r_5129_5p,mmu_mi_r_5132_3p,mmu_mi_r_5132_5p,mmu_mi_r_532_3p,mmu_mi_r_532_5p,mmu_mi_r_542_3p,mmu_mi_r_542_5p,mmu_mi_r_543_3p,mmu_mi_r_543_5p,mmu_mi_r_546,mmu_mi_r_551b_3p,mmu_mi_r_5616_3p,mmu_mi_r_5616_5p,mmu_mi_r_5617_3p,mmu_mi_r_5618_3p,mmu_mi_r_5618_5p,mmu_mi_r_5619_3p,mmu_mi_r_5620_5p,mmu_mi_r_5621_5p,mmu_mi_r_5623_3p,mmu_mi_r_5625_5p,mmu_mi_r_5626_3p,mmu_mi_r_5626_5p,mmu_mi_r_5627_3p,mmu_mi_r_5627_5p,mmu_mi_r_568,mmu_mi_r_5709_3p,mmu_mi_r_5709_5p,mmu_mi_r_574_3p,mmu_mi_r_582_3p,mmu_mi_r_590_5p,mmu_mi_r_592_3p,mmu_mi_r_598_3p,mmu_mi_r_599,mmu_mi_r_615_3p,mmu_mi_r_615_5p,mmu_mi_r_6236,mmu_mi_r_6237,mmu_mi_r_6239,mmu_mi_r_6241,mmu_mi_r_6244,mmu_mi_r_6335,mmu_mi_r_6337,mmu_mi_r_6341,mmu_mi_r_6342,mmu_mi_r_6344,mmu_mi_r_6349,mmu_mi_r_6352,mmu_mi_r_6356,mmu_mi_r_6357,mmu_mi_r_6358,mmu_mi_r_6360,mmu_mi_r_6361,mmu_mi_r_6362,mmu_mi_r_6363,mmu_mi_r_6367,mmu_mi_r_6368,mmu_mi_r_6369,mmu_mi_r_6371,mmu_mi_r_6372,mmu_mi_r_6374,mmu_mi_r_6378,mmu_mi_r_6379,mmu_mi_r_6381,mmu_mi_r_6382,mmu_mi_r_6383,mmu_mi_r_6384,mmu_mi_r_6385,mmu_mi_r_6386,mmu_mi_r_6387,mmu_mi_r_6388,mmu_mi_r_6390,mmu_mi_r_6391,mmu_mi_r_6392_3p,mmu_mi_r_6394,mmu_mi_r_6397,mmu_mi_r_6399,mmu_mi_r_6401,mmu_mi_r_6403,mmu_mi_r_6405,mmu_mi_r_6406,mmu_mi_r_6407,mmu_mi_r_6408,mmu_mi_r_6409,mmu_mi_r_6410,mmu_mi_r_6411,mmu_mi_r_6412,mmu_mi_r_6413,mmu_mi_r_6418_3p,mmu_mi_r_6418_5p,mmu_mi_r_6419,mmu_mi_r_6481,mmu_mi_r_6516_3p,mmu_mi_r_6516_5p,mmu_mi_r_652_3p,mmu_mi_r_653_3p,mmu_mi_r_6538,mmu_mi_r_654_3p,mmu_mi_r_654_5p,mmu_mi_r_6540_3p,mmu_mi_r_6546_3p,mmu_mi_r_6546_5p,mmu_mi_r_664_5p,mmu_mi_r_667_3p,mmu_mi_r_667_5p,mmu_mi_r_668_3p,mmu_mi_r_668_5p,mmu_mi_r_669a_3p_mmu_mi_r_669o_3p,mmu_mi_r_669a_5p_mmu_mi_r_669p_5p,mmu_mi_r_669b_5p,mmu_mi_r_669c_5p,mmu_mi_r_669f_3p,mmu_mi_r_669f_5p,mmu_mi_r_669h_5p,mmu_mi_r_669i,mmu_mi_r_669m_3p,mmu_mi_r_669n,mmu_mi_r_669p_3p,mmu_mi_r_670_3p,mmu_mi_r_670_5p,mmu_mi_r_671_3p,mmu_mi_r_671_5p,mmu_mi_r_6715_3p,mmu_mi_r_673_5p,mmu_mi_r_674_3p,mmu_mi_r_674_5p,mmu_mi_r_676_3p,mmu_mi_r_677_5p,mmu_mi_r_681,mmu_mi_r_684,mmu_mi_r_687,mmu_mi_r_688,mmu_mi_r_6896_3p,mmu_mi_r_6897_3p,mmu_mi_r_6897_5p,mmu_mi_r_6898_3p,mmu_mi_r_6898_5p,mmu_mi_r_6899_3p,mmu_mi_r_6900_3p,mmu_mi_r_6902_5p,mmu_mi_r_6903_3p,mmu_mi_r_6903_5p,mmu_mi_r_6904_3p,mmu_mi_r_6904_5p,mmu_mi_r_6907_3p,mmu_mi_r_6907_5p,mmu_mi_r_691,mmu_mi_r_6910_5p,mmu_mi_r_6911_5p,mmu_mi_r_6912_3p,mmu_mi_r_6914_5p,mmu_mi_r_6916_3p,mmu_mi_r_6917_3p,mmu_mi_r_6918_3p,mmu_mi_r_6918_5p,mmu_mi _r_6919_3p,mmu_mi_r_6919_5p,mmu_mi_r_692,mmu_mi_r_6920_5p,mmu_mi_r_6923_5p,mmu_mi_r_6924_3p,mmu_mi_r_6926_5p,mmu_mi_r_6927_5p,mmu_mi_r_6928_3p,mmu_mi_r_6929_3p,mmu_mi_r_6929_5p,mmu_mi_r_693_5p,mmu_mi_r_6931_5p,mmu_mi_r_6932_3p,mmu_mi_r_6932_5p,mmu_mi_r_6935_3p,mmu_mi_r_6935_5p,mmu_mi_r_6936_3p,mmu_mi_r_6936_5p,mmu_mi_r_6937_3p,mmu_mi_r_6938_3p,mmu_mi_r_6938_5p,mmu_mi_r_6939_3p,mmu_mi_r_6939_5p,mmu_mi_r_694,mmu_mi_r_6940_3p,mmu_mi_r_6940_5p,mmu_mi_r_6941_3p,mmu_mi_r_6941_5p,mmu_mi_r_6944_5p,mmu_mi_r_6946_5p,mmu_mi_r_6948_5p,mmu_mi_r_6949_3p,mmu_mi_r_6949_5p,mmu_mi_r_6950_3p,mmu_mi_r_6951_5p,mmu_mi_r_6953_3p,mmu_mi_r_6954_3p,mmu_mi_r_6954_5p,mmu_mi_r_6955_5p,mmu_mi_r_6956_5p,mmu_mi_r_6958_3p,mmu_mi_r_6959_3p,mmu_mi_r_6961_5p,mmu_mi_r_6962_5p,mmu_mi_r_6964_3p,mmu_mi_r_6965_3p,mmu_mi_r_6965_5p,mmu_mi_r_6966_3p,mmu_mi_r_6966_5p,mmu_mi_r_6967_5p,mmu_mi_r_6968_3p,mmu_mi_r_6968_5p,mmu_mi_r_6969_3p,mmu_mi_r_6969_5p,mmu_mi_r_697,mmu_mi_r_6970_3p,mmu_mi_r_6970_5p,mmu_mi_r_6971_5p,mmu_mi_r_6972_3p,mmu_mi_r_6973a_3p,mmu_mi_r_6973b_3p,mmu_mi_r_6973b_5p,mmu_mi_r_6974_5p,mmu_mi_r_6976_3p,mmu_mi_r_6976_5p,mmu_mi_r_6979_5p,mmu_mi_r_698_3p,mmu_mi_r_698_5p,mmu_mi_r_6980_3p,mmu_mi_r_6980_5p,mmu_mi_r_6981_5p,mmu_mi_r_6982_5p,mmu_mi_r_6984_3p,mmu_mi_r_6984_5p,mmu_mi_r_6986_5p,mmu_mi_r_6987_5p,mmu_mi_r_6990_3p,mmu_mi_r_6991_3p,mmu_mi_r_6992_3p,mmu_mi_r_6992_5p,mmu_mi_r_6993_3p,mmu_mi_r_6996_3p,mmu_mi_r_6997_3p,mmu_mi_r_6997_5p,mmu_mi_r_6998_3p,mmu_mi_r_6999_3p,mmu_mi_r_700_5p,mmu_mi_r_7000_5p,mmu_mi_r_7001_3p,mmu_mi_r_7002_3p,mmu_mi_r_7002_5p,mmu_mi_r_7003_5p,mmu_mi_r_7004_3p,mmu_mi_r_7004_5p,mmu_mi_r_7005_3p,mmu_mi_r_7005_5p,mmu_mi_r_7006_5p,mmu_mi_r_7007_3p,mmu_mi_r_7007_5p,mmu_mi_r_7008_5p,mmu_mi_r_701_5p,mmu_mi_r_7010_5p,mmu_mi_r_7012_3p,mmu_mi_r_7013_3p,mmu_mi_r_7014_3p,mmu_mi_r_7015_3p,mmu_mi_r_7015_5p,mmu_mi_r_7016_3p,mmu_mi_r_7017_3p,mmu_mi_r_7018_3p,mmu_mi_r_7019_3p,mmu_mi_r_7019_5p,mmu_mi_r_7020_3p,mmu_mi_r_7021_3p,mmu_mi_r_7021_5p,mmu_mi_r_7024_5p,mmu_mi_r_7029_3p,mmu_mi_r_7029_5p,mmu_mi_r_703,mmu_mi_r_7030_3p,mmu_mi_r_7031_3p,mmu_mi_r_7033_3p,mmu_mi_r_7034_5p,mmu_mi_r_7035_5p,mmu_mi_r_7036b_5p,mmu_mi_r_7037_3p,mmu_mi_r_7037_5p,mmu_mi_r_7038_5p,mmu_mi_r_7039_5p,mmu_mi_r_704,mmu_mi_r_7041_5p,mmu_mi_r_7042_3p,mmu_mi_r_7046_5p,mmu_mi_r_7047_3p,mmu_mi_r_7049_3p,mmu_mi_r_7050_3p,mmu_mi_r_7050_5p,mmu_mi_r_7051_3p,mmu_mi_r_7054_3p,mmu_mi_r_7056_3p,mmu_mi_r_7056_5p,mmu_mi_r_7057_3p,mmu_mi_r_7057_5p,mmu_mi_r_7058_3p,mmu_mi_r_7058_5p,mmu_mi_r_7059_3p,mmu_mi_r_7060_3p,mmu_mi_r_7060_5p,mmu_mi_r_7061_5p,mmu_mi_r_7062_3p,mmu_mi_r_7062_5p,mmu_mi_r_7064_5p,mmu_mi_r_7065_3p,mmu_mi_r_7066_5p,mmu_mi_r_7067_3p,mmu_mi_r_7068_5p,mmu_mi_r_7069_5p,mmu_mi_r_707,mmu_mi_r_7070_3p,mmu_mi_r_7074_5p,mmu_mi_r_7076_5p,mmu_mi_r_7077_3p,mmu_mi_r_708_5p,mmu_mi_r_7080_5p,mmu_mi_r_7082_3p,mmu_mi_r_7084_5p,mmu_mi_r_7085_5p,mmu_mi_r_7087_3p,mmu_mi_r_7089_5p,mmu_mi_r_709,mmu_mi_r_7090_3p,mmu_mi_r_7091_5p,mmu_mi_r_7092_5p,mmu_mi_r_7093_3p,mmu_mi_r_7094_1_5p,mmu_mi_r_7094_3p,mmu_mi_r_7094b_2_5p,mmu_mi_r_710,mmu_mi_r_7117_3p,mmu_mi_r_7117_5p,mmu_mi_r_7119_3p,mmu_mi_r_721,mmu_mi_r_7210_3p,mmu_mi_r_7210_5p,mmu_mi_r_7211_3p,mmu_mi_r_7211_5p,mmu_mi_r_7212_5p,mmu_mi_r_7213_3p,mmu_mi_r_7213_5p,mmu_mi_r_7215_5p,mmu_mi_r_7216_5p,mmu_mi_r_7219_3p,mmu_mi_r_7219_5p,mmu_mi_r_7220_3p,mmu_mi_r_7221_3p,mmu_mi_r_7221_5p,mmu_mi_r_7222_3p,mmu_mi_r_7223_5p,mmu_mi_r_7224_3p,mmu_mi_r_7225_5p,mmu_mi_r_7226_5p,mmu_mi_r_7227_5p,mmu_mi_r_7228_5p,mmu_mi_r_7229_3p,mmu_mi_r_7230_3p,mmu_mi_r_7230_5p,mmu_mi_r_7232_3p,mmu_mi_r_7232_5p,mmu_mi_r_7233_5p,mmu_mi_r_7235_5p,mmu_mi_r_7237_5p,mmu_mi_r_7239_5p,mmu_mi_r_7240_5p,mmu_mi_r_7243_5p,mmu_mi_r_741_5p,mmu_mi_r_743a_3p,mmu_mi_r_743a_5p,mmu_mi_r_743b_3p,mmu_mi_r_744_3p,mmu_mi_r_744_5p,mmu_mi_r_758_3p,mmu_mi_r_758_5p,mmu_mi_r_761,mmu_mi_r_763,mmu_mi_r_764_3p,mmu_mi_r_7646_5p,mmu_mi_r_7648_5p,mmu_mi_r_7649_3p,mmu_mi_r_7650_3p,mmu_mi_r_7650_5p,mmu_mi_r_7651_5p,mmu_mi_r_7652_3p,mmu_mi_r_7652_5p,mmu_mi_r_7654_3p,mmu_mi_r_7654_5p,mmu_mi_r_7655_3p,mmu_mi_r_7656_3p,mmu_mi_r_7657_3p,mmu_mi_r_7658_3p,mmu_mi_r_7659_3p,mmu_mi_r_7660_5p,mmu_mi_r_7661_3p,mmu_mi_r_7662_5p,mmu_mi_r_7663_5p,mmu_mi_r_7664_3p,mmu_mi_r_7664_5p,mmu_mi_r_7666_5p,mmu_mi_r_7668_3p,mmu_mi_r_7668_5p,mmu_mi_r_7669_3p,mmu_mi_r_7669_5p,mmu_mi_r_767,mmu_mi_r_7670_3p,mmu_mi_r_7671_3p,mmu_mi_r_7673_3p,mmu_mi_r_7674_3p,mmu_mi_r_7675_3p,mmu_mi_r_7677_3p,mmu_mi_r_7677_5p,mmu_mi_r_7678_3p,mmu_mi_r_7679_3p,mmu_mi_r_7680_5p,mmu_mi_r_7681_3p,mmu_mi_r_7682_3p,mmu_mi_r_7682_5p,mmu_mi_r_7683_3p,mmu_mi_r_7683_5p,mmu_mi_r_7684_3p,mmu_mi_r_7685_3p,mmu_mi_r_7687_3p,mmu_mi_r_7687_5p,mmu_mi_r_7688_5p,mmu_mi_r_7689_5p,mmu_mi_r_770_3p,mmu_mi_r_7a_5p,mmu_mi_r_7b_3p,mmu_mi_r_7b_5p,mmu_mi_r_802_3p,mmu_mi_r_802_5p,mmu_mi_r_8090,mmu_mi_r_8091,mmu_mi_r_8094,mmu_mi_r_8096,mmu_mi_r_8098,mmu_mi_r_8099,mmu_mi_r_8100,mmu_mi_r_8101,mmu_mi_r_8103,mmu_mi_r_8106,mmu_mi_r_8107,mmu_mi_r_8108,mmu_mi_r_8109,mmu_mi_r_8110,mmu_mi_r_8112,mmu_mi_r_8116,mmu_mi_r_8118,mmu_mi_r_8120,mmu_mi_r_871_3p,mmu_mi_r_872_3p,mmu_mi_r_873a_5p,mmu_mi_r_874_3p,mmu_mi_r_875_3p,mmu_mi_r_875_5p,mmu_mi_r_876_3p,mmu_mi_r_877_3p,mmu_mi_r_879_5p,mmu_mi_r_880_3p,mmu_mi_r_880_5p,mmu_mi_r_881_3p,mmu_mi_r_883a_5p,mmu_mi_r_9_3p,mmu_mi_r_9_5p,mmu_mi_r_92a_2_5p,mmu_mi_r_92b_3p,mmu_mi_r_92b_5p,mmu_mi_r_93_3p,mmu_mi_r_93_5p,mmu_mi_r_935,mmu_mi_r_96_3p,mmu_mi_r_98_3p,mmu_mi_r_98_5p,mmu_mi_r_99a_3p,mmu_mi_r_99b_3p,mmu_mi_r_99b_5p,mmu_pi_r_000159_gb_dq539904_mus_musculus_2_73668844_73668871_plus,mmu_pi_r_000159_gb_dq539904_mus_musculus_6_87962871_87962898_minus,mmu_pi_r_000159_gb_dq539904_mus_musculus_9_118523651_118523678_minus,mmu_pi_r_000366_gb_dq540412_mus_musculus_17_25602713_25602739_plus,mmu_pi_r_000366_gb_dq540412_mus_musculus_6_86369552_86369578_plus,mmu_pi_r_000616_gb_dq540965_mus_musculus_2_5296571_5296602_minus,mmu_pi_r_000616_gb_dq540965_mus_musculus_3_5843413_5843444_plus,mmu_pi_r_000620_gb_dq540981_mus_musculus_18_54824345_54824374_minus,mmu_pi_r_000620_gb_dq540981_mus_musculus_3_5843782_5843811_plus,mmu_pi_r_000622_gb_dq540988_mus_musculus_18_54824707_54824734_minus,mmu_pi_r_000622_gb_dq540988_mus_musculus_3_5843428_5843455_plus,mmu_pi_r_000622_gb_dq540988_mus_musculus_x_112404287_112404314_minus,mmu_pi_r_000691_gb_dq541218_mus_musculus_8_126462165_126462190_minus,mmu_pi_r_000935_gb_dq541777_mus_musculus_6_47717737_47717766_minus,mmu_pi_r_000958_gb_dq541851_mus_musculus_17_39456228_39456254_plus,mmu_pi_r_001570_gb_dq543701_mus_musculus_3_5843412_5843441_plus,mmu_pi_r_002435_gb_dq546549_mus_musculus_17_39454808_39454832_plus,mmu_pi_r_003862_gb_dq551041_mus_musculus_4_3762678_3762703_minus,mmu_pi_r_004374_gb_dq552696_mus_musculus_18_85832427_85832456_minus,mmu_pi_r_009321_gb_dq684704_mus_musculus_7_72988380_72988411_plus,mmu_pi_r_009467_gb_dq684969_mus_musculus_7_73682766_73682794_minus,mmu_pi_r_009574_gb_dq685137_mus_musculus_15_59111741_59111771_plus,mmu_pi_r_010309_gb_dq686298_mus_musculus_1_161177076_161177096_plus,mmu_pi_r_010565_gb_dq686723_mus_musculus_16_38309443_38309460_plus,mmu_pi_r_012641_gb_dq689910_mus_musculus_17_27048950_27048977_minus,mmu_pi_r_013503_gb_dq691233_mus_musculus_8_24061664_24061692_plus,mmu_pi_r_017289_gb_dq696831_mus_musculus_6_128804704_128804734_plus,mmu_pi_r_017405_gb_dq696996_mus_musculus_11_65550994_65551015_minus,mmu_pi_r_020492_gb_dq701563_mus_musculus_11_108827972_108827997_minus,mmu_pi_r_020692_gb_dq701869_mus_musculus_5_115840031_115840052_plus,mmu_pi_r_022097_gb_dq703900_mus_musculus_6_86369527_86369558_plus,mmu_pi_r_022956_gb_dq705141_mus_musculus_5_115112809_115112837_minus,mmu_pi_r_023799_gb_dq706399_mus_musculus_2_150950762_150950792_minus,mmu_pi_r_025576_gb_dq708952_mus_musculus_x_6405415_6405436_minus,mmu_pi_r_027673_gb_dq711996_mus_musculus_11_106317081_106317102_plus,mmu_pi_r_028252_gb_dq712837_mus_musculus_7_81403433_81403455_plus,mmu_pi_r_028903_gb_dq713755_mus_musculus_1_109786123_109786152_minus,mmu_pi_r_028975_gb_dq713872_mus_musculus_x_6405378_6405399_minus,mmu_pi_r_029303_gb_dq714361_mus_musculus_10_18490237_18490267_minus,mmu_pi_r_032865_gb_dq719430_mus_musculus_2_116876592_116876614_plus,mmu_pi_r_032974_gb_dq719597_mus_musculus_4_130021751_130021778_plus,mmu_pi_r_036119_gb_dq724251_mus_musculus_2_71134696_71134717_plus,mmu_pi_r_038323_gb_pi_rna_t34_mus_musculus_13_44880547_44880577_minus,mmu_pi_r_038323_gb_pi_rna_t34_mus_musculus_9_3258895_3258925_minus,mmu_pi_r_038328_gb_pi_rna_t47_mus_musculus_19_13121850_13121879_plus,mmu_pi_r_038328_gb_pi_rna_t47_mus_musculus_6_3151091_3151120_plus,mmu_pi_r_038328_gb_pi_rna_t47_mus_musculus_x_23165477_23165506_plus
290.087	x290_087_A	mmu_let_7a_1_3p_mmu_let_7c_2_3p,mmu_let_7b_3p,mmu_let_7c_1_3p,mmu_let_7c_5p,mmu_let_7d_5p,mmu_let_7e_5p,mmu_let_7f_2_3p,mmu_let_7g_3p,mmu_let_7i_5p,mmu_let_7j,mmu_let_7k,mmu_mi_r_100_5p,mmu_mi_r_101a_3p,mmu_mi_r_101a_5p,mmu_mi_r_101b_3p,mmu_mi_r_101b_5p,mmu_mi_r_101c,mmu_mi_r_103_1_5p,mmu_mi_r_103_2_5p,mmu_mi_r_103_3p,mmu_mi_r_106a_3p,mmu_mi_r_106b_3p,mmu_mi_r_107_3p,mmu_mi_r_10a_3p,mmu_mi_r_10b_3p,mmu_mi_r_1188_5p,mmu_mi_r_1191a,mmu_mi_r_1191b_3p,mmu_mi_r_1191b_5p,mmu_mi_r_1192,mmu_mi_r_1193_3p,mmu_mi_r_1195,mmu_mi_r_1198_5p,mmu_mi_r_1199_5p,mmu_mi_r_122_3p,mmu_mi_r_1231_3p,mmu_mi_r_124_3p,mmu_mi_r_1247_3p,mmu_mi_r_1249_5p,mmu_mi_r_1251_3p,mmu_mi_r_1251_5p,mmu_mi_r_1258_3p,mmu_mi_r_1258_5p,mmu_mi_r_125a_3p,mmu_mi_r_125b_1_3p,mmu_mi_r_1264_5p,mmu_mi_r_127_3p,mmu_mi_r_128_1_5p,mmu_mi_r_129_1_3p,mmu_mi_r_1291,mmu_mi_r_1298_3p,mmu_mi_r_129b_5p,mmu_mi_r_1306_3p,mmu_mi_r_130c,mmu_mi_r_133a_5p,mmu_mi_r_134_3p,mmu_mi_r_135a_5p,mmu_mi_r_135b_5p,mmu_mi_r_137_3p,mmu_mi_r_137_5p,mmu_mi_r_138_2_3p,mmu_mi_r_139_3p,mmu_mi_r_140_3p,mmu_mi_r_141_3p,mmu_mi_r_143_3p,mmu_mi_r_144_5p,mmu_mi_r_145a_3p,mmu_mi_r_147_5p,mmu_mi_r_148a_5p,mmu_mi_r_148b_3p,mmu_mi_r_149_3p,mmu_mi_r_151_5p,mmu_mi_r_154_5p,mmu_mi_r_155_3p,mmu_mi_r_16_2_3p,mmu_mi_r_16_5p,mmu_mi_r_1668,mmu_mi_r_17_3p,mmu_mi_r_181a_1_3p,mmu_mi_r_181b_1_3p,mmu_mi_r_181b_2_3p,mmu_mi_r_181b_5p,mmu_mi_r_181c_3p,mmu_mi_r_182_3p,mmu_mi_r_1839_5p,mmu_mi_r_184_3p,mmu_mi_r_184_5p,mmu_mi_r_1843a_3p,mmu_mi_r_1843b_3p,mmu_mi_r_1843b_5p,mmu_mi_r_185_3p,mmu_mi_r_186_3p,mmu_mi_r_187_5p,mmu_mi_r_1893,mmu_mi_r_1894_3p,mmu_mi_r_1896,mmu_mi_r_1897_5p,mmu_mi_r_1898,mmu_mi_r_18b_5p,mmu_mi_r_1900,mmu_mi_r_1901,mmu_mi_r_1902,mmu_mi_r_1907,mmu_mi_r_190b_5p,mmu_mi_r_191_3p,mmu_mi_r_1912_3p,mmu_mi_r_192_3p,mmu_mi_r_1929_5p,mmu_mi_r_1930_3p,mmu_mi_r_1931,mmu_mi_r_1933_5p,mmu_mi_r_1934_5p,mmu_mi_r_1938,mmu_mi_r_1940,mmu_mi_r_1941_3p,mmu_mi_r_1943_3p,mmu_mi_r_1943_5p,mmu_mi_r_1946a,mmu_mi_r_1946b,mmu_mi_r_1947_5p,mmu_mi_r_1948_3p,mmu_mi_r_1951,mmu_mi_r_1954,mmu_mi_r_1957a,mmu_mi_r_1957b,mmu_mi_r_195a_3p,mmu_mi_r_195a_5p,mmu_mi_r_1961,mmu_mi_r_1962,mmu_mi_r_1966_5p,mmu_mi_r_1968_3p,mmu_mi_r_196a_1_3p,mmu_mi_r_196a_2_3p,mmu_mi_r_196b_3p,mmu_mi_r_1981_5p,mmu_mi_r_1982_3p,mmu_mi_r_1983,mmu_mi_r_199b_5p,mmu_mi_r_200c_3p,mmu_mi_r_203_5p,mmu_mi_r_204_3p,mmu_mi_r_204_5p,mmu_mi_r_205_3p,mmu_mi_r_206_3p,mmu_mi_r_207,mmu_mi_r_208b_3p,mmu_mi_r_20a_3p,mmu_mi_r_20b_3p,mmu_mi_r_20b_5p,mmu_mi_r_210_5p,mmu_mi_r_212_3p,mmu_mi_r_212_5p,mmu_mi_r_216a_5p,mmu_mi_r_216b_3p,mmu_mi_r_216b_5p,mmu_mi_r_216c_5p,mmu_mi_r_217_3p,mmu_mi_r_218_2_3p,mmu_mi_r_218_5p,mmu_mi_r_2183,mmu_mi_r_219a_1_3p,mmu_mi_r_219a_5p,mmu_mi_r_219b_3p,mmu_mi_r_219b_5p,mmu_mi_r_219c_3p,mmu_mi_r_219c_5p,mmu_mi_r_21a_3p,mmu_mi_r_21a_5p,mmu_mi_r_21b,mmu_mi_r_21c,mmu_mi_r_221_5p,mmu_mi_r_224_3p,mmu_mi_r_23a_5p,mmu_mi_r_23b_5p,mmu_mi_r_25_3p,mmu_mi_r_25_5p,mmu_mi_r_27a_5p,mmu_mi_r_28a_5p,mmu_mi_r_28b,mmu_mi_r_290a_3p,mmu_mi_r_291b_5p,mmu_mi_r_292a_3p,mmu_mi_r_293_3p,mmu_mi_r_294_3p,mmu_mi_r_297b_5p,mmu_mi_r_299a_3p,mmu_mi_r_29a_3p,mmu_mi_r_29a_5p,mmu_mi_r_29b_1_5p,mmu_mi_r_29b_3p,mmu_mi_r_29c_3p,mmu_mi_r_301a_3p,mmu_mi_r_301b_5p,mmu_mi_r_302a_5p,mmu_mi_r_302c_3p,mmu_mi_r_302c_5p,mmu_mi_r_302d_5p,mmu_mi_r_3057_3p,mmu_mi_r_3057_5p,mmu_mi_r_3058_5p,mmu_mi_r_3059_3p,mmu_mi_r_3060_5p,mmu_mi_r_3063_5p,mmu_mi_r_3064_5p,mmu_mi_r_3065_3p,mmu_mi_r_3066_3p,mmu_mi_r_3067_3p,mmu_mi_r_3070_2_3p,mmu_mi_r_3070_3p,mmu_mi_r_3071_5p,mmu_mi_r_3072_3p,mmu_mi_r_3073a_5p,mmu_mi_r_3073b_5p,mmu_mi_r_3074_1_3p,mmu_mi_r_3076_3p,mmu_mi_r_3077_3p,mmu_mi_r_3078_3p,mmu_mi_r_3078_5p,mmu_mi_r_3080_3p,mmu_mi_r_3082_3p,mmu_mi_r_3085_5p,mmu_mi_r_3089_3p,mmu_mi_r_3090_3p,mmu_mi_r_3091_3p,mmu_mi_r_3093_5p,mmu_mi_r_3094_3p,mmu_mi_r_3094_5p,mmu_mi_r_3095_3p,mmu_mi_r_3098_3p,mmu_mi_r_3098_5p,mmu_mi_r_30a_3p,mmu_mi_r_30a_5p,mmu_mi_r_30b_3p,mmu_mi_r_30c_1_3p,mmu_mi_r_30c_2_3p,mmu_mi_r_30d_3p,mmu_mi_r_30d_5p,mmu_mi_r_30e_3p,mmu_mi_r_30f.,mmu_mi_r_3100_5p,mmu_mi_r_3101_3p,mmu_mi_r_3103_3p,mmu_mi_r_3103_5p,mmu_mi_r_3105_3p,mmu_mi_r_3105_5p,mmu_mi_r_3108_5p,mmu_mi_r_3112_3p,mmu_mi_r_3112_5p,mmu_mi_r_32_3p,mmu_mi_r_320_5p,mmu_mi_r_322_3p,mmu_mi_r_322_5p,mmu_mi_r_324_5p,mmu_mi_r_328_5p,mmu_mi_r_329_3p,mmu_mi_r_33_3p,mmu_mi_r_331_3p,mmu_mi_r_331_5p,mmu_mi_r_335_5p,mmu_mi_r_337_5p,mmu_mi_r_338_3p,mmu_mi_r_339_3p,mmu_mi_r_340_3p,mmu_mi_r_340_5p,mmu_mi_r_343,mmu_mi_r_344_3p,mmu_mi_r_344_5p,mmu_mi_r_344b_3p,mmu_mi_r_344b_5p,mmu_mi_r_344c_3p,mmu_mi_r_344d_2_5p,mmu_mi_r_344f_3p,mmu_mi_r_344f_5p,mmu_mi_r_344g_3p,mmu_mi_r_344h_3p,mmu_mi_r_345_3p,mmu_mi_r_3470a,mmu_mi_r_3470b,mmu_mi_r_3471,mmu_mi_r_3472,mmu_mi_r_3473a,mmu_mi_r_3473d,mmu_mi_r_3473g,mmu_mi_r_3474,mmu_mi_r_3475_3p,mmu_mi_r_3475_5p,mmu_mi_r_34c_3p,mmu_mi_r_350_3p,mmu_mi_r_350_5p,mmu_mi_r_3535,mmu_mi_r_3544_3p,mmu_mi_r_3547_3p,mmu_mi_r_3547_5p,mmu_mi_r_3552,mmu_mi_r_3572_5p,mmu_mi_r_361_3p,mmu_mi_r_3618_3p,mmu_mi_r_3618_5p,mmu_mi_r_3620_3p,mmu_mi_r_3620_5p,mmu_mi_r_363_5p,mmu_mi_r_365_1_5p,mmu_mi_r_365_2_5p,mmu_mi_r_365_3p,mmu_mi_r_367_3p,mmu_mi_r_369_3p,mmu_mi_r_370_3p,mmu_mi_r_370_5p,mmu_mi_r_374b_3p,mmu_mi_r_374c_3p,mmu_mi_r_374c_5p,mmu_mi_r_376a_3p,mmu_mi_r_376a_5p,mmu_mi_r_376c_5p,mmu_mi_r_378b,mmu_mi_r_378c,mmu_mi_r_378d,mmu_mi_r_379_5p,mmu_mi_r_383_3p,mmu_mi_r_383_5p,mmu_mi_r_384_3p,mmu_mi_r_3965,mmu_mi_r_3967,mmu_mi_r_3970,mmu_mi_r_412_3p,mmu_mi_r_429_5p,mmu_mi_r_431_3p,mmu_mi_r_433_3p,mmu_mi_r_433_5p,mmu_mi_r_448_3p,mmu_mi_r_448_5p,mmu_mi_r_450a_1_3p,mmu_mi_r_450a_5p,mmu_mi_r_450b_5p,mmu_mi_r_451b,mmu_mi_r_455_3p,mmu_mi_r_465b_5p,mmu_mi_r_465d_5p,mmu_mi_r_466i_3p,mmu_mi_r_466j,mmu_mi_r_466k,mmu_mi_r_466l_3p,mmu_mi_r_466m_3p,mmu_mi_r_466n_3p,mmu_mi_r_467b_3p,mmu_mi_r_467d_3p,mmu_mi_r_467e_5p,mmu_mi_r_468_3p,mmu_mi_r_470_5p,mmu_mi_r_471_3p,mmu_mi_r_486a_3p,mmu_mi_r_486a_5p,mmu_mi_r_486b_3p,mmu_mi_r_487b_5p,mmu_mi_r_488_3p,mmu_mi_r_488_5p,mmu_mi_r_489_3p,mmu_mi_r_490_3p,mmu_mi_r_491_5p,mmu_mi_r_493_5p,mmu_mi_r_495_5p,mmu_mi_r_496b,mmu_mi_r_497a_3p,mmu_mi_r_499_3p,mmu_mi_r_500_5p,mmu_mi_r_501_5p,mmu_mi_r_503_3p,mmu_mi_r_503_5p,mmu_mi_r_504_3p,mmu_mi_r_504_5p,mmu_mi_r_505_3p,mmu_mi_r_509_3p,mmu_mi_r_5100,mmu_mi_r_5101,mmu_mi_r_5103,mmu_mi_r_5104,mmu_mi_r_5106,mmu_mi_r_511_3p,mmu_mi_r_511_5p,mmu_mi_r_5119,mmu_mi_r_5121,mmu_mi_r_5123,mmu_mi_r_5124b,mmu_mi_r_5131,mmu_mi_r_5132_3p,mmu_mi_r_5134_3p,mmu_mi_r_5134_5p,mmu_mi_r_5136,mmu_mi_r_532_5p,mmu_mi_r_541_5p,mmu_mi_r_542_3p,mmu_mi_r_542_5p,mmu_mi_r_543_3p,mmu_mi_r_544_3p,mmu_mi_r_544_5p,mmu_mi_r_551b_5p,mmu_mi_r_5615_3p,mmu_mi_r_5615_5p,mmu_mi_r_5616_5p,mmu_mi_r_5617_5p,mmu_mi_r_5619_3p,mmu_mi_r_5619_5p,mmu_mi_r_5620_3p,mmu_mi_r_5623_5p,mmu_mi_r_5625_3p,mmu_mi_r_5626_3p,mmu_mi_r_5627_3p,mmu_mi_r_5709_3p,mmu_mi_r_5709_5p,mmu_mi_r_5710,mmu_mi_r_582_5p,mmu_mi_r_592_3p,mmu_mi_r_598_3p,mmu_mi_r_598_5p,mmu_mi_r_599,mmu_mi_r_6237,mmu_mi_r_6241,mmu_mi_r_6335,mmu_mi_r_6337,mmu_mi_r_6340,mmu_mi_r_6342,mmu_mi_r_6343,mmu_mi_r_6344,mmu_mi_r_6347,mmu_mi_r_6349,mmu_mi_r_6355,mmu_mi_r_6356,mmu_mi_r_6357,mmu_mi_r_6358,mmu_mi_r_6365,mmu_mi_r_6367,mmu_mi_r_6368,mmu_mi_r_6369,mmu_mi_r_6371,mmu_mi_r_6372,mmu_mi_r_6373,mmu_mi_r_6374,mmu_mi_r_6376,mmu_mi_r_6382,mmu_mi_r_6383,mmu_mi_r_6384,mmu_mi_r_6391,mmu_mi_r_6392_3p,mmu_mi_r_6392_5p,mmu_mi_r_6396,mmu_mi_r_6398,mmu_mi_r_6400,mmu_mi_r_6402,mmu_mi_r_6404,mmu_mi_r_6411,mmu_mi_r_6412,mmu_mi_r_6414,mmu_mi_r_6416_3p,mmu_mi_r_6416_5p,mmu_mi_r_6419,mmu_mi_r_6516_5p,mmu_mi_r_653_5p,mmu_mi_r_6546_5p,mmu_mi_r_664_3p,mmu_mi_r_664_5p,mmu_mi_r_665_3p,mmu_mi_r_665_5p,mmu_mi_r_666_5p,mmu_mi_r_669b_5p,mmu_mi_r_669c_3p,mmu_mi_r_669f_3p,mmu_mi_r_669f_5p,mmu_mi_r_669i,mmu_mi_r_669k_3p,mmu_mi_r_669n,mmu_mi_r_670_3p,mmu_mi_r_6715_5p,mmu_mi_r_672_3p,mmu_mi_r_675_3p,mmu_mi_r_675_5p,mmu_mi_r_676_3p,mmu_mi_r_6769b_3p,mmu_mi_r_6769b_5p,mmu_mi_r_677_3p,mmu_mi_r_682,mmu_mi_r_684,mmu_mi_r_686,mmu_mi_r_6896_3p,mmu_mi_r_6897_3p,mmu_mi_r_6897_5p,mmu_mi_r_6898_3p,mmu_mi_r_6898_5p,mmu_mi_r_6899_5p,mmu_mi_r_690,mmu_mi_r_6900_5p,mmu_mi_r_6902_3p,mmu_mi_r_6902_5p,mmu_mi_r_6903_5p,mmu_mi_r_6905_3p,mmu_mi_r_6907_5p,mmu_mi_r_6908_3p,mmu_mi_r_6908_5p,mmu_mi_r_6910_5p,mmu_mi_r_6913_5p,mmu_mi_r_6914_3p,mmu_mi_r_6914_5p,mmu_mi_r_6915_3p,mmu_mi_r_6917_3p,mmu_mi_r_6917_5p,mmu_mi_r_6918_3p,mmu_mi_r_6919_5p,mmu_mi_r_6921_3p,mmu_mi_r_6922_3p,mmu_mi_r_6922_5p,mmu_mi_r_6923_3p,mmu_mi_r_6923_5p,mmu_mi_r_6924_3p,mmu_mi_r_6924_5p,mmu_mi_r_6925_5p,mmu_mi_r_6928_3p,mmu_mi_r_6929_3p,mmu_mi_r_6931_5p,mmu_mi_r_6932_3p,mmu_mi_r_6932_5p,mmu_mi_r_6933_3p,mmu_mi_r_6935_5p,mmu_mi_r_6936_3p,mmu_mi_r_6937_5p,mmu_mi_r_6938_3p,mmu_mi_r_6939_3p,mmu_mi_r_6939_5p,mmu_mi_r_694,mmu_mi_r_6940_3p,mmu_mi_r_6940_5p,mmu_mi_r_6942_3p,mmu_mi_r_6942_5p,mmu_mi_r_6943_3p,mmu_mi_r_6943_5p,mmu_mi_r_6947_3p,mmu_mi_r_6947_5p,mmu_mi_r_6948_3p,mmu_mi_r_6948_5p,mmu_mi_r_6949_3p,mmu_mi_r_6950_3p,mmu_mi_r_6950_5p,mmu_mi_r_6952_3p,mmu_mi_r_6952_5p,mmu_mi_r_6954_3p,mmu_mi_r_6955_5p,mmu_mi_r_6956_3p,mmu_mi_r_6956_5p,mmu_mi_r_6957_3p,mmu_mi_r_6958_3p,mmu_mi_r_6958_5p,mmu_mi_r_6960_5p,mmu_mi_r_6962_5p,mmu_mi_r_6964_3p,mmu_mi_r_6965_3p,mmu_mi_r_6965_5p,mmu_mi_r_6966_5p,mmu_mi_r_6967_5p,mmu_mi_r_6969_5p,mmu_mi_r_697,mmu_mi_r_6973b_3p,mmu_mi_r_6975_5p,mmu_mi_r_6978_5p,mmu_mi_r_6979_3p,mmu_mi_r_698_5p,mmu_mi_r_6980_5p,mmu_mi_r_6981_5p,mmu_mi_r_6982_3p,mmu_mi_r_6982_5p,mmu_mi_r_6985_3p,mmu_mi_r_6986_3p,mmu_mi_r_6986_5p,mmu_mi_r_6988_3p,mmu_mi_r_6988_5p,mmu_mi_r_6989_3p,mmu_mi_r_6990_5p,mmu_mi_r_6992_3p,mmu_mi_r_6993_5p,mmu_mi_r_6995_3p,mmu_mi_r_6995_5p,mmu_mi_r_6996_3p,mmu_mi_r_6997_5p,mmu_mi_r_6998_5p,mmu_mi_r_700_3p,mmu_mi_r_7003_5p,mmu_mi_r_7005_3p,mmu_mi_r_7007_5p,mmu_mi_r_7008_5p,mmu_mi_r_7009_3p,mmu_mi_r_701_5p,mmu_mi_r_7010_3p,mmu_mi_r_7010_5p,mmu_mi_r_7011_5p,mmu_mi_r_7013_5p,mmu_mi_r_7014_5p,mmu_mi_r_7015_3p,mmu_mi_r_7015_5p,mmu_mi_r_7016_5p,mmu_mi_r_7017_5p,mmu_mi_r_7018_3p,mmu_mi_r_702_3p,mmu_mi_r_7020_5p,mmu_mi_r_7021_5p,mmu_mi_r_7022_5p,mmu_mi_r_7025_3p,mmu_mi_r_7028_3p,mmu_mi_r_7028_5p,mmu_mi_r_7029_5p,mmu_mi_r_7030_3p,mmu_mi_r_7030_5p,mmu_mi_r_7032_5p,mmu_mi_r_7033_3p,mmu_mi_r_7033_5p,mmu_mi_r_7034_5p,mmu_mi_r_7035_3p,mmu_mi_r_7035_5p,mmu_mi_r_7036a_3p,mmu_mi_r_7036b_5p,mmu_mi_r_7037_5p,mmu_mi_r_7038_5p,mmu_mi_r_7042_3p,mmu_mi_r_7044_3p,mmu_mi_r_7045_5p,mmu_mi_r_7047_3p,mmu_mi_r_7047_5p,mmu_mi_r_7048_3p,mmu_mi_r_7049_3p,mmu_mi_r_7049_5p,mmu_mi_r_7050_5p,mmu_mi_r_7052_5p,mmu _mi_r_7053_5p,mmu_mi_r_7054_3p,mmu_mi_r_7056_5p,mmu_mi_r_7057_3p,mmu_mi_r_7058_3p,mmu_mi_r_7058_5p,mmu_mi_r_7059_3p,mmu_mi_r_7059_5p,mmu_mi_r_7060_3p,mmu_mi_r_7061_3p,mmu_mi_r_7062_5p,mmu_mi_r_7063_3p,mmu_mi_r_7063_5p,mmu_mi_r_7064_3p,mmu_mi_r_7065_3p,mmu_mi_r_7066_3p,mmu_mi_r_7066_5p,mmu_mi_r_7067_3p,mmu_mi_r_7069_3p,mmu_mi_r_707,mmu_mi_r_7070_3p,mmu_mi_r_7070_5p,mmu_mi_r_7071_5p,mmu_mi_r_7072_5p,mmu_mi_r_7074_3p,mmu_mi_r_7074_5p,mmu_mi_r_7075_3p,mmu_mi_r_7077_3p,mmu_mi_r_7078_5p,mmu_mi_r_7079_3p,mmu_mi_r_708_5p,mmu_mi_r_7081_3p,mmu_mi_r_7082_3p,mmu_mi_r_7082_5p,mmu_mi_r_7083_3p,mmu_mi_r_7083_5p,mmu_mi_r_7084_3p,mmu_mi_r_7084_5p,mmu_mi_r_7085_5p,mmu_mi_r_7086_3p,mmu_mi_r_7089_3p,mmu_mi_r_7090_3p,mmu_mi_r_7092_5p,mmu_mi_r_7093_3p,mmu_mi_r_7094_1_5p,mmu_mi_r_7094_3p,mmu_mi_r_711,mmu_mi_r_7116_5p,mmu_mi_r_7117_3p,mmu_mi_r_7117_5p,mmu_mi_r_7119_3p,mmu_mi_r_712_3p,mmu_mi_r_712_5p,mmu_mi_r_718,mmu_mi_r_721,mmu_mi_r_7210_5p,mmu_mi_r_7211_3p,mmu_mi_r_7215_3p,mmu_mi_r_7215_5p,mmu_mi_r_7216_3p,mmu_mi_r_7220_5p,mmu_mi_r_7222_5p,mmu_mi_r_7224_5p,mmu_mi_r_7225_5p,mmu_mi_r_7226_3p,mmu_mi_r_7227_3p,mmu_mi_r_7227_5p,mmu_mi_r_7228_5p,mmu_mi_r_7229_3p,mmu_mi_r_7230_3p,mmu_mi_r_7231_5p,mmu_mi_r_7233_3p,mmu_mi_r_7234_5p,mmu_mi_r_7235_3p,mmu_mi_r_7236_3p,mmu_mi_r_7236_5p,mmu_mi_r_7237_3p,mmu_mi_r_7237_5p,mmu_mi_r_7238_3p,mmu_mi_r_7238_5p,mmu_mi_r_7239_5p,mmu_mi_r_7241_3p,mmu_mi_r_7241_5p,mmu_mi_r_7242_3p,mmu_mi_r_7242_5p,mmu_mi_r_7243_5p,mmu_mi_r_741_3p,mmu_mi_r_742_3p,mmu_mi_r_742_5p,mmu_mi_r_743b_3p,mmu_mi_r_743b_5p,mmu_mi_r_758_5p,mmu_mi_r_759,mmu_mi_r_760_3p,mmu_mi_r_760_5p,mmu_mi_r_764_3p,mmu_mi_r_764_5p,mmu_mi_r_7646_3p,mmu_mi_r_7646_5p,mmu_mi_r_7649_5p,mmu_mi_r_7650_5p,mmu_mi_r_7651_5p,mmu_mi_r_7652_3p,mmu_mi_r_7652_5p,mmu_mi_r_7653_5p,mmu_mi_r_7654_3p,mmu_mi_r_7654_5p,mmu_mi_r_7655_3p,mmu_mi_r_7656_5p,mmu_mi_r_7657_3p,mmu_mi_r_7659_3p,mmu_mi_r_7659_5p,mmu_mi_r_7660_3p,mmu_mi_r_7661_3p,mmu_mi_r_7661_5p,mmu_mi_r_7662_3p,mmu_mi_r_7662_5p,mmu_mi_r_7663_5p,mmu_mi_r_7664_3p,mmu_mi_r_7665_3p,mmu_mi_r_7665_5p,mmu_mi_r_7667_5p,mmu_mi_r_7670_3p,mmu_mi_r_7672_3p,mmu_mi_r_7672_5p,mmu_mi_r_7673_5p,mmu_mi_r_7674_5p,mmu_mi_r_7675_3p,mmu_mi_r_7675_5p,mmu_mi_r_7676_5p,mmu_mi_r_7677_3p,mmu_mi_r_7677_5p,mmu_mi_r_7678_5p,mmu_mi_r_7679_5p,mmu_mi_r_7681_3p,mmu_mi_r_7683_5p,mmu_mi_r_7687_5p,mmu_mi_r_770_3p,mmu_mi_r_7a_1_3p,mmu_mi_r_7a_2_3p,mmu_mi_r_7a_5p,mmu_mi_r_802_3p,mmu_mi_r_804,mmu_mi_r_8090,mmu_mi_r_8093,mmu_mi_r_8094,mmu_mi_r_8100,mmu_mi_r_8106,mmu_mi_r_8107,mmu_mi_r_8108,mmu_mi_r_8111,mmu_mi_r_8115,mmu_mi_r_8116,mmu_mi_r_8117,mmu_mi_r_8119,mmu_mi_r_8120,mmu_mi_r_871_5p,mmu_mi_r_872_3p,mmu_mi_r_873a_3p,mmu_mi_r_873a_5p,mmu_mi_r_873b,mmu_mi_r_877_5p,mmu_mi_r_879_3p,mmu_mi_r_879_5p,mmu_mi_r_880_3p,mmu_mi_r_881_3p,mmu_mi_r_882,mmu_mi_r_883a_5p,mmu_mi_r_883b_5p,mmu_mi_r_9_5p,mmu_mi_r_92b_3p,mmu_mi_r_92b_5p,mmu_mi_r_93_5p,mmu_mi_r_96_5p,mmu_mi_r_9768_3p,mmu_mi_r_9768_5p,mmu_mi_r_9769_3p,mmu_mi_r_99a_3p,mmu_pi_r_000273_gb_dq540188_mus_musculus_9_122531596_122531620_plus,mmu_pi_r_000366_gb_dq540412_mus_musculus_17_25602713_25602739_plus,mmu_pi_r_000578_gb_dq540853_mus_musculus_17_39456112_39456137_plus,mmu_pi_r_000620_gb_dq540981_mus_musculus_18_54824345_54824374_minus,mmu_pi_r_000620_gb_dq540981_mus_musculus_3_5843782_5843811_plus,mmu_pi_r_000622_gb_dq540988_mus_musculus_18_54824707_54824734_minus,mmu_pi_r_000622_gb_dq540988_mus_musculus_2_5296560_5296587_minus,mmu_pi_r_000622_gb_dq540988_mus_musculus_3_5843428_5843455_plus,mmu_pi_r_000639_gb_dq541113_mus_musculus_17_39455268_39455298_plus,mmu_pi_r_000691_gb_dq541218_mus_musculus_8_126462165_126462190_minus,mmu_pi_r_000691_gb_dq541218_mus_musculus_8_126484247_126484272_minus,mmu_pi_r_000691_gb_dq541218_mus_musculus_8_126485950_126485975_minus,mmu_pi_r_000691_gb_dq541218_mus_musculus_8_126492702_126492727_minus,mmu_pi_r_000691_gb_dq541218_mus_musculus_8_126494409_126494434_minus,mmu_pi_r_001570_gb_dq543701_mus_musculus_2_5296574_5296603_minus,mmu_pi_r_001662_gb_dq544105_mus_musculus_2_151104333_151104361_minus,mmu_pi_r_002435_gb_dq546549_mus_musculus_17_39454808_39454832_plus,mmu_pi_r_003848_gb_dq551015_mus_musculus_10_18489292_18489321_minus,mmu_pi_r_004374_gb_dq552696_mus_musculus_18_85832427_85832456_minus,mmu_pi_r_004374_gb_dq552696_mus_musculus_9_110126452_110126481_plus,mmu_pi_r_006854_gb_dq562105_mus_musculus_1_157716808_157716836_plus,mmu_pi_r_007829_gb_dq565297_mus_musculus_2_92382336_92382366_plus,mmu_pi_r_010565_gb_dq686723_mus_musculus_16_38309443_38309460_plus,mmu_pi_r_020554_gb_dq701662_mus_musculus_2_92351039_92351069_plus,mmu_pi_r_021097_gb_dq702429_mus_musculus_7_14975966_14975994_minus,mmu_pi_r_022097_gb_dq703900_mus_musculus_17_25602688_25602719_plus,mmu_pi_r_022956_gb_dq705141_mus_musculus_5_115112809_115112837_minus,mmu_pi_r_023189_gb_dq705481_mus_musculus_16_18197850_18197871_minus,mmu_pi_r_024744_gb_dq707762_mus_musculus_17_66099819_66099850_minus,mmu_pi_r_025576_gb_dq708952_mus_musculus_x_6405415_6405436_minus,mmu_pi_r_028252_gb_dq712837_mus_musculus_7_81403433_81403455_plus,mmu_pi_r_028975_gb_dq713872_mus_musculus_x_6405378_6405399_minus,mmu_pi_r_032865_gb_dq719430_mus_musculus_2_116876592_116876614_plus,mmu_pi_r_034208_gb_dq721450_mus_musculus_7_72980839_72980868_plus,mmu_pi_r_035342_gb_dq723105_mus_musculus_15_59084194_59084224_minus,mmu_pi_r_037443_gb_dq726144_mus_musculus_2_92364888_92364918_plus,mmu_pi_r_038323_gb_pi_rna_t34_mus_musculus_13_44880547_44880577_minus,mmu_pi_r_038328_gb_pi_rna_t47_mus_musculus_19_13121850_13121879_plus,mmu_pi_r_039147_gb_pi_rna_2740_mus_musculus_6_87962869_87962889_minus
451.305	x451_305_A	mmu_let_7a_2_3p,mmu_let_7b_3p,mmu_let_7d_5p,mmu_let_7e_5p,mmu_let_7f_1_3p,mmu_let_7i_5p,mmu_let_7j,mmu_let_7k,mmu_mi_r_100_3p,mmu_mi_r_106a_3p,mmu_mi_r_106a_5p,mmu_mi_r_106b_3p,mmu_mi_r_106b_5p,mmu_mi_r_1191a,mmu_mi_r_1195,mmu_mi_r_1198_5p,mmu_mi_r_1199_3p,mmu_mi_r_1224_5p,mmu_mi_r_124_5p,mmu_mi_r_1247_5p,mmu_mi_r_129_1_3p,mmu_mi_r_135a_5p,mmu_mi_r_138_5p,mmu_mi_r_140_3p,mmu_mi_r_144_3p,mmu_mi_r_145b,mmu_mi_r_154_5p,mmu_mi_r_15a_3p,mmu_mi_r_15b_3p,mmu_mi_r_15b_5p,mmu_mi_r_16_2_3p,mmu_mi_r_16_5p,mmu_mi_r_17_3p,mmu_mi_r_17_5p,mmu_mi_r_181b_2_3p,mmu_mi_r_181c_5p,mmu_mi_r_181d_5p,mmu_mi_r_1843a_5p,mmu_mi_r_185_3p,mmu_mi_r_186_5p,mmu_mi_r_1894_5p,mmu_mi_r_1897_5p,mmu_mi_r_1898,mmu_mi_r_1899,mmu_mi_r_18a_3p,mmu_mi_r_18b_5p,mmu_mi_r_1930_3p,mmu_mi_r_1931,mmu_mi_r_1941_5p,mmu_mi_r_1942,mmu_mi_r_1946b,mmu_mi_r_1952,mmu_mi_r_1955_3p,mmu_mi_r_1958,mmu_mi_r_195a_5p,mmu_mi_r_1961,mmu_mi_r_1982_3p,mmu_mi_r_199a_5p,mmu_mi_r_200a_5p,mmu_mi_r_202_3p,mmu_mi_r_204_5p,mmu_mi_r_207,mmu_mi_r_20b_5p,mmu_mi_r_210_5p,mmu_mi_r_211_5p,mmu_mi_r_2137,mmu_mi_r_217_3p,mmu_mi_r_219c_3p,mmu_mi_r_221_3p,mmu_mi_r_222_3p,mmu_mi_r_222_5p,mmu_mi_r_26a_1_3p,mmu_mi_r_26b_3p,mmu_mi_r_27a_5p,mmu_mi_r_28c,mmu_mi_r_290a_3p,mmu_mi_r_290b_3p,mmu_mi_r_291a_3p,mmu_mi_r_293_5p,mmu_mi_r_295_3p,mmu_mi_r_297a_3p_mmu_mi_r_297b_3p_mmu_mi_r_297c_3p,mmu_mi_r_298_3p,mmu_mi_r_299b_3p,mmu_mi_r_29b_1_5p,mmu_mi_r_300_5p,mmu_mi_r_301a_5p,mmu_mi_r_301b_3p,mmu_mi_r_302a_5p,mmu_mi_r_3057_3p,mmu_mi_r_3057_5p,mmu_mi_r_3058_5p,mmu_mi_r_3059_3p,mmu_mi_r_3061_5p,mmu_mi_r_3070_3p,mmu_mi_r_3071_3p,mmu_mi_r_3072_3p,mmu_mi_r_3077_3p,mmu_mi_r_3078_5p,mmu_mi_r_3080_3p,mmu_mi_r_3082_5p,mmu_mi_r_3084_3p,mmu_mi_r_3085_3p,mmu_mi_r_3087_5p,mmu_mi_r_3089_5p,mmu_mi_r_3090_3p,mmu_mi_r_3092_3p,mmu_mi_r_3097_3p,mmu_mi_r_3098_5p,mmu_mi_r_30f.,mmu_mi_r_3100_3p,mmu_mi_r_3102_5p,mmu_mi_r_3105_3p,mmu_mi_r_3105_5p,mmu_mi_r_3109_5p,mmu_mi_r_324_3p,mmu_mi_r_324_5p,mmu_mi_r_325_5p,mmu_mi_r_328_3p,mmu_mi_r_331_5p,mmu_mi_r_344_3p,mmu_mi_r_344d_3_5p,mmu_mi_r_344f_3p,mmu_mi_r_344f_5p,mmu_mi_r_344i,mmu_mi_r_3475_3p,mmu_mi_r_3475_5p,mmu_mi_r_34a_3p,mmu_mi_r_34c_3p,mmu_mi_r_3544_3p,mmu_mi_r_3547_3p,mmu_mi_r_3552,mmu_mi_r_3572_3p,mmu_mi_r_361_3p,mmu_mi_r_376c_5p,mmu_mi_r_379_5p,mmu_mi_r_381_3p,mmu_mi_r_382_3p,mmu_mi_r_3964,mmu_mi_r_3966,mmu_mi_r_3969,mmu_mi_r_3970,mmu_mi_r_421_3p,mmu_mi_r_423_5p,mmu_mi_r_449a_3p,mmu_mi_r_449a_5p,mmu_mi_r_451a,mmu_mi_r_453,mmu_mi_r_465d_3p,mmu_mi_r_466a_3p_mmu_mi_r_466e_3p,mmu_mi_r_466k,mmu_mi_r_466l_3p,mmu_mi_r_467b_3p,mmu_mi_r_485_5p,mmu_mi_r_486a_3p,mmu_mi_r_486a_5p,mmu_mi_r_486b_3p,mmu_mi_r_486b_5p,mmu_mi_r_491_5p,mmu_mi_r_495_5p,mmu_mi_r_5100,mmu_mi_r_5106,mmu_mi_r_5120,mmu_mi_r_5130,mmu_mi_r_5131,mmu_mi_r_541_5p,mmu_mi_r_5617_3p,mmu_mi_r_5624_3p,mmu_mi_r_5709_3p,mmu_mi_r_590_3p,mmu_mi_r_598_5p,mmu_mi_r_6337,mmu_mi_r_6340,mmu_mi_r_6344,mmu_mi_r_6345,mmu_mi_r_6349,mmu_mi_r_6351,mmu_mi_r_6358,mmu_mi_r_6360,mmu_mi_r_6364,mmu_mi_r_6365,mmu_mi_r_6369,mmu_mi_r_6371,mmu_mi_r_6386,mmu_mi_r_6392_5p,mmu_mi_r_6396,mmu_mi_r_6398,mmu_mi_r_6403,mmu_mi_r_6404,mmu_mi_r_6408,mmu_mi_r_6409,mmu_mi_r_6411,mmu_mi_r_6412,mmu_mi_r_6414,mmu_mi_r_6415,mmu_mi_r_6419,mmu_mi_r_652_3p,mmu_mi_r_6537_3p,mmu_mi_r_668_3p,mmu_mi_r_669c_3p,mmu_mi_r_669e_5p,mmu_mi_r_669g,mmu_mi_r_669h_3p,mmu_mi_r_672_3p,mmu_mi_r_673_5p,mmu_mi_r_674_5p,mmu_mi_r_675_5p,mmu_mi_r_676_3p,mmu_mi_r_687,mmu_mi_r_6905_3p,mmu_mi_r_6908_5p,mmu_mi_r_6910_3p,mmu_mi_r_6914_5p,mmu_mi_r_6916_3p,mmu_mi_r_6917_3p,mmu_mi_r_6919_3p,mmu_mi_r_6921_3p,mmu_mi_r_6925_3p,mmu_mi_r_6925_5p,mmu_mi_r_6928_5p,mmu_mi_r_6930_3p,mmu_mi_r_6933_3p,mmu_mi_r_6934_3p,mmu_mi_r_6937_3p,mmu_mi_r_6941_5p,mmu_mi_r_6943_5p,mmu_mi_r_6952_3p,mmu_mi_r_6954_3p,mmu_mi_r_6955_3p,mmu_mi_r_6960_3p,mmu_mi_r_6962_3p,mmu_mi_r_6962_5p,mmu_mi_r_6970_5p,mmu_mi_r_6979_3p,mmu_mi_r_698_5p,mmu_mi_r_6982_5p,mmu_mi_r_6984_5p,mmu_mi_r_6986_3p,mmu_mi_r_6994_3p,mmu_mi_r_7000_5p,mmu_mi_r_7005_5p,mmu_mi_r_7018_3p,mmu_mi_r_7021_5p,mmu_mi_r_7023_3p,mmu_mi_r_7025_3p,mmu_mi_r_7033_3p,mmu_mi_r_7034_3p,mmu_mi_r_7035_3p,mmu_mi_r_7036a_5p,mmu_mi_r_7038_5p,mmu_mi_r_7051_5p,mmu_mi_r_7054_5p,mmu_mi_r_7057_5p,mmu_mi_r_7060_5p,mmu_mi_r_7065_3p,mmu_mi_r_7067_3p,mmu_mi_r_7070_3p,mmu_mi_r_7073_5p,mmu_mi_r_7074_3p,mmu_mi_r_7075_5p,mmu_mi_r_7076_5p,mmu_mi_r_7077_3p,mmu_mi_r_7079_5p,mmu_mi_r_708_3p,mmu_mi_r_7091_5p,mmu_mi_r_7094_3p,mmu_mi_r_7117_5p,mmu_mi_r_712_5p,mmu_mi_r_717,mmu_mi_r_7218_3p,mmu_mi_r_7221_3p,mmu_mi_r_7223_5p,mmu_mi_r_7237_5p,mmu_mi_r_7239_5p,mmu_mi_r_760_3p,mmu_mi_r_7651_3p,mmu_mi_r_7652_3p,mmu_mi_r_7655_3p,mmu_mi_r_7657_5p,mmu_mi_r_7658_5p,mmu_mi_r_7663_5p,mmu_mi_r_7666_5p,mmu_mi_r_7676_3p,mmu_mi_r_7677_3p,mmu_mi_r_7678_3p,mmu_mi_r_7680_5p,mmu_mi_r_7687_5p,mmu_mi_r_7688_5p,mmu_mi_r_770_3p,mmu_mi_r_8092,mmu_mi_r_8106,mmu_mi_r_8107,mmu_mi_r_8108,mmu_mi_r_8110,mmu_mi_r_8119,mmu_mi_r_871_3p,mmu_mi_r_871_5p,mmu_mi_r_881_3p,mmu_mi_r_883a_3p,mmu_mi_r_92a_3p,mmu_mi_r_92b_3p,mmu_mi_r_93_3p,mmu_mi_r_93_5p,mmu_mi_r_9768_5p,mmu_mi_r_98_5p,mmu_pi_r_000619_gb_dq540976_mus_musculus_17_39454691_39454717_plus,mmu_pi_r_001570_gb_dq543701_mus_musculus_3_5843412_5843441_plus,mmu_pi_r_002728_gb_dq547492_mus_musculus_2_150846558_150846586_plus,mmu_pi_r_023189_gb_dq705481_mus_musculus_16_18197850_18197871_minus,mmu_pi_r_023366_gb_dq705744_mus_musculus_7_73687678_73687707_minus,mmu_pi_r_025576_gb_dq708952_mus_musculus_x_6405415_6405436_minus,mmu_pi_r_028975_gb_dq713872_mus_musculus_x_6405378_6405399_minus,mmu_pi_r_032865_gb_dq719430_mus_musculus_2_116876592_116876614_plus,mmu_pi_r_038328_gb_pi_rna_t47_mus_musculus_17_39455055_39455084_plus,mmu_pi_r_038328_gb_pi_rna_t47_mus_musculus_19_13121850_13121879_plus,mmu_pi_r_038649_gb_pi_rna_1701_mus_musculus_17_27070232_27070255_plus
493.165	x493_165_A	mmu_let_7g_3p,mmu_mi_r_103_2_5p,mmu_mi_r_106a_3p,mmu_mi_r_10a_3p,mmu_mi_r_10b_5p,mmu_mi_r_1193_5p,mmu_mi_r_1194,mmu_mi_r_1197_5p,mmu_mi_r_122_3p,mmu_mi_r_122_5p,mmu_mi_r_124_3p,mmu_mi_r_1258_5p,mmu_mi_r_129_1_3p,mmu_mi_r_129_5p,mmu_mi_r_137_5p,mmu_mi_r_138_1_3p,mmu_mi_r_139_3p,mmu_mi_r_146a_3p,mmu_mi_r_146a_5p,mmu_mi_r_150_3p,mmu_mi_r_153_5p,mmu_mi_r_154_5p,mmu_mi_r_155_5p,mmu_mi_r_182_5p,mmu_mi_r_183_5p,mmu_mi_r_1843a_3p,mmu_mi_r_1894_5p,mmu_mi_r_1898,mmu_mi_r_1906,mmu_mi_r_190a_5p,mmu_mi_r_1912_5p,mmu_mi_r_192_3p,mmu_mi_r_192_5p,mmu_mi_r_193a_3p,mmu_mi_r_194_1_3p,mmu_mi_r_194_5p,mmu_mi_r_1941_3p,mmu_mi_r_1941_5p,mmu_mi_r_1943_5p,mmu_mi_r_1952,mmu_mi_r_1966_5p,mmu_mi_r_200a_3p,mmu_mi_r_200b_3p,mmu_mi_r_200b_5p,mmu_mi_r_203_3p,mmu_mi_r_205_5p,mmu_mi_r_20a_3p,mmu_mi_r_210_5p,mmu_mi_r_211_3p,mmu_mi_r_212_3p,mmu_mi_r_212_5p,mmu_mi_r_2136,mmu_mi_r_215_3p,mmu_mi_r_215_5p,mmu_mi_r_217_5p,mmu_mi_r_219c_3p,mmu_mi_r_219c_5p,mmu_mi_r_24_1_5p,mmu_mi_r_26a_1_3p,mmu_mi_r_28a_3p,mmu_mi_r_28c,mmu_mi_r_290a_3p,mmu_mi_r_292a_5p,mmu_mi_r_29c_3p,mmu_mi_r_300_5p,mmu_mi_r_301a_5p,mmu_mi_r_302a_3p,mmu_mi_r_3069_5p,mmu_mi_r_3070_5p,mmu_mi_r_3074_2_3p,mmu_mi_r_3076_3p,mmu_mi_r_3078_3p,mmu_mi_r_3081_5p,mmu_mi_r_3082_3p,mmu_mi_r_3086_3p,mmu_mi_r_3088_5p,mmu_mi_r_3089_3p,mmu_mi_r_3089_5p,mmu_mi_r_3093_3p,mmu_mi_r_3105_5p,mmu_mi_r_331_3p,mmu_mi_r_338_3p,mmu_mi_r_341_5p,mmu_mi_r_3473c,mmu_mi_r_34a_3p,mmu_mi_r_34b_3p,mmu_mi_r_34c_3p,mmu_mi_r_3547_3p,mmu_mi_r_3618_3p,mmu_mi_r_3620_3p,mmu_mi_r_367_3p,mmu_mi_r_370_3p,mmu_mi_r_375_3p,mmu_mi_r_376a_3p,mmu_mi_r_3962,mmu_mi_r_421_5p,mmu_mi_r_429_3p,mmu_mi_r_431_3p,mmu_mi_r_452_5p,mmu_mi_r_465d_3p,mmu_mi_r_466l_3p,mmu_mi_r_466m_3p,mmu_mi_r_470_5p,mmu_mi_r_471_5p,mmu_mi_r_496a_3p,mmu_mi_r_503_5p,mmu_mi_r_509_3p,mmu_mi_r_509_5p,mmu_mi_r_5100,mmu_mi_r_511_5p,mmu_mi_r_5120,mmu_mi_r_5124a,mmu_mi_r_5124b,mmu_mi_r_543_3p,mmu_mi_r_5621_5p,mmu_mi_r_5626_3p,mmu_mi_r_5626_5p,mmu_mi_r_582_3p,mmu_mi_r_592_3p,mmu_mi_r_598_3p,mmu_mi_r_6236,mmu_mi_r_6241,mmu_mi_r_6335,mmu_mi_r_6341,mmu_mi_r_6342,mmu_mi_r_6349,mmu_mi_r_6352,mmu_mi_r_6357,mmu_mi_r_6360,mmu_mi_r_6361,mmu_mi_r_6363,mmu_mi_r_6369,mmu_mi_r_6371,mmu_mi_r_6381,mmu_mi_r_6394,mmu_mi_r_6403,mmu_mi_r_6406,mmu_mi_r_6418_3p,mmu_mi_r_6516_5p,mmu_mi_r_6546_5p,mmu_mi_r_667_5p,mmu_mi_r_668_3p,mmu_mi_r_668_5p,mmu_mi_r_669c_5p,mmu_mi_r_669f_5p,mmu_mi_r_669i,mmu_mi_r_669n,mmu_mi_r_670_5p,mmu_mi_r_671_3p,mmu_mi_r_673_5p,mmu_mi_r_6897_3p,mmu_mi_r_6898_5p,mmu_mi_r_6899_3p,mmu_mi_r_6902_5p,mmu_mi_r_6904_3p,mmu_mi_r_6907_5p,mmu_mi_r_691,mmu_mi_r_6910_5p,mmu_mi_r_6916_3p,mmu_mi_r_6918_3p,mmu_mi_r_6918_5p,mmu_mi_r_6919_5p,mmu_mi_r_692,mmu_mi_r_6920_5p,mmu_mi_r_6927_5p,mmu_mi_r_6929_5p,mmu_mi_r_6936_5p,mmu_mi_r_6938_5p,mmu_mi_r_6940_3p,mmu_mi_r_6940_5p,mmu_mi_r_6941_3p,mmu_mi_r_6941_5p,mmu_mi_r_6946_5p,mmu_mi_r_6948_5p,mmu_mi_r_6951_5p,mmu_mi_r_6954_5p,mmu_mi_r_6955_5p,mmu_mi_r_6966_3p,mmu_mi_r_6966_5p,mmu_mi_r_6969_5p,mmu_mi_r_6970_5p,mmu_mi_r_6972_3p,mmu_mi_r_6973b_3p,mmu_mi_r_6973b_5p,mmu_mi_r_6976_3p,mmu_mi_r_6979_5p,mmu_mi_r_6980_5p,mmu_mi_r_6984_3p,mmu_mi_r_6986_5p,mmu_mi_r_6991_3p,mmu_mi_r_700_5p,mmu_mi_r_7000_5p,mmu_mi_r_7002_5p,mmu_mi_r_7006_5p,mmu_mi_r_7007_5p,mmu_mi_r_7016_3p,mmu_mi_r_7019_3p,mmu_mi_r_7021_3p,mmu_mi_r_7021_5p,mmu_mi_r_7024_5p,mmu_mi_r_7030_3p,mmu_mi_r_7050_3p,mmu_mi_r_7051_3p,mmu_mi_r_7058_3p,mmu_mi_r_7059_3p,mmu_mi_r_7060_3p,mmu_mi_r_7061_5p,mmu_mi_r_7062_5p,mmu_mi_r_7064_5p,mmu_mi_r_7065_3p,mmu_mi_r_7069_5p,mmu_mi_r_7076_5p,mmu_mi_r_7077_3p,mmu_mi_r_708_5p,mmu_mi_r_7084_5p,mmu_mi_r_7085_5p,mmu_mi_r_7089_5p,mmu_mi_r_709,mmu_mi_r_7090_3p,mmu_mi_r_7092_5p,mmu_mi_r_7094_3p,mmu_mi_r_7117_5p,mmu_mi_r_7119_3p,mmu_mi_r_7210_5p,mmu_mi_r_7211_3p,mmu_mi_r_7213_5p,mmu_mi_r_7216_5p,mmu_mi_r_7221_3p,mmu_mi_r_7221_5p,mmu_mi_r_7226_5p,mmu_mi_r_7230_3p,mmu_mi_r_7230_5p,mmu_mi_r_7235_5p,mmu_mi_r_7237_5p,mmu_mi_r_758_3p,mmu_mi_r_758_5p,mmu_mi_r_7648_5p,mmu_mi_r_7649_3p,mmu_mi_r_7650_5p,mmu_mi_r_7660_5p,mmu_mi_r_7669_3p,mmu_mi_r_7670_3p,mmu_mi_r_7674_3p,mmu_mi_r_7681_3p,mmu_mi_r_7685_3p,mmu_mi_r_770_3p,mmu_mi_r_802_5p,mmu_mi_r_8090,mmu_mi_r_8091,mmu_mi_r_8094,mmu_mi_r_8100,mmu_mi_r_8118,mmu_mi_r_871_3p,mmu_mi_r_873a_5p,mmu_mi_r_875_3p,mmu_mi_r_876_3p,mmu_mi_r_880_5p,mmu_mi_r_9_3p,mmu_mi_r_92b_5p,mmu_mi_r_96_3p,mmu_mi_r_99a_3p,mmu_pi_r_000620_gb_dq540981_mus_musculus_18_54824345_54824374_minus,mmu_pi_r_000620_gb_dq540981_mus_musculus_3_5843782_5843811_plus,mmu_pi_r_000622_gb_dq540988_mus_musculus_18_54824707_54824734_minus,mmu_pi_r_000622_gb_dq540988_mus_musculus_3_5843428_5843455_plus,mmu_pi_r_001570_gb_dq543701_mus_musculus_3_5843412_5843441_plus,mmu_pi_r_004374_gb_dq552696_mus_musculus_18_85832427_85832456_minus,mmu_pi_r_009321_gb_dq684704_mus_musculus_7_72988380_72988411_plus,mmu_pi_r_010565_gb_dq686723_mus_musculus_16_38309443_38309460_plus,mmu_pi_r_012641_gb_dq689910_mus_musculus_17_27048950_27048977_minus,mmu_pi_r_017289_gb_dq696831_mus_musculus_6_128804704_128804734_plus,mmu_pi_r_028903_gb_dq713755_mus_musculus_1_109786123_109786152_minus,mmu_pi_r_029303_gb_dq714361_mus_musculus_10_18490237_18490267_minus
546.523	x546_523_A	mmu_let_7c_1_3p,mmu_let_7e_3p,mmu_let_7f_2_3p,mmu_let_7g_3p,mmu_mi_r_1187,mmu_mi_r_1190,mmu_mi_r_1191b_3p,mmu_mi_r_1199_5p,mmu_mi_r_124_3p,mmu_mi_r_1251_3p,mmu_mi_r_1258_3p,mmu_mi_r_1264_5p,mmu_mi_r_126b_3p,mmu_mi_r_126b_5p,mmu_mi_r_127_3p,mmu_mi_r_1298_3p,mmu_mi_r_129b_3p,mmu_mi_r_130c,mmu_mi_r_133b_3p,mmu_mi_r_139_3p,mmu_mi_r_146b_3p,mmu_mi_r_146b_5p,mmu_mi_r_148a_5p,mmu_mi_r_152_3p,mmu_mi_r_152_5p,mmu_mi_r_153_3p,mmu_mi_r_1839_3p,mmu_mi_r_187_3p,mmu_mi_r_188_5p,mmu_mi_r_1899,mmu_mi_r_18b_5p,mmu_mi_r_1927,mmu_mi_r_1932,mmu_mi_r_1933_5p,mmu_mi_r_1948_3p,mmu_mi_r_1952,mmu_mi_r_1963,mmu_mi_r_1966_3p,mmu_mi_r_196b_3p,mmu_mi_r_19b_1_5p,mmu_mi_r_1b_5p,mmu_mi_r_211_3p,mmu_mi_r_218_1_3p,mmu_mi_r_219a_2_3p,mmu_mi_r_21a_3p,mmu_mi_r_21c,mmu_mi_r_223_5p,mmu_mi_r_23b_5p,mmu_mi_r_24_2_5p,mmu_mi_r_27a_3p,mmu_mi_r_290a_5p,mmu_mi_r_290b_5p,mmu_mi_r_291b_5p,mmu_mi_r_297b_5p,mmu_mi_r_298_5p,mmu_mi_r_299a_3p,mmu_mi_r_299a_5p,mmu_mi_r_29b_2_5p,mmu_mi_r_302a_5p,mmu_mi_r_302c_5p,mmu_mi_r_3057_5p,mmu_mi_r_3059_5p,mmu_mi_r_3061_3p,mmu_mi_r_3073b_3p,mmu_mi_r_3074_5p,mmu_mi_r_3076_5p,mmu_mi_r_3079_5p,mmu_mi_r_3083_3p,mmu_mi_r_3095_5p,mmu_mi_r_3097_5p,mmu_mi_r_3100_5p,mmu_mi_r_3108_3p,mmu_mi_r_3109_3p,mmu_mi_r_3110_5p,mmu_mi_r_3113_5p,mmu_mi_r_325_5p,mmu_mi_r_328_5p,mmu_mi_r_329_5p,mmu_mi_r_330_3p,mmu_mi_r_335_3p,mmu_mi_r_337_5p,mmu_mi_r_344b_3p,mmu_mi_r_344e_3p,mmu_mi_r_344h_3p,mmu_mi_r_3473a,mmu_mi_r_3473e,mmu_mi_r_3535,mmu_mi_r_3552,mmu_mi_r_3618_5p,mmu_mi_r_367_5p,mmu_mi_r_374c_5p,mmu_mi_r_376c_3p,mmu_mi_r_3965,mmu_mi_r_3967,mmu_mi_r_412_5p,mmu_mi_r_448_3p,mmu_mi_r_449c_5p,mmu_mi_r_450a_2_3p,mmu_mi_r_450b_3p,mmu_mi_r_452_5p,mmu_mi_r_453,mmu_mi_r_463_3p,mmu_mi_r_465b_5p,mmu_mi_r_466d_5p,mmu_mi_r_466i_5p,mmu_mi_r_467b_3p,mmu_mi_r_467b_5p,mmu_mi_r_467d_5p,mmu_mi_r_467f.,mmu_mi_r_483_3p,mmu_mi_r_485_3p,mmu_mi_r_487b_5p,mmu_mi_r_493_3p,mmu_mi_r_495_3p,mmu_mi_r_5099,mmu_mi_r_5118,mmu_mi_r_5126,mmu_mi_r_541_3p,mmu_mi_r_544_3p,mmu_mi_r_544_5p,mmu_mi_r_546,mmu_mi_r_547_5p,mmu_mi_r_5618_3p,mmu_mi_r_5620_5p,mmu_mi_r_5623_5p,mmu_mi_r_5624_3p,mmu_mi_r_5625_5p,mmu_mi_r_5627_5p,mmu_mi_r_5710,mmu_mi_r_592_5p,mmu_mi_r_615_5p,mmu_mi_r_6237,mmu_mi_r_6336,mmu_mi_r_6339,mmu_mi_r_6350,mmu_mi_r_6373,mmu_mi_r_6376,mmu_mi_r_6377,mmu_mi_r_6384,mmu_mi_r_6390,mmu_mi_r_6398,mmu_mi_r_6400,mmu_mi_r_6402,mmu_mi_r_6408,mmu_mi_r_6410,mmu_mi_r_652_5p,mmu_mi_r_653_5p,mmu_mi_r_6537_5p,mmu_mi_r_6538,mmu_mi_r_668_5p,mmu_mi_r_669b_5p,mmu_mi_r_669c_3p,mmu_mi_r_669l_3p,mmu_mi_r_669p_3p,mmu_mi_r_6715_5p,mmu_mi_r_677_5p,mmu_mi_r_679_3p,mmu_mi_r_682,mmu_mi_r_6896_5p,mmu_mi_r_6901_5p,mmu_mi_r_6906_5p,mmu_mi_r_6907_5p,mmu_mi_r_6908_3p,mmu_mi_r_6909_3p,mmu_mi_r_6915_3p,mmu_mi_r_6917_3p,mmu_mi_r_6920_3p,mmu_mi_r_6922_3p,mmu_mi_r_6923_3p,mmu_mi_r_6924_3p,mmu_mi_r_6924_5p,mmu_mi_r_6928_3p,mmu_mi_r_6930_5p,mmu_mi_r_6935_5p,mmu_mi_r_6938_3p,mmu_mi_r_6939_5p,mmu_mi_r_6941_5p,mmu_mi_r_6947_3p,mmu_mi_r_6948_3p,mmu_mi_r_6953_5p,mmu_mi_r_6956_5p,mmu_mi_r_6958_3p,mmu_mi_r_6959_3p,mmu_mi_r_6961_5p,mmu_mi_r_6963_5p,mmu_mi_r_6969_3p,mmu_mi_r_697,mmu_mi_r_6973b_5p,mmu_mi_r_6979_3p,mmu_mi_r_698_3p,mmu_mi_r_6981_5p,mmu_mi_r_6984_5p,mmu_mi_r_6985_5p,mmu_mi_r_6989_3p,mmu_mi_r_6992_5p,mmu_mi_r_6993_5p,mmu_mi_r_6995_3p,mmu_mi_r_6996_3p,mmu_mi_r_7000_3p,mmu_mi_r_7005_3p,mmu_mi_r_701_5p,mmu_mi_r_7011_5p,mmu_mi_r_7018_3p,mmu_mi_r_7029_3p,mmu_mi_r_7029_5p,mmu_mi_r_7031_5p,mmu_mi_r_7035_5p,mmu_mi_r_7036b_3p,mmu_mi_r_7037_3p,mmu_mi_r_7038_5p,mmu_mi_r_7039_3p,mmu_mi_r_7039_5p,mmu_mi_r_7041_3p,mmu_mi_r_7046_5p,mmu_mi_r_7047_3p,mmu_mi_r_7049_3p,mmu_mi_r_7050_3p,mmu_mi_r_7053_3p,mmu_mi_r_7054_3p,mmu_mi_r_7057_5p,mmu_mi_r_7068_5p,mmu_mi_r_7074_5p,mmu_mi_r_7079_3p,mmu_mi_r_708_5p,mmu_mi_r_7091_3p,mmu_mi_r_7093_5p,mmu_mi_r_7116_3p,mmu_mi_r_7117_3p,mmu_mi_r_7119_5p,mmu_mi_r_7211_3p,mmu_mi_r_7213_3p,mmu_mi_r_7218_5p,mmu_mi_r_7220_3p,mmu_mi_r_7222_5p,mmu_mi_r_7224_3p,mmu_mi_r_7238_5p,mmu_mi_r_7240_5p,mmu_mi_r_7241_5p,mmu_mi_r_741_3p,mmu_mi_r_758_3p,mmu_mi_r_761,mmu_mi_r_762,mmu_mi_r_7647_5p,mmu_mi_r_7648_3p,mmu_mi_r_7650_3p,mmu_mi_r_7654_3p,mmu_mi_r_7659_5p,mmu_mi_r_7664_5p,mmu_mi_r_7670_5p,mmu_mi_r_7671_3p,mmu_mi_r_7673_5p,mmu_mi_r_7675_3p,mmu_mi_r_7677_5p,mmu_mi_r_7684_5p,mmu_mi_r_7685_5p,mmu_mi_r_7686_3p,mmu_mi_r_7687_3p,mmu_mi_r_804,mmu_mi_r_8092,mmu_mi_r_8095,mmu_mi_r_8103,mmu_mi_r_8120,mmu_mi_r_873a_3p,mmu_mi_r_878_5p,mmu_mi_r_880_3p,mmu_mi_r_880_5p,mmu_mi_r_883a_5p,mmu_mi_r_92a_2_5p,mmu_mi_r_96_5p,mmu_mi_r_9768_5p,mmu_pi_r_000578_gb_dq540853_mus_musculus_17_39456112_39456137_plus,mmu_pi_r_000622_gb_dq540988_mus_musculus_x_112404287_112404314_minus,mmu_pi_r_000691_gb_dq541218_mus_musculus_8_126462165_126462190_minus,mmu_pi_r_001662_gb_dq544105_mus_musculus_2_151104333_151104361_minus,mmu_pi_r_002435_gb_dq546549_mus_musculus_17_39454808_39454832_plus,mmu_pi_r_003129_gb_dq548834_mus_musculus_17_27053369_27053399_plus,mmu_pi_r_004374_gb_dq552696_mus_musculus_9_110126452_110126481_plus,mmu_pi_r_013859_gb_dq691760_mus_musculus_9_54046065_54046096_minus,mmu_pi_r_020554_gb_dq701662_mus_musculus_2_92351039_92351069_plus,mmu_pi_r_022097_gb_dq703900_mus_musculus_17_25602688_25602719_plus,mmu_pi_r_022097_gb_dq703900_mus_musculus_6_86369527_86369558_plus,mmu_pi_r_022663_gb_dq704740_mus_musculus_17_27083947_27083976_plus,mmu_pi_r_023799_gb_dq706399_mus_musculus_2_151104329_151104359_minus,mmu_pi_r_029416_gb_dq714514_mus_musculus_9_67542046_67542076_plus,mmu_pi_r_032015_gb_dq718174_mus_musculus_2_10427172_10427193_plus,mmu_pi_r_038323_gb_pi_rna_t34_mus_musculus_13_44880547_44880577_minus,mmu_pi_r_038323_gb_pi_rna_t34_mus_musculus_14_18071278_18071308_plus,mmu_pi_r_039147_gb_pi_rna_2740_mus_musculus_9_118523649_118523669_minus
564.533	x564_533_A	mmu_let_7c_1_3p,mmu_let_7e_3p,mmu_let_7f_2_3p,mmu_let_7g_3p,mmu_mi_r_1187,mmu_mi_r_1190,mmu_mi_r_1191b_3p,mmu_mi_r_1199_5p,mmu_mi_r_124_3p,mmu_mi_r_1251_3p,mmu_mi_r_1258_3p,mmu_mi_r_1264_5p,mmu_mi_r_126b_3p,mmu_mi_r_126b_5p,mmu_mi_r_127_3p,mmu_mi_r_1298_3p,mmu_mi_r_129b_3p,mmu_mi_r_130c,mmu_mi_r_133b_3p,mmu_mi_r_139_3p,mmu_mi_r_146b_3p,mmu_mi_r_146b_5p,mmu_mi_r_148a_5p,mmu_mi_r_152_3p,mmu_mi_r_152_5p,mmu_mi_r_153_3p,mmu_mi_r_1839_3p,mmu_mi_r_187_3p,mmu_mi_r_188_5p,mmu_mi_r_1899,mmu_mi_r_18b_5p,mmu_mi_r_1927,mmu_mi_r_1932,mmu_mi_r_1933_5p,mmu_mi_r_1948_3p,mmu_mi_r_1952,mmu_mi_r_1963,mmu_mi_r_1966_3p,mmu_mi_r_196b_3p,mmu_mi_r_19b_1_5p,mmu_mi_r_1b_5p,mmu_mi_r_211_3p,mmu_mi_r_218_1_3p,mmu_mi_r_219a_2_3p,mmu_mi_r_21a_3p,mmu_mi_r_21c,mmu_mi_r_223_5p,mmu_mi_r_23b_5p,mmu_mi_r_24_2_5p,mmu_mi_r_27a_3p,mmu_mi_r_290a_5p,mmu_mi_r_290b_5p,mmu_mi_r_291b_5p,mmu_mi_r_297b_5p,mmu_mi_r_298_5p,mmu_mi_r_299a_3p,mmu_mi_r_299a_5p,mmu_mi_r_29b_2_5p,mmu_mi_r_302a_5p,mmu_mi_r_302c_5p,mmu_mi_r_3057_5p,mmu_mi_r_3059_5p,mmu_mi_r_3061_3p,mmu_mi_r_3073b_3p,mmu_mi_r_3074_5p,mmu_mi_r_3076_5p,mmu_mi_r_3079_5p,mmu_mi_r_3083_3p,mmu_mi_r_3095_5p,mmu_mi_r_3097_5p,mmu_mi_r_3100_5p,mmu_mi_r_3108_3p,mmu_mi_r_3109_3p,mmu_mi_r_3110_5p,mmu_mi_r_3113_5p,mmu_mi_r_325_5p,mmu_mi_r_328_5p,mmu_mi_r_329_5p,mmu_mi_r_330_3p,mmu_mi_r_335_3p,mmu_mi_r_337_5p,mmu_mi_r_344b_3p,mmu_mi_r_344e_3p,mmu_mi_r_344h_3p,mmu_mi_r_3473a,mmu_mi_r_3473e,mmu_mi_r_3535,mmu_mi_r_3552,mmu_mi_r_3618_5p,mmu_mi_r_367_5p,mmu_mi_r_374c_5p,mmu_mi_r_376c_3p,mmu_mi_r_3965,mmu_mi_r_3967,mmu_mi_r_412_5p,mmu_mi_r_448_3p,mmu_mi_r_449c_5p,mmu_mi_r_450a_2_3p,mmu_mi_r_450b_3p,mmu_mi_r_452_5p,mmu_mi_r_453,mmu_mi_r_463_3p,mmu_mi_r_465b_5p,mmu_mi_r_466d_5p,mmu_mi_r_466i_5p,mmu_mi_r_467b_3p,mmu_mi_r_467b_5p,mmu_mi_r_467d_5p,mmu_mi_r_467f.,mmu_mi_r_483_3p,mmu_mi_r_485_3p,mmu_mi_r_487b_5p,mmu_mi_r_493_3p,mmu_mi_r_495_3p,mmu_mi_r_5099,mmu_mi_r_5118,mmu_mi_r_5126,mmu_mi_r_541_3p,mmu_mi_r_544_3p,mmu_mi_r_544_5p,mmu_mi_r_546,mmu_mi_r_547_5p,mmu_mi_r_5618_3p,mmu_mi_r_5620_5p,mmu_mi_r_5623_5p,mmu_mi_r_5624_3p,mmu_mi_r_5625_5p,mmu_mi_r_5627_5p,mmu_mi_r_5710,mmu_mi_r_592_5p,mmu_mi_r_615_5p,mmu_mi_r_6237,mmu_mi_r_6336,mmu_mi_r_6339,mmu_mi_r_6350,mmu_mi_r_6373,mmu_mi_r_6376,mmu_mi_r_6377,mmu_mi_r_6384,mmu_mi_r_6390,mmu_mi_r_6398,mmu_mi_r_6400,mmu_mi_r_6402,mmu_mi_r_6408,mmu_mi_r_6410,mmu_mi_r_652_5p,mmu_mi_r_653_5p,mmu_mi_r_6537_5p,mmu_mi_r_6538,mmu_mi_r_668_5p,mmu_mi_r_669b_5p,mmu_mi_r_669c_3p,mmu_mi_r_669l_3p,mmu_mi_r_669p_3p,mmu_mi_r_6715_5p,mmu_mi_r_677_5p,mmu_mi_r_679_3p,mmu_mi_r_682,mmu_mi_r_6896_5p,mmu_mi_r_6901_5p,mmu_mi_r_6906_5p,mmu_mi_r_6907_5p,mmu_mi_r_6908_3p,mmu_mi_r_6909_3p,mmu_mi_r_6915_3p,mmu_mi_r_6917_3p,mmu_mi_r_6920_3p,mmu_mi_r_6922_3p,mmu_mi_r_6923_3p,mmu_mi_r_6924_3p,mmu_mi_r_6924_5p,mmu_mi_r_6928_3p,mmu_mi_r_6930_5p,mmu_mi_r_6935_5p,mmu_mi_r_6938_3p,mmu_mi_r_6939_5p,mmu_mi_r_6941_5p,mmu_mi_r_6947_3p,mmu_mi_r_6948_3p,mmu_mi_r_6953_5p,mmu_mi_r_6956_5p,mmu_mi_r_6958_3p,mmu_mi_r_6959_3p,mmu_mi_r_6961_5p,mmu_mi_r_6963_5p,mmu_mi_r_6969_3p,mmu_mi_r_697,mmu_mi_r_6973b_5p,mmu_mi_r_6979_3p,mmu_mi_r_698_3p,mmu_mi_r_6981_5p,mmu_mi_r_6984_5p,mmu_mi_r_6985_5p,mmu_mi_r_6989_3p,mmu_mi_r_6992_5p,mmu_mi_r_6993_5p,mmu_mi_r_6995_3p,mmu_mi_r_6996_3p,mmu_mi_r_7000_3p,mmu_mi_r_7005_3p,mmu_mi_r_701_5p,mmu_mi_r_7011_5p,mmu_mi_r_7018_3p,mmu_mi_r_7029_3p,mmu_mi_r_7029_5p,mmu_mi_r_7031_5p,mmu_mi_r_7035_5p,mmu_mi_r_7036b_3p,mmu_mi_r_7037_3p,mmu_mi_r_7038_5p,mmu_mi_r_7039_3p,mmu_mi_r_7039_5p,mmu_mi_r_7041_3p,mmu_mi_r_7046_5p,mmu_mi_r_7047_3p,mmu_mi_r_7049_3p,mmu_mi_r_7050_3p,mmu_mi_r_7053_3p,mmu_mi_r_7054_3p,mmu_mi_r_7057_5p,mmu_mi_r_7068_5p,mmu_mi_r_7074_5p,mmu_mi_r_7079_3p,mmu_mi_r_708_5p,mmu_mi_r_7091_3p,mmu_mi_r_7093_5p,mmu_mi_r_7116_3p,mmu_mi_r_7117_3p,mmu_mi_r_7119_5p,mmu_mi_r_7211_3p,mmu_mi_r_7213_3p,mmu_mi_r_7218_5p,mmu_mi_r_7220_3p,mmu_mi_r_7222_5p,mmu_mi_r_7224_3p,mmu_mi_r_7238_5p,mmu_mi_r_7240_5p,mmu_mi_r_7241_5p,mmu_mi_r_741_3p,mmu_mi_r_758_3p,mmu_mi_r_761,mmu_mi_r_762,mmu_mi_r_7647_5p,mmu_mi_r_7648_3p,mmu_mi_r_7650_3p,mmu_mi_r_7654_3p,mmu_mi_r_7659_5p,mmu_mi_r_7664_5p,mmu_mi_r_7670_5p,mmu_mi_r_7671_3p,mmu_mi_r_7673_5p,mmu_mi_r_7675_3p,mmu_mi_r_7677_5p,mmu_mi_r_7684_5p,mmu_mi_r_7685_5p,mmu_mi_r_7686_3p,mmu_mi_r_7687_3p,mmu_mi_r_804,mmu_mi_r_8092,mmu_mi_r_8095,mmu_mi_r_8103,mmu_mi_r_8120,mmu_mi_r_873a_3p,mmu_mi_r_878_5p,mmu_mi_r_880_3p,mmu_mi_r_880_5p,mmu_mi_r_883a_5p,mmu_mi_r_92a_2_5p,mmu_mi_r_96_5p,mmu_mi_r_9768_5p,mmu_pi_r_000578_gb_dq540853_mus_musculus_17_39456112_39456137_plus,mmu_pi_r_000622_gb_dq540988_mus_musculus_x_112404287_112404314_minus,mmu_pi_r_000691_gb_dq541218_mus_musculus_8_126462165_126462190_minus,mmu_pi_r_001662_gb_dq544105_mus_musculus_2_151104333_151104361_minus,mmu_pi_r_002435_gb_dq546549_mus_musculus_17_39454808_39454832_plus,mmu_pi_r_003129_gb_dq548834_mus_musculus_17_27053369_27053399_plus,mmu_pi_r_004374_gb_dq552696_mus_musculus_9_110126452_110126481_plus,mmu_pi_r_013859_gb_dq691760_mus_musculus_9_54046065_54046096_minus,mmu_pi_r_020554_gb_dq701662_mus_musculus_2_92351039_92351069_plus,mmu_pi_r_022097_gb_dq703900_mus_musculus_17_25602688_25602719_plus,mmu_pi_r_022097_gb_dq703900_mus_musculus_6_86369527_86369558_plus,mmu_pi_r_022663_gb_dq704740_mus_musculus_17_27083947_27083976_plus,mmu_pi_r_023799_gb_dq706399_mus_musculus_2_151104329_151104359_minus,mmu_pi_r_029416_gb_dq714514_mus_musculus_9_67542046_67542076_plus,mmu_pi_r_032015_gb_dq718174_mus_musculus_2_10427172_10427193_plus,mmu_pi_r_038323_gb_pi_rna_t34_mus_musculus_13_44880547_44880577_minus,mmu_pi_r_038323_gb_pi_rna_t34_mus_musculus_14_18071278_18071308_plus,mmu_pi_r_039147_gb_pi_rna_2740_mus_musculus_9_118523649_118523669_minus
581.183	x581_183_A	mmu_let_7e_3p,mmu_let_7f_2_3p,mmu_let_7g_3p,mmu_let_7j,mmu_let_7k,mmu_mi_r_100_3p,mmu_mi_r_100_5p,mmu_mi_r_101a_5p,mmu_mi_r_101b_5p,mmu_mi_r_103_1_5p,mmu_mi_r_103_2_5p,mmu_mi_r_105,mmu_mi_r_106a_5p,mmu_mi_r_107_5p,mmu_mi_r_10a_3p,mmu_mi_r_10b_3p,mmu_mi_r_1187,mmu_mi_r_1188_3p,mmu_mi_r_1188_5p,mmu_mi_r_1193_3p,mmu_mi_r_1198_3p,mmu_mi_r_1199_3p,mmu_mi_r_122_3p,mmu_mi_r_122_5p,mmu_mi_r_1224_3p,mmu_mi_r_1224_5p,mmu_mi_r_1231_3p,mmu_mi_r_124_3p,mmu_mi_r_124_5p,mmu_mi_r_1249_3p,mmu_mi_r_1249_5p,mmu_mi_r_1251_5p,mmu_mi_r_125a_3p,mmu_mi_r_125a_5p,mmu_mi_r_125b_1_3p,mmu_mi_r_125b_2_3p,mmu_mi_r_125b_5p,mmu_mi_r_1264_3p,mmu_mi_r_1264_5p,mmu_mi_r_126a_3p,mmu_mi_r_126a_5p,mmu_mi_r_126b_3p,mmu_mi_r_126b_5p,mmu_mi_r_128_1_5p,mmu_mi_r_128_2_5p,mmu_mi_r_129_1_3p,mmu_mi_r_129_2_3p,mmu_mi_r_129_5p,mmu_mi_r_1291,mmu_mi_r_1298_3p,mmu_mi_r_1298_5p,mmu_mi_r_129b_5p,mmu_mi_r_1306_5p,mmu_mi_r_130a_3p,mmu_mi_r_130a_5p,mmu_mi_r_130c,mmu_mi_r_133a_5p,mmu_mi_r_133b_3p,mmu_mi_r_133b_5p,mmu_mi_r_135a_2_3p,mmu_mi_r_135a_5p,mmu_mi_r_135b_3p,mmu_mi_r_135b_5p,mmu_mi_r_136_3p,mmu_mi_r_138_2_3p,mmu_mi_r_141_5p,mmu_mi_r_143_3p,mmu_mi_r_143_5p,mmu_mi_r_145a_3p,mmu_mi_r_145a_5p,mmu_mi_r_145b,mmu_mi_r_146a_3p,mmu_mi_r_146a_5p,mmu_mi_r_146b_3p,mmu_mi_r_146b_5p,mmu_mi_r_147_3p,mmu_mi_r_147_5p,mmu_mi_r_148a_5p,mmu_mi_r_149_3p,mmu_mi_r_152_3p,mmu_mi_r_154_3p,mmu_mi_r_154_5p,mmu_mi_r_16_2_3p,mmu_mi_r_1668,mmu_mi_r_181a_1_3p,mmu_mi_r_181a_5p,mmu_mi_r_181b_2_3p,mmu_mi_r_181b_5p,mmu_mi_r_183_3p,mmu_mi_r_183_5p,mmu_mi_r_184_3p,mmu_mi_r_1843b_5p,mmu_mi_r_185_3p,mmu_mi_r_186_3p,mmu_mi_r_187_3p,mmu_mi_r_187_5p,mmu_mi_r_188_5p,mmu_mi_r_1893,mmu_mi_r_1895,mmu_mi_r_1898,mmu_mi_r_18b_3p,mmu_mi_r_1901,mmu_mi_r_1903,mmu_mi_r_1906,mmu_mi_r_1907,mmu_mi_r_190a_3p,mmu_mi_r_190a_5p,mmu_mi_r_190b_3p,mmu_mi_r_191_3p,mmu_mi_r_1912_3p,mmu_mi_r_1912_5p,mmu_mi_r_192_3p,mmu_mi_r_192_5p,mmu_mi_r_1929_3p,mmu_mi_r_1929_5p,mmu_mi_r_1930_5p,mmu_mi_r_1931,mmu_mi_r_1932,mmu_mi_r_1933_3p,mmu_mi_r_1933_5p,mmu_mi_r_1934_3p,mmu_mi_r_1934_5p,mmu_mi_r_1938,mmu_mi_r_193a_5p,mmu_mi_r_193b_3p,mmu_mi_r_194_1_3p,mmu_mi_r_194_5p,mmu_mi_r_1940,mmu_mi_r_1941_3p,mmu_mi_r_1941_5p,mmu_mi_r_1943_3p,mmu_mi_r_1945,mmu_mi_r_1946a,mmu_mi_r_1946b,mmu_mi_r_1947_3p,mmu_mi_r_1947_5p,mmu_mi_r_1949,mmu_mi_r_1950,mmu_mi_r_1952,mmu_mi_r_1953,mmu_mi_r_1954,mmu_mi_r_195a_3p,mmu_mi_r_195b,mmu_mi_r_1960,mmu_mi_r_1961,mmu_mi_r_1963,mmu_mi_r_1964_3p,mmu_mi_r_1964_5p,mmu_mi_r_1966_5p,mmu_mi_r_1968_5p,mmu_mi_r_196a_1_3p,mmu_mi_r_196a_2_3p,mmu_mi_r_196a_5p,mmu_mi_r_1970,mmu_mi_r_1971,mmu_mi_r_1981_3p,mmu_mi_r_199a_3p_mmu_mi_r_199b_3p,mmu_mi_r_199a_5p,mmu_mi_r_199b_5p,mmu_mi_r_19a_5p,mmu_mi_r_19b_1_5p,mmu_mi_r_19b_2_5p,mmu_mi_r_1a_1_5p,mmu_mi_r_1a_2_5p,mmu_mi_r_1b_3p,mmu_mi_r_1b_5p,mmu_mi_r_200a_5p,mmu_mi_r_200b_3p,mmu_mi_r_200b_5p,mmu_mi_r_201_3p,mmu_mi_r_201_5p,mmu_mi_r_202_5p,mmu_mi_r_204_5p,mmu_mi_r_205_3p,mmu_mi_r_208a_5p,mmu_mi_r_20a_3p,mmu_mi_r_20b_3p,mmu_mi_r_20b_5p,mmu_mi_r_211_3p,mmu_mi_r_212_3p,mmu_mi_r_212_5p,mmu_mi_r_2136,mmu_mi_r_214_3p,mmu_mi_r_214_5p,mmu_mi_r_215_3p,mmu_mi_r_215_5p,mmu_mi_r_216a_3p,mmu_mi_r_216b_3p,mmu_mi_r_216c_3p,mmu_mi_r_216c_5p,mmu_mi_r_218_2_3p,mmu_mi_r_218_5p,mmu_mi_r_2183,mmu_mi_r_219a_1_3p,mmu_mi_r_219a_2_3p,mmu_mi_r_219c_3p,mmu_mi_r_21a_3p,mmu_mi_r_21c,mmu_mi_r_223_3p,mmu_mi_r_223_5p,mmu_mi_r_224_3p,mmu_mi_r_224_5p,mmu_mi_r_23a_3p,mmu_mi_r_23a_5p,mmu_mi_r_23b_3p,mmu_mi_r_23b_5p,mmu_mi_r_24_2_5p,mmu_mi_r_24_3p,mmu_mi_r_26a_1_3p,mmu_mi_r_27b_5p,mmu_mi_r_28a_3p,mmu_mi_r_28a_5p,mmu_mi_r_28b,mmu_mi_r_291a_3p,mmu_mi_r_292a_5p,mmu_mi_r_293_3p,mmu_mi_r_293_5p,mmu_mi_r_294_3p,mmu_mi_r_297a_3p_mmu_mi_r_297b_3p_mmu_mi_r_297c_3p,mmu_mi_r_297b_5p,mmu_mi_r_299a_5p,mmu_mi_r_299b_3p,mmu_mi_r_299b_5p,mmu_mi_r_29a_3p,mmu_mi_r_29b_1_5p,mmu_mi_r_29b_2_5p,mmu_mi_r_29b_3p,mmu_mi_r_29c_3p,mmu_mi_r_302a_3p,mmu_mi_r_302b_3p,mmu_mi_r_302c_5p,mmu_mi_r_3057_3p,mmu_mi_r_3057_5p,mmu_mi_r_3058_3p,mmu_mi_r_3058_5p,mmu_mi_r_3060_3p,mmu_mi_r_3060_5p,mmu_mi_r_3061_5p,mmu_mi_r_3062_3p,mmu_mi_r_3062_5p,mmu_mi_r_3064_5p,mmu_mi_r_3065_3p,mmu_mi_r_3065_5p,mmu_mi_r_3066_3p,mmu_mi_r_3066_5p,mmu_mi_r_3067_3p,mmu_mi_r_3067_5p,mmu_mi_r_3069_3p,mmu_mi_r_3069_5p,mmu_mi_r_3070_2_3p,mmu_mi_r_3070_3p,mmu_mi_r_3071_5p,mmu_mi_r_3072_5p,mmu_mi_r_3074_1_3p,mmu_mi_r_3074_2_3p,mmu_mi_r_3074_5p,mmu_mi_r_3075_3p,mmu_mi_r_3075_5p,mmu_mi_r_3078_3p,mmu_mi_r_3078_5p,mmu_mi_r_3079_5p,mmu_mi_r_3081_3p,mmu_mi_r_3081_5p,mmu_mi_r_3084_3p,mmu_mi_r_3084_5p,mmu_mi_r_3085_3p,mmu_mi_r_3086_3p,mmu_mi_r_3087_5p,mmu_mi_r_3088_3p,mmu_mi_r_3089_3p,mmu_mi_r_3090_3p,mmu_mi_r_3091_3p,mmu_mi_r_3091_5p,mmu_mi_r_3092_3p,mmu_mi_r_3092_5p,mmu_mi_r_3094_3p,mmu_mi_r_3094_5p,mmu_mi_r_3095_5p,mmu_mi_r_3097_3p,mmu_mi_r_3098_3p,mmu_mi_r_3098_5p,mmu_mi_r_3099_3p,mmu_mi_r_3099_5p,mmu_mi_r_30b_3p,mmu_mi_r_30c_2_3p,mmu_mi_r_30e_3p,mmu_mi_r_30f.,mmu_mi_r_31_3p,mmu_mi_r_3100_3p,mmu_mi_r_3100_5p,mmu_mi_r_3101_5p,mmu_mi_r_3102_3p_2_3p,mmu_mi_r_3102_5p,mmu_mi_r_3102_5p_2_5p,mmu_mi_r_3103_5p,mmu_mi_r_3104_3p,mmu_mi_r_3105_3p,mmu_mi_r_3105_5p,mmu_mi_r_3106_5p,mmu_mi_r_3108_5p,mmu_mi_r_3109_5p,mmu_mi_r_3110_5p,mmu_mi_r_3112_3p,mmu_mi_r_320_5p,mmu_mi_r_322_3p,mmu_mi_r_322_5p,mmu_mi_r_325_3p,mmu_mi_r_327,mmu_mi_r_328_5p,mmu_mi_r_329_5p,mmu_mi_r_33_3p,mmu_mi_r_33_5p,mmu_mi_r_331_5p,mmu_mi_r_335_3p,mmu_mi_r_335_5p,mmu_mi_r_337_3p,mmu_mi_r_338_3p,mmu_mi_r_338_5p,mmu_mi_r_340_3p,mmu_mi_r_343,mmu_mi_r_344_5p,mmu_mi_r_344b_3p,mmu_mi_r_344b_5p,mmu_mi_r_344c_3p,mmu_mi_r_344d_3p,mmu_mi_r_344f_3p,mmu_mi_r_344h_3p,mmu_mi_r_3470b,mmu_mi_r_3471,mmu_mi_r_3472,mmu_mi_r_3473a,mmu_mi_r_3473d,mmu_mi_r_3473f.,mmu_mi_r_3473g,mmu_mi_r_3474,mmu_mi_r_3475_5p,mmu_mi_r_34a_5p,mmu_mi_r_34b_3p,mmu_mi_r_34b_5p,mmu_mi_r_34c_3p,mmu_mi_r_34c_5p,mmu_mi_r_350_5p,mmu_mi_r_351_3p,mmu_mi_r_3535,mmu_mi_r_3544_3p,mmu_mi_r_3544_5p,mmu_mi_r_3547_5p,mmu_mi_r_3569_3p,mmu_mi_r_3572_5p,mmu_mi_r_3620_5p,mmu_mi_r_365_1_5p,mmu_mi_r_365_2_5p,mmu_mi_r_365_3p,mmu_mi_r_369_3p,mmu_mi_r_374b_3p,mmu_mi_r_374c_5p,mmu_mi_r_376a_3p,mmu_mi_r_376b_3p,mmu_mi_r_376b_5p,mmu_mi_r_376c_3p,mmu_mi_r_376c_5p,mmu_mi_r_377_3p,mmu_mi_r_378b,mmu_mi_r_378d,mmu_mi_r_379_3p,mmu_mi_r_379_5p,mmu_mi_r_380_3p,mmu_mi_r_382_3p,mmu_mi_r_383_3p,mmu_mi_r_384_3p,mmu_mi_r_384_5p,mmu_mi_r_3961,mmu_mi_r_3962,mmu_mi_r_3964,mmu_mi_r_3965,mmu_mi_r_3966,mmu_mi_r_3967,mmu_mi_r_3969,mmu_mi_r_3970,mmu_mi_r_3971,mmu_mi_r_409_5p,mmu_mi_r_411_3p,mmu_mi_r_412_5p,mmu_mi_r_421_5p,mmu_mi_r_429_3p,mmu_mi_r_429_5p,mmu_mi_r_431_3p,mmu_mi_r_434_3p,mmu_mi_r_448_3p,mmu_mi_r_448_5p,mmu_mi_r_450a_5p,mmu_mi_r_451b,mmu_mi_r_452_3p,mmu_mi_r_452_5p,mmu_mi_r_455_3p,mmu_mi_r_455_5p,mmu_mi_r_465d_3p,mmu_mi_r_466a_3p_mmu_mi_r_466e_3p,mmu_mi_r_466d_3p,mmu_mi_r_466h_3p,mmu_mi_r_466i_3p,mmu_mi_r_466i_5p,mmu_mi_r_466j,mmu_mi_r_466k,mmu_mi_r_466m_5p_mmu_mi_r_669m_5p,mmu_mi_r_466n_3p,mmu_mi_r_466q,mmu_mi_r_467c_3p,mmu_mi_r_467c_5p,mmu_mi_r_467e_3p,mmu_mi_r_467e_5p,mmu_mi_r_467f.,mmu_mi_r_467g,mmu_mi_r_468_5p,mmu_mi_r_483_5p,mmu_mi_r_485_3p,mmu_mi_r_486b_3p,mmu_mi_r_487b_3p,mmu_mi_r_488_3p,mmu_mi_r_488_5p,mmu_mi_r_489_3p,mmu_mi_r_490_3p,mmu_mi_r_490_5p,mmu_mi_r_491_5p,mmu_mi_r_494_5p,mmu_mi_r_495_3p,mmu_mi_r_495_5p,mmu_mi_r_496a_3p,mmu_mi_r_496a_5p,mmu_mi_r_497a_3p,mmu_mi_r_497b,mmu_mi_r_499_3p,mmu_mi_r_500_5p,mmu_mi_r_501_5p,mmu_mi_r_503_3p,mmu_mi_r_503_5p,mmu_mi_r_504_3p,mmu_mi_r_504_5p,mmu_mi_r_505_3p,mmu_mi_r_5101,mmu_mi_r_5103,mmu_mi_r_5106,mmu_mi_r_5107_3p,mmu_mi_r_511_3p,mmu_mi_r_511_5p,mmu_mi_r_5110,mmu_mi_r_5114,mmu_mi_r_5116,mmu_mi_r_5118,mmu_mi_r_5119,mmu_mi_r_5120,mmu_mi_r_5121,mmu_mi_r_5122,mmu_mi_r_5123,mmu_mi_r_5124b,mmu_mi_r_5125,mmu_mi_r_5128,mmu_mi_r_5129_3p,mmu_mi_r_5129_5p,mmu_mi_r_5130,mmu_mi_r_5132_3p,mmu_mi_r_5134_5p,mmu_mi_r_5135,mmu_mi_r_5136,mmu_mi_r_532_3p,mmu_mi_r_541_3p,mmu_mi_r_541_5p,mmu_mi_r_542_3p,mmu_mi_r_542_5p,mmu_mi_r_543_3p,mmu_mi_r_544_5p,mmu_mi_r_547_3p,mmu_mi_r_551b_5p,mmu_mi_r_5615_3p,mmu_mi_r_5615_5p,mmu_mi_r_5616_3p,mmu_mi_r_5617_3p,mmu_mi_r_5618_5p,mmu_mi_r_5619_5p,mmu_mi_r_5621_5p,mmu_mi_r_5623_5p,mmu_mi_r_5627_3p,mmu_mi_r_5627_5p,mmu_mi_r_568,mmu_mi_r_5709_5p,mmu_mi_r_5710,mmu_mi_r_574_3p,mmu_mi_r_582_5p,mmu_mi_r_590_3p,mmu_mi_r_590_5p,mmu_mi_r_592_3p,mmu_mi_r_592_5p,mmu_mi_r_598_5p,mmu_mi_r_615_3p,mmu_mi_r_615_5p,mmu_mi_r_6237,mmu_mi_r_6238,mmu_mi_r_6241,mmu_mi_r_6336,mmu_mi_r_6339,mmu_mi_r_6340,mmu_mi_r_6341,mmu_mi_r_6342,mmu_mi_r_6343,mmu_mi_r_6348,mmu_mi_r_6351,mmu_mi_r_6352,mmu_mi_r_6353,mmu_mi_r_6355,mmu_mi_r_6356,mmu_mi_r_6357,mmu_mi_r_6359,mmu_mi_r_6362,mmu_mi_r_6364,mmu_mi_r_6365,mmu_mi_r_6367,mmu_mi_r_6370,mmu_mi_r_6372,mmu_mi_r_6378,mmu_mi_r_6380,mmu_mi_r_6382,mmu_mi_r_6383,mmu_mi_r_6384,mmu_mi_r_6387,mmu_mi_r_6389,mmu_mi_r_6390,mmu_mi_r_6392_5p,mmu_mi_r_6394,mmu_mi_r_6395,mmu_mi_r_6396,mmu_mi_r_6397,mmu_mi_r_6398,mmu_mi_r_6400,mmu_mi_r_6401,mmu_mi_r_6404,mmu_mi_r_6407,mmu_mi_r_6408,mmu_mi_r_6410,mmu_mi_r_6411,mmu_mi_r_6414,mmu_mi_r_6417,mmu_mi_r_6418_3p,mmu_mi_r_6418_5p,mmu_mi_r_6419,mmu_mi_r_6516_3p,mmu_mi_r_6516_5p,mmu_mi_r_653_5p,mmu_mi_r_6537_5p,mmu_mi_r_6538,mmu_mi_r_654_5p,mmu_mi_r_6540_3p,mmu_mi_r_6541,mmu_mi_r_6546_3p,mmu_mi_r_6546_5p,mmu_mi_r_664_5p,mmu_mi_r_665_3p,mmu_mi_r_665_5p,mmu_mi_r_666_3p,mmu_mi_r_666_5p,mmu_mi_r_669a_3_3p,mmu_mi_r_669b_5p,mmu_mi_r_669c_3p,mmu_mi_r_669c_5p,mmu_mi_r_669h_5p,mmu_mi_r_669i,mmu_mi_r_669j,mmu_mi_r_669p_3p,mmu_mi_r_670_5p,mmu_mi_r_6715_5p,mmu_mi_r_672_3p,mmu_mi_r_673_3p,mmu_mi_r_673_5p,mmu_mi_r_675_3p,mmu_mi_r_675_5p,mmu_mi_r_6769b_3p,mmu_mi_r_677_3p,mmu_mi_r_679_5p,mmu_mi_r_681,mmu_mi_r_684,mmu_mi_r_687,mmu_mi_r_688,mmu_mi_r_6896_3p,mmu_mi_r_6897_5p,mmu_mi_r_6898_3p,mmu_mi_r_6899_3p,mmu_mi_r_690,mmu_mi_r_6900_3p,mmu_mi_r_6901_5p,mmu_mi_r_6902_3p,mmu_mi_r_6902_5p,mmu_mi_r_6903_5p,mmu_mi_r_6904_5p,mmu_mi_r_6905_3p,mmu_mi_r_6907_5p,mmu_mi_r_6908_3p,mmu_mi_r_6908_5p,mmu_mi_r_691,mmu_mi_r_6910_5p,mmu_mi_r_6911_3p,mmu_mi_r_6911_5p,mmu_mi_r_6912_3p,mmu_mi_r_6912_5p,mmu_mi_r_6913_5p,mmu_mi_r_6914_3p,mmu_mi_r_6914_5p,mmu_mi_r_6915_3p,mmu_mi_r_6917_3p,mmu_mi_r_6917_5p,mmu_mi_r_6918_3p,mmu_mi_r_6918_5p,mmu_mi_r_6919_3p,mmu_mi_r_692,mmu_mi_r_6920_3p,mmu_mi_r_6920_5p,mmu_mi_r_6921_3p,mmu_mi_r_6921_5p,mmu_mi_r_6922_3p,mmu_mi_r_6922_5p,mmu_mi_r_6923_5p,mmu_mi_r_6925_3p,mmu_mi_r_6925_5p,mmu_mi_r_6926_3p,mmu_mi_r_6926_5p,mmu_mi_r_6929_3p,mmu_mi_r_693_3p,mmu_mi_r_693_5p,mmu_mi_r_6930_5p,mmu_mi_r_6931_5p,mmu_mi_r_6932_3p,mmu_mi_r_693 2_5p,mmu_mi_r_6933_3p,mmu_mi_r_6933_5p,mmu_mi_r_6934_3p,mmu_mi_r_6934_5p,mmu_mi_r_6935_3p,mmu_mi_r_6935_5p,mmu_mi_r_6937_5p,mmu_mi_r_694,mmu_mi_r_6940_3p,mmu_mi_r_6941_3p,mmu_mi_r_6941_5p,mmu_mi_r_6942_3p,mmu_mi_r_6942_5p,mmu_mi_r_6943_5p,mmu_mi_r_6945_5p,mmu_mi_r_6947_3p,mmu_mi_r_6947_5p,mmu_mi_r_6948_5p,mmu_mi_r_6949_3p,mmu_mi_r_6949_5p,mmu_mi_r_695,mmu_mi_r_6951_5p,mmu_mi_r_6952_3p,mmu_mi_r_6952_5p,mmu_mi_r_6953_3p,mmu_mi_r_6953_5p,mmu_mi_r_6955_5p,mmu_mi_r_6956_3p,mmu_mi_r_6957_3p,mmu_mi_r_6958_5p,mmu_mi_r_6959_3p,mmu_mi_r_696,mmu_mi_r_6960_5p,mmu_mi_r_6961_3p,mmu_mi_r_6961_5p,mmu_mi_r_6963_5p,mmu_mi_r_6965_3p,mmu_mi_r_6965_5p,mmu_mi_r_6967_5p,mmu_mi_r_6968_3p,mmu_mi_r_6969_5p,mmu_mi_r_697,mmu_mi_r_6970_3p,mmu_mi_r_6970_5p,mmu_mi_r_6971_3p,mmu_mi_r_6972_5p,mmu_mi_r_6973a_5p,mmu_mi_r_6973b_3p,mmu_mi_r_6973b_5p,mmu_mi_r_6974_3p,mmu_mi_r_6974_5p,mmu_mi_r_6975_3p,mmu_mi_r_6975_5p,mmu_mi_r_6976_3p,mmu_mi_r_6976_5p,mmu_mi_r_6978_5p,mmu_mi_r_6979_5p,mmu_mi_r_698_3p,mmu_mi_r_698_5p,mmu_mi_r_6981_5p,mmu_mi_r_6983_5p,mmu_mi_r_6984_3p,mmu_mi_r_6986_5p,mmu_mi_r_6987_5p,mmu_mi_r_6988_3p,mmu_mi_r_6988_5p,mmu_mi_r_6989_3p,mmu_mi_r_6989_5p,mmu_mi_r_6990_5p,mmu_mi_r_6991_3p,mmu_mi_r_6993_3p,mmu_mi_r_6993_5p,mmu_mi_r_6994_3p,mmu_mi_r_6994_5p,mmu_mi_r_6996_5p,mmu_mi_r_6997_3p,mmu_mi_r_6997_5p,mmu_mi_r_6998_5p,mmu_mi_r_6999_3p,mmu_mi_r_6999_5p,mmu_mi_r_7000_3p,mmu_mi_r_7000_5p,mmu_mi_r_7003_5p,mmu_mi_r_7005_5p,mmu_mi_r_7006_3p,mmu_mi_r_7008_3p,mmu_mi_r_7008_5p,mmu_mi_r_7009_3p,mmu_mi_r_7009_5p,mmu_mi_r_701_3p,mmu_mi_r_701_5p,mmu_mi_r_7010_3p,mmu_mi_r_7010_5p,mmu_mi_r_7011_3p,mmu_mi_r_7012_3p,mmu_mi_r_7012_5p,mmu_mi_r_7013_3p,mmu_mi_r_7013_5p,mmu_mi_r_7014_5p,mmu_mi_r_7015_3p,mmu_mi_r_7015_5p,mmu_mi_r_7016_3p,mmu_mi_r_7016_5p,mmu_mi_r_7017_3p,mmu_mi_r_7018_5p,mmu_mi_r_7019_3p,mmu_mi_r_7019_5p,mmu_mi_r_702_3p,mmu_mi_r_702_5p,mmu_mi_r_7020_5p,mmu_mi_r_7022_5p,mmu_mi_r_7023_3p,mmu_mi_r_7023_5p,mmu_mi_r_7024_5p,mmu_mi_r_7025_3p,mmu_mi_r_7025_5p,mmu_mi_r_7026_5p,mmu_mi_r_7027_3p,mmu_mi_r_7027_5p,mmu_mi_r_7028_3p,mmu_mi_r_7029_3p,mmu_mi_r_7029_5p,mmu_mi_r_703,mmu_mi_r_7032_5p,mmu_mi_r_7033_3p,mmu_mi_r_7033_5p,mmu_mi_r_7034_3p,mmu_mi_r_7034_5p,mmu_mi_r_7035_3p,mmu_mi_r_7036a_3p,mmu_mi_r_7036b_5p,mmu_mi_r_7037_3p,mmu_mi_r_7037_5p,mmu_mi_r_7038_3p,mmu_mi_r_7038_5p,mmu_mi_r_7039_5p,mmu_mi_r_7040_5p,mmu_mi_r_7041_3p,mmu_mi_r_7041_5p,mmu_mi_r_7042_3p,mmu_mi_r_7043_5p,mmu_mi_r_7044_5p,mmu_mi_r_7046_5p,mmu_mi_r_7047_3p,mmu_mi_r_7048_3p,mmu_mi_r_7048_5p,mmu_mi_r_7050_3p,mmu_mi_r_7051_3p,mmu_mi_r_7051_5p,mmu_mi_r_7052_5p,mmu_mi_r_7053_3p,mmu_mi_r_7055_3p,mmu_mi_r_7055_5p,mmu_mi_r_7056_5p,mmu_mi_r_7057_3p,mmu_mi_r_7058_3p,mmu_mi_r_7059_3p,mmu_mi_r_706,mmu_mi_r_7060_3p,mmu_mi_r_7061_5p,mmu_mi_r_7062_5p,mmu_mi_r_7063_3p,mmu_mi_r_7063_5p,mmu_mi_r_7064_3p,mmu_mi_r_7064_5p,mmu_mi_r_7065_3p,mmu_mi_r_7066_3p,mmu_mi_r_7066_5p,mmu_mi_r_7067_3p,mmu_mi_r_7067_5p,mmu_mi_r_7068_5p,mmu_mi_r_7069_3p,mmu_mi_r_707,mmu_mi_r_7070_5p,mmu_mi_r_7073_5p,mmu_mi_r_7074_3p,mmu_mi_r_7074_5p,mmu_mi_r_7075_3p,mmu_mi_r_7076_3p,mmu_mi_r_7077_3p,mmu_mi_r_7078_5p,mmu_mi_r_7079_3p,mmu_mi_r_708_5p,mmu_mi_r_7080_3p,mmu_mi_r_7080_5p,mmu_mi_r_7081_3p,mmu_mi_r_7082_3p,mmu_mi_r_7082_5p,mmu_mi_r_7083_3p,mmu_mi_r_7083_5p,mmu_mi_r_7084_3p,mmu_mi_r_7085_3p,mmu_mi_r_7085_5p,mmu_mi_r_7086_3p,mmu_mi_r_7086_5p,mmu_mi_r_7088_3p,mmu_mi_r_7089_3p,mmu_mi_r_709,mmu_mi_r_7090_3p,mmu_mi_r_7091_3p,mmu_mi_r_7091_5p,mmu_mi_r_7093_3p,mmu_mi_r_7093_5p,mmu_mi_r_7094_1_5p,mmu_mi_r_7094_3p,mmu_mi_r_7094b_2_5p,mmu_mi_r_711,mmu_mi_r_7115_5p,mmu_mi_r_7116_5p,mmu_mi_r_7117_5p,mmu_mi_r_7119_5p,mmu_mi_r_712_3p,mmu_mi_r_714,mmu_mi_r_717,mmu_mi_r_719,mmu_mi_r_721,mmu_mi_r_7211_3p,mmu_mi_r_7212_3p,mmu_mi_r_7212_5p,mmu_mi_r_7214_3p,mmu_mi_r_7214_5p,mmu_mi_r_7215_3p,mmu_mi_r_7216_3p,mmu_mi_r_7216_5p,mmu_mi_r_7217_3p,mmu_mi_r_7219_5p,mmu_mi_r_7220_5p,mmu_mi_r_7222_3p,mmu_mi_r_7222_5p,mmu_mi_r_7223_5p,mmu_mi_r_7224_3p,mmu_mi_r_7225_3p,mmu_mi_r_7226_3p,mmu_mi_r_7227_5p,mmu_mi_r_7228_3p,mmu_mi_r_7229_3p,mmu_mi_r_7230_3p,mmu_mi_r_7230_5p,mmu_mi_r_7231_3p,mmu_mi_r_7233_3p,mmu_mi_r_7234_3p,mmu_mi_r_7235_5p,mmu_mi_r_7236_5p,mmu_mi_r_7238_3p,mmu_mi_r_7238_5p,mmu_mi_r_7239_3p,mmu_mi_r_7239_5p,mmu_mi_r_7241_5p,mmu_mi_r_7242_3p,mmu_mi_r_7243_5p,mmu_mi_r_741_3p,mmu_mi_r_742_3p,mmu_mi_r_743a_3p,mmu_mi_r_743b_3p,mmu_mi_r_744_3p,mmu_mi_r_758_3p,mmu_mi_r_758_5p,mmu_mi_r_759,mmu_mi_r_760_5p,mmu_mi_r_761,mmu_mi_r_762,mmu_mi_r_7646_5p,mmu_mi_r_7647_5p,mmu_mi_r_7648_3p,mmu_mi_r_7648_5p,mmu_mi_r_7649_3p,mmu_mi_r_7649_5p,mmu_mi_r_7650_5p,mmu_mi_r_7651_3p,mmu_mi_r_7653_3p,mmu_mi_r_7653_5p,mmu_mi_r_7654_5p,mmu_mi_r_7655_3p,mmu_mi_r_7655_5p,mmu_mi_r_7656_3p,mmu_mi_r_7657_3p,mmu_mi_r_7658_5p,mmu_mi_r_7659_3p,mmu_mi_r_7660_3p,mmu_mi_r_7661_3p,mmu_mi_r_7662_5p,mmu_mi_r_7664_3p,mmu_mi_r_7664_5p,mmu_mi_r_7665_5p,mmu_mi_r_7666_5p,mmu_mi_r_7667_3p,mmu_mi_r_7667_5p,mmu_mi_r_767,mmu_mi_r_7670_3p,mmu_mi_r_7671_5p,mmu_mi_r_7672_5p,mmu_mi_r_7673_3p,mmu_mi_r_7674_3p,mmu_mi_r_7674_5p,mmu_mi_r_7675_3p,mmu_mi_r_7675_5p,mmu_mi_r_7676_3p,mmu_mi_r_7677_3p,mmu_mi_r_7677_5p,mmu_mi_r_7678_5p,mmu_mi_r_7679_3p,mmu_mi_r_7679_5p,mmu_mi_r_7681_5p,mmu_mi_r_7683_5p,mmu_mi_r_7687_3p,mmu_mi_r_7687_5p,mmu_mi_r_7689_3p,mmu_mi_r_770_3p,mmu_mi_r_7a_2_3p,mmu_mi_r_7b_3p,mmu_mi_r_7b_5p,mmu_mi_r_802_3p,mmu_mi_r_802_5p,mmu_mi_r_8091,mmu_mi_r_8092,mmu_mi_r_8093,mmu_mi_r_8094,mmu_mi_r_8097,mmu_mi_r_8099,mmu_mi_r_8102,mmu_mi_r_8103,mmu_mi_r_8105,mmu_mi_r_8107,mmu_mi_r_8109,mmu_mi_r_8111,mmu_mi_r_8113,mmu_mi_r_8115,mmu_mi_r_8116,mmu_mi_r_8117,mmu_mi_r_8119,mmu_mi_r_871_3p,mmu_mi_r_873a_5p,mmu_mi_r_873b,mmu_mi_r_876_3p,mmu_mi_r_877_3p,mmu_mi_r_877_5p,mmu_mi_r_878_5p,mmu_mi_r_879_3p,mmu_mi_r_879_5p,mmu_mi_r_880_3p,mmu_mi_r_881_3p,mmu_mi_r_881_5p,mmu_mi_r_882,mmu_mi_r_92a_1_5p,mmu_mi_r_92a_2_5p,mmu_mi_r_92b_5p,mmu_mi_r_96_3p,mmu_mi_r_9768_3p,mmu_mi_r_9769_3p,mmu_mi_r_9769_5p,mmu_mi_r_99a_3p,mmu_mi_r_99a_5p,mmu_mi_r_99b_3p,mmu_mi_r_99b_5p,mmu_pi_r_000159_gb_dq539904_mus_musculus_9_118523651_118523678_minus,mmu_pi_r_000219_gb_dq540058_mus_musculus_17_39455665_39455691_plus,mmu_pi_r_000580_gb_dq540862_mus_musculus_11_106317126_106317155_plus,mmu_pi_r_000616_gb_dq540965_mus_musculus_2_5296571_5296602_minus,mmu_pi_r_000616_gb_dq540965_mus_musculus_3_5843413_5843444_plus,mmu_pi_r_000620_gb_dq540981_mus_musculus_18_54824345_54824374_minus,mmu_pi_r_000620_gb_dq540981_mus_musculus_3_5843782_5843811_plus,mmu_pi_r_000622_gb_dq540988_mus_musculus_18_54824707_54824734_minus,mmu_pi_r_000622_gb_dq540988_mus_musculus_2_5296560_5296587_minus,mmu_pi_r_000622_gb_dq540988_mus_musculus_3_5843428_5843455_plus,mmu_pi_r_000691_gb_dq541218_mus_musculus_8_126457090_126457115_minus,mmu_pi_r_000691_gb_dq541218_mus_musculus_8_126462165_126462190_minus,mmu_pi_r_000691_gb_dq541218_mus_musculus_8_126472331_126472356_minus,mmu_pi_r_000691_gb_dq541218_mus_musculus_8_126484247_126484272_minus,mmu_pi_r_000691_gb_dq541218_mus_musculus_8_126485950_126485975_minus,mmu_pi_r_000691_gb_dq541218_mus_musculus_8_126492702_126492727_minus,mmu_pi_r_000691_gb_dq541218_mus_musculus_8_126494409_126494434_minus,mmu_pi_r_000958_gb_dq541851_mus_musculus_17_39456228_39456254_plus,mmu_pi_r_001662_gb_dq544105_mus_musculus_2_151104333_151104361_minus,mmu_pi_r_002770_gb_dq547637_mus_musculus_2_92382387_92382415_plus,mmu_pi_r_003274_gb_dq549318_mus_musculus_17_27047610_27047635_minus,mmu_pi_r_003399_gb_dq549760_mus_musculus_5_108144856_108144884_plus,mmu_pi_r_003848_gb_dq551015_mus_musculus_10_18489292_18489321_minus,mmu_pi_r_004086_gb_dq551625_mus_musculus_2_129969457_129969485_plus,mmu_pi_r_004374_gb_dq552696_mus_musculus_9_110126452_110126481_plus,mmu_pi_r_005109_gb_dq555094_mus_musculus_5_146565258_146565289_plus,mmu_pi_r_007729_gb_dq564913_mus_musculus_2_102630254_102630283_minus,mmu_pi_r_007729_gb_dq564913_mus_musculus_9_123310826_123310855_plus,mmu_pi_r_007829_gb_dq565297_mus_musculus_2_92382336_92382366_plus,mmu_pi_r_016623_gb_dq695859_mus_musculus_9_54023526_54023555_minus,mmu_pi_r_017289_gb_dq696831_mus_musculus_18_60298547_60298577_plus,mmu_pi_r_017405_gb_dq696996_mus_musculus_11_65550994_65551015_minus,mmu_pi_r_018111_gb_dq698026_mus_musculus_14_22942510_22942534_plus,mmu_pi_r_020492_gb_dq701563_mus_musculus_11_108827972_108827997_minus,mmu_pi_r_020692_gb_dq701869_mus_musculus_5_115840031_115840052_plus,mmu_pi_r_022545_gb_dq704578_mus_musculus_9_67495705_67495734_minus,mmu_pi_r_022828_gb_dq704964_mus_musculus_18_67193120_67193149_plus,mmu_pi_r_022956_gb_dq705141_mus_musculus_5_115112809_115112837_minus,mmu_pi_r_023189_gb_dq705481_mus_musculus_16_18197850_18197871_minus,mmu_pi_r_025576_gb_dq708952_mus_musculus_x_6405415_6405436_minus,mmu_pi_r_027673_gb_dq711996_mus_musculus_11_106317081_106317102_plus,mmu_pi_r_028241_gb_dq712821_mus_musculus_x_56741084_56741107_minus,mmu_pi_r_028252_gb_dq712837_mus_musculus_7_81403433_81403455_plus,mmu_pi_r_028975_gb_dq713872_mus_musculus_x_6405378_6405399_minus,mmu_pi_r_030152_gb_dq715561_mus_musculus_18_67167521_67167549_minus,mmu_pi_r_032015_gb_dq718174_mus_musculus_2_10427172_10427193_plus,mmu_pi_r_032974_gb_dq719597_mus_musculus_4_130021751_130021778_plus,mmu_pi_r_034512_gb_dq721887_mus_musculus_14_22936178_22936204_plus,mmu_pi_r_035342_gb_dq723105_mus_musculus_15_59084194_59084224_minus,mmu_pi_r_038322_gb_pi_rna_t33_mus_musculus_6_3151475_3151505_plus,mmu_pi_r_038323_gb_pi_rna_t34_mus_musculus_13_44880547_44880577_minus,mmu_pi_r_038323_gb_pi_rna_t34_mus_musculus_14_18071278_18071308_plus,mmu_pi_r_038328_gb_pi_rna_t47_mus_musculus_17_39455055_39455084_plus,mmu_pi_r_038328_gb_pi_rna_t47_mus_musculus_6_3151091_3151120_plus,mmu_pi_r_038328_gb_pi_rna_t47_mus_musculus_x_23165477_23165506_plus,mmu_pi_r_039147_gb_pi_rna_2740_mus_musculus_6_87962869_87962889_minus,mmu_pi_r_039147_gb_pi_rna_2740_mus_musculus_9_118523649_118523669_minus
581.309	x581_309_A	mmu_let_7a_1_3p_mmu_let_7c_2_3p,mmu_let_7a_2_3p,mmu_mi_r_106a_3p,mmu_mi_r_1191a,mmu_mi_r_1191b_5p,mmu_mi_r_1199_3p,mmu_mi_r_1199_5p,mmu_mi_r_122_3p,mmu_mi_r_1224_5p,mmu_mi_r_124_3p,mmu_mi_r_129_1_3p,mmu_mi_r_1291,mmu_mi_r_130b_5p,mmu_mi_r_134_5p,mmu_mi_r_136_5p,mmu_mi_r_138_2_3p,mmu_mi_r_142a_3p,mmu_mi_r_142a_5p,mmu_mi_r_142b,mmu_mi_r_146a_5p,mmu_mi_r_150_3p,mmu_mi_r_150_5p,mmu_mi_r_155_5p,mmu_mi_r_15a_3p,mmu_mi_r_181a_5p,mmu_mi_r_182_5p,mmu_mi_r_1843a_5p,mmu_mi_r_188_3p,mmu_mi_r_1896,mmu_mi_r_18b_5p,mmu_mi_r_192_3p,mmu_mi_r_1929_3p,mmu_mi_r_1932,mmu_mi_r_1934_3p,mmu_mi_r_1943_5p,mmu_mi_r_1946b,mmu_mi_r_1948_5p,mmu_mi_r_1960,mmu_mi_r_1968_3p,mmu_mi_r_196a_5p,mmu_mi_r_199b_5p,mmu_mi_r_200c_5p,mmu_mi_r_203_5p,mmu_mi_r_204_3p,mmu_mi_r_210_5p,mmu_mi_r_211_3p,mmu_mi_r_2137,mmu_mi_r_218_1_3p,mmu_mi_r_23a_3p,mmu_mi_r_23b_3p,mmu_mi_r_24_3p,mmu_mi_r_2861,mmu_mi_r_290a_5p,mmu_mi_r_290b_5p,mmu_mi_r_292b_3p,mmu_mi_r_296_3p,mmu_mi_r_297a_5p,mmu_mi_r_300_3p,mmu_mi_r_3060_5p,mmu_mi_r_3068_5p,mmu_mi_r_3069_3p,mmu_mi_r_3070_3p,mmu_mi_r_3073b_3p,mmu_mi_r_3079_3p,mmu_mi_r_3080_3p,mmu_mi_r_3090_5p,mmu_mi_r_3091_5p,mmu_mi_r_3099_3p,mmu_mi_r_30c_1_3p,mmu_mi_r_30d_3p,mmu_mi_r_3101_5p,mmu_mi_r_3154,mmu_mi_r_322_3p,mmu_mi_r_322_5p,mmu_mi_r_326_3p,mmu_mi_r_326_5p,mmu_mi_r_342_3p,mmu_mi_r_344f_5p,mmu_mi_r_3470a,mmu_mi_r_3473b,mmu_mi_r_3473g,mmu_mi_r_351_5p,mmu_mi_r_3535,mmu_mi_r_3572_5p,mmu_mi_r_362_3p,mmu_mi_r_3620_5p,mmu_mi_r_367_3p,mmu_mi_r_369_5p,mmu_mi_r_376b_3p,mmu_mi_r_380_5p,mmu_mi_r_383_3p,mmu_mi_r_3966,mmu_mi_r_3968,mmu_mi_r_423_3p,mmu_mi_r_433_3p,mmu_mi_r_449a_5p,mmu_mi_r_449c_5p,mmu_mi_r_450b_5p,mmu_mi_r_463_3p,mmu_mi_r_466b_3p_mmu_mi_r_466c_3p_mmu_mi_r_466p_3p,mmu_mi_r_467d_5p,mmu_mi_r_467e_5p,mmu_mi_r_471_5p,mmu_mi_r_487b_5p,mmu_mi_r_488_5p,mmu_mi_r_489_5p,mmu_mi_r_490_3p,mmu_mi_r_490_5p,mmu_mi_r_497a_3p,mmu_mi_r_497b,mmu_mi_r_500_3p,mmu_mi_r_500_5p,mmu_mi_r_503_3p,mmu_mi_r_5100,mmu_mi_r_5103,mmu_mi_r_5110,mmu_mi_r_5112,mmu_mi_r_5118,mmu_mi_r_5120,mmu_mi_r_5134_3p,mmu_mi_r_542_3p,mmu_mi_r_551b_5p,mmu_mi_r_5621_5p,mmu_mi_r_5622_3p,mmu_mi_r_5624_3p,mmu_mi_r_5624_5p,mmu_mi_r_5627_3p,mmu_mi_r_598_5p,mmu_mi_r_6236,mmu_mi_r_6237,mmu_mi_r_6239,mmu_mi_r_6241,mmu_mi_r_6345,mmu_mi_r_6346,mmu_mi_r_6350,mmu_mi_r_6356,mmu_mi_r_6359,mmu_mi_r_6366,mmu_mi_r_6370,mmu_mi_r_6373,mmu_mi_r_6379,mmu_mi_r_6389,mmu_mi_r_6391,mmu_mi_r_6396,mmu_mi_r_6400,mmu_mi_r_6404,mmu_mi_r_6407,mmu_mi_r_6415,mmu_mi_r_667_5p,mmu_mi_r_669c_5p,mmu_mi_r_669e_3p,mmu_mi_r_669h_5p,mmu_mi_r_671_5p,mmu_mi_r_672_5p,mmu_mi_r_675_5p,mmu_mi_r_676_5p,mmu_mi_r_679_3p,mmu_mi_r_680,mmu_mi_r_6897_5p,mmu_mi_r_6898_3p,mmu_mi_r_6905_5p,mmu_mi_r_6909_5p,mmu_mi_r_6912_3p,mmu_mi_r_6919_3p,mmu_mi_r_6927_3p,mmu_mi_r_6934_3p,mmu_mi_r_6935_3p,mmu_mi_r_6938_3p,mmu_mi_r_6945_3p,mmu_mi_r_695,mmu_mi_r_6951_5p,mmu_mi_r_6952_5p,mmu_mi_r_6955_5p,mmu_mi_r_696,mmu_mi_r_6961_3p,mmu_mi_r_6970_3p,mmu_mi_r_6974_5p,mmu_mi_r_6976_3p,mmu_mi_r_6976_5p,mmu_mi_r_6977_3p,mmu_mi_r_6980_3p,mmu_mi_r_6981_5p,mmu_mi_r_6982_3p,mmu_mi_r_6982_5p,mmu_mi_r_6983_3p,mmu_mi_r_6984_5p,mmu_mi_r_6987_5p,mmu_mi_r_6988_3p,mmu_mi_r_6990_3p,mmu_mi_r_6992_3p,mmu_mi_r_6994_5p,mmu_mi_r_6995_3p,mmu_mi_r_6995_5p,mmu_mi_r_7001_3p,mmu_mi_r_7004_3p,mmu_mi_r_7014_3p,mmu_mi_r_7021_3p,mmu_mi_r_7022_3p,mmu_mi_r_7023_5p,mmu_mi_r_7024_3p,mmu_mi_r_7027_5p,mmu_mi_r_7035_5p,mmu_mi_r_7041_5p,mmu_mi_r_7043_5p,mmu_mi_r_7045_3p,mmu_mi_r_7048_5p,mmu_mi_r_7050_3p,mmu_mi_r_7056_5p,mmu_mi_r_7057_5p,mmu_mi_r_7060_3p,mmu_mi_r_7060_5p,mmu_mi_r_7061_5p,mmu_mi_r_7068_3p,mmu_mi_r_707,mmu_mi_r_7076_3p,mmu_mi_r_7079_5p,mmu_mi_r_7080_5p,mmu_mi_r_7085_5p,mmu_mi_r_7089_3p,mmu_mi_r_7090_5p,mmu_mi_r_7092_5p,mmu_mi_r_7116_5p,mmu_mi_r_7118_3p,mmu_mi_r_718,mmu_mi_r_7212_3p,mmu_mi_r_7221_5p,mmu_mi_r_7225_5p,mmu_mi_r_7227_3p,mmu_mi_r_7235_5p,mmu_mi_r_7239_5p,mmu_mi_r_7240_3p,mmu_mi_r_760_3p,mmu_mi_r_7648_5p,mmu_mi_r_7654_5p,mmu_mi_r_7661_3p,mmu_mi_r_7661_5p,mmu_mi_r_7662_3p,mmu_mi_r_7668_3p,mmu_mi_r_7669_3p,mmu_mi_r_7674_3p,mmu_mi_r_7678_3p,mmu_mi_r_7678_5p,mmu_mi_r_7679_5p,mmu_mi_r_7680_5p,mmu_mi_r_7685_3p,mmu_mi_r_802_3p,mmu_mi_r_8097,mmu_mi_r_8101,mmu_mi_r_8110,mmu_mi_r_8115,mmu_mi_r_8120,mmu_mi_r_872_5p,mmu_mi_r_875_5p,mmu_mi_r_877_5p,mmu_mi_r_879_5p,mmu_mi_r_881_5p,mmu_mi_r_883a_5p,mmu_mi_r_9_5p,mmu_mi_r_92a_1_5p,mmu_mi_r_96_5p,mmu_mi_r_98_3p,mmu_mi_r_99a_3p,mmu_pi_r_000366_gb_dq540412_mus_musculus_17_25602713_25602739_plus,mmu_pi_r_000366_gb_dq540412_mus_musculus_6_86369552_86369578_plus,mmu_pi_r_000414_gb_dq540526_mus_musculus_16_18446800_18446827_minus,mmu_pi_r_000691_gb_dq541218_mus_musculus_11_74136081_74136106_plus,mmu_pi_r_000958_gb_dq541851_mus_musculus_17_39456228_39456254_plus,mmu_pi_r_004374_gb_dq552696_mus_musculus_9_110126452_110126481_plus,mmu_pi_r_004567_gb_dq553409_mus_musculus_17_39455256_39455282_plus,mmu_pi_r_006854_gb_dq562105_mus_musculus_12_59958363_59958391_plus,mmu_pi_r_011460_gb_dq688079_mus_musculus_9_67527113_67527141_minus,mmu_pi_r_021807_gb_dq703459_mus_musculus_5_115112996_115113025_minus,mmu_pi_r_027673_gb_dq711996_mus_musculus_11_106317081_106317102_plus,mmu_pi_r_030152_gb_dq715561_mus_musculus_18_67167521_67167549_minus,mmu_pi_r_032974_gb_dq719597_mus_musculus_4_130021751_130021778_plus,mmu_pi_r_036747_gb_dq725127_mus_musculus_10_62058989_62059017_plus,mmu_pi_r_038328_gb_pi_rna_t47_mus_musculus_6_3151091_3151120_plus
682.283	x682_283_A	mmu_let_7e_3p,mmu_let_7g_3p,mmu_let_7j,mmu_mi_r_101b_5p,mmu_mi_r_103_1_5p,mmu_mi_r_105,mmu_mi_r_10a_3p,mmu_mi_r_1224_3p,mmu_mi_r_1251_5p,mmu_mi_r_129_1_3p,mmu_mi_r_129_5p,mmu_mi_r_129b_5p,mmu_mi_r_130a_5p,mmu_mi_r_133a_5p,mmu_mi_r_135a_5p,mmu_mi_r_138_2_3p,mmu_mi_r_141_5p,mmu_mi_r_146a_5p,mmu_mi_r_146b_3p,mmu_mi_r_146b_5p,mmu_mi_r_147_3p,mmu_mi_r_147_5p,mmu_mi_r_148a_5p,mmu_mi_r_16_2_3p,mmu_mi_r_188_5p,mmu_mi_r_1895,mmu_mi_r_1898,mmu_mi_r_1906,mmu_mi_r_190a_3p,mmu_mi_r_191_3p,mmu_mi_r_1912_5p,mmu_mi_r_1929_5p,mmu_mi_r_1932,mmu_mi_r_1934_3p,mmu_mi_r_1934_5p,mmu_mi_r_1938,mmu_mi_r_193b_3p,mmu_mi_r_1940,mmu_mi_r_1943_3p,mmu_mi_r_1945,mmu_mi_r_1946a,mmu_mi_r_1946b,mmu_mi_r_1947_3p,mmu_mi_r_1949,mmu_mi_r_1950,mmu_mi_r_195a_3p,mmu_mi_r_1961,mmu_mi_r_1963,mmu_mi_r_1964_3p,mmu_mi_r_196a_5p,mmu_mi_r_1970,mmu_mi_r_1971,mmu_mi_r_19a_5p,mmu_mi_r_19b_1_5p,mmu_mi_r_1b_3p,mmu_mi_r_205_3p,mmu_mi_r_208a_5p,mmu_mi_r_20a_3p,mmu_mi_r_20b_3p,mmu_mi_r_212_3p,mmu_mi_r_218_5p,mmu_mi_r_219a_1_3p,mmu_mi_r_21a_3p,mmu_mi_r_223_5p,mmu_mi_r_24_2_5p,mmu_mi_r_28a_5p,mmu_mi_r_302c_5p,mmu_mi_r_3057_5p,mmu_mi_r_3058_3p,mmu_mi_r_3058_5p,mmu_mi_r_3060_3p,mmu_mi_r_3062_3p,mmu_mi_r_3064_5p,mmu_mi_r_3066_5p,mmu_mi_r_3067_5p,mmu_mi_r_3069_3p,mmu_mi_r_3074_1_3p,mmu_mi_r_3079_5p,mmu_mi_r_3085_3p,mmu_mi_r_3088_3p,mmu_mi_r_3094_3p,mmu_mi_r_3097_3p,mmu_mi_r_3098_5p,mmu_mi_r_30c_2_3p,mmu_mi_r_3102_5p_2_5p,mmu_mi_r_3108_5p,mmu_mi_r_3112_3p,mmu_mi_r_327,mmu_mi_r_33_3p,mmu_mi_r_33_5p,mmu_mi_r_331_5p,mmu_mi_r_335_3p,mmu_mi_r_338_5p,mmu_mi_r_344b_3p,mmu_mi_r_344c_3p,mmu_mi_r_3470b,mmu_mi_r_3473f.,mmu_mi_r_3473g,mmu_mi_r_34b_3p,mmu_mi_r_34c_5p,mmu_mi_r_350_5p,mmu_mi_r_3535,mmu_mi_r_3544_3p,mmu_mi_r_3569_3p,mmu_mi_r_3572_5p,mmu_mi_r_3620_5p,mmu_mi_r_374c_5p,mmu_mi_r_383_3p,mmu_mi_r_384_3p,mmu_mi_r_3966,mmu_mi_r_411_3p,mmu_mi_r_429_5p,mmu_mi_r_451b,mmu_mi_r_455_3p,mmu_mi_r_466a_3p_mmu_mi_r_466e_3p,mmu_mi_r_467g,mmu_mi_r_487b_3p,mmu_mi_r_490_3p,mmu_mi_r_490_5p,mmu_mi_r_494_5p,mmu_mi_r_495_3p,mmu_mi_r_496a_5p,mmu_mi_r_497a_3p,mmu_mi_r_497b,mmu_mi_r_504_5p,mmu_mi_r_505_3p,mmu_mi_r_5103,mmu_mi_r_5107_3p,mmu_mi_r_511_5p,mmu_mi_r_5114,mmu_mi_r_5119,mmu_mi_r_5120,mmu_mi_r_5124b,mmu_mi_r_5125,mmu_mi_r_5129_3p,mmu_mi_r_5129_5p,mmu_mi_r_5134_5p,mmu_mi_r_532_3p,mmu_mi_r_543_3p,mmu_mi_r_544_5p,mmu_mi_r_547_3p,mmu_mi_r_551b_5p,mmu_mi_r_5709_5p,mmu_mi_r_5710,mmu_mi_r_582_5p,mmu_mi_r_590_5p,mmu_mi_r_592_5p,mmu_mi_r_598_5p,mmu_mi_r_615_5p,mmu_mi_r_6237,mmu_mi_r_6355,mmu_mi_r_6357,mmu_mi_r_6378,mmu_mi_r_6389,mmu_mi_r_6390,mmu_mi_r_6395,mmu_mi_r_6419,mmu_mi_r_653_5p,mmu_mi_r_6546_3p,mmu_mi_r_664_5p,mmu_mi_r_669i,mmu_mi_r_669p_3p,mmu_mi_r_670_5p,mmu_mi_r_673_3p,mmu_mi_r_675_3p,mmu_mi_r_675_5p,mmu_mi_r_681,mmu_mi_r_6900_3p,mmu_mi_r_6908_3p,mmu_mi_r_691,mmu_mi_r_6912_5p,mmu_mi_r_6914_3p,mmu_mi_r_6914_5p,mmu_mi_r_6915_3p,mmu_mi_r_6917_5p,mmu_mi_r_6919_3p,mmu_mi_r_6921_3p,mmu_mi_r_693_3p,mmu_mi_r_6930_5p,mmu_mi_r_6932_5p,mmu_mi_r_6933_3p,mmu_mi_r_6937_5p,mmu_mi_r_6945_5p,mmu_mi_r_6947_5p,mmu_mi_r_6949_3p,mmu_mi_r_6949_5p,mmu_mi_r_6951_5p,mmu_mi_r_6952_3p,mmu_mi_r_6953_3p,mmu_mi_r_6953_5p,mmu_mi_r_6956_3p,mmu_mi_r_6958_5p,mmu_mi_r_6960_5p,mmu_mi_r_6973b_3p,mmu_mi_r_6974_5p,mmu_mi_r_698_5p,mmu_mi_r_6983_5p,mmu_mi_r_6986_5p,mmu_mi_r_6991_3p,mmu_mi_r_6994_3p,mmu_mi_r_701_3p,mmu_mi_r_7012_5p,mmu_mi_r_7015_3p,mmu_mi_r_7015_5p,mmu_mi_r_7024_5p,mmu_mi_r_7027_3p,mmu_mi_r_7027_5p,mmu_mi_r_7029_3p,mmu_mi_r_7029_5p,mmu_mi_r_7034_5p,mmu_mi_r_7052_5p,mmu_mi_r_7058_3p,mmu_mi_r_7061_5p,mmu_mi_r_7062_5p,mmu_mi_r_7063_5p,mmu_mi_r_7065_3p,mmu_mi_r_7070_5p,mmu_mi_r_7081_3p,mmu_mi_r_7083_3p,mmu_mi_r_7088_3p,mmu_mi_r_709,mmu_mi_r_7091_3p,mmu_mi_r_7091_5p,mmu_mi_r_7093_3p,mmu_mi_r_7093_5p,mmu_mi_r_7115_5p,mmu_mi_r_7116_5p,mmu_mi_r_7117_5p,mmu_mi_r_719,mmu_mi_r_7212_5p,mmu_mi_r_7219_5p,mmu_mi_r_7223_5p,mmu_mi_r_7228_3p,mmu_mi_r_7235_5p,mmu_mi_r_7239_3p,mmu_mi_r_742_3p,mmu_mi_r_7646_5p,mmu_mi_r_7648_5p,mmu_mi_r_7651_3p,mmu_mi_r_7653_5p,mmu_mi_r_7654_5p,mmu_mi_r_7655_5p,mmu_mi_r_7656_3p,mmu_mi_r_7658_5p,mmu_mi_r_7665_5p,mmu_mi_r_7667_3p,mmu_mi_r_7667_5p,mmu_mi_r_7671_5p,mmu_mi_r_7675_3p,mmu_mi_r_7675_5p,mmu_mi_r_7687_5p,mmu_mi_r_7a_2_3p,mmu_mi_r_8091,mmu_mi_r_8092,mmu_mi_r_8097,mmu_mi_r_8105,mmu_mi_r_8109,mmu_mi_r_8116,mmu_mi_r_873a_5p,mmu_mi_r_877_3p,mmu_mi_r_877_5p,mmu_mi_r_92a_1_5p,mmu_pi_r_000159_gb_dq539904_mus_musculus_9_118523651_118523678_minus,mmu_pi_r_000219_gb_dq540058_mus_musculus_17_39455665_39455691_plus,mmu_pi_r_004086_gb_dq551625_mus_musculus_2_129969457_129969485_plus,mmu_pi_r_020692_gb_dq701869_mus_musculus_5_115840031_115840052_plus,mmu_pi_r_022545_gb_dq704578_mus_musculus_9_67495705_67495734_minus,mmu_pi_r_023189_gb_dq705481_mus_musculus_16_18197850_18197871_minus,mmu_pi_r_025576_gb_dq708952_mus_musculus_x_6405415_6405436_minus,mmu_pi_r_028241_gb_dq712821_mus_musculus_x_56741084_56741107_minus,mmu_pi_r_028252_gb_dq712837_mus_musculus_7_81403433_81403455_plus,mmu_pi_r_028975_gb_dq713872_mus_musculus_x_6405378_6405399_minus,mmu_pi_r_032015_gb_dq718174_mus_musculus_2_10427172_10427193_plus,mmu_pi_r_032974_gb_dq719597_mus_musculus_4_130021751_130021778_plus,mmu_pi_r_038323_gb_pi_rna_t34_mus_musculus_14_18071278_18071308_plus
710.314	x710_314_A	mmu_let_7c_1_3p,mmu_mi_r_105,mmu_mi_r_106a_3p,mmu_mi_r_1193_5p,mmu_mi_r_1194,mmu_mi_r_122_3p,mmu_mi_r_1249_5p,mmu_mi_r_127_3p,mmu_mi_r_134_5p,mmu_mi_r_135a_2_3p,mmu_mi_r_136_5p,mmu_mi_r_155_3p,mmu_mi_r_181b_2_3p,mmu_mi_r_181c_3p,mmu_mi_r_182_3p,mmu_mi_r_1901,mmu_mi_r_1902,mmu_mi_r_1904,mmu_mi_r_192_5p,mmu_mi_r_193a_3p,mmu_mi_r_193b_5p,mmu_mi_r_194_5p,mmu_mi_r_1941_3p,mmu_mi_r_1947_5p,mmu_mi_r_1955_3p,mmu_mi_r_1957a,mmu_mi_r_1961,mmu_mi_r_196a_2_3p,mmu_mi_r_1a_1_5p,mmu_mi_r_1a_2_5p,mmu_mi_r_1a_3p,mmu_mi_r_1b_5p,mmu_mi_r_200a_3p,mmu_mi_r_200b_5p,mmu_mi_r_203_3p,mmu_mi_r_210_5p,mmu_mi_r_215_3p,mmu_mi_r_215_5p,mmu_mi_r_216b_5p,mmu_mi_r_217_5p,mmu_mi_r_219b_3p,mmu_mi_r_21b,mmu_mi_r_2861,mmu_mi_r_290a_5p,mmu_mi_r_290b_3p,mmu_mi_r_291b_3p,mmu_mi_r_294_5p,mmu_mi_r_297a_3p_mmu_mi_r_297b_3p_mmu_mi_r_297c_3p,mmu_mi_r_297a_5p,mmu_mi_r_300_3p,mmu_mi_r_301b_5p,mmu_mi_r_302a_5p,mmu_mi_r_302c_3p,mmu_mi_r_3060_5p,mmu_mi_r_3061_3p,mmu_mi_r_3065_5p,mmu_mi_r_3069_5p,mmu_mi_r_3072_3p,mmu_mi_r_3073b_3p,mmu_mi_r_3073b_5p,mmu_mi_r_3074_1_3p,mmu_mi_r_3078_5p,mmu_mi_r_3079_3p,mmu_mi_r_3081_5p,mmu_mi_r_3086_3p,mmu_mi_r_3087_5p,mmu_mi_r_3089_3p,mmu_mi_r_3091_3p,mmu_mi_r_3094_3p,mmu_mi_r_3098_3p,mmu_mi_r_3099_3p,mmu_mi_r_30b_3p,mmu_mi_r_3103_3p,mmu_mi_r_3113_3p,mmu_mi_r_32_3p,mmu_mi_r_323_5p,mmu_mi_r_325_5p,mmu_mi_r_344e_3p,mmu_mi_r_344e_5p_mmu_mi_r_344h_5p,mmu_mi_r_3472,mmu_mi_r_3475_3p,mmu_mi_r_34a_3p,mmu_mi_r_350_5p,mmu_mi_r_3618_3p,mmu_mi_r_3620_3p,mmu_mi_r_363_5p,mmu_mi_r_365_2_5p,mmu_mi_r_367_5p,mmu_mi_r_369_3p,mmu_mi_r_370_3p,mmu_mi_r_375_3p,mmu_mi_r_376a_5p,mmu_mi_r_376c_3p,mmu_mi_r_378d,mmu_mi_r_382_5p,mmu_mi_r_3960,mmu_mi_r_3971,mmu_mi_r_409_3p,mmu_mi_r_431_5p,mmu_mi_r_449b,mmu_mi_r_452_5p,mmu_mi_r_467c_3p,mmu_mi_r_470_3p,mmu_mi_r_471_3p,mmu_mi_r_471_5p,mmu_mi_r_485_3p,mmu_mi_r_487b_3p,mmu_mi_r_493_5p,mmu_mi_r_494_5p,mmu_mi_r_499_5p,mmu_mi_r_503_5p,mmu_mi_r_5131,mmu_mi_r_5132_5p,mmu_mi_r_539_3p,mmu_mi_r_540_5p,mmu_mi_r_5623_3p,mmu_mi_r_5624_3p,mmu_mi_r_5626_3p,mmu_mi_r_5709_5p,mmu_mi_r_590_3p,mmu_mi_r_598_3p,mmu_mi_r_599,mmu_mi_r_6244,mmu_mi_r_6335,mmu_mi_r_6337,mmu_mi_r_6346,mmu_mi_r_6353,mmu_mi_r_6359,mmu_mi_r_6362,mmu_mi_r_6370,mmu_mi_r_6371,mmu_mi_r_6385,mmu_mi_r_6391,mmu_mi_r_6396,mmu_mi_r_6403,mmu_mi_r_6406,mmu_mi_r_6408,mmu_mi_r_6481,mmu_mi_r_6537_5p,mmu_mi_r_6539,mmu_mi_r_668_5p,mmu_mi_r_669b_5p,mmu_mi_r_669e_5p,mmu_mi_r_669g,mmu_mi_r_669j,mmu_mi_r_669l_3p,mmu_mi_r_669m_3p,mmu_mi_r_672_5p,mmu_mi_r_673_3p,mmu_mi_r_677_5p,mmu_mi_r_678,mmu_mi_r_686,mmu_mi_r_6896_3p,mmu_mi_r_6905_3p,mmu_mi_r_6905_5p,mmu_mi_r_6906_3p,mmu_mi_r_6907_5p,mmu_mi_r_6909_5p,mmu_mi_r_6910_3p,mmu_mi_r_6921_3p,mmu_mi_r_6925_3p,mmu_mi_r_6925_5p,mmu_mi_r_6928_5p,mmu_mi_r_693_3p,mmu_mi_r_6930_3p,mmu_mi_r_694,mmu_mi_r_6940_5p,mmu_mi_r_6943_5p,mmu_mi_r_6945_3p,mmu_mi_r_6945_5p,mmu_mi_r_6949_3p,mmu_mi_r_6953_5p,mmu_mi_r_6957_5p,mmu_mi_r_6963_5p,mmu_mi_r_6970_5p,mmu_mi_r_6971_3p,mmu_mi_r_6972_3p,mmu_mi_r_6985_5p,mmu_mi_r_6987_3p,mmu_mi_r_6990_3p,mmu_mi_r_6991_5p,mmu_mi_r_6992_3p,mmu_mi_r_6997_3p,mmu_mi_r_7018_3p,mmu_mi_r_7020_5p,mmu_mi_r_7021_3p,mmu_mi_r_7026_3p,mmu_mi_r_7038_3p,mmu_mi_r_7043_5p,mmu_mi_r_7049_3p,mmu_mi_r_7050_3p,mmu_mi_r_7051_3p,mmu_mi_r_7053_5p,mmu_mi_r_7055_5p,mmu_mi_r_7057_3p,mmu_mi_r_706,mmu_mi_r_7073_3p,mmu_mi_r_7075_5p,mmu_mi_r_7078_3p,mmu_mi_r_7079_3p,mmu_mi_r_7080_3p,mmu_mi_r_7081_5p,mmu_mi_r_7083_3p,mmu_mi_r_7085_5p,mmu_mi_r_7086_3p,mmu_mi_r_7090_5p,mmu_mi_r_7094b_2_5p,mmu_mi_r_710,mmu_mi_r_711,mmu_mi_r_7115_3p,mmu_mi_r_7116_3p,mmu_mi_r_7118_3p,mmu_mi_r_7210_3p,mmu_mi_r_7213_3p,mmu_mi_r_7216_3p,mmu_mi_r_7218_3p,mmu_mi_r_7221_5p,mmu_mi_r_7222_3p,mmu_mi_r_7224_3p,mmu_mi_r_7225_5p,mmu_mi_r_7226_5p,mmu_mi_r_7227_5p,mmu_mi_r_7231_3p,mmu_mi_r_7233_3p,mmu_mi_r_741_5p,mmu_mi_r_743b_3p,mmu_mi_r_7578,mmu_mi_r_760_5p,mmu_mi_r_7646_3p,mmu_mi_r_7647_5p,mmu_mi_r_7656_3p,mmu_mi_r_7660_5p,mmu_mi_r_7661_5p,mmu_mi_r_7662_3p,mmu_mi_r_7664_3p,mmu_mi_r_7665_3p,mmu_mi_r_7665_5p,mmu_mi_r_7667_3p,mmu_mi_r_7670_3p,mmu_mi_r_7675_5p,mmu_mi_r_7677_5p,mmu_mi_r_7683_3p,mmu_mi_r_7684_3p,mmu_mi_r_7684_5p,mmu_mi_r_7687_5p,mmu_mi_r_7689_3p,mmu_mi_r_802_5p,mmu_mi_r_8101,mmu_mi_r_8102,mmu_mi_r_8103,mmu_mi_r_8113,mmu_mi_r_8115,mmu_mi_r_8116,mmu_mi_r_8118,mmu_mi_r_871_3p,mmu_mi_r_875_3p,mmu_mi_r_879_5p,mmu_mi_r_9768_5p,mmu_pi_r_000159_gb_dq539904_mus_musculus_2_73668844_73668871_plus,mmu_pi_r_000619_gb_dq540976_mus_musculus_17_39454691_39454717_plus,mmu_pi_r_000622_gb_dq540988_mus_musculus_2_5296560_5296587_minus,mmu_pi_r_000622_gb_dq540988_mus_musculus_3_5843428_5843455_plus,mmu_pi_r_000691_gb_dq541218_mus_musculus_11_74136081_74136106_plus,mmu_pi_r_000691_gb_dq541218_mus_musculus_8_126424875_126424900_minus,mmu_pi_r_000691_gb_dq541218_mus_musculus_8_126455417_126455442_minus,mmu_pi_r_000691_gb_dq541218_mus_musculus_8_126457090_126457115_minus,mmu_pi_r_000691_gb_dq541218_mus_musculus_8_126467244_126467269_minus,mmu_pi_r_000691_gb_dq541218_mus_musculus_8_126472331_126472356_minus,mmu_pi_r_000691_gb_dq541218_mus_musculus_8_126492702_126492727_minus,mmu_pi_r_000691_gb_dq541218_mus_musculus_8_126494409_126494434_minus,mmu_pi_r_002643_gb_dq547181_mus_musculus_3_5843705_5843732_plus,mmu_pi_r_005109_gb_dq555094_mus_musculus_5_146565258_146565289_plus,mmu_pi_r_011460_gb_dq688079_mus_musculus_9_67527113_67527141_minus,mmu_pi_r_020492_gb_dq701563_mus_musculus_11_108827972_108827997_minus,mmu_pi_r_020554_gb_dq701662_mus_musculus_2_92351039_92351069_plus,mmu_pi_r_023189_gb_dq705481_mus_musculus_16_18197850_18197871_minus,mmu_pi_r_037947_gb_dq726864_mus_musculus_9_54023523_54023552_minus,mmu_pi_r_038323_gb_pi_rna_t34_mus_musculus_14_18071278_18071308_plus,mmu_pi_r_038328_gb_pi_rna_t47_mus_musculus_6_3151091_3151120_plus
726.589	x726_589_H	mmu_let_7a_1_3p_mmu_let_7c_2_3p,mmu_let_7a_2_3p,mmu_let_7a_5p,mmu_let_7b_3p,mmu_let_7b_5p,mmu_let_7c_5p,mmu_let_7d_3p,mmu_let_7d_5p,mmu_let_7f_5p,mmu_let_7g_3p,mmu_let_7g_5p,mmu_let_7i_3p,mmu_let_7i_5p,mmu_let_7k,mmu_mi_r_101a_3p,mmu_mi_r_101a_5p,mmu_mi_r_101b_3p,mmu_mi_r_101c,mmu_mi_r_103_1_5p,mmu_mi_r_103_3p,mmu_mi_r_106b_3p,mmu_mi_r_107_3p,mmu_mi_r_107_5p,mmu_mi_r_1188_3p,mmu_mi_r_1194,mmu_mi_r_1198_5p,mmu_mi_r_122_3p,mmu_mi_r_1247_3p,mmu_mi_r_1264_3p,mmu_mi_r_128_3p,mmu_mi_r_1306_3p,mmu_mi_r_1306_5p,mmu_mi_r_130b_3p,mmu_mi_r_130b_5p,mmu_mi_r_133a_3p,mmu_mi_r_133a_5p,mmu_mi_r_133b_3p,mmu_mi_r_135a_1_3p,mmu_mi_r_135b_5p,mmu_mi_r_137_5p,mmu_mi_r_140_3p,mmu_mi_r_140_5p,mmu_mi_r_142a_3p,mmu_mi_r_142a_5p,mmu_mi_r_144_3p,mmu_mi_r_146b_3p,mmu_mi_r_148b_3p,mmu_mi_r_148b_5p,mmu_mi_r_149_5p,mmu_mi_r_150_5p,mmu_mi_r_154_3p,mmu_mi_r_15a_3p,mmu_mi_r_15a_5p,mmu_mi_r_15b_3p,mmu_mi_r_15b_5p,mmu_mi_r_16_1_3p,mmu_mi_r_16_2_3p,mmu_mi_r_16_5p,mmu_mi_r_181b_2_3p,mmu_mi_r_181c_3p,mmu_mi_r_181c_5p,mmu_mi_r_181d_5p,mmu_mi_r_1839_5p,mmu_mi_r_184_3p,mmu_mi_r_184_5p,mmu_mi_r_1843a_5p,mmu_mi_r_185_3p,mmu_mi_r_185_5p,mmu_mi_r_186_5p,mmu_mi_r_1894_3p,mmu_mi_r_1896,mmu_mi_r_1898,mmu_mi_r_18a_3p,mmu_mi_r_1903,mmu_mi_r_1905,mmu_mi_r_1907,mmu_mi_r_190b_5p,mmu_mi_r_191_5p,mmu_mi_r_1934_3p,mmu_mi_r_1943_3p,mmu_mi_r_1946a,mmu_mi_r_1947_5p,mmu_mi_r_1948_3p,mmu_mi_r_1948_5p,mmu_mi_r_1951,mmu_mi_r_1958,mmu_mi_r_195a_5p,mmu_mi_r_1961,mmu_mi_r_1968_3p,mmu_mi_r_1970,mmu_mi_r_1981_5p,mmu_mi_r_19a_3p,mmu_mi_r_19a_5p,mmu_mi_r_19b_3p,mmu_mi_r_1a_2_5p,mmu_mi_r_202_3p,mmu_mi_r_202_5p,mmu_mi_r_204_3p,mmu_mi_r_208b_3p,mmu_mi_r_208b_5p,mmu_mi_r_20a_5p,mmu_mi_r_20b_5p,mmu_mi_r_210_3p,mmu_mi_r_211_3p,mmu_mi_r_2139,mmu_mi_r_217_3p,mmu_mi_r_218_1_3p,mmu_mi_r_218_2_3p,mmu_mi_r_21c,mmu_mi_r_222_3p,mmu_mi_r_222_5p,mmu_mi_r_23b_5p,mmu_mi_r_24_2_5p,mmu_mi_r_25_3p,mmu_mi_r_25_5p,mmu_mi_r_26a_1_3p,mmu_mi_r_26a_5p,mmu_mi_r_26b_5p,mmu_mi_r_28b,mmu_mi_r_292a_5p,mmu_mi_r_293_5p,mmu_mi_r_294_5p,mmu_mi_r_296_5p,mmu_mi_r_297a_3p_mmu_mi_r_297b_3p_mmu_mi_r_297c_3p,mmu_mi_r_297b_5p,mmu_mi_r_298_5p,mmu_mi_r_299b_3p,mmu_mi_r_300_3p,mmu_mi_r_301a_3p,mmu_mi_r_302b_5p,mmu_mi_r_302c_3p,mmu_mi_r_3058_3p,mmu_mi_r_3059_3p,mmu_mi_r_3062_3p,mmu_mi_r_3064_3p,mmu_mi_r_3064_5p,mmu_mi_r_3068_5p,mmu_mi_r_3070_5p,mmu_mi_r_3075_3p,mmu_mi_r_3075_5p,mmu_mi_r_3077_3p,mmu_mi_r_3079_3p,mmu_mi_r_3082_5p,mmu_mi_r_3089_5p,mmu_mi_r_3093_5p,mmu_mi_r_3095_3p,mmu_mi_r_30a_3p,mmu_mi_r_30a_5p,mmu_mi_r_30b_5p,mmu_mi_r_30c_1_3p,mmu_mi_r_30c_2_3p,mmu_mi_r_30c_5p,mmu_mi_r_30d_5p,mmu_mi_r_30e_3p,mmu_mi_r_30e_5p,mmu_mi_r_3101_3p,mmu_mi_r_3101_5p,mmu_mi_r_3102_3p,mmu_mi_r_3102_3p_2_3p,mmu_mi_r_3102_5p_2_5p,mmu_mi_r_3104_5p,mmu_mi_r_3108_3p,mmu_mi_r_3154,mmu_mi_r_32_3p,mmu_mi_r_322_3p,mmu_mi_r_322_5p,mmu_mi_r_328_3p,mmu_mi_r_330_5p,mmu_mi_r_337_3p,mmu_mi_r_337_5p,mmu_mi_r_340_5p,mmu_mi_r_341_3p,mmu_mi_r_341_5p,mmu_mi_r_342_3p,mmu_mi_r_343,mmu_mi_r_344b_5p,mmu_mi_r_344c_3p,mmu_mi_r_344c_5p,mmu_mi_r_344d_3_5p,mmu_mi_r_344d_3p,mmu_mi_r_345_3p,mmu_mi_r_3471,mmu_mi_r_3473d,mmu_mi_r_3475_3p,mmu_mi_r_350_3p,mmu_mi_r_350_5p,mmu_mi_r_3572_3p,mmu_mi_r_361_3p,mmu_mi_r_3620_5p,mmu_mi_r_369_5p,mmu_mi_r_374b_5p,mmu_mi_r_376a_3p,mmu_mi_r_376b_3p,mmu_mi_r_376b_5p,mmu_mi_r_377_3p,mmu_mi_r_378a_3p,mmu_mi_r_378a_5p,mmu_mi_r_378b,mmu_mi_r_378c,mmu_mi_r_378d,mmu_mi_r_383_3p,mmu_mi_r_3960,mmu_mi_r_3965,mmu_mi_r_3967,mmu_mi_r_3970,mmu_mi_r_409_3p,mmu_mi_r_421_3p,mmu_mi_r_425_3p,mmu_mi_r_425_5p,mmu_mi_r_450b_3p,mmu_mi_r_450b_5p,mmu_mi_r_451a,mmu_mi_r_452_5p,mmu_mi_r_455_3p,mmu_mi_r_466i_3p,mmu_mi_r_468_5p,mmu_mi_r_486a_3p,mmu_mi_r_486a_5p,mmu_mi_r_486b_3p,mmu_mi_r_486b_5p,mmu_mi_r_487b_5p,mmu_mi_r_490_3p,mmu_mi_r_491_5p,mmu_mi_r_494_3p,mmu_mi_r_497a_5p,mmu_mi_r_497b,mmu_mi_r_499_3p,mmu_mi_r_503_3p,mmu_mi_r_503_5p,mmu_mi_r_504_5p,mmu_mi_r_5046,mmu_mi_r_5099,mmu_mi_r_5100,mmu_mi_r_5123,mmu_mi_r_5131,mmu_mi_r_5135,mmu_mi_r_532_5p,mmu_mi_r_542_3p,mmu_mi_r_542_5p,mmu_mi_r_5616_3p,mmu_mi_r_5617_3p,mmu_mi_r_5618_5p,mmu_mi_r_5621_5p,mmu_mi_r_5622_3p,mmu_mi_r_5627_3p,mmu_mi_r_590_3p,mmu_mi_r_592_5p,mmu_mi_r_615_3p,mmu_mi_r_6237,mmu_mi_r_6241,mmu_mi_r_6335,mmu_mi_r_6337,mmu_mi_r_6339,mmu_mi_r_6341,mmu_mi_r_6345,mmu_mi_r_6353,mmu_mi_r_6355,mmu_mi_r_6366,mmu_mi_r_6367,mmu_mi_r_6368,mmu_mi_r_6371,mmu_mi_r_6375,mmu_mi_r_6379,mmu_mi_r_6381,mmu_mi_r_6392_3p,mmu_mi_r_6405,mmu_mi_r_6412,mmu_mi_r_6413,mmu_mi_r_6420,mmu_mi_r_6541,mmu_mi_r_6546_5p,mmu_mi_r_664_5p,mmu_mi_r_667_5p,mmu_mi_r_669c_5p,mmu_mi_r_669m_3p,mmu_mi_r_669n,mmu_mi_r_670_5p,mmu_mi_r_672_5p,mmu_mi_r_673_5p,mmu_mi_r_674_5p,mmu_mi_r_676_3p,mmu_mi_r_676_5p,mmu_mi_r_677_5p,mmu_mi_r_6903_3p,mmu_mi_r_6905_5p,mmu_mi_r_691,mmu_mi_r_6925_5p,mmu_mi_r_6929_5p,mmu_mi_r_6939_5p,mmu_mi_r_6942_3p,mmu_mi_r_6945_3p,mmu_mi_r_6947_5p,mmu_mi_r_6952_5p,mmu_mi_r_6955_5p,mmu_mi_r_6956_5p,mmu_mi_r_6958_5p,mmu_mi_r_6959_5p,mmu_mi_r_6960_5p,mmu_mi_r_6961_5p,mmu_mi_r_6962_5p,mmu_mi_r_6963_5p,mmu_mi_r_6964_5p,mmu_mi_r_6966_5p,mmu_mi_r_6967_3p,mmu_mi_r_6969_5p,mmu_mi_r_6973a_5p,mmu_mi_r_6976_5p,mmu_mi_r_6977_3p,mmu_mi_r_6977_5p,mmu_mi_r_6979_5p,mmu_mi_r_698_5p,mmu_mi_r_6987_3p,mmu_mi_r_6992_5p,mmu_mi_r_6993_5p,mmu_mi_r_6994_3p,mmu_mi_r_6999_5p,mmu_mi_r_700_3p,mmu_mi_r_7001_3p,mmu_mi_r_7001_5p,mmu_mi_r_7003_5p,mmu_mi_r_7004_3p,mmu_mi_r_7007_3p,mmu_mi_r_701_5p,mmu_mi_r_7011_5p,mmu_mi_r_7014_5p,mmu_mi_r_7015_3p,mmu_mi_r_7016_3p,mmu_mi_r_7020_5p,mmu_mi_r_7021_5p,mmu_mi_r_7023_5p,mmu_mi_r_7025_5p,mmu_mi_r_7030_3p,mmu_mi_r_7031_5p,mmu_mi_r_7033_3p,mmu_mi_r_7034_5p,mmu_mi_r_7041_5p,mmu_mi_r_7045_3p,mmu_mi_r_7048_5p,mmu_mi_r_705,mmu_mi_r_7054_3p,mmu_mi_r_7054_5p,mmu_mi_r_7055_3p,mmu_mi_r_7055_5p,mmu_mi_r_7057_3p,mmu_mi_r_7060_3p,mmu_mi_r_7063_3p,mmu_mi_r_7066_5p,mmu_mi_r_7067_5p,mmu_mi_r_7071_5p,mmu_mi_r_7074_5p,mmu_mi_r_7079_3p,mmu_mi_r_7080_3p,mmu_mi_r_7080_5p,mmu_mi_r_7084_3p,mmu_mi_r_7087_3p,mmu_mi_r_7091_3p,mmu_mi_r_7093_3p,mmu_mi_r_7115_5p,mmu_mi_r_7116_3p,mmu_mi_r_7116_5p,mmu_mi_r_7117_3p,mmu_mi_r_7118_3p,mmu_mi_r_721,mmu_mi_r_7220_3p,mmu_mi_r_7230_5p,mmu_mi_r_7234_5p,mmu_mi_r_7235_5p,mmu_mi_r_7243_3p,mmu_mi_r_742_3p,mmu_mi_r_743a_3p,mmu_mi_r_744_5p,mmu_mi_r_760_3p,mmu_mi_r_7646_5p,mmu_mi_r_7648_5p,mmu_mi_r_7652_5p,mmu_mi_r_7657_5p,mmu_mi_r_7658_5p,mmu_mi_r_7661_3p,mmu_mi_r_7665_5p,mmu_mi_r_7671_5p,mmu_mi_r_7679_5p,mmu_mi_r_7683_3p,mmu_mi_r_7688_5p,mmu_mi_r_770_3p,mmu_mi_r_7a_1_3p,mmu_mi_r_7a_5p,mmu_mi_r_8090,mmu_mi_r_8091,mmu_mi_r_8097,mmu_mi_r_8105,mmu_mi_r_8117,mmu_mi_r_873a_5p,mmu_mi_r_882,mmu_mi_r_883a_5p,mmu_mi_r_92a_3p,mmu_mi_r_92b_3p,mmu_mi_r_93_3p,mmu_mi_r_93_5p,mmu_mi_r_96_5p,mmu_mi_r_9768_3p,mmu_mi_r_98_5p,mmu_mi_r_99a_3p,mmu_mi_r_99b_3p,mmu_pi_r_000159_gb_dq539904_mus_musculus_2_73668844_73668871_plus,mmu_pi_r_000366_gb_dq540412_mus_musculus_17_25602713_25602739_plus,mmu_pi_r_000691_gb_dq541218_mus_musculus_8_126494409_126494434_minus,mmu_pi_r_000935_gb_dq541777_mus_musculus_6_47717737_47717766_minus,mmu_pi_r_004086_gb_dq551625_mus_musculus_2_129969457_129969485_plus,mmu_pi_r_004567_gb_dq553409_mus_musculus_17_39455256_39455282_plus,mmu_pi_r_005109_gb_dq555094_mus_musculus_5_146565258_146565289_plus,mmu_pi_r_017405_gb_dq696996_mus_musculus_11_65550994_65551015_minus,mmu_pi_r_020554_gb_dq701662_mus_musculus_2_92351039_92351069_plus,mmu_pi_r_023189_gb_dq705481_mus_musculus_16_18197850_18197871_minus,mmu_pi_r_024744_gb_dq707762_mus_musculus_17_66099819_66099850_minus,mmu_pi_r_028252_gb_dq712837_mus_musculus_7_81403433_81403455_plus,mmu_pi_r_032865_gb_dq719430_mus_musculus_2_116876592_116876614_plus
877.291	x877_291_A	mmu_let_7c_1_3p,mmu_let_7e_3p,mmu_let_7f_2_3p,mmu_let_7g_3p,mmu_mi_r_1187,mmu_mi_r_1190,mmu_mi_r_1191b_3p,mmu_mi_r_1199_5p,mmu_mi_r_124_3p,mmu_mi_r_1251_3p,mmu_mi_r_1258_3p,mmu_mi_r_1264_5p,mmu_mi_r_126b_3p,mmu_mi_r_126b_5p,mmu_mi_r_127_3p,mmu_mi_r_1298_3p,mmu_mi_r_129b_3p,mmu_mi_r_130c,mmu_mi_r_133b_3p,mmu_mi_r_139_3p,mmu_mi_r_146b_3p,mmu_mi_r_146b_5p,mmu_mi_r_148a_5p,mmu_mi_r_152_3p,mmu_mi_r_152_5p,mmu_mi_r_153_3p,mmu_mi_r_1839_3p,mmu_mi_r_187_3p,mmu_mi_r_188_5p,mmu_mi_r_1899,mmu_mi_r_18b_5p,mmu_mi_r_1927,mmu_mi_r_1932,mmu_mi_r_1933_5p,mmu_mi_r_1948_3p,mmu_mi_r_1952,mmu_mi_r_1963,mmu_mi_r_1966_3p,mmu_mi_r_196b_3p,mmu_mi_r_19b_1_5p,mmu_mi_r_1b_5p,mmu_mi_r_211_3p,mmu_mi_r_218_1_3p,mmu_mi_r_219a_2_3p,mmu_mi_r_21a_3p,mmu_mi_r_21c,mmu_mi_r_223_5p,mmu_mi_r_23b_5p,mmu_mi_r_24_2_5p,mmu_mi_r_27a_3p,mmu_mi_r_290a_5p,mmu_mi_r_290b_5p,mmu_mi_r_291b_5p,mmu_mi_r_297b_5p,mmu_mi_r_298_5p,mmu_mi_r_299a_3p,mmu_mi_r_299a_5p,mmu_mi_r_29b_2_5p,mmu_mi_r_302a_5p,mmu_mi_r_302c_5p,mmu_mi_r_3057_5p,mmu_mi_r_3059_5p,mmu_mi_r_3061_3p,mmu_mi_r_3073b_3p,mmu_mi_r_3074_5p,mmu_mi_r_3076_5p,mmu_mi_r_3079_5p,mmu_mi_r_3083_3p,mmu_mi_r_3095_5p,mmu_mi_r_3097_5p,mmu_mi_r_3100_5p,mmu_mi_r_3108_3p,mmu_mi_r_3109_3p,mmu_mi_r_3110_5p,mmu_mi_r_3113_5p,mmu_mi_r_325_5p,mmu_mi_r_328_5p,mmu_mi_r_329_5p,mmu_mi_r_330_3p,mmu_mi_r_335_3p,mmu_mi_r_337_5p,mmu_mi_r_344b_3p,mmu_mi_r_344e_3p,mmu_mi_r_344h_3p,mmu_mi_r_3473a,mmu_mi_r_3473e,mmu_mi_r_3535,mmu_mi_r_3552,mmu_mi_r_3618_5p,mmu_mi_r_367_5p,mmu_mi_r_374c_5p,mmu_mi_r_376c_3p,mmu_mi_r_3965,mmu_mi_r_3967,mmu_mi_r_412_5p,mmu_mi_r_448_3p,mmu_mi_r_449c_5p,mmu_mi_r_450a_2_3p,mmu_mi_r_450b_3p,mmu_mi_r_452_5p,mmu_mi_r_453,mmu_mi_r_463_3p,mmu_mi_r_465b_5p,mmu_mi_r_466d_5p,mmu_mi_r_466i_5p,mmu_mi_r_467b_3p,mmu_mi_r_467b_5p,mmu_mi_r_467d_5p,mmu_mi_r_467f,mmu_mi_r_483_3p,mmu_mi_r_485_3p,mmu_mi_r_487b_5p,mmu_mi_r_493_3p,mmu_mi_r_495_3p,mmu_mi_r_5099,mmu_mi_r_5118,mmu_mi_r_5126,mmu_mi_r_541_3p,mmu_mi_r_544_3p,mmu_mi_r_544_5p,mmu_mi_r_546,mmu_mi_r_547_5p,mmu_mi_r_5618_3p,mmu_mi_r_5620_5p,mmu_mi_r_5623_5p,mmu_mi_r_5624_3p,mmu_mi_r_5625_5p,mmu_mi_r_5627_5p,mmu_mi_r_5710,mmu_mi_r_592_5p,mmu_mi_r_615_5p,mmu_mi_r_6237,mmu_mi_r_6336,mmu_mi_r_6339,mmu_mi_r_6350,mmu_mi_r_6373,mmu_mi_r_6376,mmu_mi_r_6377,mmu_mi_r_6384,mmu_mi_r_6390,mmu_mi_r_6398,mmu_mi_r_6400,mmu_mi_r_6402,mmu_mi_r_6408,mmu_mi_r_6410,mmu_mi_r_652_5p,mmu_mi_r_653_5p,mmu_mi_r_6537_5p,mmu_mi_r_6538,mmu_mi_r_668_5p,mmu_mi_r_669b_5p,mmu_mi_r_669c_3p,mmu_mi_r_669l_3p,mmu_mi_r_669p_3p,mmu_mi_r_6715_5p,mmu_mi_r_677_5p,mmu_mi_r_679_3p,mmu_mi_r_682,mmu_mi_r_6896_5p,mmu_mi_r_6901_5p,mmu_mi_r_6906_5p,mmu_mi_r_6907_5p,mmu_mi_r_6908_3p,mmu_mi_r_6909_3p,mmu_mi_r_6915_3p,mmu_mi_r_6917_3p,mmu_mi_r_6920_3p,mmu_mi_r_6922_3p,mmu_mi_r_6923_3p,mmu_mi_r_6924_3p,mmu_mi_r_6924_5p,mmu_mi_r_6928_3p,mmu_mi_r_6930_5p,mmu_mi_r_6935_5p,mmu_mi_r_6938_3p,mmu_mi_r_6939_5p,mmu_mi_r_6941_5p,mmu_mi_r_6947_3p,mmu_mi_r_6948_3p,mmu_mi_r_6953_5p,mmu_mi_r_6956_5p,mmu_mi_r_6958_3p,mmu_mi_r_6959_3p,mmu_mi_r_6961_5p,mmu_mi_r_6963_5p,mmu_mi_r_6969_3p,mmu_mi_r_697,mmu_mi_r_6973b_5p,mmu_mi_r_6979_3p,mmu_mi_r_698_3p,mmu_mi_r_6981_5p,mmu_mi_r_6984_5p,mmu_mi_r_6985_5p,mmu_mi_r_6989_3p,mmu_mi_r_6992_5p,mmu_mi_r_6993_5p,mmu_mi_r_6995_3p,mmu_mi_r_6996_3p,mmu_mi_r_7000_3p,mmu_mi_r_7005_3p,mmu_mi_r_701_5p,mmu_mi_r_7011_5p,mmu_mi_r_7018_3p,mmu_mi_r_7029_3p,mmu_mi_r_7029_5p,mmu_mi_r_7031_5p,mmu_mi_r_7035_5p,mmu_mi_r_7036b_3p,mmu_mi_r_7037_3p,mmu_mi_r_7038_5p,mmu_mi_r_7039_3p,mmu_mi_r_7039_5p,mmu_mi_r_7041_3p,mmu_mi_r_7046_5p,mmu_mi_r_7047_3p,mmu_mi_r_7049_3p,mmu_mi_r_7050_3p,mmu_mi_r_7053_3p,mmu_mi_r_7054_3p,mmu_mi_r_7057_5p,mmu_mi_r_7068_5p,mmu_mi_r_7074_5p,mmu_mi_r_7079_3p,mmu_mi_r_708_5p,mmu_mi_r_7091_3p,mmu_mi_r_7093_5p,mmu_mi_r_7116_3p,mmu_mi_r_7117_3p,mmu_mi_r_7119_5p,mmu_mi_r_7211_3p,mmu_mi_r_7213_3p,mmu_mi_r_7218_5p,mmu_mi_r_7220_3p,mmu_mi_r_7222_5p,mmu_mi_r_7224_3p,mmu_mi_r_7238_5p,mmu_mi_r_7240_5p,mmu_mi_r_7241_5p,mmu_mi_r_741_3p,mmu_mi_r_758_3p,mmu_mi_r_761,mmu_mi_r_762,mmu_mi_r_7647_5p,mmu_mi_r_7648_3p,mmu_mi_r_7650_3p,mmu_mi_r_7654_3p,mmu_mi_r_7659_5p,mmu_mi_r_7664_5p,mmu_mi_r_7670_5p,mmu_mi_r_7671_3p,mmu_mi_r_7673_5p,mmu_mi_r_7675_3p,mmu_mi_r_7677_5p,mmu_mi_r_7684_5p,mmu_mi_r_7685_5p,mmu_mi_r_7686_3p,mmu_mi_r_7687_3p,mmu_mi_r_804,mmu_mi_r_8092,mmu_mi_r_8095,mmu_mi_r_8103,mmu_mi_r_8120,mmu_mi_r_873a_3p,mmu_mi_r_878_5p,mmu_mi_r_880_3p,mmu_mi_r_880_5p,mmu_mi_r_883a_5p,mmu_mi_r_92a_2_5p,mmu_mi_r_96_5p,mmu_mi_r_9768_5p,mmu_pi_r_000578_gb_dq540853_mus_musculus_17_39456112_39456137_plus,mmu_pi_r_000622_gb_dq540988_mus_musculus_x_112404287_112404314_minus,mmu_pi_r_000691_gb_dq541218_mus_musculus_8_126462165_126462190_minus,mmu_pi_r_001662_gb_dq544105_mus_musculus_2_151104333_151104361_minus,mmu_pi_r_002435_gb_dq546549_mus_musculus_17_39454808_39454832_plus,mmu_pi_r_003129_gb_dq548834_mus_musculus_17_27053369_27053399_plus,mmu_pi_r_004374_gb_dq552696_mus_musculus_9_110126452_110126481_plus,mmu_pi_r_013859_gb_dq691760_mus_musculus_9_54046065_54046096_minus,mmu_pi_r_020554_gb_dq701662_mus_musculus_2_92351039_92351069_plus,mmu_pi_r_022097_gb_dq703900_mus_musculus_17_25602688_25602719_plus,mmu_pi_r_022097_gb_dq703900_mus_musculus_6_86369527_86369558_plus,mmu_pi_r_022663_gb_dq704740_mus_musculus_17_27083947_27083976_plus,mmu_pi_r_023799_gb_dq706399_mus_musculus_2_151104329_151104359_minus,mmu_pi_r_029416_gb_dq714514_mus_musculus_9_67542046_67542076_plus,mmu_pi_r_032015_gb_dq718174_mus_musculus_2_10427172_10427193_plus,mmu_pi_r_038323_gb_pi_rna_t34_mus_musculus_13_44880547_44880577_minus,mmu_pi_r_038323_gb_pi_rna_t34_mus_musculus_14_18071278_18071308_plus,mmu_pi_r_039147_gb_pi_rna_2740_mus_musculus_9_118523649_118523669_minus
886.553	x886_553_A	mmu_let_7c_1_3p,mmu_let_7e_3p,mmu_mi_r_105,mmu_mi_r_106a_3p,mmu_mi_r_107_5p,mmu_mi_r_1188_5p,mmu_mi_r_1191b_5p,mmu_mi_r_1193_5p,mmu_mi_r_1194,mmu_mi_r_1197_3p,mmu_mi_r_1198_3p,mmu_mi_r_1199_5p,mmu_mi_r_122_3p,mmu_mi_r_1249_5p,mmu_mi_r_1258_5p,mmu_mi_r_126a_5p,mmu_mi_r_127_3p,mmu_mi_r_127_5p,mmu_mi_r_134_5p,mmu_mi_r_135a_2_3p,mmu_mi_r_135a_5p,mmu_mi_r_135b_3p,mmu_mi_r_136_5p,mmu_mi_r_137_5p,mmu_mi_r_138_1_3p,mmu_mi_r_146b_5p,mmu_mi_r_149_3p,mmu_mi_r_153_5p,mmu_mi_r_155_3p,mmu_mi_r_155_5p,mmu_mi_r_1668,mmu_mi_r_181a_1_3p,mmu_mi_r_181b_2_3p,mmu_mi_r_181c_3p,mmu_mi_r_181d_5p,mmu_mi_r_182_3p,mmu_mi_r_1843a_5p,mmu_mi_r_188_3p,mmu_mi_r_1893,mmu_mi_r_18b_3p,mmu_mi_r_1901,mmu_mi_r_1902,mmu_mi_r_1904,mmu_mi_r_1912_3p,mmu_mi_r_192_5p,mmu_mi_r_1930_3p,mmu_mi_r_1930_5p,mmu_mi_r_1932,mmu_mi_r_1934_3p,mmu_mi_r_193a_3p,mmu_mi_r_193b_5p,mmu_mi_r_194_5p,mmu_mi_r_1941_3p,mmu_mi_r_1942,mmu_mi_r_1943_3p,mmu_mi_r_1943_5p,mmu_mi_r_1947_5p,mmu_mi_r_1955_3p,mmu_mi_r_1957a,mmu_mi_r_1961,mmu_mi_r_196a_2_3p,mmu_mi_r_1982_3p,mmu_mi_r_1982_5p,mmu_mi_r_1983,mmu_mi_r_1a_1_5p,mmu_mi_r_1a_2_5p,mmu_mi_r_1a_3p,mmu_mi_r_1b_5p,mmu_mi_r_200a_3p,mmu_mi_r_200b_3p,mmu_mi_r_200b_5p,mmu_mi_r_200c_3p,mmu_mi_r_200c_5p,mmu_mi_r_201_3p,mmu_mi_r_203_3p,mmu_mi_r_210_5p,mmu_mi_r_2136,mmu_mi_r_215_3p,mmu_mi_r_215_5p,mmu_mi_r_216a_3p,mmu_mi_r_216b_5p,mmu_mi_r_217_5p,mmu_mi_r_219b_3p,mmu_mi_r_219c_3p,mmu_mi_r_21b,mmu_mi_r_22_5p,mmu_mi_r_222_5p,mmu_mi_r_224_3p,mmu_mi_r_27b_5p,mmu_mi_r_2861,mmu_mi_r_28b,mmu_mi_r_28c,mmu_mi_r_290a_5p,mmu_mi_r_290b_3p,mmu_mi_r_291a_5p,mmu_mi_r_291b_3p,mmu_mi_r_294_5p,mmu_mi_r_297a_3p_mmu_mi_r_297b_3p_mmu_mi_r_297c_3p,mmu_mi_r_297a_5p,mmu_mi_r_298_3p,mmu_mi_r_29a_5p,mmu_mi_r_29b_1_5p,mmu_mi_r_29b_3p,mmu_mi_r_300_3p,mmu_mi_r_300_5p,mmu_mi_r_301b_5p,mmu_mi_r_302a_5p,mmu_mi_r_302b_3p,mmu_mi_r_302c_3p,mmu_mi_r_302d_5p,mmu_mi_r_3060_5p,mmu_mi_r_3061_3p,mmu_mi_r_3062_5p,mmu_mi_r_3063_5p,mmu_mi_r_3064_5p,mmu_mi_r_3065_5p,mmu_mi_r_3068_3p,mmu_mi_r_3069_5p,mmu_mi_r_3070_3p,mmu_mi_r_3070_5p,mmu_mi_r_3072_3p,mmu_mi_r_3073b_3p,mmu_mi_r_3073b_5p,mmu_mi_r_3074_1_3p,mmu_mi_r_3075_5p,mmu_mi_r_3076_5p,mmu_mi_r_3078_5p,mmu_mi_r_3079_3p,mmu_mi_r_3081_5p,mmu_mi_r_3082_3p,mmu_mi_r_3082_5p,mmu_mi_r_3086_3p,mmu_mi_r_3087_5p,mmu_mi_r_3089_3p,mmu_mi_r_3090_3p,mmu_mi_r_3091_3p,mmu_mi_r_3094_3p,mmu_mi_r_3098_3p,mmu_mi_r_3099_3p,mmu_mi_r_30b_3p,mmu_mi_r_31_5p,mmu_mi_r_3103_3p,mmu_mi_r_3113_3p,mmu_mi_r_3113_5p,mmu_mi_r_32_3p,mmu_mi_r_322_3p,mmu_mi_r_322_5p,mmu_mi_r_323_5p,mmu_mi_r_325_5p,mmu_mi_r_335_3p,mmu_mi_r_337_3p,mmu_mi_r_337_5p,mmu_mi_r_338_5p,mmu_mi_r_344b_5p,mmu_mi_r_344e_3p,mmu_mi_r_344e_5p_mmu_mi_r_344h_5p,mmu_mi_r_3470a,mmu_mi_r_3472,mmu_mi_r_3473g,mmu_mi_r_3475_3p,mmu_mi_r_34a_3p,mmu_mi_r_350_5p,mmu_mi_r_351_5p,mmu_mi_r_3572_5p,mmu_mi_r_3618_3p,mmu_mi_r_3620_3p,mmu_mi_r_363_5p,mmu_mi_r_365_2_5p,mmu_mi_r_367_5p,mmu_mi_r_369_3p,mmu_mi_r_370_3p,mmu_mi_r_375_3p,mmu_mi_r_376a_5p,mmu_mi_r_376c_3p,mmu_mi_r_378d,mmu_mi_r_381_5p,mmu_mi_r_382_5p,mmu_mi_r_3960,mmu_mi_r_3971,mmu_mi_r_409_3p,mmu_mi_r_431_5p,mmu_mi_r_448_5p,mmu_mi_r_449a_5p,mmu_mi_r_449b,mmu_mi_r_452_5p,mmu_mi_r_466a_3p_mmu_mi_r_466e_3p,mmu_mi_r_466b_3p_mmu_mi_r_466c_3p_mmu_mi_r_466p_3p,mmu_mi_r_466d_3p,mmu_mi_r_466m_3p,mmu_mi_r_467a_5p,mmu_mi_r_467c_3p,mmu_mi_r_467c_5p,mmu_mi_r_467d_5p,mmu_mi_r_467e_3p,mmu_mi_r_467e_5p,mmu_mi_r_470_3p,mmu_mi_r_471_3p,mmu_mi_r_471_5p,mmu_mi_r_485_3p,mmu_mi_r_487b_3p,mmu_mi_r_493_5p,mmu_mi_r_494_5p,mmu_mi_r_497b,mmu_mi_r_499_5p,mmu_mi_r_503_5p,mmu_mi_r_504_3p,mmu_mi_r_5101,mmu_mi_r_5116,mmu_mi_r_5121,mmu_mi_r_5129_3p,mmu_mi_r_5130,mmu_mi_r_5131,mmu_mi_r_5132_5p,mmu_mi_r_5134_3p,mmu_mi_r_539_3p,mmu_mi_r_540_3p,mmu_mi_r_540_5p,mmu_mi_r_5616_3p,mmu_mi_r_5620_5p,mmu_mi_r_5621_5p,mmu_mi_r_5623_3p,mmu_mi_r_5624_3p,mmu_mi_r_5626_3p,mmu_mi_r_5626_5p,mmu_mi_r_568,mmu_mi_r_5709_5p,mmu_mi_r_590_3p,mmu_mi_r_592_3p,mmu_mi_r_598_3p,mmu_mi_r_598_5p,mmu_mi_r_599,mmu_mi_r_6238,mmu_mi_r_6244,mmu_mi_r_6335,mmu_mi_r_6336,mmu_mi_r_6337,mmu_mi_r_6342,mmu_mi_r_6346,mmu_mi_r_6349,mmu_mi_r_6353,mmu_mi_r_6359,mmu_mi_r_6360,mmu_mi_r_6362,mmu_mi_r_6366,mmu_mi_r_6369,mmu_mi_r_6370,mmu_mi_r_6371,mmu_mi_r_6373,mmu_mi_r_6385,mmu_mi_r_6386,mmu_mi_r_6389,mmu_mi_r_6391,mmu_mi_r_6396,mmu_mi_r_6403,mmu_mi_r_6406,mmu_mi_r_6408,mmu_mi_r_6481,mmu_mi_r_6537_5p,mmu_mi_r_6539,mmu_mi_r_6540_3p,mmu_mi_r_6540_5p,mmu_mi_r_6541,mmu_mi_r_6546_5p,mmu_mi_r_668_5p,mmu_mi_r_669a_3p_mmu_mi_r_669o_3p,mmu_mi_r_669b_3p,mmu_mi_r_669b_5p,mmu_mi_r_669c_5p,mmu_mi_r_669e_5p,mmu_mi_r_669g,mmu_mi_r_669j,mmu_mi_r_669l_3p,mmu_mi_r_669m_3p,mmu_mi_r_670_5p,mmu_mi_r_672_5p,mmu_mi_r_673_3p,mmu_mi_r_674_3p,mmu_mi_r_677_5p,mmu_mi_r_678,mmu_mi_r_686,mmu_mi_r_6896_3p,mmu_mi_r_6899_5p,mmu_mi_r_6901_3p,mmu_mi_r_6902_5p,mmu_mi_r_6904_3p,mmu_mi_r_6905_3p,mmu_mi_r_6905_5p,mmu_mi_r_6906_3p,mmu_mi_r_6907_5p,mmu_mi_r_6909_5p,mmu_mi_r_6910_3p,mmu_mi_r_6914_3p,mmu_mi_r_6914_5p,mmu_mi_r_6919_3p,mmu_mi_r_6921_3p,mmu_mi_r_6922_3p,mmu_mi_r_6925_3p,mmu_mi_r_6925_5p,mmu_mi_r_6927_3p,mmu_mi_r_6927_5p,mmu_mi_r_6928_5p,mmu_mi_r_693_3p,mmu_mi_r_6930_3p,mmu_mi_r_6935_3p,mmu_mi_r_6936_5p,mmu_mi_r_6938_3p,mmu_mi_r_6939_3p,mmu_mi_r_694,mmu_mi_r_6940_5p,mmu_mi_r_6941_3p,mmu_mi_r_6943_5p,mmu_mi_r_6944_3p,mmu_mi_r_6945_3p,mmu_mi_r_6945_5p,mmu_mi_r_6947_5p,mmu_mi_r_6949_3p,mmu_mi_r_6951_5p,mmu_mi_r_6952_3p,mmu_mi_r_6953_5p,mmu_mi_r_6954_5p,mmu_mi_r_6956_3p,mmu_mi_r_6957_5p,mmu_mi_r_6961_3p,mmu_mi_r_6962_5p,mmu_mi_r_6963_5p,mmu_mi_r_6964_3p,mmu_mi_r_6965_5p,mmu_mi_r_6966_5p,mmu_mi_r_6969_3p,mmu_mi_r_6970_5p,mmu_mi_r_6971_3p,mmu_mi_r_6972_3p,mmu_mi_r_6977_3p,mmu_mi_r_6978_3p,mmu_mi_r_6979_3p,mmu_mi_r_6982_5p,mmu_mi_r_6984_5p,mmu_mi_r_6985_5p,mmu_mi_r_6987_3p,mmu_mi_r_6988_3p,mmu_mi_r_6990_3p,mmu_mi_r_6991_3p,mmu_mi_r_6991_5p,mmu_mi_r_6992_3p,mmu_mi_r_6993_3p,mmu_mi_r_6995_3p,mmu_mi_r_6997_3p,mmu_mi_r_7007_3p,mmu_mi_r_7008_3p,mmu_mi_r_7010_3p,mmu_mi_r_7013_5p,mmu_mi_r_7017_5p,mmu_mi_r_7018_3p,mmu_mi_r_7019_5p,mmu_mi_r_702_3p,mmu_mi_r_7020_5p,mmu_mi_r_7021_3p,mmu_mi_r_7024_3p,mmu_mi_r_7026_3p,mmu_mi_r_7027_5p,mmu_mi_r_7033_5p,mmu_mi_r_7034_3p,mmu_mi_r_7037_5p,mmu_mi_r_7038_3p,mmu_mi_r_7040_5p,mmu_mi_r_7041_3p,mmu_mi_r_7043_5p,mmu_mi_r_7049_3p,mmu_mi_r_7049_5p,mmu_mi_r_7050_3p,mmu_mi_r_7051_3p,mmu_mi_r_7051_5p,mmu_mi_r_7053_5p,mmu_mi_r_7055_5p,mmu_mi_r_7057_3p,mmu_mi_r_7057_5p,mmu_mi_r_706,mmu_mi_r_7068_3p,mmu_mi_r_7073_3p,mmu_mi_r_7075_5p,mmu_mi_r_7078_3p,mmu_mi_r_7079_3p,mmu_mi_r_7080_3p,mmu_mi_r_7080_5p,mmu_mi_r_7081_5p,mmu_mi_r_7082_3p,mmu_mi_r_7083_3p,mmu_mi_r_7083_5p,mmu_mi_r_7085_5p,mmu_mi_r_7086_3p,mmu_mi_r_7089_3p,mmu_mi_r_709,mmu_mi_r_7090_5p,mmu_mi_r_7094b_2_5p,mmu_mi_r_710,mmu_mi_r_711,mmu_mi_r_7115_3p,mmu_mi_r_7116_3p,mmu_mi_r_7118_3p,mmu_mi_r_7119_3p,mmu_mi_r_7210_3p,mmu_mi_r_7212_3p,mmu_mi_r_7213_3p,mmu_mi_r_7216_3p,mmu_mi_r_7218_3p,mmu_mi_r_7221_5p,mmu_mi_r_7222_3p,mmu_mi_r_7223_3p,mmu_mi_r_7224_3p,mmu_mi_r_7225_5p,mmu_mi_r_7226_5p,mmu_mi_r_7227_5p,mmu_mi_r_7229_3p,mmu_mi_r_7230_5p,mmu_mi_r_7231_3p,mmu_mi_r_7233_3p,mmu_mi_r_7243_3p,mmu_mi_r_741_5p,mmu_mi_r_743a_3p,mmu_mi_r_743a_5p,mmu_mi_r_743b_3p,mmu_mi_r_7578,mmu_mi_r_758_3p,mmu_mi_r_760_5p,mmu_mi_r_7646_3p,mmu_mi_r_7647_3p,mmu_mi_r_7647_5p,mmu_mi_r_7652_3p,mmu_mi_r_7656_3p,mmu_mi_r_7658_5p,mmu_mi_r_7659_5p,mmu_mi_r_7660_5p,mmu_mi_r_7661_3p,mmu_mi_r_7661_5p,mmu_mi_r_7662_3p,mmu_mi_r_7664_3p,mmu_mi_r_7665_3p,mmu_mi_r_7665_5p,mmu_mi_r_7667_3p,mmu_mi_r_7668_3p,mmu_mi_r_7670_3p,mmu_mi_r_7673_3p,mmu_mi_r_7674_3p,mmu_mi_r_7675_5p,mmu_mi_r_7676_3p,mmu_mi_r_7677_5p,mmu_mi_r_7683_3p,mmu_mi_r_7684_3p,mmu_mi_r_7684_5p,mmu_mi_r_7687_5p,mmu_mi_r_7689_3p,mmu_mi_r_7b_3p,mmu_mi_r_802_5p,mmu_mi_r_8101,mmu_mi_r_8102,mmu_mi_r_8103,mmu_mi_r_8106,mmu_mi_r_8109,mmu_mi_r_8113,mmu_mi_r_8115,mmu_mi_r_8116,mmu_mi_r_8118,mmu_mi_r_871_3p,mmu_mi_r_875_3p,mmu_mi_r_876_5p,mmu_mi_r_877_3p,mmu_mi_r_879_5p,mmu_mi_r_880_3p,mmu_mi_r_880_5p,mmu_mi_r_881_3p,mmu_mi_r_883a_3p,mmu_mi_r_9768_5p,mmu_pi_r_000159_gb_dq539904_mus_musculus_2_73668844_73668871_plus,mmu_pi_r_000362_gb_dq540403_mus_musculus_6_3151474_3151503_plus,mmu_pi_r_000619_gb_dq540976_mus_musculus_17_39454691_39454717_plus,mmu_pi_r_000622_gb_dq540988_mus_musculus_2_5296560_5296587_minus,mmu_pi_r_000622_gb_dq540988_mus_musculus_3_5843428_5843455_plus,mmu_pi_r_000622_gb_dq540988_mus_musculus_x_112404287_112404314_minus,mmu_pi_r_000691_gb_dq541218_mus_musculus_11_74136081_74136106_plus,mmu_pi_r_000691_gb_dq541218_mus_musculus_8_126424875_126424900_minus,mmu_pi_r_000691_gb_dq541218_mus_musculus_8_126455417_126455442_minus,mmu_pi_r_000691_gb_dq541218_mus_musculus_8_126457090_126457115_minus,mmu_pi_r_000691_gb_dq541218_mus_musculus_8_126467244_126467269_minus,mmu_pi_r_000691_gb_dq541218_mus_musculus_8_126472331_126472356_minus,mmu_pi_r_000691_gb_dq541218_mus_musculus_8_126492702_126492727_minus,mmu_pi_r_000691_gb_dq541218_mus_musculus_8_126494409_126494434_minus,mmu_pi_r_002643_gb_dq547181_mus_musculus_3_5843705_5843732_plus,mmu_pi_r_003399_gb_dq549760_mus_musculus_5_108144856_108144884_plus,mmu_pi_r_005109_gb_dq555094_mus_musculus_5_146565258_146565289_plus,mmu_pi_r_011460_gb_dq688079_mus_musculus_9_67527113_67527141_minus,mmu_pi_r_016623_gb_dq695859_mus_musculus_9_54023526_54023555_minus,mmu_pi_r_017289_gb_dq696831_mus_musculus_6_128804704_128804734_plus,mmu_pi_r_020492_gb_dq701563_mus_musculus_11_108827972_108827997_minus,mmu_pi_r_020554_gb_dq701662_mus_musculus_2_92351039_92351069_plus,mmu_pi_r_023189_gb_dq705481_mus_musculus_16_18197850_18197871_minus,mmu_pi_r_034512_gb_dq721887_mus_musculus_14_22936178_22936204_plus,mmu_pi_r_037947_gb_dq726864_mus_musculus_9_54023523_54023552_minus,mmu_pi_r_038323_gb_pi_rna_t34_mus_musculus_14_18071278_18071308_plus,mmu_pi_r_038328_gb_pi_rna_t47_mus_musculus_17_39455055_39455084_plus,mmu_pi_r_038328_gb_pi_rna_t47_mus_musculus_19_13121850_13121879_plus,mmu_pi_r_038328_gb_pi_rna_t47_mus_musculus_6_3151091_3151120_plus
888.641	x888_641_A	mmu_let_7f_2_3p,mmu_mi_r_100_5p,mmu_mi_r_101c,mmu_mi_r_106b_3p,mmu_mi_r_1188_3p,mmu_mi_r_1191b_3p,mmu_mi_r_1195,mmu_mi_r_1197_5p,mmu_mi_r_122_5p,mmu_mi_r_1247_5p,mmu_mi_r_129b_5p,mmu_mi_r_130a_3p,mmu_mi_r_130b_5p,mmu_mi_r_134_3p,mmu_mi_r_146b_3p,mmu_mi_r_1839_3p,mmu_mi_r_184_3p,mmu_mi_r_187_3p,mmu_mi_r_188_5p,mmu_mi_r_1892,mmu_mi_r_1900,mmu_mi_r_1902,mmu_mi_r_192_5p,mmu_mi_r_1933_3p,mmu_mi_r_194_5p,mmu_mi_r_1942,mmu_mi_r_1948_3p,mmu_mi_r_1957a,mmu_mi_r_1958,mmu_mi_r_1981_3p,mmu_mi_r_1981_5p,mmu_mi_r_205_3p,mmu_mi_r_215_3p,mmu_mi_r_215_5p,mmu_mi_r_216c_5p,mmu_mi_r_218_2_3p,mmu_mi_r_224_3p,mmu_mi_r_26a_1_3p,mmu_mi_r_290a_5p,mmu_mi_r_290b_5p,mmu_mi_r_294_3p,mmu_mi_r_295_3p,mmu_mi_r_29a_3p,mmu_mi_r_29b_3p,mmu_mi_r_29c_3p,mmu_mi_r_300_5p,mmu_mi_r_301b_5p,mmu_mi_r_3057_3p,mmu_mi_r_3062_3p,mmu_mi_r_3063_5p,mmu_mi_r_3066_3p,mmu_mi_r_3067_5p,mmu_mi_r_3070_2_3p,mmu_mi_r_3070_3p,mmu_mi_r_3073b_3p,mmu_mi_r_3079_3p,mmu_mi_r_3079_5p,mmu_mi_r_3082_5p,mmu_mi_r_3085_3p,mmu_mi_r_3089_5p,mmu_mi_r_3091_5p,mmu_mi_r_3095_5p,mmu_mi_r_30c_1_3p,mmu_mi_r_3101_3p,mmu_mi_r_3102_3p,mmu_mi_r_3102_5p_2_5p,mmu_mi_r_3103_3p,mmu_mi_r_3106_3p,mmu_mi_r_3113_3p,mmu_mi_r_324_5p,mmu_mi_r_325_5p,mmu_mi_r_326_5p,mmu_mi_r_328_5p,mmu_mi_r_337_3p,mmu_mi_r_338_5p,mmu_mi_r_343,mmu_mi_r_344_5p,mmu_mi_r_344f_5p,mmu_mi_r_346_3p,mmu_mi_r_346_5p,mmu_mi_r_3471,mmu_mi_r_3473g,mmu_mi_r_3535,mmu_mi_r_3572_5p,mmu_mi_r_365_1_5p,mmu_mi_r_365_3p,mmu_mi_r_376b_3p,mmu_mi_r_380_3p,mmu_mi_r_383_5p,mmu_mi_r_3965,mmu_mi_r_409_5p,mmu_mi_r_412_3p,mmu_mi_r_421_5p,mmu_mi_r_429_3p,mmu_mi_r_434_3p,mmu_mi_r_449a_3p,mmu_mi_r_450a_5p,mmu_mi_r_451b,mmu_mi_r_465a_5p,mmu_mi_r_466c_5p,mmu_mi_r_466f_3p,mmu_mi_r_466g,mmu_mi_r_466k,mmu_mi_r_466m_5p_mmu_mi_r_669m_5p,mmu_mi_r_466q,mmu_mi_r_467a_3p,mmu_mi_r_467h,mmu_mi_r_470_5p,mmu_mi_r_483_5p,mmu_mi_r_485_3p,mmu_mi_r_485_5p,mmu_mi_r_486b_3p,mmu_mi_r_487b_3p,mmu_mi_r_491_3p,mmu_mi_r_496a_3p,mmu_mi_r_496a_5p,mmu_mi_r_505_3p,mmu_mi_r_5103,mmu_mi_r_5107_5p,mmu_mi_r_5127,mmu_mi_r_5132_5p,mmu_mi_r_532_5p,mmu_mi_r_540_5p,mmu_mi_r_5615_5p,mmu_mi_r_5622_5p,mmu_mi_r_5624_5p,mmu_mi_r_5625_5p,mmu_mi_r_582_5p,mmu_mi_r_590_3p,mmu_mi_r_6340,mmu_mi_r_6341,mmu_mi_r_6346,mmu_mi_r_6370,mmu_mi_r_6372,mmu_mi_r_6384,mmu_mi_r_6385,mmu_mi_r_6387,mmu_mi_r_6393,mmu_mi_r_6400,mmu_mi_r_6540_3p,mmu_mi_r_6540_5p,mmu_mi_r_664_5p,mmu_mi_r_668_3p,mmu_mi_r_668_5p,mmu_mi_r_669a_5p_mmu_mi_r_669p_5p,mmu_mi_r_669b_3p,mmu_mi_r_669g,mmu_mi_r_669h_5p,mmu_mi_r_673_5p,mmu_mi_r_674_3p,mmu_mi_r_677_3p,mmu_mi_r_683,mmu_mi_r_6896_3p,mmu_mi_r_6896_5p,mmu_mi_r_6897_5p,mmu_mi_r_6903_5p,mmu_mi_r_6905_5p,mmu_mi_r_6906_3p,mmu_mi_r_6907_5p,mmu_mi_r_6918_5p,mmu_mi_r_6919_3p,mmu_mi_r_692,mmu_mi_r_6920_3p,mmu_mi_r_6924_3p,mmu_mi_r_693_5p,mmu_mi_r_6930_3p,mmu_mi_r_694,mmu_mi_r_6943_3p,mmu_mi_r_6944_3p,mmu_mi_r_6953_3p,mmu_mi_r_6953_5p,mmu_mi_r_6954_3p,mmu_mi_r_6956_3p,mmu_mi_r_6959_3p,mmu_mi_r_6959_5p,mmu_mi_r_696,mmu_mi_r_6960_3p,mmu_mi_r_6962_3p,mmu_mi_r_6964_5p,mmu_mi_r_6965_3p,mmu_mi_r_6965_5p,mmu_mi_r_697,mmu_mi_r_6973a_3p,mmu_mi_r_6975_3p,mmu_mi_r_6978_5p,mmu_mi_r_6979_3p,mmu_mi_r_6983_3p,mmu_mi_r_6984_3p,mmu_mi_r_6988_5p,mmu_mi_r_6992_3p,mmu_mi_r_6992_5p,mmu_mi_r_6993_5p,mmu_mi_r_6994_3p,mmu_mi_r_6996_3p,mmu_mi_r_6999_5p,mmu_mi_r_7008_5p,mmu_mi_r_7009_3p,mmu_mi_r_7012_3p,mmu_mi_r_7012_5p,mmu_mi_r_7013_3p,mmu_mi_r_7022_3p,mmu_mi_r_7024_3p,mmu_mi_r_7025_3p,mmu_mi_r_7030_3p,mmu_mi_r_7030_5p,mmu_mi_r_7033_5p,mmu_mi_r_7036b_3p,mmu_mi_r_7038_3p,mmu_mi_r_7038_5p,mmu_mi_r_7043_3p,mmu_mi_r_7043_5p,mmu_mi_r_7048_3p,mmu_mi_r_7051_3p,mmu_mi_r_7051_5p,mmu_mi_r_7053_5p,mmu_mi_r_7059_3p,mmu_mi_r_7059_5p,mmu_mi_r_7065_5p,mmu_mi_r_7072_5p,mmu_mi_r_7074_3p,mmu_mi_r_7077_5p,mmu_mi_r_7078_3p,mmu_mi_r_7079_3p,mmu_mi_r_7079_5p,mmu_mi_r_7082_5p,mmu_mi_r_7085_5p,mmu_mi_r_7089_3p,mmu_mi_r_7092_3p,mmu_mi_r_7115_5p,mmu_mi_r_7117_3p,mmu_mi_r_719,mmu_mi_r_7211_3p,mmu_mi_r_7213_5p,mmu_mi_r_7214_3p,mmu_mi_r_7215_3p,mmu_mi_r_7219_3p,mmu_mi_r_7225_3p,mmu_mi_r_7229_3p,mmu_mi_r_7231_3p,mmu_mi_r_7232_5p,mmu_mi_r_7234_3p,mmu_mi_r_7234_5p,mmu_mi_r_7236_5p,mmu_mi_r_7237_5p,mmu_mi_r_7238_3p,mmu_mi_r_7238_5p,mmu_mi_r_7239_3p,mmu_mi_r_7243_5p,mmu_mi_r_742_3p,mmu_mi_r_743b_5p,mmu_mi_r_7578,mmu_mi_r_758_5p,mmu_mi_r_764_3p,mmu_mi_r_7648_3p,mmu_mi_r_7648_5p,mmu_mi_r_7651_3p,mmu_mi_r_7653_5p,mmu_mi_r_7654_5p,mmu_mi_r_7655_3p,mmu_mi_r_7655_5p,mmu_mi_r_7658_5p,mmu_mi_r_7660_5p,mmu_mi_r_7662_3p,mmu_mi_r_7665_5p,mmu_mi_r_7667_3p,mmu_mi_r_7667_5p,mmu_mi_r_7680_3p,mmu_mi_r_7684_5p,mmu_mi_r_7686_5p,mmu_mi_r_770_3p,mmu_mi_r_802_5p,mmu_mi_r_8092,mmu_mi_r_8099,mmu_mi_r_8103,mmu_mi_r_8105,mmu_mi_r_8106,mmu_mi_r_8110,mmu_mi_r_8111,mmu_mi_r_8115,mmu_mi_r_871_3p,mmu_mi_r_873a_3p,mmu_mi_r_873b,mmu_mi_r_876_5p,mmu_mi_r_881_3p,mmu_mi_r_881_5p,mmu_mi_r_883b_5p,mmu_mi_r_92a_2_5p,mmu_mi_r_96_3p,mmu_mi_r_9768_3p,mmu_mi_r_9769_3p,mmu_mi_r_99b_3p,mmu_pi_r_000620_gb_dq540981_mus_musculus_18_54824345_54824374_minus,mmu_pi_r_000620_gb_dq540981_mus_musculus_3_5843782_5843811_plus,mmu_pi_r_000639_gb_dq541113_mus_musculus_17_39455268_39455298_plus,mmu_pi_r_000691_gb_dq541218_mus_musculus_8_126462165_126462190_minus,mmu_pi_r_000691_gb_dq541218_mus_musculus_8_126485950_126485975_minus,mmu_pi_r_010309_gb_dq686298_mus_musculus_1_161177076_161177096_plus,mmu_pi_r_010565_gb_dq686723_mus_musculus_16_38309443_38309460_plus,mmu_pi_r_024456_gb_dq707344_mus_musculus_7_72990109_72990139_plus,mmu_pi_r_029416_gb_dq714514_mus_musculus_9_67542046_67542076_plus,mmu_pi_r_030303_gb_dq715776_mus_musculus_6_127764268_127764297_plus,mmu_pi_r_034249_gb_dq721513_mus_musculus_5_113579122_113579149_minus,mmu_pi_r_035552_gb_dq723401_mus_musculus_17_66117579_66117604_plus,mmu_pi_r_038323_gb_pi_rna_t34_mus_musculus_13_44880547_44880577_minus
1253.773	x1253_773_A	mmu_let_7a_1_3p_mmu_let_7c_2_3p,mmu_let_7a_2_3p,mmu_mi_r_106a_3p,mmu_mi_r_1191a,mmu_mi_r_1191b_5p,mmu_mi_r_1199_3p,mmu_mi_r_1199_5p,mmu_mi_r_122_3p,mmu_mi_r_1224_5p,mmu_mi_r_124_3p,mmu_mi_r_129_1_3p,mmu_mi_r_1291,mmu_mi_r_130b_5p,mmu_mi_r_134_5p,mmu_mi_r_136_5p,mmu_mi_r_138_2_3p,mmu_mi_r_142a_3p,mmu_mi_r_142a_5p,mmu_mi_r_142b,mmu_mi_r_146a_5p,mmu_mi_r_150_3p,mmu_mi_r_150_5p,mmu_mi_r_155_5p,mmu_mi_r_15a_3p,mmu_mi_r_181a_5p,mmu_mi_r_182_5p,mmu_mi_r_1843a_5p,mmu_mi_r_188_3p,mmu_mi_r_1896,mmu_mi_r_18b_5p,mmu_mi_r_192_3p,mmu_mi_r_1929_3p,mmu_mi_r_1932,mmu_mi_r_1934_3p,mmu_mi_r_1943_5p,mmu_mi_r_1946b,mmu_mi_r_1948_5p,mmu_mi_r_1960,mmu_mi_r_1968_3p,mmu_mi_r_196a_5p,mmu_mi_r_199b_5p,mmu_mi_r_200c_5p,mmu_mi_r_203_5p,mmu_mi_r_204_3p,mmu_mi_r_210_5p,mmu_mi_r_211_3p,mmu_mi_r_2137,mmu_mi_r_218_1_3p,mmu_mi_r_23a_3p,mmu_mi_r_23b_3p,mmu_mi_r_24_3p,mmu_mi_r_2861,mmu_mi_r_290a_5p,mmu_mi_r_290b_5p,mmu_mi_r_292b_3p,mmu_mi_r_296_3p,mmu_mi_r_297a_5p,mmu_mi_r_300_3p,mmu_mi_r_3060_5p,mmu_mi_r_3068_5p,mmu_mi_r_3069_3p,mmu_mi_r_3070_3p,mmu_mi_r_3073b_3p,mmu_mi_r_3079_3p,mmu_mi_r_3080_3p,mmu_mi_r_3090_5p,mmu_mi_r_3091_5p,mmu_mi_r_3099_3p,mmu_mi_r_30c_1_3p,mmu_mi_r_30d_3p,mmu_mi_r_3101_5p,mmu_mi_r_3154,mmu_mi_r_322_3p,mmu_mi_r_322_5p,mmu_mi_r_326_3p,mmu_mi_r_326_5p,mmu_mi_r_342_3p,mmu_mi_r_344f_5p,mmu_mi_r_3470a,mmu_mi_r_3473b,mmu_mi_r_3473g,mmu_mi_r_351_5p,mmu_mi_r_3535,mmu_mi_r_3572_5p,mmu_mi_r_362_3p,mmu_mi_r_3620_5p,mmu_mi_r_367_3p,mmu_mi_r_369_5p,mmu_mi_r_376b_3p,mmu_mi_r_380_5p,mmu_mi_r_383_3p,mmu_mi_r_3966,mmu_mi_r_3968,mmu_mi_r_423_3p,mmu_mi_r_433_3p,mmu_mi_r_449a_5p,mmu_mi_r_449c_5p,mmu_mi_r_450b_5p,mmu_mi_r_463_3p,mmu_mi_r_466b_3p_mmu_mi_r_466c_3p_mmu_mi_r_466p_3p,mmu_mi_r_467d_5p,mmu_mi_r_467e_5p,mmu_mi_r_471_5p,mmu_mi_r_487b_5p,mmu_mi_r_488_5p,mmu_mi_r_489_5p,mmu_mi_r_490_3p,mmu_mi_r_490_5p,mmu_mi_r_497a_3p,mmu_mi_r_497b,mmu_mi_r_500_3p,mmu_mi_r_500_5p,mmu_mi_r_503_3p,mmu_mi_r_5100,mmu_mi_r_5103,mmu_mi_r_5110,mmu_mi_r_5112,mmu_mi_r_5118,mmu_mi_r_5120,mmu_mi_r_5134_3p,mmu_mi_r_542_3p,mmu_mi_r_551b_5p,mmu_mi_r_5621_5p,mmu_mi_r_5622_3p,mmu_mi_r_5624_3p,mmu_mi_r_5624_5p,mmu_mi_r_5627_3p,mmu_mi_r_598_5p,mmu_mi_r_6236,mmu_mi_r_6237,mmu_mi_r_6239,mmu_mi_r_6241,mmu_mi_r_6345,mmu_mi_r_6346,mmu_mi_r_6350,mmu_mi_r_6356,mmu_mi_r_6359,mmu_mi_r_6366,mmu_mi_r_6370,mmu_mi_r_6373,mmu_mi_r_6379,mmu_mi_r_6389,mmu_mi_r_6391,mmu_mi_r_6396,mmu_mi_r_6400,mmu_mi_r_6404,mmu_mi_r_6407,mmu_mi_r_6415,mmu_mi_r_667_5p,mmu_mi_r_669c_5p,mmu_mi_r_669e_3p,mmu_mi_r_669h_5p,mmu_mi_r_671_5p,mmu_mi_r_672_5p,mmu_mi_r_675_5p,mmu_mi_r_676_5p,mmu_mi_r_679_3p,mmu_mi_r_680,mmu_mi_r_6897_5p,mmu_mi_r_6898_3p,mmu_mi_r_6905_5p,mmu_mi_r_6909_5p,mmu_mi_r_6912_3p,mmu_mi_r_6919_3p,mmu_mi_r_6927_3p,mmu_mi_r_6934_3p,mmu_mi_r_6935_3p,mmu_mi_r_6938_3p,mmu_mi_r_6945_3p,mmu_mi_r_695,mmu_mi_r_6951_5p,mmu_mi_r_6952_5p,mmu_mi_r_6955_5p,mmu_mi_r_696,mmu_mi_r_6961_3p,mmu_mi_r_6970_3p,mmu_mi_r_6974_5p,mmu_mi_r_6976_3p,mmu_mi_r_6976_5p,mmu_mi_r_6977_3p,mmu_mi_r_6980_3p,mmu_mi_r_6981_5p,mmu_mi_r_6982_3p,mmu_mi_r_6982_5p,mmu_mi_r_6983_3p,mmu_mi_r_6984_5p,mmu_mi_r_6987_5p,mmu_mi_r_6988_3p,mmu_mi_r_6990_3p,mmu_mi_r_6992_3p,mmu_mi_r_6994_5p,mmu_mi_r_6995_3p,mmu_mi_r_6995_5p,mmu_mi_r_7001_3p,mmu_mi_r_7004_3p,mmu_mi_r_7014_3p,mmu_mi_r_7021_3p,mmu_mi_r_7022_3p,mmu_mi_r_7023_5p,mmu_mi_r_7024_3p,mmu_mi_r_7027_5p,mmu_mi_r_7035_5p,mmu_mi_r_7041_5p,mmu_mi_r_7043_5p,mmu_mi_r_7045_3p,mmu_mi_r_7048_5p,mmu_mi_r_7050_3p,mmu_mi_r_7056_5p,mmu_mi_r_7057_5p,mmu_mi_r_7060_3p,mmu_mi_r_7060_5p,mmu_mi_r_7061_5p,mmu_mi_r_7068_3p,mmu_mi_r_707,mmu_mi_r_7076_3p,mmu_mi_r_7079_5p,mmu_mi_r_7080_5p,mmu_mi_r_7085_5p,mmu_mi_r_7089_3p,mmu_mi_r_7090_5p,mmu_mi_r_7092_5p,mmu_mi_r_7116_5p,mmu_mi_r_7118_3p,mmu_mi_r_718,mmu_mi_r_7212_3p,mmu_mi_r_7221_5p,mmu_mi_r_7225_5p,mmu_mi_r_7227_3p,mmu_mi_r_7235_5p,mmu_mi_r_7239_5p,mmu_mi_r_7240_3p,mmu_mi_r_760_3p,mmu_mi_r_7648_5p,mmu_mi_r_7654_5p,mmu_mi_r_7661_3p,mmu_mi_r_7661_5p,mmu_mi_r_7662_3p,mmu_mi_r_7668_3p,mmu_mi_r_7669_3p,mmu_mi_r_7674_3p,mmu_mi_r_7678_3p,mmu_mi_r_7678_5p,mmu_mi_r_7679_5p,mmu_mi_r_7680_5p,mmu_mi_r_7685_3p,mmu_mi_r_802_3p,mmu_mi_r_8097,mmu_mi_r_8101,mmu_mi_r_8110,mmu_mi_r_8115,mmu_mi_r_8120,mmu_mi_r_872_5p,mmu_mi_r_875_5p,mmu_mi_r_877_5p,mmu_mi_r_879_5p,mmu_mi_r_881_5p,mmu_mi_r_883a_5p,mmu_mi_r_9_5p,mmu_mi_r_92a_1_5p,mmu_mi_r_96_5p,mmu_mi_r_98_3p,mmu_mi_r_99a_3p,mmu_pi_r_000366_gb_dq540412_mus_musculus_17_25602713_25602739_plus,mmu_pi_r_000366_gb_dq540412_mus_musculus_6_86369552_86369578_plus,mmu_pi_r_000414_gb_dq540526_mus_musculus_16_18446800_18446827_minus,mmu_pi_r_000691_gb_dq541218_mus_musculus_11_74136081_74136106_plus,mmu_pi_r_000958_gb_dq541851_mus_musculus_17_39456228_39456254_plus,mmu_pi_r_004374_gb_dq552696_mus_musculus_9_110126452_110126481_plus,mmu_pi_r_004567_gb_dq553409_mus_musculus_17_39455256_39455282_plus,mmu_pi_r_006854_gb_dq562105_mus_musculus_12_59958363_59958391_plus,mmu_pi_r_011460_gb_dq688079_mus_musculus_9_67527113_67527141_minus,mmu_pi_r_021807_gb_dq703459_mus_musculus_5_115112996_115113025_minus,mmu_pi_r_027673_gb_dq711996_mus_musculus_11_106317081_106317102_plus,mmu_pi_r_030152_gb_dq715561_mus_musculus_18_67167521_67167549_minus,mmu_pi_r_032974_gb_dq719597_mus_musculus_4_130021751_130021778_plus,mmu_pi_r_036747_gb_dq725127_mus_musculus_10_62058989_62059017_plus,mmu_pi_r_038328_gb_pi_rna_t47_mus_musculus_6_3151091_3151120_plus
1281.79	x1281_79_A	mmu_let_7a_2_3p,mmu_let_7b_3p,mmu_let_7d_5p,mmu_let_7e_5p,mmu_let_7f_1_3p,mmu_let_7i_5p,mmu_let_7j,mmu_let_7k,mmu_mi_r_100_3p,mmu_mi_r_106a_3p,mmu_mi_r_106a_5p,mmu_mi_r_106b_3p,mmu_mi_r_106b_5p,mmu_mi_r_1191a,mmu_mi_r_1195,mmu_mi_r_1198_5p,mmu_mi_r_1199_3p,mmu_mi_r_1224_5p,mmu_mi_r_124_5p,mmu_mi_r_1247_5p,mmu_mi_r_129_1_3p,mmu_mi_r_135a_5p,mmu_mi_r_138_5p,mmu_mi_r_140_3p,mmu_mi_r_144_3p,mmu_mi_r_145b,mmu_mi_r_154_5p,mmu_mi_r_15a_3p,mmu_mi_r_15b_3p,mmu_mi_r_15b_5p,mmu_mi_r_16_2_3p,mmu_mi_r_16_5p,mmu_mi_r_17_3p,mmu_mi_r_17_5p,mmu_mi_r_181b_2_3p,mmu_mi_r_181c_5p,mmu_mi_r_181d_5p,mmu_mi_r_1843a_5p,mmu_mi_r_185_3p,mmu_mi_r_186_5p,mmu_mi_r_1894_5p,mmu_mi_r_1897_5p,mmu_mi_r_1898,mmu_mi_r_1899,mmu_mi_r_18a_3p,mmu_mi_r_18b_5p,mmu_mi_r_1930_3p,mmu_mi_r_1931,mmu_mi_r_1941_5p,mmu_mi_r_1942,mmu_mi_r_1946b,mmu_mi_r_1952,mmu_mi_r_1955_3p,mmu_mi_r_1958,mmu_mi_r_195a_5p,mmu_mi_r_1961,mmu_mi_r_1982_3p,mmu_mi_r_199a_5p,mmu_mi_r_200a_5p,mmu_mi_r_202_3p,mmu_mi_r_204_5p,mmu_mi_r_207,mmu_mi_r_20b_5p,mmu_mi_r_210_5p,mmu_mi_r_211_5p,mmu_mi_r_2137,mmu_mi_r_217_3p,mmu_mi_r_219c_3p,mmu_mi_r_221_3p,mmu_mi_r_222_3p,mmu_mi_r_222_5p,mmu_mi_r_26a_1_3p,mmu_mi_r_26b_3p,mmu_mi_r_27a_5p,mmu_mi_r_28c,mmu_mi_r_290a_3p,mmu_mi_r_290b_3p,mmu_mi_r_291a_3p,mmu_mi_r_293_5p,mmu_mi_r_295_3p,mmu_mi_r_297a_3p_mmu_mi_r_297b_3p_mmu_mi_r_297c_3p,mmu_mi_r_298_3p,mmu_mi_r_299b_3p,mmu_mi_r_29b_1_5p,mmu_mi_r_300_5p,mmu_mi_r_301a_5p,mmu_mi_r_301b_3p,mmu_mi_r_302a_5p,mmu_mi_r_3057_3p,mmu_mi_r_3057_5p,mmu_mi_r_3058_5p,mmu_mi_r_3059_3p,mmu_mi_r_3061_5p,mmu_mi_r_3070_3p,mmu_mi_r_3071_3p,mmu_mi_r_3072_3p,mmu_mi_r_3077_3p,mmu_mi_r_3078_5p,mmu_mi_r_3080_3p,mmu_mi_r_3082_5p,mmu_mi_r_3084_3p,mmu_mi_r_3085_3p,mmu_mi_r_3087_5p,mmu_mi_r_3089_5p,mmu_mi_r_3090_3p,mmu_mi_r_3092_3p,mmu_mi_r_3097_3p,mmu_mi_r_3098_5p,mmu_mi_r_30f,mmu_mi_r_3100_3p,mmu_mi_r_3102_5p,mmu_mi_r_3105_3p,mmu_mi_r_3105_5p,mmu_mi_r_3109_5p,mmu_mi_r_324_3p,mmu_mi_r_324_5p,mmu_mi_r_325_5p,mmu_mi_r_328_3p,mmu_mi_r_331_5p,mmu_mi_r_344_3p,mmu_mi_r_344d_3_5p,mmu_mi_r_344f_3p,mmu_mi_r_344f_5p,mmu_mi_r_344i,mmu_mi_r_3475_3p,mmu_mi_r_3475_5p,mmu_mi_r_34a_3p,mmu_mi_r_34c_3p,mmu_mi_r_3544_3p,mmu_mi_r_3547_3p,mmu_mi_r_3552,mmu_mi_r_3572_3p,mmu_mi_r_361_3p,mmu_mi_r_376c_5p,mmu_mi_r_379_5p,mmu_mi_r_381_3p,mmu_mi_r_382_3p,mmu_mi_r_3964,mmu_mi_r_3966,mmu_mi_r_3969,mmu_mi_r_3970,mmu_mi_r_421_3p,mmu_mi_r_423_5p,mmu_mi_r_449a_3p,mmu_mi_r_449a_5p,mmu_mi_r_451a,mmu_mi_r_453,mmu_mi_r_465d_3p,mmu_mi_r_466a_3p_mmu_mi_r_466e_3p,mmu_mi_r_466k,mmu_mi_r_466l_3p,mmu_mi_r_467b_3p,mmu_mi_r_485_5p,mmu_mi_r_486a_3p,mmu_mi_r_486a_5p,mmu_mi_r_486b_3p,mmu_mi_r_486b_5p,mmu_mi_r_491_5p,mmu_mi_r_495_5p,mmu_mi_r_5100,mmu_mi_r_5106,mmu_mi_r_5120,mmu_mi_r_5130,mmu_mi_r_5131,mmu_mi_r_541_5p,mmu_mi_r_5617_3p,mmu_mi_r_5624_3p,mmu_mi_r_5709_3p,mmu_mi_r_590_3p,mmu_mi_r_598_5p,mmu_mi_r_6337,mmu_mi_r_6340,mmu_mi_r_6344,mmu_mi_r_6345,mmu_mi_r_6349,mmu_mi_r_6351,mmu_mi_r_6358,mmu_mi_r_6360,mmu_mi_r_6364,mmu_mi_r_6365,mmu_mi_r_6369,mmu_mi_r_6371,mmu_mi_r_6386,mmu_mi_r_6392_5p,mmu_mi_r_6396,mmu_mi_r_6398,mmu_mi_r_6403,mmu_mi_r_6404,mmu_mi_r_6408,mmu_mi_r_6409,mmu_mi_r_6411,mmu_mi_r_6412,mmu_mi_r_6414,mmu_mi_r_6415,mmu_mi_r_6419,mmu_mi_r_652_3p,mmu_mi_r_6537_3p,mmu_mi_r_668_3p,mmu_mi_r_669c_3p,mmu_mi_r_669e_5p,mmu_mi_r_669g,mmu_mi_r_669h_3p,mmu_mi_r_672_3p,mmu_mi_r_673_5p,mmu_mi_r_674_5p,mmu_mi_r_675_5p,mmu_mi_r_676_3p,mmu_mi_r_687,mmu_mi_r_6905_3p,mmu_mi_r_6908_5p,mmu_mi_r_6910_3p,mmu_mi_r_6914_5p,mmu_mi_r_6916_3p,mmu_mi_r_6917_3p,mmu_mi_r_6919_3p,mmu_mi_r_6921_3p,mmu_mi_r_6925_3p,mmu_mi_r_6925_5p,mmu_mi_r_6928_5p,mmu_mi_r_6930_3p,mmu_mi_r_6933_3p,mmu_mi_r_6934_3p,mmu_mi_r_6937_3p,mmu_mi_r_6941_5p,mmu_mi_r_6943_5p,mmu_mi_r_6952_3p,mmu_mi_r_6954_3p,mmu_mi_r_6955_3p,mmu_mi_r_6960_3p,mmu_mi_r_6962_3p,mmu_mi_r_6962_5p,mmu_mi_r_6970_5p,mmu_mi_r_6979_3p,mmu_mi_r_698_5p,mmu_mi_r_6982_5p,mmu_mi_r_6984_5p,mmu_mi_r_6986_3p,mmu_mi_r_6994_3p,mmu_mi_r_7000_5p,mmu_mi_r_7005_5p,mmu_mi_r_7018_3p,mmu_mi_r_7021_5p,mmu_mi_r_7023_3p,mmu_mi_r_7025_3p,mmu_mi_r_7033_3p,mmu_mi_r_7034_3p,mmu_mi_r_7035_3p,mmu_mi_r_7036a_5p,mmu_mi_r_7038_5p,mmu_mi_r_7051_5p,mmu_mi_r_7054_5p,mmu_mi_r_7057_5p,mmu_mi_r_7060_5p,mmu_mi_r_7065_3p,mmu_mi_r_7067_3p,mmu_mi_r_7070_3p,mmu_mi_r_7073_5p,mmu_mi_r_7074_3p,mmu_mi_r_7075_5p,mmu_mi_r_7076_5p,mmu_mi_r_7077_3p,mmu_mi_r_7079_5p,mmu_mi_r_708_3p,mmu_mi_r_7091_5p,mmu_mi_r_7094_3p,mmu_mi_r_7117_5p,mmu_mi_r_712_5p,mmu_mi_r_717,mmu_mi_r_7218_3p,mmu_mi_r_7221_3p,mmu_mi_r_7223_5p,mmu_mi_r_7237_5p,mmu_mi_r_7239_5p,mmu_mi_r_760_3p,mmu_mi_r_7651_3p,mmu_mi_r_7652_3p,mmu_mi_r_7655_3p,mmu_mi_r_7657_5p,mmu_mi_r_7658_5p,mmu_mi_r_7663_5p,mmu_mi_r_7666_5p,mmu_mi_r_7676_3p,mmu_mi_r_7677_3p,mmu_mi_r_7678_3p,mmu_mi_r_7680_5p,mmu_mi_r_7687_5p,mmu_mi_r_7688_5p,mmu_mi_r_770_3p,mmu_mi_r_8092,mmu_mi_r_8106,mmu_mi_r_8107,mmu_mi_r_8108,mmu_mi_r_8110,mmu_mi_r_8119,mmu_mi_r_871_3p,mmu_mi_r_871_5p,mmu_mi_r_881_3p,mmu_mi_r_883a_3p,mmu_mi_r_92a_3p,mmu_mi_r_92b_3p,mmu_mi_r_93_3p,mmu_mi_r_93_5p,mmu_mi_r_9768_5p,mmu_mi_r_98_5p,mmu_pi_r_000619_gb_dq540976_mus_musculus_17_39454691_39454717_plus,mmu_pi_r_001570_gb_dq543701_mus_musculus_3_5843412_5843441_plus,mmu_pi_r_002728_gb_dq547492_mus_musculus_2_150846558_150846586_plus,mmu_pi_r_023189_gb_dq705481_mus_musculus_16_18197850_18197871_minus,mmu_pi_r_023366_gb_dq705744_mus_musculus_7_73687678_73687707_minus,mmu_pi_r_025576_gb_dq708952_mus_musculus_x_6405415_6405436_minus,mmu_pi_r_028975_gb_dq713872_mus_musculus_x_6405378_6405399_minus,mmu_pi_r_032865_gb_dq719430_mus_musculus_2_116876592_116876614_plus,mmu_pi_r_038328_gb_pi_rna_t47_mus_musculus_17_39455055_39455084_plus,mmu_pi_r_038328_gb_pi_rna_t47_mus_musculus_19_13121850_13121879_plus,mmu_pi_r_038649_gb_pi_rna_1701_mus_musculus_17_27070232_27070255_plus
1544.847	x1544_847_A	mmu_let_7g_3p,mmu_mi_r_103_2_5p,mmu_mi_r_106a_3p,mmu_mi_r_10a_3p,mmu_mi_r_10b_5p,mmu_mi_r_1193_5p,mmu_mi_r_1194,mmu_mi_r_1197_5p,mmu_mi_r_122_3p,mmu_mi_r_122_5p,mmu_mi_r_124_3p,mmu_mi_r_1258_5p,mmu_mi_r_129_1_3p,mmu_mi_r_129_5p,mmu_mi_r_137_5p,mmu_mi_r_138_1_3p,mmu_mi_r_139_3p,mmu_mi_r_146a_3p,mmu_mi_r_146a_5p,mmu_mi_r_150_3p,mmu_mi_r_153_5p,mmu_mi_r_154_5p,mmu_mi_r_155_5p,mmu_mi_r_182_5p,mmu_mi_r_183_5p,mmu_mi_r_1843a_3p,mmu_mi_r_1894_5p,mmu_mi_r_1898,mmu_mi_r_1906,mmu_mi_r_190a_5p,mmu_mi_r_1912_5p,mmu_mi_r_192_3p,mmu_mi_r_192_5p,mmu_mi_r_193a_3p,mmu_mi_r_194_1_3p,mmu_mi_r_194_5p,mmu_mi_r_1941_3p,mmu_mi_r_1941_5p,mmu_mi_r_1943_5p,mmu_mi_r_1952,mmu_mi_r_1966_5p,mmu_mi_r_200a_3p,mmu_mi_r_200b_3p,mmu_mi_r_200b_5p,mmu_mi_r_203_3p,mmu_mi_r_205_5p,mmu_mi_r_20a_3p,mmu_mi_r_210_5p,mmu_mi_r_211_3p,mmu_mi_r_212_3p,mmu_mi_r_212_5p,mmu_mi_r_2136,mmu_mi_r_215_3p,mmu_mi_r_215_5p,mmu_mi_r_217_5p,mmu_mi_r_219c_3p,mmu_mi_r_219c_5p,mmu_mi_r_24_1_5p,mmu_mi_r_26a_1_3p,mmu_mi_r_28a_3p,mmu_mi_r_28c,mmu_mi_r_290a_3p,mmu_mi_r_292a_5p,mmu_mi_r_29c_3p,mmu_mi_r_300_5p,mmu_mi_r_301a_5p,mmu_mi_r_302a_3p,mmu_mi_r_3069_5p,mmu_mi_r_3070_5p,mmu_mi_r_3074_2_3p,mmu_mi_r_3076_3p,mmu_mi_r_3078_3p,mmu_mi_r_3081_5p,mmu_mi_r_3082_3p,mmu_mi_r_3086_3p,mmu_mi_r_3088_5p,mmu_mi_r_3089_3p,mmu_mi_r_3089_5p,mmu_mi_r_3093_3p,mmu_mi_r_3105_5p,mmu_mi_r_331_3p,mmu_mi_r_338_3p,mmu_mi_r_341_5p,mmu_mi_r_3473c,mmu_mi_r_34a_3p,mmu_mi_r_34b_3p,mmu_mi_r_34c_3p,mmu_mi_r_3547_3p,mmu_mi_r_3618_3p,mmu_mi_r_3620_3p,mmu_mi_r_367_3p,mmu_mi_r_370_3p,mmu_mi_r_375_3p,mmu_mi_r_376a_3p,mmu_mi_r_3962,mmu_mi_r_421_5p,mmu_mi_r_429_3p,mmu_mi_r_431_3p,mmu_mi_r_452_5p,mmu_mi_r_465d_3p,mmu_mi_r_466l_3p,mmu_mi_r_466m_3p,mmu_mi_r_470_5p,mmu_mi_r_471_5p,mmu_mi_r_496a_3p,mmu_mi_r_503_5p,mmu_mi_r_509_3p,mmu_mi_r_509_5p,mmu_mi_r_5100,mmu_mi_r_511_5p,mmu_mi_r_5120,mmu_mi_r_5124a,mmu_mi_r_5124b,mmu_mi_r_543_3p,mmu_mi_r_5621_5p,mmu_mi_r_5626_3p,mmu_mi_r_5626_5p,mmu_mi_r_582_3p,mmu_mi_r_592_3p,mmu_mi_r_598_3p,mmu_mi_r_6236,mmu_mi_r_6241,mmu_mi_r_6335,mmu_mi_r_6341,mmu_mi_r_6342,mmu_mi_r_6349,mmu_mi_r_6352,mmu_mi_r_6357,mmu_mi_r_6360,mmu_mi_r_6361,mmu_mi_r_6363,mmu_mi_r_6369,mmu_mi_r_6371,mmu_mi_r_6381,mmu_mi_r_6394,mmu_mi_r_6403,mmu_mi_r_6406,mmu_mi_r_6418_3p,mmu_mi_r_6516_5p,mmu_mi_r_6546_5p,mmu_mi_r_667_5p,mmu_mi_r_668_3p,mmu_mi_r_668_5p,mmu_mi_r_669c_5p,mmu_mi_r_669f_5p,mmu_mi_r_669i,mmu_mi_r_669n,mmu_mi_r_670_5p,mmu_mi_r_671_3p,mmu_mi_r_673_5p,mmu_mi_r_6897_3p,mmu_mi_r_6898_5p,mmu_mi_r_6899_3p,mmu_mi_r_6902_5p,mmu_mi_r_6904_3p,mmu_mi_r_6907_5p,mmu_mi_r_691,mmu_mi_r_6910_5p,mmu_mi_r_6916_3p,mmu_mi_r_6918_3p,mmu_mi_r_6918_5p,mmu_mi_r_6919_5p,mmu_mi_r_692,mmu_mi_r_6920_5p,mmu_mi_r_6927_5p,mmu_mi_r_6929_5p,mmu_mi_r_6936_5p,mmu_mi_r_6938_5p,mmu_mi_r_6940_3p,mmu_mi_r_6940_5p,mmu_mi_r_6941_3p,mmu_mi_r_6941_5p,mmu_mi_r_6946_5p,mmu_mi_r_6948_5p,mmu_mi_r_6951_5p,mmu_mi_r_6954_5p,mmu_mi_r_6955_5p,mmu_mi_r_6966_3p,mmu_mi_r_6966_5p,mmu_mi_r_6969_5p,mmu_mi_r_6970_5p,mmu_mi_r_6972_3p,mmu_mi_r_6973b_3p,mmu_mi_r_6973b_5p,mmu_mi_r_6976_3p,mmu_mi_r_6979_5p,mmu_mi_r_6980_5p,mmu_mi_r_6984_3p,mmu_mi_r_6986_5p,mmu_mi_r_6991_3p,mmu_mi_r_700_5p,mmu_mi_r_7000_5p,mmu_mi_r_7002_5p,mmu_mi_r_7006_5p,mmu_mi_r_7007_5p,mmu_mi_r_7016_3p,mmu_mi_r_7019_3p,mmu_mi_r_7021_3p,mmu_mi_r_7021_5p,mmu_mi_r_7024_5p,mmu_mi_r_7030_3p,mmu_mi_r_7050_3p,mmu_mi_r_7051_3p,mmu_mi_r_7058_3p,mmu_mi_r_7059_3p,mmu_mi_r_7060_3p,mmu_mi_r_7061_5p,mmu_mi_r_7062_5p,mmu_mi_r_7064_5p,mmu_mi_r_7065_3p,mmu_mi_r_7069_5p,mmu_mi_r_7076_5p,mmu_mi_r_7077_3p,mmu_mi_r_708_5p,mmu_mi_r_7084_5p,mmu_mi_r_7085_5p,mmu_mi_r_7089_5p,mmu_mi_r_709,mmu_mi_r_7090_3p,mmu_mi_r_7092_5p,mmu_mi_r_7094_3p,mmu_mi_r_7117_5p,mmu_mi_r_7119_3p,mmu_mi_r_7210_5p,mmu_mi_r_7211_3p,mmu_mi_r_7213_5p,mmu_mi_r_7216_5p,mmu_mi_r_7221_3p,mmu_mi_r_7221_5p,mmu_mi_r_7226_5p,mmu_mi_r_7230_3p,mmu_mi_r_7230_5p,mmu_mi_r_7235_5p,mmu_mi_r_7237_5p,mmu_mi_r_758_3p,mmu_mi_r_758_5p,mmu_mi_r_7648_5p,mmu_mi_r_7649_3p,mmu_mi_r_7650_5p,mmu_mi_r_7660_5p,mmu_mi_r_7669_3p,mmu_mi_r_7670_3p,mmu_mi_r_7674_3p,mmu_mi_r_7681_3p,mmu_mi_r_7685_3p,mmu_mi_r_770_3p,mmu_mi_r_802_5p,mmu_mi_r_8090,mmu_mi_r_8091,mmu_mi_r_8094,mmu_mi_r_8100,mmu_mi_r_8118,mmu_mi_r_871_3p,mmu_mi_r_873a_5p,mmu_mi_r_875_3p,mmu_mi_r_876_3p,mmu_mi_r_880_5p,mmu_mi_r_9_3p,mmu_mi_r_92b_5p,mmu_mi_r_96_3p,mmu_mi_r_99a_3p,mmu_pi_r_000620_gb_dq540981_mus_musculus_18_54824345_54824374_minus,mmu_pi_r_000620_gb_dq540981_mus_musculus_3_5843782_5843811_plus,mmu_pi_r_000622_gb_dq540988_mus_musculus_18_54824707_54824734_minus,mmu_pi_r_000622_gb_dq540988_mus_musculus_3_5843428_5843455_plus,mmu_pi_r_001570_gb_dq543701_mus_musculus_3_5843412_5843441_plus,mmu_pi_r_004374_gb_dq552696_mus_musculus_18_85832427_85832456_minus,mmu_pi_r_009321_gb_dq684704_mus_musculus_7_72988380_72988411_plus,mmu_pi_r_010565_gb_dq686723_mus_musculus_16_38309443_38309460_plus,mmu_pi_r_012641_gb_dq689910_mus_musculus_17_27048950_27048977_minus,mmu_pi_r_017289_gb_dq696831_mus_musculus_6_128804704_128804734_plus,mmu_pi_r_028903_gb_dq713755_mus_musculus_1_109786123_109786152_minus,mmu_pi_r_029303_gb_dq714361_mus_musculus_10_18490237_18490267_minus
1544.867	x1544_867_A	mmu_let_7a_1_3p_mmu_let_7c_2_3p,mmu_let_7b_3p,mmu_let_7c_1_3p,mmu_let_7c_5p,mmu_let_7d_5p,mmu_let_7e_5p,mmu_let_7f_2_3p,mmu_let_7i_5p,mmu_let_7j,mmu_let_7k,mmu_mi_r_100_5p,mmu_mi_r_101a_3p,mmu_mi_r_101a_5p,mmu_mi_r_101b_3p,mmu_mi_r_101c,mmu_mi_r_103_2_5p,mmu_mi_r_103_3p,mmu_mi_r_106a_3p,mmu_mi_r_106b_3p,mmu_mi_r_107_3p,mmu_mi_r_10a_3p,mmu_mi_r_1188_3p,mmu_mi_r_1191a,mmu_mi_r_1191b_3p,mmu_mi_r_1191b_5p,mmu_mi_r_1195,mmu_mi_r_1197_5p,mmu_mi_r_1198_5p,mmu_mi_r_1199_5p,mmu_mi_r_122_5p,mmu_mi_r_1231_3p,mmu_mi_r_124_3p,mmu_mi_r_1247_3p,mmu_mi_r_1247_5p,mmu_mi_r_1251_5p,mmu_mi_r_1258_3p,mmu_mi_r_1258_5p,mmu_mi_r_125b_1_3p,mmu_mi_r_1264_5p,mmu_mi_r_127_3p,mmu_mi_r_129_1_3p,mmu_mi_r_1291,mmu_mi_r_1298_3p,mmu_mi_r_129b_5p,mmu_mi_r_1306_3p,mmu_mi_r_130a_3p,mmu_mi_r_130b_5p,mmu_mi_r_130c,mmu_mi_r_134_3p,mmu_mi_r_135b_5p,mmu_mi_r_140_3p,mmu_mi_r_141_3p,mmu_mi_r_143_3p,mmu_mi_r_144_5p,mmu_mi_r_145a_3p,mmu_mi_r_146b_3p,mmu_mi_r_147_5p,mmu_mi_r_148a_5p,mmu_mi_r_148b_3p,mmu_mi_r_149_3p,mmu_mi_r_154_5p,mmu_mi_r_155_3p,mmu_mi_r_16_2_3p,mmu_mi_r_16_5p,mmu_mi_r_1668,mmu_mi_r_17_3p,mmu_mi_r_181a_1_3p,mmu_mi_r_181b_1_3p,mmu_mi_r_181b_5p,mmu_mi_r_181c_3p,mmu_mi_r_1839_3p,mmu_mi_r_1839_5p,mmu_mi_r_184_3p,mmu_mi_r_1843a_3p,mmu_mi_r_1843b_5p,mmu_mi_r_185_3p,mmu_mi_r_186_3p,mmu_mi_r_187_3p,mmu_mi_r_187_5p,mmu_mi_r_188_5p,mmu_mi_r_1892,mmu_mi_r_1894_3p,mmu_mi_r_1896,mmu_mi_r_1898,mmu_mi_r_18b_5p,mmu_mi_r_1900,mmu_mi_r_1901,mmu_mi_r_1902,mmu_mi_r_190b_5p,mmu_mi_r_191_3p,mmu_mi_r_1912_3p,mmu_mi_r_192_3p,mmu_mi_r_192_5p,mmu_mi_r_1930_3p,mmu_mi_r_1931,mmu_mi_r_1933_3p,mmu_mi_r_1933_5p,mmu_mi_r_1938,mmu_mi_r_194_5p,mmu_mi_r_1941_3p,mmu_mi_r_1942,mmu_mi_r_1943_5p,mmu_mi_r_1946b,mmu_mi_r_1947_5p,mmu_mi_r_1948_3p,mmu_mi_r_1951,mmu_mi_r_1954,mmu_mi_r_1957a,mmu_mi_r_1958,mmu_mi_r_195a_3p,mmu_mi_r_195a_5p,mmu_mi_r_1961,mmu_mi_r_1962,mmu_mi_r_1966_5p,mmu_mi_r_196a_1_3p,mmu_mi_r_1981_3p,mmu_mi_r_1981_5p,mmu_mi_r_1983,mmu_mi_r_199b_5p,mmu_mi_r_200c_3p,mmu_mi_r_204_3p,mmu_mi_r_205_3p,mmu_mi_r_206_3p,mmu_mi_r_207,mmu_mi_r_208b_3p,mmu_mi_r_20a_3p,mmu_mi_r_20b_3p,mmu_mi_r_20b_5p,mmu_mi_r_210_5p,mmu_mi_r_212_5p,mmu_mi_r_215_3p,mmu_mi_r_215_5p,mmu_mi_r_216a_5p,mmu_mi_r_216b_3p,mmu_mi_r_216c_5p,mmu_mi_r_218_2_3p,mmu_mi_r_2183,mmu_mi_r_219b_5p,mmu_mi_r_219c_3p,mmu_mi_r_219c_5p,mmu_mi_r_21a_5p,mmu_mi_r_21b,mmu_mi_r_21c,mmu_mi_r_221_5p,mmu_mi_r_224_3p,mmu_mi_r_23a_5p,mmu_mi_r_25_3p,mmu_mi_r_25_5p,mmu_mi_r_26a_1_3p,mmu_mi_r_28a_5p,mmu_mi_r_28b,mmu_mi_r_290a_5p,mmu_mi_r_290b_5p,mmu_mi_r_291b_5p,mmu_mi_r_293_3p,mmu_mi_r_294_3p,mmu_mi_r_295_3p,mmu_mi_r_297b_5p,mmu_mi_r_29a_3p,mmu_mi_r_29a_5p,mmu_mi_r_29b_1_5p,mmu_mi_r_29b_3p,mmu_mi_r_29c_3p,mmu_mi_r_300_5p,mmu_mi_r_301a_3p,mmu_mi_r_301b_5p,mmu_mi_r_302a_5p,mmu_mi_r_302c_3p,mmu_mi_r_302c_5p,mmu_mi_r_302d_5p,mmu_mi_r_3057_3p,mmu_mi_r_3057_5p,mmu_mi_r_3058_5p,mmu_mi_r_3059_3p,mmu_mi_r_3060_5p,mmu_mi_r_3062_3p,mmu_mi_r_3063_5p,mmu_mi_r_3064_5p,mmu_mi_r_3065_3p,mmu_mi_r_3066_3p,mmu_mi_r_3067_5p,mmu_mi_r_3070_2_3p,mmu_mi_r_3070_3p,mmu_mi_r_3072_3p,mmu_mi_r_3073a_5p,mmu_mi_r_3073b_3p,mmu_mi_r_3073b_5p,mmu_mi_r_3076_3p,mmu_mi_r_3077_3p,mmu_mi_r_3078_3p,mmu_mi_r_3078_5p,mmu_mi_r_3079_3p,mmu_mi_r_3079_5p,mmu_mi_r_3082_3p,mmu_mi_r_3082_5p,mmu_mi_r_3085_3p,mmu_mi_r_3089_3p,mmu_mi_r_3089_5p,mmu_mi_r_3090_3p,mmu_mi_r_3091_3p,mmu_mi_r_3091_5p,mmu_mi_r_3093_5p,mmu_mi_r_3095_5p,mmu_mi_r_3098_3p,mmu_mi_r_3098_5p,mmu_mi_r_30a_3p,mmu_mi_r_30a_5p,mmu_mi_r_30b_3p,mmu_mi_r_30c_1_3p,mmu_mi_r_30d_3p,mmu_mi_r_30d_5p,mmu_mi_r_30e_3p,mmu_mi_r_30f.,mmu_mi_r_3100_5p,mmu_mi_r_3101_3p,mmu_mi_r_3102_3p,mmu_mi_r_3102_5p_2_5p,mmu_mi_r_3103_3p,mmu_mi_r_3103_5p,mmu_mi_r_3105_5p,mmu_mi_r_3106_3p,mmu_mi_r_3108_5p,mmu_mi_r_3112_3p,mmu_mi_r_3113_3p,mmu_mi_r_320_5p,mmu_mi_r_322_3p,mmu_mi_r_322_5p,mmu_mi_r_324_5p,mmu_mi_r_325_5p,mmu_mi_r_326_5p,mmu_mi_r_328_5p,mmu_mi_r_33_3p,mmu_mi_r_335_5p,mmu_mi_r_337_3p,mmu_mi_r_338_5p,mmu_mi_r_340_3p,mmu_mi_r_340_5p,mmu_mi_r_343,mmu_mi_r_344_3p,mmu_mi_r_344_5p,mmu_mi_r_344b_5p,mmu_mi_r_344c_3p,mmu_mi_r_344f_3p,mmu_mi_r_344f_5p,mmu_mi_r_344g_3p,mmu_mi_r_344h_3p,mmu_mi_r_346_3p,mmu_mi_r_346_5p,mmu_mi_r_3471,mmu_mi_r_3472,mmu_mi_r_3473d,mmu_mi_r_3473g,mmu_mi_r_3474,mmu_mi_r_3475_3p,mmu_mi_r_3475_5p,mmu_mi_r_34c_3p,mmu_mi_r_350_5p,mmu_mi_r_3535,mmu_mi_r_3544_3p,mmu_mi_r_3547_3p,mmu_mi_r_3572_5p,mmu_mi_r_361_3p,mmu_mi_r_3618_3p,mmu_mi_r_3620_3p,mmu_mi_r_3620_5p,mmu_mi_r_365_1_5p,mmu_mi_r_365_2_5p,mmu_mi_r_365_3p,mmu_mi_r_367_3p,mmu_mi_r_374b_3p,mmu_mi_r_374c_5p,mmu_mi_r_376b_3p,mmu_mi_r_376c_5p,mmu_mi_r_378b,mmu_mi_r_378c,mmu_mi_r_378d,mmu_mi_r_380_3p,mmu_mi_r_383_3p,mmu_mi_r_383_5p,mmu_mi_r_384_3p,mmu_mi_r_3965,mmu_mi_r_3967,mmu_mi_r_3970,mmu_mi_r_409_5p,mmu_mi_r_412_3p,mmu_mi_r_421_5p,mmu_mi_r_429_3p,mmu_mi_r_431_3p,mmu_mi_r_433_3p,mmu_mi_r_433_5p,mmu_mi_r_434_3p,mmu_mi_r_448_3p,mmu_mi_r_448_5p,mmu_mi_r_449a_3p,mmu_mi_r_450a_1_3p,mmu_mi_r_450a_5p,mmu_mi_r_451b,mmu_mi_r_455_3p,mmu_mi_r_465a_5p,mmu_mi_r_465b_5p,mmu_mi_r_465d_5p,mmu_mi_r_466c_5p,mmu_mi_r_466f_3p,mmu_mi_r_466g,mmu_mi_r_466i_3p,mmu_mi_r_466j,mmu_mi_r_466k,mmu_mi_r_466m_3p,mmu_mi_r_466m_5p_mmu_mi_r_669m_5p,mmu_mi_r_466n_3p,mmu_mi_r_466q,mmu_mi_r_467a_3p,mmu_mi_r_467b_3p,mmu_mi_r_467e_5p,mmu_mi_r_467h,mmu_mi_r_470_5p,mmu_mi_r_483_5p,mmu_mi_r_485_3p,mmu_mi_r_485_5p,mmu_mi_r_486a_3p,mmu_mi_r_486a_5p,mmu_mi_r_486b_3p,mmu_mi_r_487b_3p,mmu_mi_r_487b_5p,mmu_mi_r_490_3p,mmu_mi_r_491_3p,mmu_mi_r_491_5p,mmu_mi_r_493_5p,mmu_mi_r_496a_3p,mmu_mi_r_496a_5p,mmu_mi_r_496b,mmu_mi_r_500_5p,mmu_mi_r_503_3p,mmu_mi_r_503_5p,mmu_mi_r_505_3p,mmu_mi_r_509_3p,mmu_mi_r_5103,mmu_mi_r_5106,mmu_mi_r_5107_5p,mmu_mi_r_511_3p,mmu_mi_r_5119,mmu_mi_r_5121,mmu_mi_r_5123,mmu_mi_r_5124b,mmu_mi_r_5127,mmu_mi_r_5132_3p,mmu_mi_r_5132_5p,mmu_mi_r_532_5p,mmu_mi_r_540_5p,mmu_mi_r_542_5p,mmu_mi_r_544_3p,mmu_mi_r_544_5p,mmu_mi_r_5615_3p,mmu_mi_r_5615_5p,mmu_mi_r_5617_5p,mmu_mi_r_5619_3p,mmu_mi_r_5619_5p,mmu_mi_r_5620_3p,mmu_mi_r_5622_5p,mmu_mi_r_5623_5p,mmu_mi_r_5624_5p,mmu_mi_r_5625_3p,mmu_mi_r_5625_5p,mmu_mi_r_5627_3p,mmu_mi_r_5709_3p,mmu_mi_r_5709_5p,mmu_mi_r_582_5p,mmu_mi_r_590_3p,mmu_mi_r_592_3p,mmu_mi_r_598_5p,mmu_mi_r_6237,mmu_mi_r_6337,mmu_mi_r_6340,mmu_mi_r_6341,mmu_mi_r_6342,mmu_mi_r_6346,mmu_mi_r_6349,mmu_mi_r_6355,mmu_mi_r_6356,mmu_mi_r_6357,mmu_mi_r_6365,mmu_mi_r_6367,mmu_mi_r_6368,mmu_mi_r_6370,mmu_mi_r_6371,mmu_mi_r_6372,mmu_mi_r_6374,mmu_mi_r_6382,mmu_mi_r_6383,mmu_mi_r_6384,mmu_mi_r_6385,mmu_mi_r_6387,mmu_mi_r_6392_5p,mmu_mi_r_6393,mmu_mi_r_6396,mmu_mi_r_6398,mmu_mi_r_6400,mmu_mi_r_6402,mmu_mi_r_6404,mmu_mi_r_6411,mmu_mi_r_6412,mmu_mi_r_6414,mmu_mi_r_6416_5p,mmu_mi_r_6419,mmu_mi_r_653_5p,mmu_mi_r_6540_3p,mmu_mi_r_6540_5p,mmu_mi_r_6546_5p,mmu_mi_r_664_5p,mmu_mi_r_665_3p,mmu_mi_r_666_5p,mmu_mi_r_668_3p,mmu_mi_r_668_5p,mmu_mi_r_669a_5p_mmu_mi_r_669p_5p,mmu_mi_r_669b_3p,mmu_mi_r_669b_5p,mmu_mi_r_669c_3p,mmu_mi_r_669f_3p,mmu_mi_r_669g,mmu_mi_r_669h_5p,mmu_mi_r_669i,mmu_mi_r_669n,mmu_mi_r_670_3p,mmu_mi_r_672_3p,mmu_mi_r_673_5p,mmu_mi_r_674_3p,mmu_mi_r_676_3p,mmu_mi_r_6769b_3p,mmu_mi_r_677_3p,mmu_mi_r_682,mmu_mi_r_683,mmu_mi_r_6896_3p,mmu_mi_r_6896_5p,mmu_mi_r_6897_3p,mmu_mi_r_6897_5p,mmu_mi_r_6898_3p,mmu_mi_r_6898_5p,mmu_mi_r_6899_5p,mmu_mi_r_690,mmu_mi_r_6900_5p,mmu_mi_r_6902_3p,mmu_mi_r_6902_5p,mmu_mi_r_6903_5p,mmu_mi_r_6905_5p,mmu_mi_r_6906_3p,mmu_mi_r_6907_5p,mmu_mi_r_6908_3p,mmu_mi_r_6908_5p,mmu_mi_r_6910_5p,mmu_mi_r_6913_5p,mmu_mi_r_6914_5p,mmu_mi_r_6917_3p,mmu_mi_r_6917_5p,mmu_mi_r_6918_3p,mmu_mi_r_6918_5p,mmu_mi_r_6919_3p,mmu_mi_r_6919_5p,mmu_mi_r_692,mmu_mi_r_6920_3p,mmu_mi_r_6921_3p,mmu_mi_r_6922_5p,mmu_mi_r_6923_5p,mmu_mi_r_6924_3p,mmu_mi_r_6924_5p,mmu_mi_r_6928_3p,mmu_mi_r_6929_3p,mmu_mi_r_693_5p,mmu_mi_r_6930_3p,mmu_mi_r_6932_3p,mmu_mi_r_6932_5p,mmu_mi_r_6933_3p,mmu_mi_r_6935_5p,mmu_mi_r_6936_3p,mmu_mi_r_6937_5p,mmu_mi_r_6939_3p,mmu_mi_r_6939_5p,mmu_mi_r_694,mmu_mi_r_6940_3p,mmu_mi_r_6940_5p,mmu_mi_r_6942_3p,mmu_mi_r_6942_5p,mmu_mi_r_6943_3p,mmu_mi_r_6943_5p,mmu_mi_r_6944_3p,mmu_mi_r_6947_5p,mmu_mi_r_6948_5p,mmu_mi_r_6952_3p,mmu_mi_r_6953_3p,mmu_mi_r_6953_5p,mmu_mi_r_6954_3p,mmu_mi_r_6955_5p,mmu_mi_r_6956_3p,mmu_mi_r_6956_5p,mmu_mi_r_6958_3p,mmu_mi_r_6958_5p,mmu_mi_r_6959_3p,mmu_mi_r_6959_5p,mmu_mi_r_696,mmu_mi_r_6960_3p,mmu_mi_r_6962_3p,mmu_mi_r_6964_3p,mmu_mi_r_6964_5p,mmu_mi_r_6965_3p,mmu_mi_r_6965_5p,mmu_mi_r_6966_5p,mmu_mi_r_697,mmu_mi_r_6973a_3p,mmu_mi_r_6973b_3p,mmu_mi_r_6975_3p,mmu_mi_r_6975_5p,mmu_mi_r_6978_5p,mmu_mi_r_6979_3p,mmu_mi_r_6980_5p,mmu_mi_r_6981_5p,mmu_mi_r_6982_3p,mmu_mi_r_6982_5p,mmu_mi_r_6983_3p,mmu_mi_r_6984_3p,mmu_mi_r_6985_3p,mmu_mi_r_6986_3p,mmu_mi_r_6986_5p,mmu_mi_r_6988_5p,mmu_mi_r_6989_3p,mmu_mi_r_6990_5p,mmu_mi_r_6992_3p,mmu_mi_r_6992_5p,mmu_mi_r_6993_5p,mmu_mi_r_6994_3p,mmu_mi_r_6995_5p,mmu_mi_r_6996_3p,mmu_mi_r_6997_5p,mmu_mi_r_6998_5p,mmu_mi_r_6999_5p,mmu_mi_r_700_3p,mmu_mi_r_7005_3p,mmu_mi_r_7007_5p,mmu_mi_r_7008_5p,mmu_mi_r_7009_3p,mmu_mi_r_7010_3p,mmu_mi_r_7010_5p,mmu_mi_r_7012_3p,mmu_mi_r_7012_5p,mmu_mi_r_7013_3p,mmu_mi_r_7015_3p,mmu_mi_r_7016_5p,mmu_mi_r_7018_3p,mmu_mi_r_702_3p,mmu_mi_r_7022_3p,mmu_mi_r_7022_5p,mmu_mi_r_7024_3p,mmu_mi_r_7025_3p,mmu_mi_r_7030_3p,mmu_mi_r_7030_5p,mmu_mi_r_7032_5p,mmu_mi_r_7033_3p,mmu_mi_r_7033_5p,mmu_mi_r_7035_3p,mmu_mi_r_7035_5p,mmu_mi_r_7036a_3p,mmu_mi_r_7036b_3p,mmu_mi_r_7036b_5p,mmu_mi_r_7037_5p,mmu_mi_r_7038_3p,mmu_mi_r_7038_5p,mmu_mi_r_7043_3p,mmu_mi_r_7043_5p,mmu_mi_r_7047_3p,mmu_mi_r_7047_5p,mmu_mi_r_7048_3p,mmu_mi_r_7049_3p,mmu_mi_r_7050_5p,mmu_mi_r_7051_3p,mmu_mi_r_7051_5p,mmu_mi_r_7052_5p,mmu_mi_r_7053_5p,mmu_mi_r_7054_3p,mmu_mi_r_7057_3p,mmu_mi_r_7058_3p,mmu_mi_r_7059_3p,mmu_mi_r_7059_5p,mmu_mi_r_7063_5p,mmu_mi_r_7064_3p,mmu_mi_r_7065_5p,mmu_mi_r_7066_3p,mmu_mi_r_7066_5p,mmu_mi_r_7069_3p,mmu_mi_r_7070_5p,mmu_mi_r_7071_5p,mmu_mi_r_7072_5p,mmu_mi_r_7074_3p,mmu_mi_r_7075_3p,mmu_mi_r_7077_3p,mmu_mi_r_7077_5p,mmu_mi_r_7078_3p,mmu_mi_r_7078_5p,mmu_mi_r_7079_3p,mmu_mi_r_7079_5p,mmu_mi_r_708_5p,mmu_mi_r_7081_3p,mmu_mi_r_7082_5p,mmu_mi_r_7083_5p,mmu_mi_r_7084_5p,mmu_mi_r_7085_5p,mmu_mi_r_7086_3p,mmu_mi_r_7089_3p,mmu_mi_r_7090_3p,mmu_mi_r_7092_3p,mmu_mi_r_7094_1_5p,mmu_mi_r_7094_3p,mmu_mi_r_7115_5p,mmu_mi_r_7116_5p,mmu_mi_r_7117_3p,mmu_mi_r_7119_3p,mmu_mi_r_712_3p,mmu_mi_r_712_5p,mmu_mi_r_719,mmu_mi_r_7211_3p,mmu_mi_r_7213_5p,mmu_mi_r_7214_3p,mmu_mi_r_7215_3p,mmu_mi_r_7215_5p,mmu_mi_r_7216_3p,mmu_mi_r_7219_3p,mmu_mi_r_7 220_5p,mmu_mi_r_7222_5p,mmu_mi_r_7224_5p,mmu_mi_r_7225_3p,mmu_mi_r_7226_3p,mmu_mi_r_7227_5p,mmu_mi_r_7228_5p,mmu_mi_r_7229_3p,mmu_mi_r_7230_3p,mmu_mi_r_7231_3p,mmu_mi_r_7232_5p,mmu_mi_r_7233_3p,mmu_mi_r_7234_3p,mmu_mi_r_7234_5p,mmu_mi_r_7235_3p,mmu_mi_r_7236_3p,mmu_mi_r_7236_5p,mmu_mi_r_7237_5p,mmu_mi_r_7238_3p,mmu_mi_r_7238_5p,mmu_mi_r_7239_3p,mmu_mi_r_7241_3p,mmu_mi_r_7243_5p,mmu_mi_r_741_3p,mmu_mi_r_742_3p,mmu_mi_r_742_5p,mmu_mi_r_743b_5p,mmu_mi_r_7578,mmu_mi_r_758_5p,mmu_mi_r_759,mmu_mi_r_764_3p,mmu_mi_r_7646_3p,mmu_mi_r_7646_5p,mmu_mi_r_7648_3p,mmu_mi_r_7648_5p,mmu_mi_r_7649_5p,mmu_mi_r_7651_3p,mmu_mi_r_7651_5p,mmu_mi_r_7653_5p,mmu_mi_r_7654_3p,mmu_mi_r_7654_5p,mmu_mi_r_7655_3p,mmu_mi_r_7655_5p,mmu_mi_r_7656_5p,mmu_mi_r_7657_3p,mmu_mi_r_7658_5p,mmu_mi_r_7659_3p,mmu_mi_r_7660_5p,mmu_mi_r_7661_3p,mmu_mi_r_7662_3p,mmu_mi_r_7662_5p,mmu_mi_r_7663_5p,mmu_mi_r_7664_3p,mmu_mi_r_7665_3p,mmu_mi_r_7665_5p,mmu_mi_r_7667_3p,mmu_mi_r_7667_5p,mmu_mi_r_7670_3p,mmu_mi_r_7675_3p,mmu_mi_r_7675_5p,mmu_mi_r_7676_5p,mmu_mi_r_7677_3p,mmu_mi_r_7677_5p,mmu_mi_r_7679_5p,mmu_mi_r_7680_3p,mmu_mi_r_7681_3p,mmu_mi_r_7683_5p,mmu_mi_r_7684_5p,mmu_mi_r_7686_5p,mmu_mi_r_770_3p,mmu_mi_r_7a_1_3p,mmu_mi_r_7a_2_3p,mmu_mi_r_7a_5p,mmu_mi_r_802_3p,mmu_mi_r_802_5p,mmu_mi_r_8090,mmu_mi_r_8092,mmu_mi_r_8099,mmu_mi_r_8100,mmu_mi_r_8103,mmu_mi_r_8105,mmu_mi_r_8106,mmu_mi_r_8108,mmu_mi_r_8110,mmu_mi_r_8111,mmu_mi_r_8115,mmu_mi_r_8120,mmu_mi_r_871_3p,mmu_mi_r_873a_3p,mmu_mi_r_873a_5p,mmu_mi_r_873b,mmu_mi_r_876_5p,mmu_mi_r_879_3p,mmu_mi_r_879_5p,mmu_mi_r_880_3p,mmu_mi_r_881_3p,mmu_mi_r_881_5p,mmu_mi_r_882,mmu_mi_r_883a_5p,mmu_mi_r_883b_5p,mmu_mi_r_9_5p,mmu_mi_r_92a_2_5p,mmu_mi_r_92b_3p,mmu_mi_r_93_5p,mmu_mi_r_96_3p,mmu_mi_r_96_5p,mmu_mi_r_9768_3p,mmu_mi_r_9769_3p,mmu_mi_r_99a_3p,mmu_mi_r_99b_3p,mmu_pi_r_000366_gb_dq540412_mus_musculus_17_25602713_25602739_plus,mmu_pi_r_000578_gb_dq540853_mus_musculus_17_39456112_39456137_plus,mmu_pi_r_000620_gb_dq540981_mus_musculus_18_54824345_54824374_minus,mmu_pi_r_000620_gb_dq540981_mus_musculus_3_5843782_5843811_plus,mmu_pi_r_000622_gb_dq540988_mus_musculus_18_54824707_54824734_minus,mmu_pi_r_000622_gb_dq540988_mus_musculus_2_5296560_5296587_minus,mmu_pi_r_000622_gb_dq540988_mus_musculus_3_5843428_5843455_plus,mmu_pi_r_000639_gb_dq541113_mus_musculus_17_39455268_39455298_plus,mmu_pi_r_000691_gb_dq541218_mus_musculus_8_126462165_126462190_minus,mmu_pi_r_000691_gb_dq541218_mus_musculus_8_126484247_126484272_minus,mmu_pi_r_000691_gb_dq541218_mus_musculus_8_126485950_126485975_minus,mmu_pi_r_000691_gb_dq541218_mus_musculus_8_126492702_126492727_minus,mmu_pi_r_000691_gb_dq541218_mus_musculus_8_126494409_126494434_minus,mmu_pi_r_001570_gb_dq543701_mus_musculus_2_5296574_5296603_minus,mmu_pi_r_001662_gb_dq544105_mus_musculus_2_151104333_151104361_minus,mmu_pi_r_002435_gb_dq546549_mus_musculus_17_39454808_39454832_plus,mmu_pi_r_003848_gb_dq551015_mus_musculus_10_18489292_18489321_minus,mmu_pi_r_004374_gb_dq552696_mus_musculus_18_85832427_85832456_minus,mmu_pi_r_004374_gb_dq552696_mus_musculus_9_110126452_110126481_plus,mmu_pi_r_006854_gb_dq562105_mus_musculus_1_157716808_157716836_plus,mmu_pi_r_007829_gb_dq565297_mus_musculus_2_92382336_92382366_plus,mmu_pi_r_010309_gb_dq686298_mus_musculus_1_161177076_161177096_plus,mmu_pi_r_010565_gb_dq686723_mus_musculus_16_38309443_38309460_plus,mmu_pi_r_021097_gb_dq702429_mus_musculus_7_14975966_14975994_minus,mmu_pi_r_022956_gb_dq705141_mus_musculus_5_115112809_115112837_minus,mmu_pi_r_023189_gb_dq705481_mus_musculus_16_18197850_18197871_minus,mmu_pi_r_024456_gb_dq707344_mus_musculus_7_72990109_72990139_plus,mmu_pi_r_024744_gb_dq707762_mus_musculus_17_66099819_66099850_minus,mmu_pi_r_025576_gb_dq708952_mus_musculus_x_6405415_6405436_minus,mmu_pi_r_028252_gb_dq712837_mus_musculus_7_81403433_81403455_plus,mmu_pi_r_029416_gb_dq714514_mus_musculus_9_67542046_67542076_plus,mmu_pi_r_030303_gb_dq715776_mus_musculus_6_127764268_127764297_plus,mmu_pi_r_032865_gb_dq719430_mus_musculus_2_116876592_116876614_plus,mmu_pi_r_034208_gb_dq721450_mus_musculus_7_72980839_72980868_plus,mmu_pi_r_034249_gb_dq721513_mus_musculus_5_113579122_113579149_minus,mmu_pi_r_035342_gb_dq723105_mus_musculus_15_59084194_59084224_minus,mmu_pi_r_035552_gb_dq723401_mus_musculus_17_66117579_66117604_plus,mmu_pi_r_037443_gb_dq726144_mus_musculus_2_92364888_92364918_plus,mmu_pi_r_038323_gb_pi_rna_t34_mus_musculus_13_44880547_44880577_minus,mmu_pi_r_039147_gb_pi_rna_2740_mus_musculus_6_87962869_87962889_minus
1544.867	x1544_867_H	mmu_let_7a_2_3p,mmu_let_7b_5p,mmu_let_7d_3p,mmu_let_7d_5p,mmu_let_7g_3p,mmu_let_7g_5p,mmu_let_7i_3p,mmu_mi_r_101a_3p,mmu_mi_r_101b_3p,mmu_mi_r_101c,mmu_mi_r_103_1_5p,mmu_mi_r_1188_3p,mmu_mi_r_1194,mmu_mi_r_122_3p,mmu_mi_r_1264_3p,mmu_mi_r_128_3p,mmu_mi_r_1306_5p,mmu_mi_r_130b_3p,mmu_mi_r_130b_5p,mmu_mi_r_133a_3p,mmu_mi_r_133a_5p,mmu_mi_r_133b_3p,mmu_mi_r_135a_1_3p,mmu_mi_r_135b_5p,mmu_mi_r_137_5p,mmu_mi_r_140_3p,mmu_mi_r_140_5p,mmu_mi_r_142a_3p,mmu_mi_r_148b_3p,mmu_mi_r_148b_5p,mmu_mi_r_149_5p,mmu_mi_r_150_5p,mmu_mi_r_154_3p,mmu_mi_r_15a_3p,mmu_mi_r_15a_5p,mmu_mi_r_15b_5p,mmu_mi_r_16_1_3p,mmu_mi_r_16_5p,mmu_mi_r_181c_5p,mmu_mi_r_181d_5p,mmu_mi_r_1839_5p,mmu_mi_r_184_3p,mmu_mi_r_184_5p,mmu_mi_r_185_3p,mmu_mi_r_185_5p,mmu_mi_r_186_5p,mmu_mi_r_1896,mmu_mi_r_1898,mmu_mi_r_1905,mmu_mi_r_1907,mmu_mi_r_190b_5p,mmu_mi_r_191_5p,mmu_mi_r_1934_3p,mmu_mi_r_1946a,mmu_mi_r_1947_5p,mmu_mi_r_1958,mmu_mi_r_195a_5p,mmu_mi_r_1968_3p,mmu_mi_r_1970,mmu_mi_r_1981_5p,mmu_mi_r_19b_3p,mmu_mi_r_1a_2_5p,mmu_mi_r_202_3p,mmu_mi_r_202_5p,mmu_mi_r_204_3p,mmu_mi_r_208b_3p,mmu_mi_r_20a_5p,mmu_mi_r_20b_5p,mmu_mi_r_210_3p,mmu_mi_r_2139,mmu_mi_r_218_1_3p,mmu_mi_r_21c,mmu_mi_r_222_5p,mmu_mi_r_23b_5p,mmu_mi_r_24_2_5p,mmu_mi_r_25_3p,mmu_mi_r_25_5p,mmu_mi_r_26a_1_3p,mmu_mi_r_293_5p,mmu_mi_r_294_5p,mmu_mi_r_296_5p,mmu_mi_r_297a_3p_mmu_mi_r_297b_3p_mmu_mi_r_297c_3p,mmu_mi_r_297b_5p,mmu_mi_r_298_5p,mmu_mi_r_299b_3p,mmu_mi_r_300_3p,mmu_mi_r_301a_3p,mmu_mi_r_302b_5p,mmu_mi_r_302c_3p,mmu_mi_r_3059_3p,mmu_mi_r_3064_3p,mmu_mi_r_3068_5p,mmu_mi_r_3070_5p,mmu_mi_r_3075_3p,mmu_mi_r_3075_5p,mmu_mi_r_3077_3p,mmu_mi_r_3082_5p,mmu_mi_r_3089_5p,mmu_mi_r_3095_3p,mmu_mi_r_30a_3p,mmu_mi_r_30a_5p,mmu_mi_r_30b_5p,mmu_mi_r_30c_2_3p,mmu_mi_r_30c_5p,mmu_mi_r_30d_5p,mmu_mi_r_30e_5p,mmu_mi_r_3101_3p,mmu_mi_r_3102_3p,mmu_mi_r_3102_3p_2_3p,mmu_mi_r_3102_5p_2_5p,mmu_mi_r_3104_5p,mmu_mi_r_3108_3p,mmu_mi_r_3154,mmu_mi_r_32_3p,mmu_mi_r_322_3p,mmu_mi_r_322_5p,mmu_mi_r_328_3p,mmu_mi_r_337_3p,mmu_mi_r_337_5p,mmu_mi_r_341_3p,mmu_mi_r_341_5p,mmu_mi_r_342_3p,mmu_mi_r_343,mmu_mi_r_344b_5p,mmu_mi_r_344c_3p,mmu_mi_r_344c_5p,mmu_mi_r_344d_3p,mmu_mi_r_345_3p,mmu_mi_r_3471,mmu_mi_r_3473d,mmu_mi_r_3475_3p,mmu_mi_r_350_3p,mmu_mi_r_350_5p,mmu_mi_r_3572_3p,mmu_mi_r_369_5p,mmu_mi_r_376a_3p,mmu_mi_r_376b_3p,mmu_mi_r_376b_5p,mmu_mi_r_378a_3p,mmu_mi_r_378a_5p,mmu_mi_r_378b,mmu_mi_r_378c,mmu_mi_r_378d,mmu_mi_r_383_3p,mmu_mi_r_3960,mmu_mi_r_3965,mmu_mi_r_3967,mmu_mi_r_409_3p,mmu_mi_r_421_3p,mmu_mi_r_425_3p,mmu_mi_r_450b_3p,mmu_mi_r_450b_5p,mmu_mi_r_452_5p,mmu_mi_r_455_3p,mmu_mi_r_466i_3p,mmu_mi_r_468_5p,mmu_mi_r_486b_5p,mmu_mi_r_487b_5p,mmu_mi_r_490_3p,mmu_mi_r_494_3p,mmu_mi_r_497a_5p,mmu_mi_r_499_3p,mmu_mi_r_503_3p,mmu_mi_r_503_5p,mmu_mi_r_504_5p,mmu_mi_r_5099,mmu_mi_r_5123,mmu_mi_r_5135,mmu_mi_r_542_3p,mmu_mi_r_542_5p,mmu_mi_r_5617_3p,mmu_mi_r_5618_5p,mmu_mi_r_590_3p,mmu_mi_r_615_3p,mmu_mi_r_6237,mmu_mi_r_6241,mmu_mi_r_6335,mmu_mi_r_6339,mmu_mi_r_6341,mmu_mi_r_6345,mmu_mi_r_6355,mmu_mi_r_6366,mmu_mi_r_6375,mmu_mi_r_6379,mmu_mi_r_6405,mmu_mi_r_6412,mmu_mi_r_6541,mmu_mi_r_664_5p,mmu_mi_r_667_5p,mmu_mi_r_669m_3p,mmu_mi_r_670_5p,mmu_mi_r_672_5p,mmu_mi_r_673_5p,mmu_mi_r_676_3p,mmu_mi_r_676_5p,mmu_mi_r_677_5p,mmu_mi_r_6905_5p,mmu_mi_r_691,mmu_mi_r_6925_5p,mmu_mi_r_6939_5p,mmu_mi_r_6942_3p,mmu_mi_r_6945_3p,mmu_mi_r_6947_5p,mmu_mi_r_6952_5p,mmu_mi_r_6955_5p,mmu_mi_r_6956_5p,mmu_mi_r_6958_5p,mmu_mi_r_6960_5p,mmu_mi_r_6961_5p,mmu_mi_r_6962_5p,mmu_mi_r_6963_5p,mmu_mi_r_6964_5p,mmu_mi_r_6966_5p,mmu_mi_r_6967_3p,mmu_mi_r_6969_5p,mmu_mi_r_6973a_5p,mmu_mi_r_6979_5p,mmu_mi_r_698_5p,mmu_mi_r_6992_5p,mmu_mi_r_6993_5p,mmu_mi_r_6999_5p,mmu_mi_r_7001_5p,mmu_mi_r_7003_5p,mmu_mi_r_7004_3p,mmu_mi_r_7011_5p,mmu_mi_r_7014_5p,mmu_mi_r_7015_3p,mmu_mi_r_7016_3p,mmu_mi_r_7020_5p,mmu_mi_r_7021_5p,mmu_mi_r_7023_5p,mmu_mi_r_7025_5p,mmu_mi_r_7031_5p,mmu_mi_r_7033_3p,mmu_mi_r_7045_3p,mmu_mi_r_7048_5p,mmu_mi_r_705,mmu_mi_r_7054_3p,mmu_mi_r_7054_5p,mmu_mi_r_7055_3p,mmu_mi_r_7057_3p,mmu_mi_r_7060_3p,mmu_mi_r_7063_3p,mmu_mi_r_7067_5p,mmu_mi_r_7071_5p,mmu_mi_r_7074_5p,mmu_mi_r_7079_3p,mmu_mi_r_7080_3p,mmu_mi_r_7084_3p,mmu_mi_r_7087_3p,mmu_mi_r_7091_3p,mmu_mi_r_7093_3p,mmu_mi_r_7115_5p,mmu_mi_r_721,mmu_mi_r_7220_3p,mmu_mi_r_7230_5p,mmu_mi_r_7234_5p,mmu_mi_r_7235_5p,mmu_mi_r_7243_3p,mmu_mi_r_742_3p,mmu_mi_r_743a_3p,mmu_mi_r_744_5p,mmu_mi_r_760_3p,mmu_mi_r_7648_5p,mmu_mi_r_7661_3p,mmu_mi_r_7665_5p,mmu_mi_r_7671_5p,mmu_mi_r_7679_5p,mmu_mi_r_7688_5p,mmu_mi_r_770_3p,mmu_mi_r_7a_1_3p,mmu_mi_r_8090,mmu_mi_r_8091,mmu_mi_r_8097,mmu_mi_r_8105,mmu_mi_r_8117,mmu_mi_r_882,mmu_mi_r_92a_3p,mmu_mi_r_93_5p,mmu_mi_r_96_5p,mmu_mi_r_9768_3p,mmu_mi_r_99a_3p,mmu_mi_r_99b_3p,mmu_pi_r_000159_gb_dq539904_mus_musculus_2_73668844_73668871_plus,mmu_pi_r_000366_gb_dq540412_mus_musculus_17_25602713_25602739_plus,mmu_pi_r_000691_gb_dq541218_mus_musculus_8_126494409_126494434_minus,mmu_pi_r_000935_gb_dq541777_mus_musculus_6_47717737_47717766_minus,mmu_pi_r_004086_gb_dq551625_mus_musculus_2_129969457_129969485_plus,mmu_pi_r_005109_gb_dq555094_mus_musculus_5_146565258_146565289_plus,mmu_pi_r_017405_gb_dq696996_mus_musculus_11_65550994_65551015_minus,mmu_pi_r_024744_gb_dq707762_mus_musculus_17_66099819_66099850_minus,mmu_pi_r_028252_gb_dq712837_mus_musculus_7_81403433_81403455_plus,mmu_pi_r_032865_gb_dq719430_mus_musculus_2_116876592_116876614_plus
1544.869	x1544_869_A	mmu_let_7c_1_3p,mmu_let_7e_3p,mmu_mi_r_107_5p,mmu_mi_r_1188_5p,mmu_mi_r_1191b_5p,mmu_mi_r_1194,mmu_mi_r_1197_3p,mmu_mi_r_1198_3p,mmu_mi_r_1199_5p,mmu_mi_r_1258_5p,mmu_mi_r_126a_5p,mmu_mi_r_127_5p,mmu_mi_r_135a_2_3p,mmu_mi_r_135a_5p,mmu_mi_r_135b_3p,mmu_mi_r_137_5p,mmu_mi_r_138_1_3p,mmu_mi_r_146b_5p,mmu_mi_r_149_3p,mmu_mi_r_153_5p,mmu_mi_r_155_3p,mmu_mi_r_155_5p,mmu_mi_r_1668,mmu_mi_r_181a_1_3p,mmu_mi_r_181d_5p,mmu_mi_r_1843a_5p,mmu_mi_r_188_3p,mmu_mi_r_1893,mmu_mi_r_18b_3p,mmu_mi_r_1904,mmu_mi_r_1912_3p,mmu_mi_r_1930_3p,mmu_mi_r_1930_5p,mmu_mi_r_1932,mmu_mi_r_1934_3p,mmu_mi_r_1942,mmu_mi_r_1943_3p,mmu_mi_r_1943_5p,mmu_mi_r_1957a,mmu_mi_r_196a_2_3p,mmu_mi_r_1982_3p,mmu_mi_r_1982_5p,mmu_mi_r_1983,mmu_mi_r_200b_3p,mmu_mi_r_200b_5p,mmu_mi_r_200c_3p,mmu_mi_r_200c_5p,mmu_mi_r_201_3p,mmu_mi_r_2136,mmu_mi_r_216a_3p,mmu_mi_r_216b_5p,mmu_mi_r_219c_3p,mmu_mi_r_22_5p,mmu_mi_r_222_5p,mmu_mi_r_224_3p,mmu_mi_r_27b_5p,mmu_mi_r_28b,mmu_mi_r_28c,mmu_mi_r_290b_3p,mmu_mi_r_291a_5p,mmu_mi_r_297a_3p_mmu_mi_r_297b_3p_mmu_mi_r_297c_3p,mmu_mi_r_298_3p,mmu_mi_r_29a_5p,mmu_mi_r_29b_1_5p,mmu_mi_r_29b_3p,mmu_mi_r_300_5p,mmu_mi_r_301b_5p,mmu_mi_r_302b_3p,mmu_mi_r_302d_5p,mmu_mi_r_3061_3p,mmu_mi_r_3062_5p,mmu_mi_r_3063_5p,mmu_mi_r_3064_5p,mmu_mi_r_3065_5p,mmu_mi_r_3068_3p,mmu_mi_r_3070_3p,mmu_mi_r_3070_5p,mmu_mi_r_3074_1_3p,mmu_mi_r_3075_5p,mmu_mi_r_3076_5p,mmu_mi_r_3081_5p,mmu_mi_r_3082_3p,mmu_mi_r_3082_5p,mmu_mi_r_3089_3p,mmu_mi_r_3090_3p,mmu_mi_r_3091_3p,mmu_mi_r_3099_3p,mmu_mi_r_31_5p,mmu_mi_r_3103_3p,mmu_mi_r_3113_5p,mmu_mi_r_322_3p,mmu_mi_r_322_5p,mmu_mi_r_335_3p,mmu_mi_r_337_3p,mmu_mi_r_337_5p,mmu_mi_r_338_5p,mmu_mi_r_344b_5p,mmu_mi_r_344e_3p,mmu_mi_r_3470a,mmu_mi_r_3473g,mmu_mi_r_350_5p,mmu_mi_r_351_5p,mmu_mi_r_3572_5p,mmu_mi_r_3620_3p,mmu_mi_r_367_5p,mmu_mi_r_381_5p,mmu_mi_r_448_5p,mmu_mi_r_449a_5p,mmu_mi_r_449b,mmu_mi_r_466a_3p_mmu_mi_r_466e_3p,mmu_mi_r_466b_3p_mmu_mi_r_466c_3p_mmu_mi_r_466p_3p,mmu_mi_r_466d_3p,mmu_mi_r_466m_3p,mmu_mi_r_467a_5p,mmu_mi_r_467c_5p,mmu_mi_r_467d_5p,mmu_mi_r_467e_3p,mmu_mi_r_467e_5p,mmu_mi_r_470_3p,mmu_mi_r_497b,mmu_mi_r_503_5p,mmu_mi_r_504_3p,mmu_mi_r_5101,mmu_mi_r_5116,mmu_mi_r_5121,mmu_mi_r_5129_3p,mmu_mi_r_5130,mmu_mi_r_5134_3p,mmu_mi_r_540_3p,mmu_mi_r_5616_3p,mmu_mi_r_5620_5p,mmu_mi_r_5621_5p,mmu_mi_r_5624_3p,mmu_mi_r_5626_5p,mmu_mi_r_568,mmu_mi_r_592_3p,mmu_mi_r_598_5p,mmu_mi_r_6238,mmu_mi_r_6336,mmu_mi_r_6342,mmu_mi_r_6349,mmu_mi_r_6360,mmu_mi_r_6362,mmu_mi_r_6366,mmu_mi_r_6369,mmu_mi_r_6373,mmu_mi_r_6385,mmu_mi_r_6386,mmu_mi_r_6389,mmu_mi_r_6391,mmu_mi_r_6396,mmu_mi_r_6481,mmu_mi_r_6540_3p,mmu_mi_r_6540_5p,mmu_mi_r_6541,mmu_mi_r_6546_5p,mmu_mi_r_669a_3p_mmu_mi_r_669o_3p,mmu_mi_r_669b_3p,mmu_mi_r_669c_5p,mmu_mi_r_670_5p,mmu_mi_r_672_5p,mmu_mi_r_674_3p,mmu_mi_r_6899_5p,mmu_mi_r_6901_3p,mmu_mi_r_6902_5p,mmu_mi_r_6904_3p,mmu_mi_r_6906_3p,mmu_mi_r_6914_3p,mmu_mi_r_6914_5p,mmu_mi_r_6919_3p,mmu_mi_r_6922_3p,mmu_mi_r_6927_3p,mmu_mi_r_6927_5p,mmu_mi_r_6928_5p,mmu_mi_r_6935_3p,mmu_mi_r_6936_5p,mmu_mi_r_6938_3p,mmu_mi_r_6939_3p,mmu_mi_r_694,mmu_mi_r_6941_3p,mmu_mi_r_6944_3p,mmu_mi_r_6945_5p,mmu_mi_r_6947_5p,mmu_mi_r_6951_5p,mmu_mi_r_6952_3p,mmu_mi_r_6954_5p,mmu_mi_r_6956_3p,mmu_mi_r_6961_3p,mmu_mi_r_6962_5p,mmu_mi_r_6964_3p,mmu_mi_r_6965_5p,mmu_mi_r_6966_5p,mmu_mi_r_6969_3p,mmu_mi_r_6970_5p,mmu_mi_r_6977_3p,mmu_mi_r_6978_3p,mmu_mi_r_6979_3p,mmu_mi_r_6982_5p,mmu_mi_r_6984_5p,mmu_mi_r_6988_3p,mmu_mi_r_6991_3p,mmu_mi_r_6993_3p,mmu_mi_r_6995_3p,mmu_mi_r_6997_3p,mmu_mi_r_7007_3p,mmu_mi_r_7008_3p,mmu_mi_r_7010_3p,mmu_mi_r_7013_5p,mmu_mi_r_7017_5p,mmu_mi_r_7018_3p,mmu_mi_r_7019_5p,mmu_mi_r_702_3p,mmu_mi_r_7024_3p,mmu_mi_r_7026_3p,mmu_mi_r_7027_5p,mmu_mi_r_7033_5p,mmu_mi_r_7034_3p,mmu_mi_r_7037_5p,mmu_mi_r_7040_5p,mmu_mi_r_7041_3p,mmu_mi_r_7049_5p,mmu_mi_r_7051_5p,mmu_mi_r_7053_5p,mmu_mi_r_7055_5p,mmu_mi_r_7057_5p,mmu_mi_r_706,mmu_mi_r_7068_3p,mmu_mi_r_7078_3p,mmu_mi_r_7080_5p,mmu_mi_r_7082_3p,mmu_mi_r_7083_5p,mmu_mi_r_7085_5p,mmu_mi_r_7086_3p,mmu_mi_r_7089_3p,mmu_mi_r_709,mmu_mi_r_7094b_2_5p,mmu_mi_r_7116_3p,mmu_mi_r_7119_3p,mmu_mi_r_7212_3p,mmu_mi_r_7218_3p,mmu_mi_r_7223_3p,mmu_mi_r_7226_5p,mmu_mi_r_7229_3p,mmu_mi_r_7230_5p,mmu_mi_r_7233_3p,mmu_mi_r_7243_3p,mmu_mi_r_741_5p,mmu_mi_r_743a_3p,mmu_mi_r_743a_5p,mmu_mi_r_743b_3p,mmu_mi_r_758_3p,mmu_mi_r_7646_3p,mmu_mi_r_7647_3p,mmu_mi_r_7652_3p,mmu_mi_r_7656_3p,mmu_mi_r_7658_5p,mmu_mi_r_7659_5p,mmu_mi_r_7661_3p,mmu_mi_r_7668_3p,mmu_mi_r_7673_3p,mmu_mi_r_7674_3p,mmu_mi_r_7676_3p,mmu_mi_r_7684_5p,mmu_mi_r_7687_5p,mmu_mi_r_7b_3p,mmu_mi_r_8106,mmu_mi_r_8109,mmu_mi_r_8115,mmu_mi_r_871_3p,mmu_mi_r_876_5p,mmu_mi_r_877_3p,mmu_mi_r_880_3p,mmu_mi_r_880_5p,mmu_mi_r_881_3p,mmu_mi_r_883a_3p,mmu_pi_r_000159_gb_dq539904_mus_musculus_2_73668844_73668871_plus,mmu_pi_r_000362_gb_dq540403_mus_musculus_6_3151474_3151503_plus,mmu_pi_r_000619_gb_dq540976_mus_musculus_17_39454691_39454717_plus,mmu_pi_r_000622_gb_dq540988_mus_musculus_2_5296560_5296587_minus,mmu_pi_r_000622_gb_dq540988_mus_musculus_3_5843428_5843455_plus,mmu_pi_r_000622_gb_dq540988_mus_musculus_x_112404287_112404314_minus,mmu_pi_r_000691_gb_dq541218_mus_musculus_11_74136081_74136106_plus,mmu_pi_r_000691_gb_dq541218_mus_musculus_8_126424875_126424900_minus,mmu_pi_r_000691_gb_dq541218_mus_musculus_8_126455417_126455442_minus,mmu_pi_r_000691_gb_dq541218_mus_musculus_8_126457090_126457115_minus,mmu_pi_r_000691_gb_dq541218_mus_musculus_8_126467244_126467269_minus,mmu_pi_r_000691_gb_dq541218_mus_musculus_8_126472331_126472356_minus,mmu_pi_r_000691_gb_dq541218_mus_musculus_8_126492702_126492727_minus,mmu_pi_r_000691_gb_dq541218_mus_musculus_8_126494409_126494434_minus,mmu_pi_r_003399_gb_dq549760_mus_musculus_5_108144856_108144884_plus,mmu_pi_r_016623_gb_dq695859_mus_musculus_9_54023526_54023555_minus,mmu_pi_r_017289_gb_dq696831_mus_musculus_6_128804704_128804734_plus,mmu_pi_r_034512_gb_dq721887_mus_musculus_14_22936178_22936204_plus,mmu_pi_r_037947_gb_dq726864_mus_musculus_9_54023523_54023552_minus,mmu_pi_r_038328_gb_pi_rna_t47_mus_musculus_17_39455055_39455084_plus,mmu_pi_r_038328_gb_pi_rna_t47_mus_musculus_19_13121850_13121879_plus
1572.899	x1572_899_A	mmu_let_7k,mmu_mi_r_100_3p,mmu_mi_r_101a_5p,mmu_mi_r_106a_5p,mmu_mi_r_107_5p,mmu_mi_r_1198_3p,mmu_mi_r_1231_3p,mmu_mi_r_124_5p,mmu_mi_r_1249_3p,mmu_mi_r_125b_1_3p,mmu_mi_r_126b_3p,mmu_mi_r_126b_5p,mmu_mi_r_129_2_3p,mmu_mi_r_135a_5p,mmu_mi_r_136_3p,mmu_mi_r_143_3p,mmu_mi_r_143_5p,mmu_mi_r_145a_5p,mmu_mi_r_145b,mmu_mi_r_146b_5p,mmu_mi_r_154_5p,mmu_mi_r_183_5p,mmu_mi_r_185_3p,mmu_mi_r_187_3p,mmu_mi_r_1903,mmu_mi_r_190a_3p,mmu_mi_r_190a_5p,mmu_mi_r_190b_3p,mmu_mi_r_1912_3p,mmu_mi_r_192_5p,mmu_mi_r_193a_5p,mmu_mi_r_194_1_3p,mmu_mi_r_194_5p,mmu_mi_r_1941_3p,mmu_mi_r_1946a,mmu_mi_r_1947_5p,mmu_mi_r_1954,mmu_mi_r_1963,mmu_mi_r_1964_5p,mmu_mi_r_1970,mmu_mi_r_1a_1_5p,mmu_mi_r_1b_5p,mmu_mi_r_200a_5p,mmu_mi_r_200b_3p,mmu_mi_r_200b_5p,mmu_mi_r_201_5p,mmu_mi_r_202_5p,mmu_mi_r_20b_3p,mmu_mi_r_20b_5p,mmu_mi_r_215_3p,mmu_mi_r_215_5p,mmu_mi_r_216a_3p,mmu_mi_r_216b_3p,mmu_mi_r_219a_1_3p,mmu_mi_r_219a_2_3p,mmu_mi_r_219c_3p,mmu_mi_r_21c,mmu_mi_r_223_3p,mmu_mi_r_223_5p,mmu_mi_r_23a_5p,mmu_mi_r_26a_1_3p,mmu_mi_r_28a_3p,mmu_mi_r_28a_5p,mmu_mi_r_292a_5p,mmu_mi_r_29a_3p,mmu_mi_r_29b_1_5p,mmu_mi_r_29b_3p,mmu_mi_r_29c_3p,mmu_mi_r_302b_3p,mmu_mi_r_3060_5p,mmu_mi_r_3061_5p,mmu_mi_r_3065_3p,mmu_mi_r_3065_5p,mmu_mi_r_3066_5p,mmu_mi_r_3069_5p,mmu_mi_r_3074_5p,mmu_mi_r_3075_3p,mmu_mi_r_3075_5p,mmu_mi_r_3078_3p,mmu_mi_r_3078_5p,mmu_mi_r_3086_3p,mmu_mi_r_3091_3p,mmu_mi_r_3091_5p,mmu_mi_r_3092_5p,mmu_mi_r_30b_3p,mmu_mi_r_30e_3p,mmu_mi_r_30f,mmu_mi_r_3100_3p,mmu_mi_r_3101_5p,mmu_mi_r_3102_3p_2_3p,mmu_mi_r_3102_5p,mmu_mi_r_3103_5p,mmu_mi_r_3104_3p,mmu_mi_r_3110_5p,mmu_mi_r_335_3p,mmu_mi_r_337_3p,mmu_mi_r_338_3p,mmu_mi_r_340_3p,mmu_mi_r_3470b,mmu_mi_r_3471,mmu_mi_r_3473d,mmu_mi_r_3474,mmu_mi_r_3475_5p,mmu_mi_r_34a_5p,mmu_mi_r_351_3p,mmu_mi_r_3544_3p,mmu_mi_r_3544_5p,mmu_mi_r_3547_5p,mmu_mi_r_376b_5p,mmu_mi_r_378b,mmu_mi_r_378d,mmu_mi_r_384_5p,mmu_mi_r_3961,mmu_mi_r_3964,mmu_mi_r_3967,mmu_mi_r_3969,mmu_mi_r_3970,mmu_mi_r_3971,mmu_mi_r_412_5p,mmu_mi_r_448_3p,mmu_mi_r_448_5p,mmu_mi_r_451b,mmu_mi_r_465d_3p,mmu_mi_r_466d_3p,mmu_mi_r_466i_3p,mmu_mi_r_466i_5p,mmu_mi_r_466m_5p_mmu_mi_r_669m_5p,mmu_mi_r_466q,mmu_mi_r_467e_3p,mmu_mi_r_468_5p,mmu_mi_r_483_5p,mmu_mi_r_486b_3p,mmu_mi_r_487b_3p,mmu_mi_r_491_5p,mmu_mi_r_503_5p,mmu_mi_r_511_5p,mmu_mi_r_5110,mmu_mi_r_5119,mmu_mi_r_5121,mmu_mi_r_5122,mmu_mi_r_5129_5p,mmu_mi_r_5134_5p,mmu_mi_r_541_3p,mmu_mi_r_541_5p,mmu_mi_r_542_3p,mmu_mi_r_6238,mmu_mi_r_6343,mmu_mi_r_6348,mmu_mi_r_6353,mmu_mi_r_6387,mmu_mi_r_6392_5p,mmu_mi_r_6395,mmu_mi_r_6398,mmu_mi_r_6410,mmu_mi_r_6414,mmu_mi_r_6417,mmu_mi_r_6418_3p,mmu_mi_r_6418_5p,mmu_mi_r_6516_3p,mmu_mi_r_6516_5p,mmu_mi_r_6540_3p,mmu_mi_r_6541,mmu_mi_r_665_3p,mmu_mi_r_666_3p,mmu_mi_r_669a_3_3p,mmu_mi_r_669p_3p,mmu_mi_r_672_3p,mmu_mi_r_673_5p,mmu_mi_r_679_5p,mmu_mi_r_688,mmu_mi_r_6899_3p,mmu_mi_r_690,mmu_mi_r_6901_5p,mmu_mi_r_6910_5p,mmu_mi_r_6912_5p,mmu_mi_r_6914_3p,mmu_mi_r_6917_5p,mmu_mi_r_6918_3p,mmu_mi_r_6918_5p,mmu_mi_r_6919_3p,mmu_mi_r_6920_3p,mmu_mi_r_6925_5p,mmu_mi_r_6929_3p,mmu_mi_r_693_3p,mmu_mi_r_693_5p,mmu_mi_r_6931_5p,mmu_mi_r_6932_5p,mmu_mi_r_6933_5p,mmu_mi_r_6935_5p,mmu_mi_r_6941_5p,mmu_mi_r_6956_3p,mmu_mi_r_6971_3p,mmu_mi_r_6973a_5p,mmu_mi_r_6975_3p,mmu_mi_r_6975_5p,mmu_mi_r_698_5p,mmu_mi_r_6984_3p,mmu_mi_r_6988_3p,mmu_mi_r_6989_5p,mmu_mi_r_6997_3p,mmu_mi_r_6998_5p,mmu_mi_r_7003_5p,mmu_mi_r_7005_5p,mmu_mi_r_7016_5p,mmu_mi_r_702_3p,mmu_mi_r_7020_5p,mmu_mi_r_7032_5p,mmu_mi_r_7036b_5p,mmu_mi_r_7037_5p,mmu_mi_r_7041_5p,mmu_mi_r_7046_5p,mmu_mi_r_7047_3p,mmu_mi_r_7050_3p,mmu_mi_r_7051_3p,mmu_mi_r_7055_3p,mmu_mi_r_7055_5p,mmu_mi_r_7063_3p,mmu_mi_r_7064_3p,mmu_mi_r_7067_5p,mmu_mi_r_7078_5p,mmu_mi_r_7080_3p,mmu_mi_r_7091_5p,mmu_mi_r_7093_5p,mmu_mi_r_711,mmu_mi_r_7119_5p,mmu_mi_r_717,mmu_mi_r_721,mmu_mi_r_7216_3p,mmu_mi_r_7216_5p,mmu_mi_r_7217_3p,mmu_mi_r_7224_3p,mmu_mi_r_7230_3p,mmu_mi_r_7231_3p,mmu_mi_r_7233_3p,mmu_mi_r_7242_3p,mmu_mi_r_741_3p,mmu_mi_r_742_3p,mmu_mi_r_743b_3p,mmu_mi_r_7648_5p,mmu_mi_r_7649_3p,mmu_mi_r_7653_3p,mmu_mi_r_7653_5p,mmu_mi_r_7660_3p,mmu_mi_r_7662_5p,mmu_mi_r_7670_3p,mmu_mi_r_7672_5p,mmu_mi_r_7676_3p,mmu_mi_r_7677_3p,mmu_mi_r_7679_3p,mmu_mi_r_7681_5p,mmu_mi_r_7b_5p,mmu_mi_r_802_5p,mmu_mi_r_8093,mmu_mi_r_8102,mmu_mi_r_8103,mmu_mi_r_8116,mmu_mi_r_8117,mmu_mi_r_8119,mmu_mi_r_873b,mmu_mi_r_881_3p,mmu_mi_r_881_5p,mmu_mi_r_9769_3p,mmu_mi_r_99b_3p,mmu_pi_r_000691_gb_dq541218_mus_musculus_8_126457090_126457115_minus,mmu_pi_r_000691_gb_dq541218_mus_musculus_8_126462165_126462190_minus,mmu_pi_r_000691_gb_dq541218_mus_musculus_8_126472331_126472356_minus,mmu_pi_r_000691_gb_dq541218_mus_musculus_8_126484247_126484272_minus,mmu_pi_r_000691_gb_dq541218_mus_musculus_8_126492702_126492727_minus,mmu_pi_r_000691_gb_dq541218_mus_musculus_8_126494409_126494434_minus,mmu_pi_r_000958_gb_dq541851_mus_musculus_17_39456228_39456254_plus,mmu_pi_r_002770_gb_dq547637_mus_musculus_2_92382387_92382415_plus,mmu_pi_r_007729_gb_dq564913_mus_musculus_9_123310826_123310855_plus,mmu_pi_r_016623_gb_dq695859_mus_musculus_9_54023526_54023555_minus,mmu_pi_r_017405_gb_dq696996_mus_musculus_11_65550994_65551015_minus,mmu_pi_r_020492_gb_dq701563_mus_musculus_11_108827972_108827997_minus,mmu_pi_r_025576_gb_dq708952_mus_musculus_x_6405415_6405436_minus,mmu_pi_r_028975_gb_dq713872_mus_musculus_x_6405378_6405399_minus,mmu_pi_r_030152_gb_dq715561_mus_musculus_18_67167521_67167549_minus,mmu_pi_r_034512_gb_dq721887_mus_musculus_14_22936178_22936204_plus,mmu_pi_r_038323_gb_pi_rna_t34_mus_musculus_13_44880547_44880577_minus,mmu_pi_r_039147_gb_pi_rna_2740_mus_musculus_9_118523649_118523669_minus
1863.995	x1863_995_H	mmu_let_7a_1_3p_mmu_let_7c_2_3p,mmu_let_7a_5p,mmu_let_7b_3p,mmu_let_7b_5p,mmu_let_7c_1_3p,mmu_let_7c_5p,mmu_let_7d_5p,mmu_let_7e_5p,mmu_let_7f_1_3p,mmu_let_7f_5p,mmu_let_7g_5p,mmu_let_7i_3p,mmu_let_7i_5p,mmu_let_7j,mmu_let_7k,mmu_mi_r_100_3p,mmu_mi_r_101a_3p,mmu_mi_r_101a_5p,mmu_mi_r_101b_3p,mmu_mi_r_101c,mmu_mi_r_103_3p,mmu_mi_r_107_3p,mmu_mi_r_1192,mmu_mi_r_125b_2_3p,mmu_mi_r_125b_5p,mmu_mi_r_126a_3p,mmu_mi_r_126b_5p,mmu_mi_r_127_3p,mmu_mi_r_128_1_5p,mmu_mi_r_1298_5p,mmu_mi_r_133b_5p,mmu_mi_r_134_3p,mmu_mi_r_139_3p,mmu_mi_r_140_5p,mmu_mi_r_142a_3p,mmu_mi_r_142a_5p,mmu_mi_r_142b,mmu_mi_r_144_3p,mmu_mi_r_144_5p,mmu_mi_r_146b_5p,mmu_mi_r_155_3p,mmu_mi_r_155_5p,mmu_mi_r_15a_3p,mmu_mi_r_15a_5p,mmu_mi_r_15b_3p,mmu_mi_r_15b_5p,mmu_mi_r_16_2_3p,mmu_mi_r_16_5p,mmu_mi_r_17_3p,mmu_mi_r_181a_2_3p,mmu_mi_r_1839_3p,mmu_mi_r_185_3p,mmu_mi_r_186_5p,mmu_mi_r_1894_5p,mmu_mi_r_1901,mmu_mi_r_1905,mmu_mi_r_1906,mmu_mi_r_190a_3p,mmu_mi_r_190a_5p,mmu_mi_r_190b_5p,mmu_mi_r_191_5p,mmu_mi_r_1942,mmu_mi_r_1950,mmu_mi_r_1954,mmu_mi_r_195a_5p,mmu_mi_r_1968_5p,mmu_mi_r_196b_5p,mmu_mi_r_1982_3p,mmu_mi_r_19a_3p,mmu_mi_r_205_3p,mmu_mi_r_20b_5p,mmu_mi_r_210_3p,mmu_mi_r_211_5p,mmu_mi_r_212_3p,mmu_mi_r_2139,mmu_mi_r_218_5p,mmu_mi_r_2183,mmu_mi_r_221_5p,mmu_mi_r_26a_5p,mmu_mi_r_26b_5p,mmu_mi_r_290b_3p,mmu_mi_r_291b_3p,mmu_mi_r_292a_3p,mmu_mi_r_295_3p,mmu_mi_r_299b_5p,mmu_mi_r_301b_3p,mmu_mi_r_302d_3p,mmu_mi_r_3063_3p,mmu_mi_r_3066_5p,mmu_mi_r_3074_2_3p,mmu_mi_r_3083_5p,mmu_mi_r_3084_5p,mmu_mi_r_3085_5p,mmu_mi_r_3087_5p,mmu_mi_r_3098_5p,mmu_mi_r_3099_3p,mmu_mi_r_3099_5p,mmu_mi_r_30a_3p,mmu_mi_r_30c_5p,mmu_mi_r_30e_3p,mmu_mi_r_3104_5p,mmu_mi_r_3106_5p,mmu_mi_r_3108_3p,mmu_mi_r_327,mmu_mi_r_329_3p,mmu_mi_r_331_3p,mmu_mi_r_338_3p,mmu_mi_r_339_5p,mmu_mi_r_340_5p,mmu_mi_r_342_3p,mmu_mi_r_344e_3p,mmu_mi_r_3474,mmu_mi_r_3475_3p,mmu_mi_r_34a_3p,mmu_mi_r_34c_5p,mmu_mi_r_3535,mmu_mi_r_361_3p,mmu_mi_r_374b_5p,mmu_mi_r_375_3p,mmu_mi_r_376b_3p,mmu_mi_r_382_3p,mmu_mi_r_384_3p,mmu_mi_r_384_5p,mmu_mi_r_3961,mmu_mi_r_3964,mmu_mi_r_3970,mmu_mi_r_410_3p,mmu_mi_r_411_5p,mmu_mi_r_412_5p,mmu_mi_r_421_3p,mmu_mi_r_429_5p,mmu_mi_r_449a_5p,mmu_mi_r_450a_2_3p,mmu_mi_r_452_3p,mmu_mi_r_465d_5p,mmu_mi_r_466q,mmu_mi_r_471_5p,mmu_mi_r_485_5p,mmu_mi_r_486a_3p,mmu_mi_r_486b_3p,mmu_mi_r_490_5p,mmu_mi_r_493_5p,mmu_mi_r_496a_5p,mmu_mi_r_497a_5p,mmu_mi_r_500_5p,mmu_mi_r_505_3p,mmu_mi_r_5098,mmu_mi_r_5104,mmu_mi_r_5106,mmu_mi_r_511_3p,mmu_mi_r_5118,mmu_mi_r_5119,mmu_mi_r_5124b,mmu_mi_r_5132_3p,mmu_mi_r_532_5p,mmu_mi_r_546,mmu_mi_r_5616_5p,mmu_mi_r_5618_3p,mmu_mi_r_5623_3p,mmu_mi_r_582_3p,mmu_mi_r_590_5p,mmu_mi_r_615_3p,mmu_mi_r_615_5p,mmu_mi_r_6237,mmu_mi_r_6244,mmu_mi_r_6344,mmu_mi_r_6361,mmu_mi_r_6371,mmu_mi_r_6372,mmu_mi_r_6383,mmu_mi_r_6386,mmu_mi_r_6405,mmu_mi_r_6407,mmu_mi_r_6409,mmu_mi_r_6410,mmu_mi_r_6715_3p,mmu_mi_r_687,mmu_mi_r_688,mmu_mi_r_6900_3p,mmu_mi_r_6904_5p,mmu_mi_r_6907_3p,mmu_mi_r_6911_5p,mmu_mi_r_6917_3p,mmu_mi_r_6918_3p,mmu_mi_r_6926_5p,mmu_mi_r_6928_3p,mmu_mi_r_6932_5p,mmu_mi_r_6935_3p,mmu_mi_r_6937_3p,mmu_mi_r_6938_3p,mmu_mi_r_6939_3p,mmu_mi_r_694,mmu_mi_r_6949_3p,mmu_mi_r_6949_5p,mmu_mi_r_6954_3p,mmu_mi_r_6966_3p,mmu_mi_r_6966_5p,mmu_mi_r_6967_5p,mmu_mi_r_6969_3p,mmu_mi_r_6970_3p,mmu_mi_r_6971_5p,mmu_mi_r_6974_5p,mmu_mi_r_6976_5p,mmu_mi_r_6984_3p,mmu_mi_r_6987_5p,mmu_mi_r_6991_3p,mmu_mi_r_6992_3p,mmu_mi_r_6996_3p,mmu_mi_r_6997_3p,mmu_mi_r_6999_3p,mmu_mi_r_7002_5p,mmu_mi_r_7005_3p,mmu_mi_r_7007_5p,mmu_mi_r_7016_3p,mmu_mi_r_7019_5p,mmu_mi_r_7020_3p,mmu_mi_r_7029_5p,mmu_mi_r_7033_3p,mmu_mi_r_7039_5p,mmu_mi_r_704,mmu_mi_r_7046_5p,mmu_mi_r_7054_3p,mmu_mi_r_7056_3p,mmu_mi_r_7064_5p,mmu_mi_r_7065_3p,mmu_mi_r_7082_3p,mmu_mi_r_7087_3p,mmu_mi_r_7091_5p,mmu_mi_r_7093_3p,mmu_mi_r_7094_1_5p,mmu_mi_r_7094b_2_5p,mmu_mi_r_7210_5p,mmu_mi_r_7212_5p,mmu_mi_r_7215_5p,mmu_mi_r_7216_5p,mmu_mi_r_7219_5p,mmu_mi_r_7224_3p,mmu_mi_r_7225_5p,mmu_mi_r_7233_5p,mmu_mi_r_7239_5p,mmu_mi_r_7240_5p,mmu_mi_r_7243_5p,mmu_mi_r_743b_3p,mmu_mi_r_758_5p,mmu_mi_r_761,mmu_mi_r_764_3p,mmu_mi_r_7652_3p,mmu_mi_r_7652_5p,mmu_mi_r_7661_3p,mmu_mi_r_7664_3p,mmu_mi_r_7664_5p,mmu_mi_r_7668_3p,mmu_mi_r_7671_3p,mmu_mi_r_7673_3p,mmu_mi_r_7682_5p,mmu_mi_r_7687_5p,mmu_mi_r_7688_5p,mmu_mi_r_7689_5p,mmu_mi_r_7a_5p,mmu_mi_r_7b_3p,mmu_mi_r_8098,mmu_mi_r_8099,mmu_mi_r_8106,mmu_mi_r_8107,mmu_mi_r_8109,mmu_mi_r_8110,mmu_mi_r_8116,mmu_mi_r_871_3p,mmu_mi_r_875_5p,mmu_mi_r_879_5p,mmu_mi_r_880_3p,mmu_mi_r_881_3p,mmu_mi_r_9_5p,mmu_mi_r_92b_3p,mmu_mi_r_93_3p,mmu_mi_r_935,mmu_mi_r_98_3p,mmu_mi_r_98_5p,mmu_mi_r_99b_5p,mmu_pi_r_000159_gb_dq539904_mus_musculus_2_73668844_73668871_plus,mmu_pi_r_000159_gb_dq539904_mus_musculus_6_87962871_87962898_minus,mmu_pi_r_000616_gb_dq540965_mus_musculus_2_5296571_5296602_minus,mmu_pi_r_000616_gb_dq540965_mus_musculus_3_5843413_5843444_plus,mmu_pi_r_000691_gb_dq541218_mus_musculus_8_126462165_126462190_minus,mmu_pi_r_001570_gb_dq543701_mus_musculus_3_5843412_5843441_plus,mmu_pi_r_009321_gb_dq684704_mus_musculus_7_72988380_72988411_plus,mmu_pi_r_009467_gb_dq684969_mus_musculus_7_73682766_73682794_minus,mmu_pi_r_009574_gb_dq685137_mus_musculus_15_59111741_59111771_plus,mmu_pi_r_010309_gb_dq686298_mus_musculus_1_161177076_161177096_plus,mmu_pi_r_013503_gb_dq691233_mus_musculus_8_24061664_24061692_plus,mmu_pi_r_017405_gb_dq696996_mus_musculus_11_65550994_65551015_minus,mmu_pi_r_025576_gb_dq708952_mus_musculus_x_6405415_6405436_minus,mmu_pi_r_032865_gb_dq719430_mus_musculus_2_116876592_116876614_plus,mmu_pi_r_032974_gb_dq719597_mus_musculus_4_130021751_130021778_plus,mmu_pi_r_038323_gb_pi_rna_t34_mus_musculus_13_44880547_44880577_minus,mmu_pi_r_038328_gb_pi_rna_t47_mus_musculus_19_13121850_13121879_plus,mmu_pi_r_038328_gb_pi_rna_t47_mus_musculus_6_3151091_3151120_plus,mmu_pi_r_038328_gb_pi_rna_t47_mus_musculus_x_23165477_23165506_plus

### Associations between lipids in the hypothalamus and amygdala and behavioral and cognitive measures

**Figure 5:**
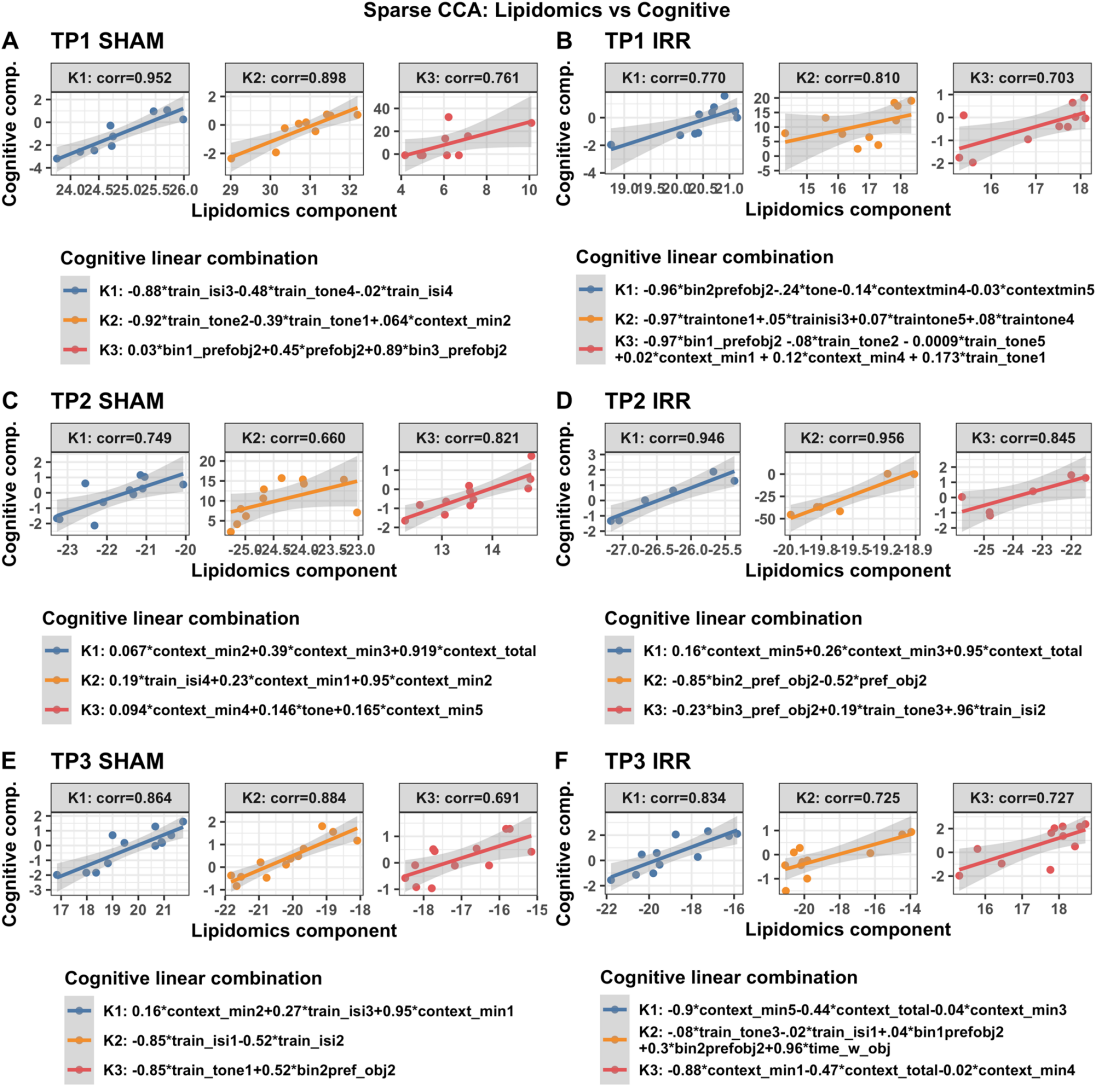
Association between select lipids and cognitive measures. Results from sCCA analyses of lipidomics and cognitive measures at time points 1, 2 and 3. Two analyses for each time point are shown: F1 irradiated mice (top row) and F1 sham mice (bottom row). The top 3 canonical vector components (linear combinations within each data set denoted K1, K2, K3) with the strongest correlations are detected and presented in the three panels of each analyses. For each component, the explicit linear combinations of cognitive measures (form_out) and lipidomics (form_lipid) are shown in the table below the scatter plots. Pearson’s correlation for each component is shown above each panel as well as in the table. The scatter plots show the linear combination for lipidomics on the x-axis and the linear combination of cognitive measure component in the Y axis, along with the best fit linear line and confidence band (grey shaded area).

Distinct and overlapping lipids in sham and irradiated mice were correlated with cognitive measures at the first (TP1, Fig. 5A,B, Supplementary Fig. 16), second (TP2, Fig. 5C,D, Supplementary Fig. 17), and third (TP3, Fig. 5E,F, Supplementary Fig. 18) time points. Details about the lipids involved in these relationships with behavioral and cognitive measures are illustrated in Tables 3 and 4.

**Table 3:** sCCA linear component formulas for lipidomics vs cognitive and behavioral measures.

Outcome type	Time Point	Irradiation Group	sCCA component number (K1–3)	Correlation	Outcome linear component	Lipidomics linear component
Cognitive	TP1	SHAM	K1	0.952	− 0.878*train_isi3 + − 0.478*train_tone4 + − 0.018*train_isi4	0.245 × 451_305_A + − 0.186 × 1281_79_A + 0.235 × 1544_847_A + 0.922 × 290_087_H
Cognitive	TP1	SHAM	K2	0.898	− 0.917*train_tone2 + − 0.393*train_tone1 + 0.064*context_minute2	0.651 × 1544_847_A + 0.731 × 419_255_A + 0.206 × 886_546_A
Cognitive	TP1	SHAM	K3	0.761	0.033*bin_1_preference_obj_2 + 0.448*preference_obj_2 + 0.893*bin_3_preference_obj_2	0.828 × 493_165_A + 0.079 × 581_18_A + 0.144 × 708_574_A + − 0.537 × 1544_849_A
Cognitive	TP1	IRR	K1	0.770	− 0.959*bin_2_preference_obj_2 + − 0.243*tone + − 0.139*context_minute4 + − 0.033*context_minute5	x290_087_A
Cognitive	TP1	IRR	K2	0.810	− 0.972*train_tone1 + 0.054*train_isi3 + 0.068*train_tone5 + 0.077*train_tone4 + 0.204*train_isi4	x290_086_H
Cognitive	TP1	IRR	K3	0.703	− 0.974*bin_1_preference_obj_2 + − 0.076*train_tone2 + − 0.009*train_tone5 + 0.021*context_minute1 + 0.122*context_minute4 + 0.173*train_tone1	x265_147_A
Cognitive	TP2	SHAM	K1	0.749	0.067context_minute2 + 0.39context_minute3 + 0.919*context_total	− 0.276 × 1253_773_A + − 0.961 × 581_309_A
Cognitive	TP2	SHAM	K2	0.660	0.191*train_isi4 + 0.23*context_minute1 + 0.954*context_minute2	− 0.276 × 291_091_A + − 0.961 × 581_183_A
Cognitive	TP2	SHAM	K3	0.821	0.094*context_minute4 + 0.146*tone + 0.165*context_minute5 + 0.971*train_tone3	0.961 × 290_087_A + − 0.276 × 419_256_A
Cognitive	TP2	IRR	K1	0.946	0.164context_minute5 + 0.259context_minute3 + 0.952*context_total	− 0.385 × 291_091_A + − 0.923 × 581_184_A
Cognitive	TP2	IRR	K2	0.956	− 0.853bin_2_preference_obj_2 + − 0.522preference_obj_2	− 0.971 × 290_088_A + − 0.153 × 581_183_A + 0.184 × 886_553_A
Cognitive	TP2	IRR	K3	0.845	− 0.227bin_3_preference_obj_2 + 0.193train_tone3 + 0.955*train_isi2	− 0.385 × 1544_867_A + − 0.923 × 835_283_A
Cognitive	TP3	SHAM	K1	0.864	0.156*context_minute2 + 0.268*train_isi3 + 0.951*context_minute1	0.04 × 290_087_A + 0.999 × 581_183_A
Cognitive	TP3	SHAM	K2	0.884	− 0.853*train_isi1 + − 0.522*train_isi2	− 0.04 × 1604_89_H + − 0.999 × 1863_995_H
Cognitive	TP3	SHAM	K2	0.691	− 0.853train_tone1 + 0.522bin_2_preference_obj_2	− 0.04 × 1544_867_H + − 0.999 × 726_589_H
Cognitive	TP3	IRR	K1	0.834	− 0.896context_minute5 + − 0.442context_total + − 0.036*context_minute3	− 1 × 283_264_H
Cognitive	TP3	IRR	K2	0.725	− 0.081*train_tone3 + − 0.021*train_isi1 + 0.035*bin_1_preference_obj_2 + 0.283*bin_2_preference_obj_2 + 0.955*time_with_objects	− 1 × 581_183_A
Cognitive	TP3	IRR	K3	0.727	− 0.879context_minute1 + − 0.476context_total + − 0.019*context_minute4	1 × 888_641_H
Behavioral	TP1	SHAM	K1	0.555	1*ofd1_fecal	− 1 × 290_086_H
Behavioral	TP1	SHAM	K2	0.444	1*fc_cued_percent_time_frz_baseline	1 × 290_087_H
Behavioral	TP1	SHAM	K3	0.509	1*nod1_fecal	1 × 265_147_A
Behavioral	TP1	IRR	K1	0.848	− 1*nod2_fecal	1 × 290_087_H
Behavioral	TP1	IRR	K2	0.404	− 1*fc_cued_percent_time_frz_baseline	− 1 × 290_087_A
Behavioral	TP1	IRR	K3	0.659	− 1*nod1_fecal	1 × 290_087_H
Behavioral	TP2	SHAM	K1	0.638	1*nod1_fecal	− 0.978 × 1253_773_A + − 0.0592 × 1544_867_A + − 0.2 × 581_309_A
Behavioral	TP2	SHAM	K2	0.765	− 1*fc_cued_percent_time_frz_baseline	− 0.157 × 888_641_A + 0.0975 × 1544_867_H + 0.983 × 581_183_H
Behavioral	TP2	SHAM	K3	0.625	− 1*ofd1_fecal	0.276 × 1253_773_A + 0.961 × 581_309_A
Behavioral	TP2	IRR	K1	0.735	1*ofd1_fecal	− 0.152 × 1544_867_A + 0.971 × 1863_996_A + − 0.185 × 888_642_A
Behavioral	TP2	IRR	K2	0.528	1*ofd2_fecal	0.385 × 1544_87_A + 0.923 × 2127_061_A
Behavioral	TP2	IRR	K3	0.697	− 1*nod2_fecal	0.0159 × 581_184_A + − 0.933 × 682_283_A + − 0.358 × 710_314_A
Behavioral	TP3	SHAM	K1	0.733	− 1*nod2_fecal	− 0.04 × 1544_867_A + − 0.999 × 1835_964_A
Behavioral	TP3	SHAM	K2	0.593	1*fc_cued_percent_time_frz_baseline	0.04 × 1544_867_H + 0.999 × 726_589_H
Behavioral	TP3	SHAM	K3	0.603	1*nod1_fecal	0.999 × 1835_964_A + − 0.04 × 1863_995_H
Behavioral	TP3	IRR	K1	0.678	− 1*ofd1_fecal	1 × 888_641_H
Behavioral	TP3	IRR	K2	0.625	− 1*nod1_fecal	− 1 × 283_264_H
Behavioral	TP3	IRR	K3	0.618	1*nod2_fecal	− 1 × 581_183_H

The top 3 sCCA components are shown for sCCA analyses relating behavioral or cognitive measures with lipidomics data. Lipid masses are specified with “_A” denoting amygdala tissue and “_H” denoting hippocampus tissue.

**Table 4:** Lipids correlated with cognitive and behavioral measures in sCCA analyses. Lipids found to be correlated with behavioral and cognitive measures in all sCCA analyses relating behavioral or cognitive measures with lipidomics data, with corresponding set of behavioral or cognitive measures found to be correlated with each individual lipid. Lipid masses are specified with “_A” denoting amygdala tissue and “_H” denoting hippocampus tissue.

Mass	Lipid_name	Cognitive or behavioral associations
265.147	x265_147_A	bin_1_preference_obj_2,context_minute1,context_minute4,nod1_fecal,train_tone1,train_tone2,train_tone5
283.264	x283_264_H	context_minute1,context_minute3,context_minute5,context_total,nod1_fecal,train_isi4
290.086	x290_086_H	nod2_fecal,ofd1_fecal,train_isi3,train_isi4,train_tone1,train_tone4,train_tone5
290.086	x290_086_A	context_minute2,ofd2_fecal,train_isi1,train_tone2,train_tone5
290.087	x290_087_H	bin_1_preference_obj_2,bin_3_preference_obj_2,context_minute4,fc_cued_percent_time_frz_baseline,nod1_fecal,nod2_fecal,ofd1_fecal,ofd2_fecal,train_isi3,train_isi4,train_tone4,train_tone5
290.087	x290_087_A	bin_2_preference_obj_2,context_minute1,context_minute2,context_minute4,context_minute5,fc_cued_percent_time_frz_baseline,nod2_fecal,tone,train_isi2,train_isi3,train_tone3
290.088	x290_088_A	bin_2_preference_obj_2,preference_obj_2
419.255	x419_255_A	context_minute2,train_tone1,train_tone2
419.256	x419_256_A	context_minute4,context_minute5,tone,train_tone3
451.305	x451_305_A	train_isi3,train_isi4,train_tone4
581.309	x581_309_A	context_minute2,context_minute3,context_total,nod1_fecal,ofd1_fecal
708.574	x708_574_A	bin_1_preference_obj_2,bin_3_preference_obj_2,preference_obj_2
710.314	x710_314_A	nod2_fecal
726.589	x726_589_H	bin_2_preference_obj_2,fc_cued_percent_time_frz_baseline,train_tone1
886.546	x886_546_A	context_minute2,train_tone1,train_tone2
886.553	x886_553_A	bin_2_preference_obj_2,preference_obj_2
888.641	x888_641_A	fc_cued_percent_time_frz_baseline,context_minute1,context_minute4,context_total,ofd1_fecal
1544.87	x1544_87_A	ofd2_fecal,train_isi1,train_tone5
1544.847	x1544_847_A	context_minute2,train_isi3,train_isi4,train_tone1,train_tone2,train_tone4
1544.849	x1544_849_A	bin_1_preference_obj_2,bin_3_preference_obj_2,preference_obj_2
1544.867	x1544_867_A	bin_1_preference_obj_2,bin_3_preference_obj_2,context_minute1,nod1_fecal,nod2_fecal,ofd1_fecal,ofd2_fecal,time_with_objects,train_isi2,train_tone3
1544.867	x1544_867_H	bin_2_preference_obj_2,fc_cued_percent_time_frz_baseline,train_tone1
1604.89	x1604_89_H	train_isi1,train_isi2
1835.964	x1835_964_A	nod1_fecal,nod2_fecal
2127.061	x2127_061_A	ofd2_fecal

**Figure 6:**
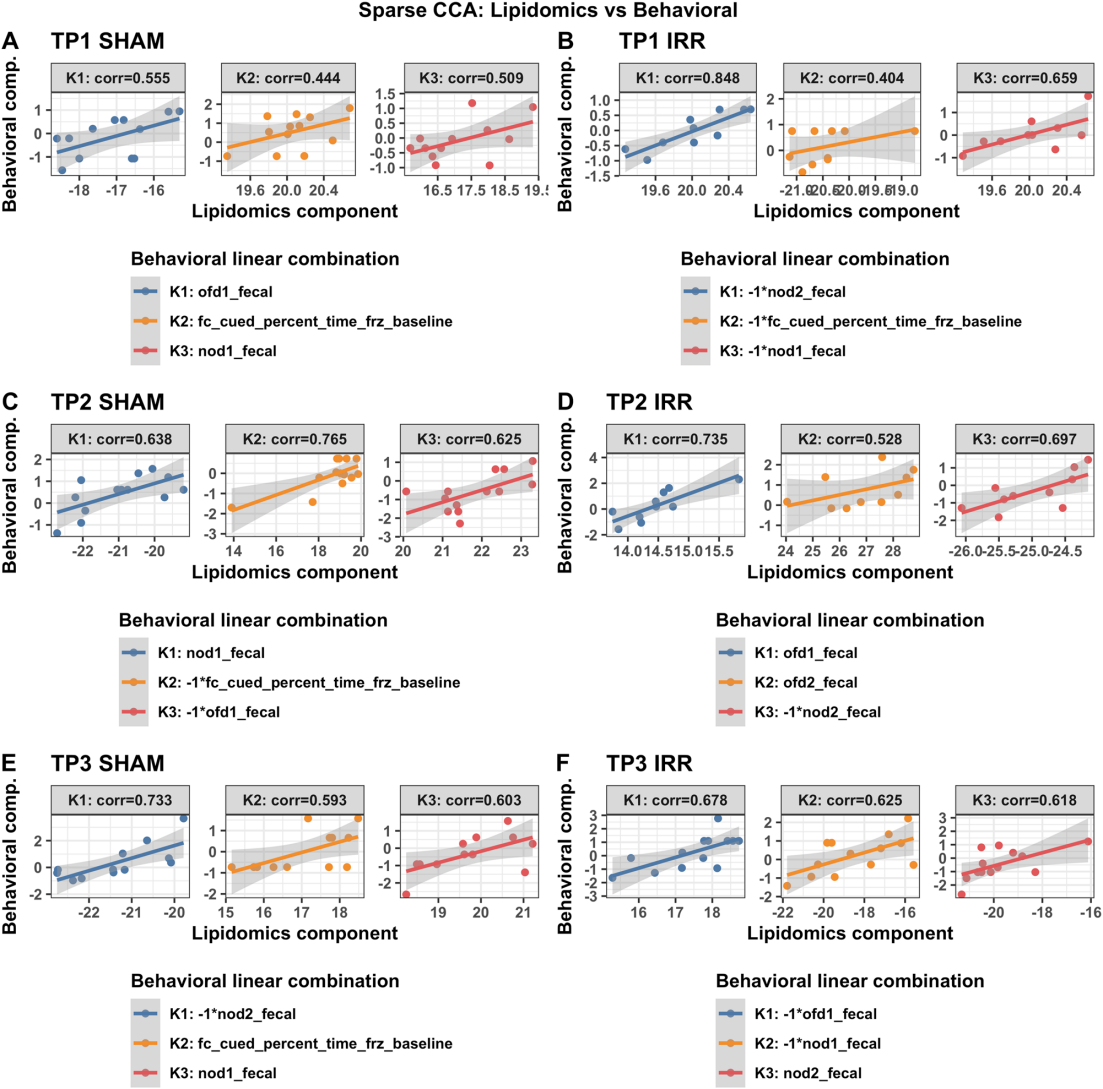
Association between select lipids and behavioral measures at TP1. Results from sCCA analyses of lipidomics and behavioral measures at time points 1, 2 and 3. Two analyses for each time point are shown: F1 irradiated mice (top row) and F1 sham mice (bottom row). The top 3 canonical vector components (linear combinations within each data set denoted K1, K2, K3) with the strongest correlations are detected and presented in the three panels of each analyses. For each component, the explicit linear combinations of behavioral measures (form_out) and lipidomics (form_lipid) are shown in the table below the scatter plots. Pearson’s correlation for each component is shown above each panel as well as in the table. The scatter plots show the linear combination for lipidomics on the x-axis and the linear combination of behavioral measure component in the Y axis, along with the best fit linear line and confidence band (grey shaded area).

Distinct and overlapping lipids in sham and irradiated mice were associated with behavioral measures at the first (TP1, Fig. 6A,B, Supplementary Fig. 19), second (TP2, Fig. 6C,D, Supplementary Fig. 20), and third (TP3, Fig. 6E,F, Supplementary Fig. 21) time points. Details about the lipids involved in these relationships are illustrated in Tables 3 and 4.

In purple, learning measures and in light blue memory measures are indicated. In green, fecal boli-related measures are indicated. Five lipids that are associated with behavioral and cognitive measures in Tables 3 and 4 were matched with lipids that were associated with plasma miRNAs in Table 2. For each of these five lipids, we ran an Ingenuity Pathway Analysis (Supplementary Reports 1–5). miRNAs associated with all five lipids revealed pathways involved in organismal injury and abnormalities, reproductive systems disease, cardiotoxicity, hepatotoxicity, and nephrotoxicity (Table 5, indicated in red). The miRNAs associated with all five shared lipids revealed pathways involved in cancer (Table 5, indicated in green). miRNAs associated with three lipids (*m/z* 451.305A, *m/z* 581.309A, and *m/z* 726.589A) revealed pathways involved in neurological disease and of those two lipids (*m/z* 451.305A and *m/z* 726.589A) revealed pathways involved in psychological disorders (Table 5, indicated in purple). miRNAs associated with 3 lipids (*m/z* 581.309A, *m/z* 710.314A, and *m/z* 888.641A) were associated with inflammatory disease (Table 5, indicated in brown). Of those, miRNAs associated with 1 lipid (*m/z* 710.314A) was associated with immunological disease and miRNAs associated with 1 lipid was associated with inflammatory response (*m/z* 888.641A). The amygdala lipids identified at *m/z* 451.305, and *m/z* 581.309 are both lysophospholipids which are important second-messenger molecules that can regulate intracellular signaling pathways that are involved in several physiological and pathological functions such as, inflammation (increased eicosanoid production), angiogenesis, nervous system regulation, atherosclerosis and tumorigenesis^30^. Lysophospholipids have been proposed to be indicators of mitochondrial dysfunction. The lipid at *m/z* 710.314 was isobaric for two lipids, sphingomyelin which makes up the myelin sheath around neurons and/or phosphatidic acid (PA) a second messenger lipid that has many signaling functions such as cell growth, proliferation, reproduction and responses to hormones and stress^31^. PA also has a connection to the mitochondria as it inhibits mitochondrial division and stimulates mitochondrial outer membrane fusion. Finally, the amygdala lipid at *m/z* 888.641 was also isobaric for two lipids, an unsaturated phosphatidylinositol with 38 carbons in its tail and/or an unsaturated triacylglycerol (TG) with 55 carbons composing its three fatty acid tails. Phosphatidylinositol is the precursor for all phosphoinositides which are second messengers, regulators of cytoskeleton, enzymes and ion channels and also serve to dock proteins to membranes^32^. Additionally, inositol lipids are enriched in brain and the phosphorylated versions are thought to play a central role in neuronal function and disease mainly in their roles of intracellular vesicular trafficking, and membrane turnover which ultimately leads to build-up of cellular components resulting in neuronal degradation^33^. The other isobaric lipid at *m/z* 888.641 is an unsaturated triglyceride (TG). TGs can readily cross the blood–brain barrier and inhibit leptin and insulin receptors leading to cognitive disfunction and Type 2 diabetes^34^. TGs are also a major energy source after oxidation as they provide 2X as much energy as the compared to carbohydrates.

**Table 5:** Summary of IPA analyses of pathways of miRNAs associated with 6 lipids correlated with behavioral and cognitive measures in Table 3.

	x451_305A	X581_309A	X710_314A	X726_589A	x888_641A
Organismal injury and abnormalities	x	x	x	x	x
Reproductive systems disease	x	x	x	x	x
GI disease					x
Respiratory disease					x
Psychological disorder	x			x	
Neurological disease	x	x		x	
Immunological disease			x		
Inflammatory disease		x	x		x
Inflammatory response					x
Cancer	x	x	x	x	x
Cardiotoxicity	x	x	x	x	x
Hepatotoxicity	x	x	x	x	x
Nephrotoxicity	x	x	x	x	x

### Effects of ^28^Si ion irradiation revealed by the Lasso analysis

In general, C3H mice were more affected by the ^28^Si ion irradiation than BALB/c mice. In Lasso regression analyses where models were fit separately for irradiated mice and sham mice, many of the associations between input and response variables were not preserved between irradiation groups except the mouse breed, and many new associations were found in irradiated mice. Here, we highlight two Lasso coefficient groups: (1) groups where the coefficient magnitude is larger than 0.1 (for lipidomics) or 0.25 (for behavior output) and the term exists only for that same group and (2) groups where the association of input and response variables is preserved.

**Figure 7:**
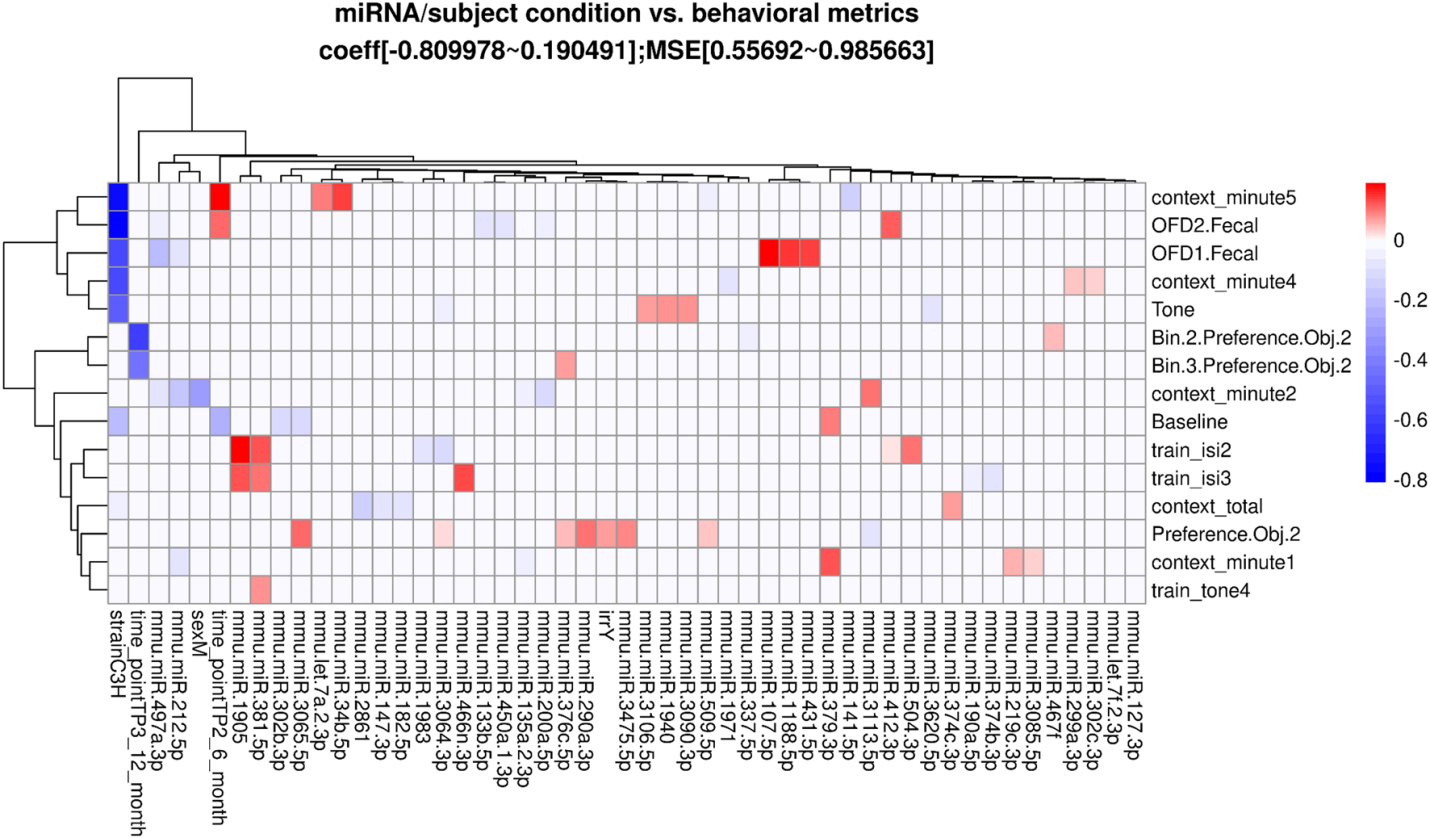
Heatmap of lasso coefficients describing associations between miRNA and condition to behavioral measure outcomes. Heatmap of magnitude of lasso regression coefficients with behavioral metrics as outcomes. Each row corresponds to a behavior outcome, with the independent predictors of miRNA, irradiation (irrY denotes irradiation yes) vs sham, sex (sexM denotes male vs female), strain (strainC3H), and time point (time_pointTP3, time_pointTP2 compared to TP1 reference group). Darker red denotes strong positive associations and darker blue denotes strong negative associations. In subtitle, “coeff” is the Lasso coefficient and the range of coefficient magnitudes; MSE denotes range of mean square error from cross-validation with 100 repeats.

The C3H sham group showed a negative correlation with freezing prior to the tone in the cued fear memory test (‘Baseline’, Fig. 7). In irradiated mice, this association was weaker (Supplementary Fig. 22). In addition, in irradiated mice we observed a new negative association with freezing during the tone in the cued fear memory test (‘Tone’) and with the number of fecal boli deposited during the first and second day of the objects recognition test (‘NOD1/2.Fecal’; Supplementary Fig. 22). Finally, in irradiated mice there was a stronger association with number of fecal boli during the second day of the open field (‘OFD2.Fecal’) and with freezing during the contextual fear memory test (‘context’).

The 12-month-old mice group was highly affected by the irradiation, showing more freezing during 4th and 5th minute of the contextual fear memory test (‘context_minute4(5)’) and less exploration of the novel object during the second and third 5-min bin of the object recognition memory test (‘Bin2(3).Preference.Obj.2’) compared to younger mice. In the 12-month-old group, levels of MAP2 in the cortex (‘Normalized.MAP2.Cortex’) was higher following irradiation (‘lasso coefficient’ in Supplementary Table 1).

Our analysis also revealed associations between specific miRNAs and contextual fear memory measures (*‘*context_minute*’ or ‘context_total’). For example, mmu-miR-369-5p and mmu-miR-379-3p were positively correlated with the first minute in the contextual fear memory test (‘context_minute1(4)’), whereas mmu-miR-429-5p was inversely proportional to freezing in the 4th and 5th minute of the contextual fear memory test (‘context_minute4(5)’. It is worthy to note that, in the miRNA enrichment analysis, the two over-represented miRNAs (mmu-miR-369-5p and mmu-miR-466o-3p) target the genes such as Foxn3, Tmem161b, Ccdc169, and Hist1h1d, and also they induce ‘syncytiotrophoblast cell differentiation involved in labyrinthine layer development’ and ‘negative regulation of amacrine cell differentiation’, and ‘nuclear migration along microtubule’. mmu-miR-369-5p was modulated in brain 24 h following moderate traumatic brain injury^35^. mmu-miR-379-3p is considered to function as a tumor suppressor gene, for example for gastric cancers^36^.

The mmu-miR-466o-3p was positively correlated with the levels of CD68 in the amygdala (‘Normalized.CD68.Amygdala’), whereas the positive correlation of mmu-miR-466n-5p with the levels of CD68 in the hypothalamus (‘Normalized.CD68.Hypothalamus’) was seen in sham-irradiated mice but not in irradiated mice.

In sham-irradiated mice, the mmu-miR-467d-3p was positively correlated with the number of fecal boli deposited in the open field ('OFD.Fecal') and also positively correlated with the levels of CD68 in the hypothalamus ('Normalized.CD68.Hypothalamus'). In irradiated mice, mmu-miR-466o-3p showed a moderate association with freezing in the contextual fear memory test (‘context_minute_total’) and a positive correlation with the levels of CD68 in the amygdala (‘Normalized.CD68.Amygdala’).

**Figure 8:**
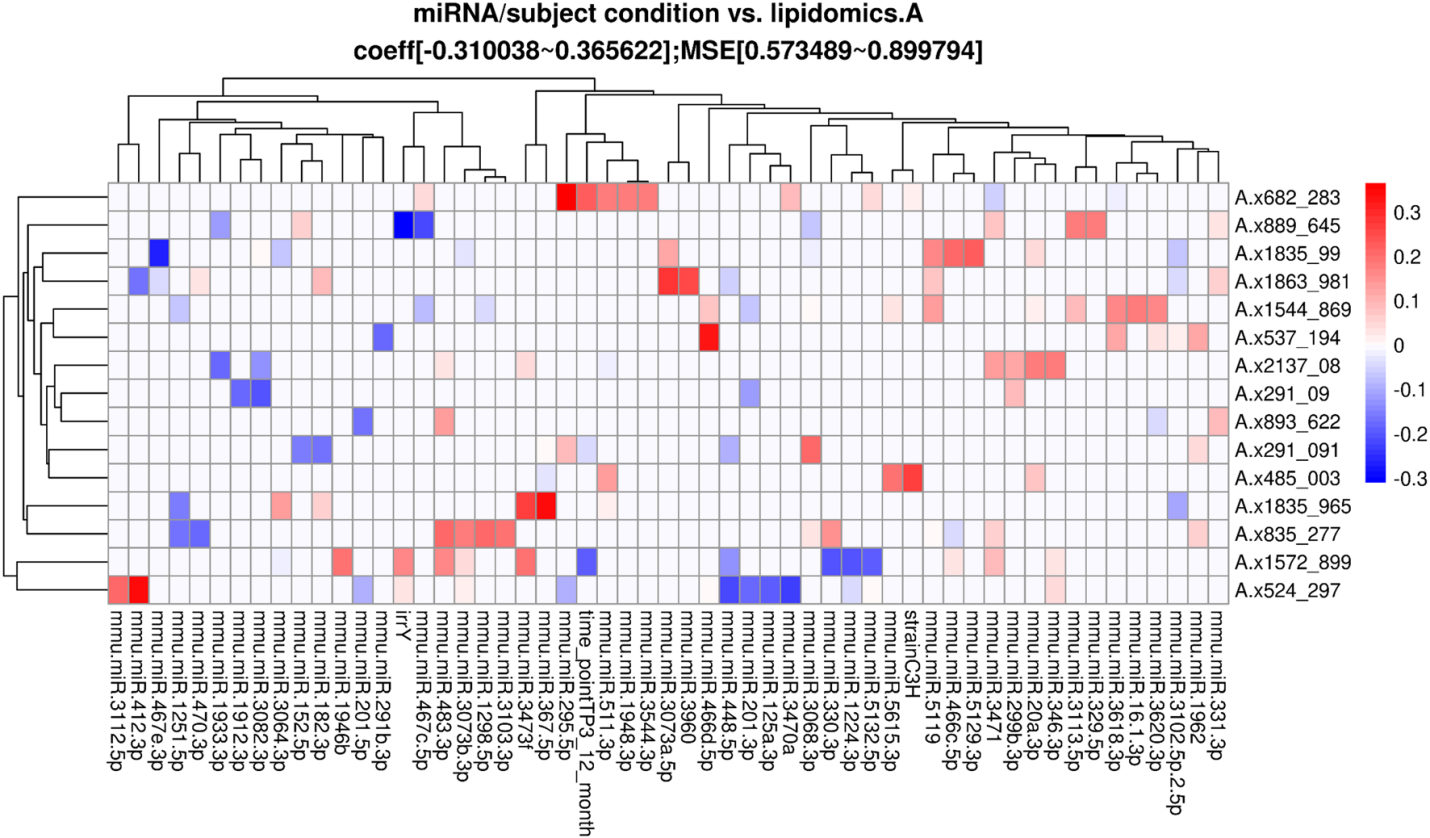
Heatmap of lasso coefficients describing associations between miRNA and condition to lipidomics in amygdala tissue. Heatmap of magnitude of lasso regression coefficients with behavioral metrics as outcomes. Each row corresponds to a behavior outcome, with the independent predictors of miRNA, irradiation (irrY denotes irradiation yes) vs sham, sex (sexM denotes male vs female), strain (strainC3H), and time point (time_pointTP3, time_pointTP2 compared to TP1 reference group). Darker red denotes strong positive associations and darker blue denotes strong negative associations. In subtitle, “coeff” is the Lasso coefficient and the range of coefficient magnitudes; MSE denotes range of mean square error from cross-validation with 100 repeats.

The irradiation effect was observed in the lipidomics analysis of amygdala tissue (A) (Fig. 8). With regard to cases where the lasso coefficient of the irradiation is higher than 0.1, in the amygdala lipid *m/z* 1572.899 (A.1572_899) identified as a sulfated globoside which is important in the composition of the neuronal myelin sheath and lipid *m/z* 708.578 (A.x708_578) identified as a phosphatidylglycerol (PG). PG is the precursor of cardiolipins which are essential for mitochondrial function. Increased ROS will oxidize cardiolipins and reduce mitochondrial function^37^. Lipids *m/z* 1253.775 (A.x1253_775) and *m/z* 889.645 (A.x889_645), identified as Phosphatidylserine and/or Phosphatidylcholine, were lower in the radiation condition.

The analysis also revealed relationships between lipids and miRNAs. The levels of the lipid *m/z* 889.645 in the amygdala (A.x889_645) were positively correlated with mmu-miR-3113-5p and mmu-miR-3471. These two miRNAs are associated with activation of adenylate cyclase activity GO0007190 and response to UV GO0009411. On the other hand, the other miRNAs, mmu-miR-1933-3p and mmu-miR-467c-5p were negatively correlated with lipid *m/z* 889.645 in the amygdala. The enrichment analysis revealed that these miRNAs are associated with vascular smooth muscle contraction.

The levels of the lipid *m/z* 1253.775 (A.x1253_775), identified as a cytidine diphosphate lipid (CDP1) in the amygdala was positively correlated with mmu-miR-201-5p and mmu-miR-3082-3p. Ms4a15 is a gene targeted by the miRNAs. These miRNAs are known to target Ms4a15, membrane-spanning 4-domains expressed in either Lung and blood, and the enrichment analysis revealed that they are involved with ‘response to mechanical stimulus GO0009612’. The other two miRNAs, miRNAs mmu-miR-3064-3p and mmu-miR-201-5p were positively correlated with levels of lipid *m/z* 1253.775 in the amygdala (A.x1253_775) and are associated with ‘response to lipid GO0033993’.

The levels of lipid *m/z* 1572.899 (A.x1572_899) identified as a fucosylated ganglioside in the amygdala was increased in the irradiated mice. Two miRNAs, mmu-miR-5132-5p and mmu-miR-466h-3p, were negatively correlated with levels of lipid *m/z* 1572.899 in the amygdala (A.x1572_899). The enrichment analysis revealed that over-represented expression of these miRNAs stimulates ‘expression T cell differentiation in thymus GO0033077’. Amygdala *m/z* 1572.899 lipid abundance in the irradiated mice was higher than the abundance in sham-irradiated mice. The two miRNAs are less expressed in the irradiated mice. Consequently, the irradiated mice’ T cell differentiation in thymus would be less activated. These data suggest that irradiation might suppress the expression T cell differentiation.

**Figure 9:**
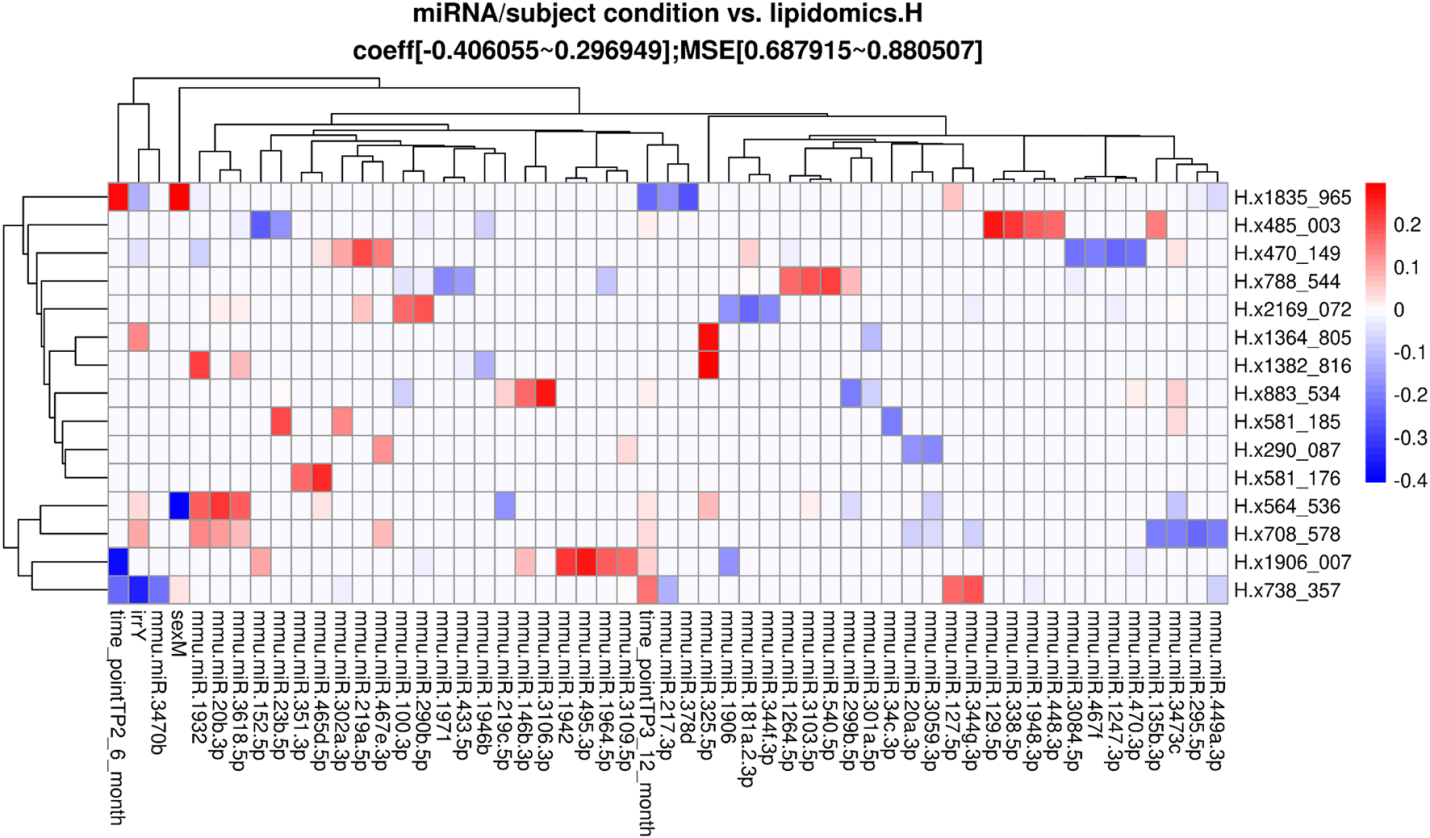
Heatmap of lasso coefficients describing associations between miRNA and condition to lipidomics in hippocampus. Heatmap of magnitude of lasso regression coefficients with behavioral metrics as outcomes. Each row corresponds to a behavior outcome, with the independent predictors of miRNA, irradiation (irrY denotes irradiation yes) vs sham, sex (sexM denotes male vs female), strain (strainC3H), and time point (time_pointTP3, time_pointTP2 compared to TP1 reference group). Darker red denotes strong positive associations and darker blue denotes strong negative associations. In subtitle, “coeff” is the Lasso coefficient and the range of coefficient magnitudes; MSE denotes range of mean square error from cross-validation with 100 repeats.

Irradiation affected lipids in the hippocampus (Fig. 9). The irradiated mice show a higher levels of the lipid *m/z* 1835.964 (H.x1835_964) in the hippocampus than the sham-irradiated mice. The lipid identified at *m/z* 1835.964 was the ganglioside Fuc-GM1(NeuGc) (d18:1(4E)/28:6(10Z,13Z,16Z,19Z,22Z,25Z), which is the mouse sialyated version of the human fucosylated brain ganglioside GM1. Fucosylated GM1 is a target for small cell lung cancer treatment (SCLC) as it is highly expressed in SCLC. Also, GM1 was has been linked with acute and chronic neuropathy syndromes^38^. The levels of this lipid in the hippocampus (H.x1835_964) was positively correlated with mmu-miR-467d-3p and mmu-miR-547-3p that are involved with photoreceptor connecting cilium and inner segment. The lipid *m/z* 708.578 in the hippocampus (H.x708_578) a phosphatidyl glycerol was inversely proportional to levels of mmu-miR-295-5p and mmu-miR-3473c expression abundance in plasma. The miRNAs are involved with multiple gene ontologies, cerebral cortex regionalization, lacrimal gland development, positive regulation of epithelial cell differentiation, and interkinetic nuclear migration.

The lipid *m/z* 979.325 (was not able to be assigned) in the hippocampus (H.x979_325) was higher in the irradiated mice. Two miRNAs, mmu-miR-495-3p and mmu-miR-450b-5p, were positively correlated with Hippocampus *m/z* 979.325. From the miRNA enrichment analysis, the miRNAs overrepresentation stimulate ubiquitin–ubiquitin ligase activity.

The levels of lipid *m/z* 738.357 (phosphatidyglycerol) in the hippocampus (H.x738_357) was lower in irradiated than sham-irradiated mice. Three miRNAs, mmu-miR-127-5p, mmu-miR-1943-5p, and mmu-miR-1954 are positively associated with levels of lipid *m/z* 738.357 in the hippocampus (H.x738_357). Their gene functions are attributed to the citrulline metabolic process GO0000052 and smooth endoplasmic reticulum GO0005790. On the other hand, mmu-miR-3470b and mmu-miR-1188-5p are negatively associated with levels of lipid *m/z* 738.357 in the hippocampus (H.x738_357). These two miRNA are associated with ‘protein import into the mitochondrial matrix’ and ‘carbonate dehydratase activity’. Consequently, the two gene functions are less active in the irradiated mice.

The levels of lipid *m/z* 1863.997 (a NeuGc sialylated and fucosylated GM1 ganglioside) the hippocampus (H.x1863_997) was lower in irradiated than sham-irradiated mice. The miRNAs mmu-miR-532-3p, mmu-miR-145a-5p and mmu-miR-432 were positively correlated with this lipid (H.x1863_997). The enrichment analysis revealed that these miRNAs are involved with multiple gene ontologies such as phenylethylamine metabolic process, primary amine oxidase activity, Histidine metabolism, lung epithelial cell differentiation, dopamine catabolic process, and microtubule cytoskeleton organization. The plasma levels of these three miRNAs were lower in the irradiated group and thus the corresponding gene functions are expected to be downregulated in the irradiated group.

## Discussion

This study shows associations between lipids in select brain regions, plasma miRNA, and cognitive measures following ^28^Si ion irradiation. Different but overlapping sets of miRNAs in plasma were found to be associated with cognitive measures in sham and irradiated mice at the three time points (Fig. 2, Supplementary Figs. 3 and 4). The radiation condition revealed pathways involved in neurodegenerative conditions (Supplementary Figs. 5–7). Remarkably, the pathways involved revealed by the radiation condition were similar for the first and third time point but distinct for the second time point. For example, at both TP1 and TP3, the radiation condition revealed pathways involved in Rett syndrome and Alzheimer’s disease (Supplementary Figs. 5 and 7). In contrast, at TP2, the radiation condition revealed pathways involved in Huntington disease and Down syndrome (Supplementary Fig. 6). The relationships revealed in the parental strain were distinct from those seen in the F2 mice but, as in the parental strains, the pathways revealed were distinct for TP1 and TP2. While at TP1, the F2 irradiated mice revealed pathways involved in mitochondria function and exosomes, at TP2 revealed pathways involved in Down syndrome and synapse. What is remarkable is that the TP2 pathways involved in Down syndrome were revealed in both the parental and F2 mice. These data indicate that time after radiation exposure is a critical variable in those relationships and that ^28^Si ion radiation exposure might increase the risk to develop neurodegenerative conditions later in life. At TP1, the radiation condition revealed pathways involved in multiple sclerosis as well. Women are more at risk than men to develop multiple sclerosis and in those with multiple sclerosis to develop macrovascular disease^39^ and Alzheimer’s disease^40, 41^. These data are consistent with the increased susceptibility of female mice to radiation-induced cognitive injury seen in C57BL/6J wild-type and human apolipoprotein E mice^42–44^ and increased long-term radiation sensitivity in women^45^. These results also indicate that conclusions that female mice would be immune to space radiation-induced long-term maladaptive responses should be carefully evaluated^46^. The radiation condition revealed not only pathways involved in neurodegenerative conditions but also those involved in cancer. For example, at TP1, the radiation condition revealed pathways involved in medulla blastoma in addition to Alzheimer’s disease.

This study shows associations between lipids in select brain regions, plasma miRNA, and behavioral measures following ^28^Si ion irradiation. Consistent with the pattern seen for the cognitive measures described above, the pathways involved revealed by the radiation condition were similar for the first and third time point but distinct for the second time point (Fig. 3, Supplementary Figs. 8 and 9). At TP1 and TP3, the radiation condition revealed pathways involved in Alzheimer’s disease, peripheral nerve injury, multiple sclerosis and an animal model of multiple sclerosis (EAE), and medulla blastoma (Supplementary Figs. 10–13). It is important to note that the majority of lipids that were correlated to miRNAs were highly active in involved in inflammatory responses, mitochondrial function, neuronal function and insulin responses. Besides peripheral nerve injuries, pathways involved in those conditions were also revealed when the cognitive measures were analyzed. In contrast, at TP2, the radiation condition did not reveal any of those pathways. These results for relationships of plasma miRNAs with cognitive and behavioral measures are intriguing considering the inversive relationship between Alzheimer’s disease and cancer^47^. These results suggest that prior radiation exposure increase risk of both Alzheimer’s disease and cancer.

Different but overlapping sets of miRNAs in plasma were associated with lipids in sham and irradiated mice at the three time points but, as for the associations of plasma miRNAs with cognitive and behavioral measures, the pathways revealed at TP1 and TP3 showed a similar pattern and therefore converging evidence (Fig. 4 and Supplementary Figs. 13–15). At TP1 and TP3, plasma miRNAs in irradiated mice were associated with lipids in the hypothalamus and amygdala enriched in pathways related to longevity and cancer that were not seen in sham-irradiated mice. At TP3, plasma miRNAs in sham-irradiated mice were associated with lipids in the hypothalamus and amygdala enriched in pathways related to Rett syndrome, seizures, Alzheimer’s disease, peripheral nerve injuries, and medulloblastoma. Considering that TP3 corresponds to middle-age, an age with the highest likelihood to successfully treat neurodegenerative conditions, these data suggest that biomarkers might be available that could be therapeutically pertinent. The fact that this is seen in the hypothalamus and amygdala is also intriguing considering that those might be brain areas showing early pathology in Alzheimer’s disease and related animal models^48^.

As supplementary analysis, Lasso regression was performed. Similar to the sCCA analysis described above, the Lasso regression analyses revealed distinct associations in sham-irradiated and irradiated mice with many new associations observed in irradiated mice. This analysis revealed that C3H mice were more affected by the ^28^Si ion irradiation than BALB/c mice. In C3H sham-irradiated mice there was a negative correlation with freezing prior to the tone in the cued fear memory test (Fig. 7). However, in irradiated mice, this association was weaker but there was a new negative association with freezing during the tone in the hippocampus-independent cued fear memory test and with freezing during the hippocampus-dependent contextual fear memory test (Supplementary Fig. 22).

The Lasso analyses also revealed relationship of radiation with levels of select biomarkers shown to be sensitive to effects of space radiation in previous studies (Supplementary Table 1). For example, levels of the dendritic marker MAP2 in the cortex were higher in irradiated than sham-irradiated mice at TP3. The elevated MAP2 levels are also seen with aging^49, 50^, X-ray irradiation^51^, and head-only ^137^Cs irradiation^52, 53^ and might be part of a compensatory response. We recognize that a limitation of this study is that BDNF, CD68, and MAP2 levels could not be analyzed for all brain regions at the three time points. Therefore, it is conceivable that additional relationships might have been revealed if those additional analyses would have been feasible.

Relationships were also revealed with the marker of activated microglia and neuroinflammation CD68. In sham-irradiated mice, the miRNA mmu-miR-466o-3p was positively correlated with the levels of CD68 in the amygdala, and the miRNAs mmu-miR-466n-5p and mmu-miR-467d-3p were positively correlated with the levels of CD68 in the hypothalamus (Figs. 8, 9). These data suggest that distinct miRNAs modulate neuroinflammation in an anatomical specific fashion.

The Lasso analysis revealed associations between specific miRNAs and specific time bins of the contextual fear memory test and that those relationships were distinct for sham-irradiated and irradiated mice (Fig. 7). While miRNAs mmu-miR-369-5p, mmu-miR-466o-3p, and mmu-miR-379-3p were positively correlated with the first minute in the contextual fear memory test, mmu-miR-429-5p was inversely proportional to freezing in the 4th and 5th minute of the contextual fear memory test. In irradiated mice, mmu-miR-466o-3p showed a moderate association with freezing in the contextual fear memory test (5th min and all 5 min combined; context_minute5/total). These data support an important role for plasma miRNAs in fine regulation of cognitive performance and the potential of using plasma miRNAs as biomarkers of brain function and therapeutic targets.

In conclusion, the biological effects induced by exposure to HZE in these experiments elicit changes in biologically important miRNAs and lipids that are involved in inflammation, mitochondrial function, cognitive disorders and other detrimental biological effects. The finding of the high levels of triacylglycerides (TGs) after HZE exposure which rapidly cross the blood brain barrier and bind to leptin and insulin receptors may play a role in insulin resistance. This HZE could be additive to previously published data of insulin resistance observed in astronauts after space flight^54^. Recent detailed space travel data also suggests several similar biological effects as reported here (increase in ROS, increase in immune response and impaired mitochondrial function) induced by space travel stressors (i.e., microgravity, increased CO_2_ levels, stress, etc.)^55^. The biological effects induced by HZE (GCR) will minimally be additive to the biological effects induced by space travel stressors. These data warrant further research to define effective countermeasures to ensure the safety of astronauts and success of their mission.

## Supplementary Information

Supplementary Information 1.

Supplementary Information 2.

Supplementary Information 3.

Supplementary Information 4.

Supplementary Information 5.

Supplementary Information 6.

Supplementary Information 7.

Supplementary Information 8.

Supplementary Information 9.

Supplementary Information 10.

Supplementary Information 11.

Supplementary Information 12.

Supplementary Information 13.

## References

[1] Raber J (2014). ^28^Silicon radiation-induced enhancement of synaptic plasticity in the hippocampus of naive and cognitively tested mice. Radiat. Res..

[2] Raber J, Marzulla T, Stewart B, Kronenberg A, Turker MS (2015). ^28^Silicon irradiation impairs contextual fear memory in B6D2F1 mice. Radiat. Res..

[3] Sheinerman K, Djukic A, Tsivinsky V, Umansky SR (2019). Brain-enriched microRNAs circulating in plasma as novel biomarkers for Rett syndrome. PLoS One.

[4] Sheinerman K (2017). Circulating brain-enriched microRNAs as novel biomarkers for detection and differentiation of neurodegenerative diseases. Alzheimers Res. Ther..

[5] Zhao Z (2019). Altered expression of microRNA-223 in the plasma of patients with first-episode schizophrenia and its possible relation to neuronal migration-related genes. Transl. Psychiatry.

[6] Sato J (2019). Brain metastasis-related microRNAs in patients with advanced breast cancer. PLoS One.

[7] Borghini A (2017). Low-dose exposure to ionizing radiation deregulates the brain-specific microRNA-134 in interventional cardiologists. Circulation.

[8] Qin X, Li L, Shu Q, Zhang Y, Wang Y (2018). Expression profile of plasma microRNAs and their roles in diagnosis of mild to severe traumatic brain injury. PLoS ONE.

[9] Yu Q (2015). Plasma microRNAs to predict the response of radiotherapy in esophageal squamous cell carcinoma patients. Am. J. Transl. Res..

[10] Raber J (1998). Detrimental effects of chronic hypothalamic-pituitary-adrenal axis activation. From obesity to memory deficits. Mol. Neurobiol..

[11] Magri F (2006). Stress and dementia: The role of the hypothalamicpituitary-adrenal axis. Aging Clin. Exp. Res..

[12] Swaab DF, Bao AM, Lucassen PJ (2005). The stress system in the human brain in depression and neurodegeneration. Ageing Res. Rev..

[13] Pape HC, Pare D (2010). Plastic synaptic networks of the amygdala for the acquisition, expression, and extinction of conditioned fear. Physiol. Rev..

[14] Ashwin C, Baron-Cohen S, Wheelwright S, O'Riordan M, Bullmore ET (2007). Differential activation of the amygdala and the 'social brain' during fearful face-processing in Asperger Syndrome. Neuropsychologia.

[15] Maren S, Quirk GJ (2004). Neuronal signalling of fear memory. Nat. Rev. Neurosci..

[16] Smidak R, Kofeler H, Hoeger H, Lubec G (2017). Comprehensive identification of age-related lipidome chnages in rat amygdala during normal aging. PLoS ONE.

[17] Wackerlig J (2020). Differences in hypothalamic lipid profiles of young and aged male rats with impaired and unimpaired spatial cognitive abilities and memory. Front. Aging Neurosci..

[18] Bolivar VJ, Caldarone BJ, Reilly AA, Flaherty L (2000). Habituation of activity in an open field: A survey of inbred strains and F1 hybrids. Behav. Genet..

[19] Anagnostaras S (2010). Automated assessment of pavlovian conditioned freezing and shock reactivity in mice using the video freeze system. Front. Behav. Neurosci..

[20] Almeida R, Pauling J, Sokol E, Hannibal-Bach H, Eising C (2015). Comprehensive lipidome analysis by shotgun lipidomics on a hybrid quadrupole-orbitrap-linear ion trap mass spectrometer. J. Am. Soc. Mass Spectrom..

[21] Rupansinghe T (2013). Lipidomics: Extraction protocols for biological matrices. Methods Mol. Biol. (Clifton, N.J.).

[22] He H (2007). Method for lipidomic analysis: p53 Expression modulation of sulfatide, ganglioside, and phospholipid composition of U87 MG glioblastoma cells. Anal. Chem..

[23] Witten D, Tibshirani R, Hastie T (2009). A penalized matrix decompisition, with applications to sparse principal components and cononical correlation analysis. Biostatistics.

[24] Wang H (2020). Finding the needle in a high-dimensional haystack: Canonical correlation analysis for neuroscientists. Neuroimage.

[25] Witten, D. & Tibshirani, R. Penalized Multivariate Analysis. R package version 1.2.1. (Accessed 5 July 2021). https://CRAN.R-project.org/package=PMA (2020).

[26] Peterson R (2019). Ordered quantile normalization: A semiparametric transformation built for the cross-validation era. J. Appl. Stat..

[27] Tibshirani R (1996). Regression shirnkage and selection via the lasso. J. R. Stat. Soc. B.

[28] Friedman J, Hastie T, Tibshirani R (2010). Regularization paths for generalized linear models via coordinate. J. Stat. Softw..

[29] Kern F (2020). miEAA 2.0: Integrating multi-species microRNA enrichment analysis and workflow management systems Nuc. Acids Res..

[30] Airfin S, Falasca M (2016). Lysophosphatidylinositol signalling and metabolic diseases. Metabolites.

[31] Wang X, Devaiah S, Zhang W, Welti R (2006). Signaling functions of phosphatidic acid. Prog. Lipid Res..

[32] Cockroft S, Carvou N (2007). Biochemical and biological functions of class I phosphatidylinositol transfer proteins. Biochim. Biophys. Acta.

[33] Raghu P, Joseph A, Krishnan H, Singh P, Saha S (2019). Phosphoinositides: Regulators of nervous system function in health and disease. Front. Mol. Neurosci..

[34] Banks W (2018). Triglycerides cross the blood–brain barrier and induce central leptin and insulin receptor resistance. Int. J. Obes..

[35] Chandran R (2017). Differential expression of microRNAs in the brains of mice subjected to increasing grade of mild traumatic brain injury. Brain Inj..

[36] Xu M, Qin S, Cao F, Ding S, Li M-H (2017). MicroRNA-379 inhibits metastasis and epithelial–mesenchymal transition via targeting FAK/AKT signaling in gastric cancer. Int. J. Oncol..

[37] Chen W-W, Chao J-J, Chang W-H, Chan J-F, Hsu Y-HH (2018). Phosphatidylglycerol incorporates into cardiolipin to improve mitochondrial activity and inhibits inflammation. Sci. Rep..

[38] Daniotti J, Vilcaes A, Torres Demichelis V, Ruggiero F, Rodriguez-Walker M (2013). Glycosylation of glycolipids in cancer: Basis for development of novel therapeutic approaches. Front. Oncol..

[39] Palladino R, Marrie R, Majeed A, Chataway J (2020). Evaluating the risk of macrovascualr events and mortality among people with multiple sclerosis in England. JAMA Neurol..

[40] Farrer LA (1997). Effects of age, sex, and ethnicity on the association between apolipoprotein E genotype and Alzheimer disease. A meta-analysis. J. Am. Med. Assoc..

[41] Podcasy J, Epperson C (2016). Considering sex and gender in Alzheimer disease and other dementias. Dialog. Clin. Neurosci..

[42] Villasana L, Acevedo S, Poage C, Raber J (2006). Sex- and ApoE Isoform-dependent effects of radiation on cognitive function. Radiat. Res..

[43] Villasana LE, Benice TS, Raber J (2011). Long-term effects of 56Fe irradiation on spatial memory of mice: Role of sex and apolipoprotein E isoform. Int. J. Radiat. Oncol. Biol. Phys..

[44] Villasana L, Rosenberg J, Raber J (2010). Sex-dependent effects of 56Fe irradiation on contextual fear conditioning in C57BL/6J mice. Hippocampus.

[45] Narendran N, Luzhna L, Kovalchuk O (2019). Sex difference of radiation response in occupational and accidental exposure. Front. Genet..

[46] Krukowski K (2018). Female mice are protected from space-radiation-induced maladaptive responses. Brain Behav. Immun..

[47] Okereke O, Meadows M-E (2019). More evidence of an inverse association between cancer and Alzheimer’s disease. JAMA Netw. Open.

[48] Canter R (2019). 3D mapping reveals network-specific amyloid progression and subcortical susceptibility in mice. Commun. Biol..

[49] Benice T, Rizk A, Pfankuch T, Kohama S, Raber J (2006). Sex-differences in age-related cognitive decline in C57BL/6J mice associated with increased brain microtubule-associated protein 2 and synaptophysin immunoreactivity. Neuroscience.

[50] Haley G, Kohama S, Urbanski H, Raber J (2010). Age-related decreases in SYN levels associated with increases in MAP-2, apoE, and GFAP levels in the rhesus nacaque prefrontal cortex and hippocampus. Age.

[51] Olsen RHJ, Marzulla T, Raber J (2014). Impairment in extinction of contextual and cued fear following post-training whole-body irradiation. Front. Behav. Neurosci..

[52] Villasana L, Pfankuch T, Raber J (2010). Isoform-dependent effects of apoE on doublecortin-positive cells and microtubule-associated protein 2 immunoreactivity following 137Cs irradiation. Radiat. Environ. Biophys..

[53] Villasana L, Rosenberg J, Raber J (2010). Sex-dependent effects of 56Fe Irradiation on contextual fear conditioning in C56BL/6J mice. Hippocampus.

[54] Tobin B, Uchakin P, Leeper-Woodford S (2002). Insulin secretion and sensitivity in space flight: diabetogenic effects. Nutrition.

[55] da Silveira W (2020). Comprehensive multi-omics analysis reveals mitochondrial stress as a central biological hub for spaceflight impact. Cell.

