# Supplementary material for: Associations between lipids in selected brain regions, plasma miRNA, and behavioral and cognitive measures following 28Si ion irradiation
Source: Sci Rep. 2021 Jul 21;11:14899. doi: 10.1038/s41598-021-93869-3 (PMC8295277; doi:10.1038/s41598-021-93869-3)
Supplement: Supplementary file 1 — Supplementary Information 1. [file 41598_2021_93869_MOESM1_ESM.pdf]

Suppl Fig 1 Cognitive vs miRNA – TP2

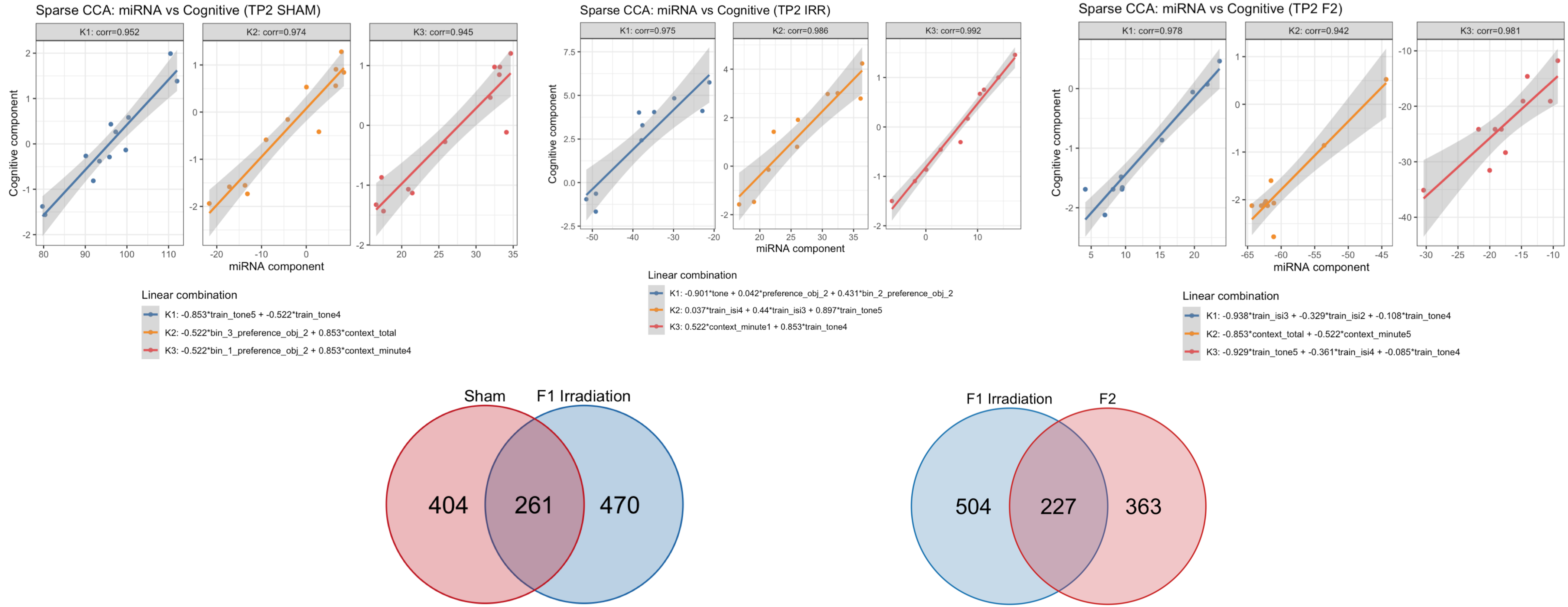

Suppl Fig 2 - Cognitive vs miRNA – TP3

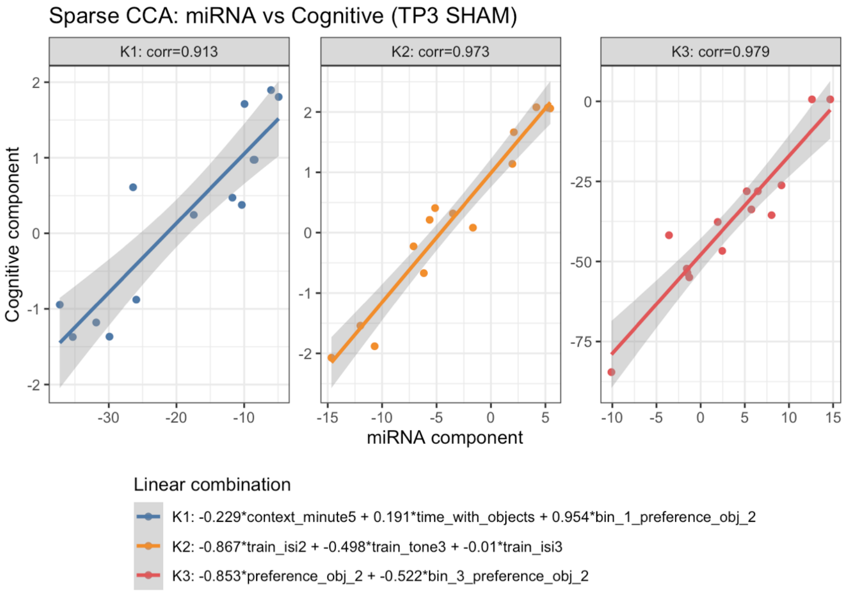

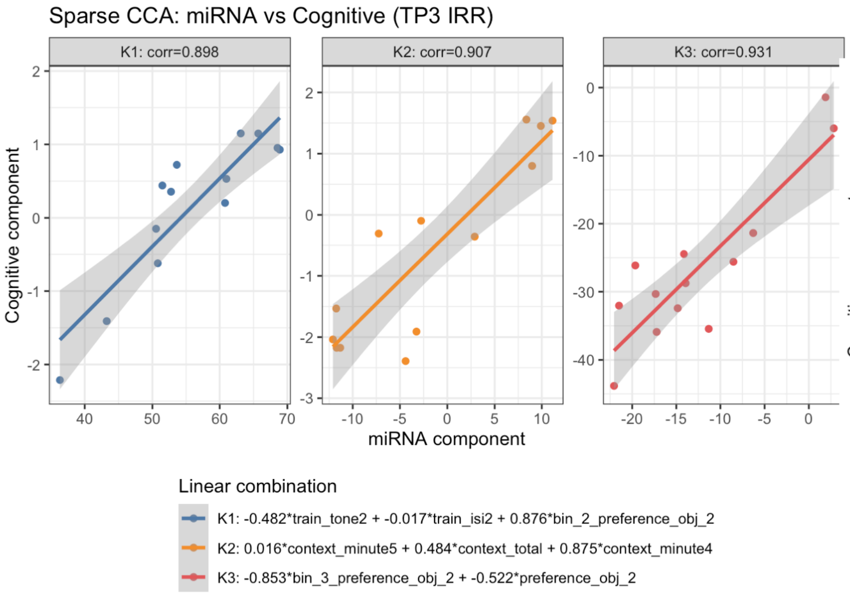

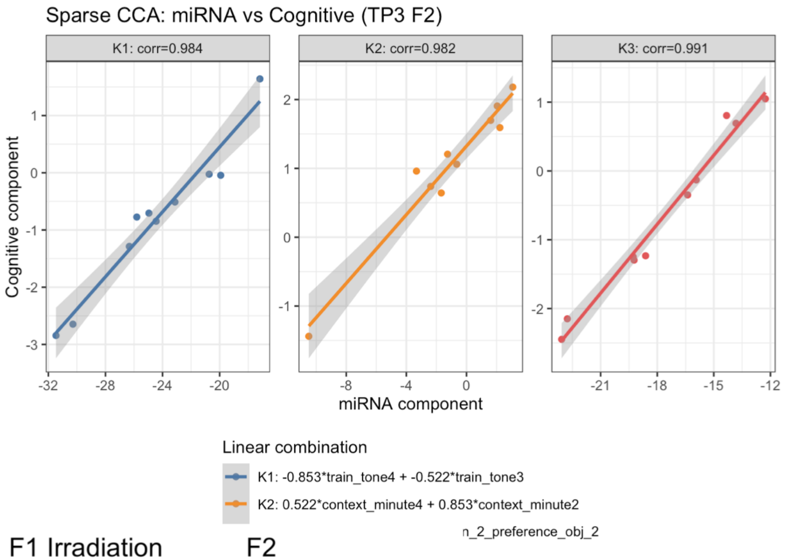

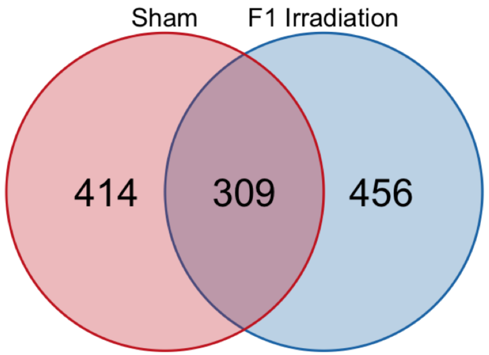

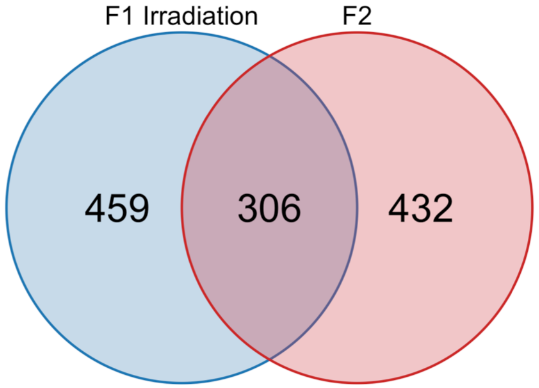

Suppl Fig 3 - Cognitive vs miRNA – TP1

F1 Sham

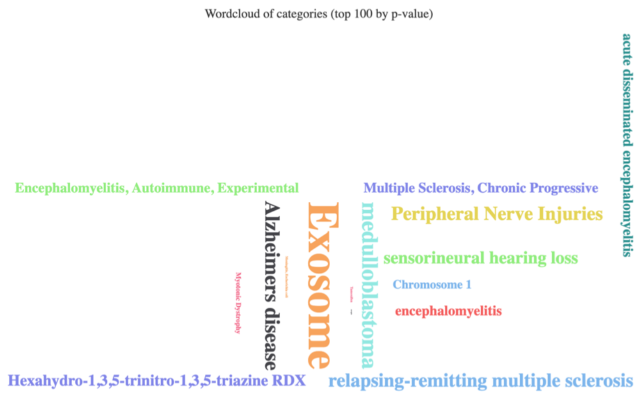

F1 IRR

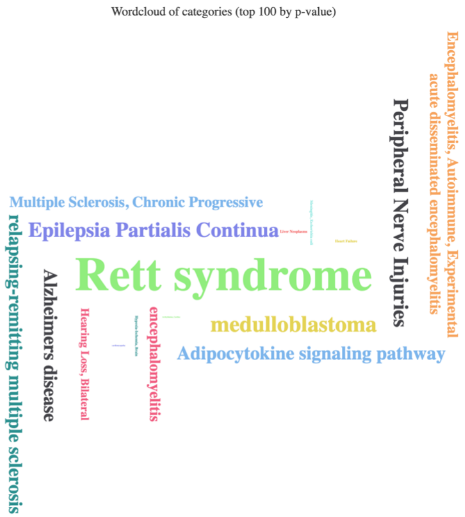

F2 IRR

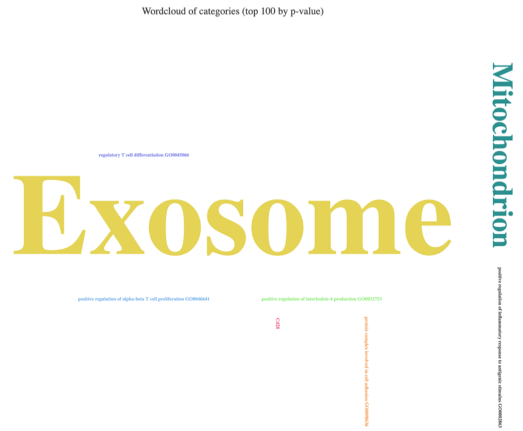

Suppl Fig 4 Cognitive vs miRNA – TP2

F1 Sham

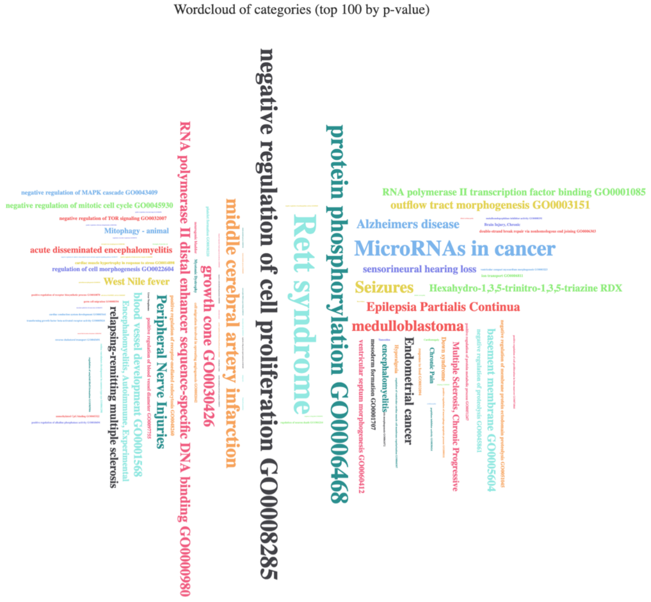

F1 IRR

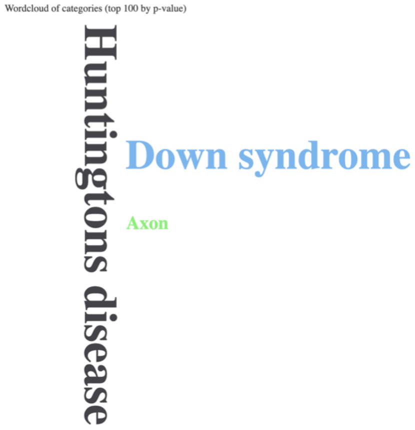

F2 IRR

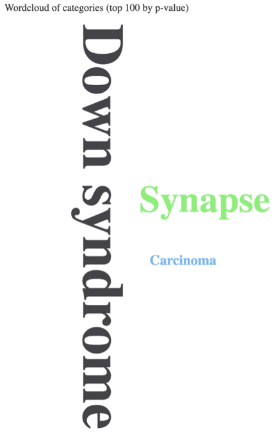

Suppl Fig 5 - Cognitive vs miRNA – TP3

F1 Sham

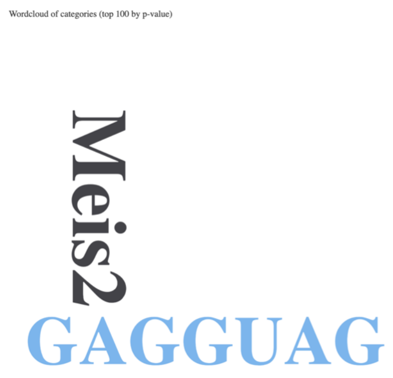

F1 IRR

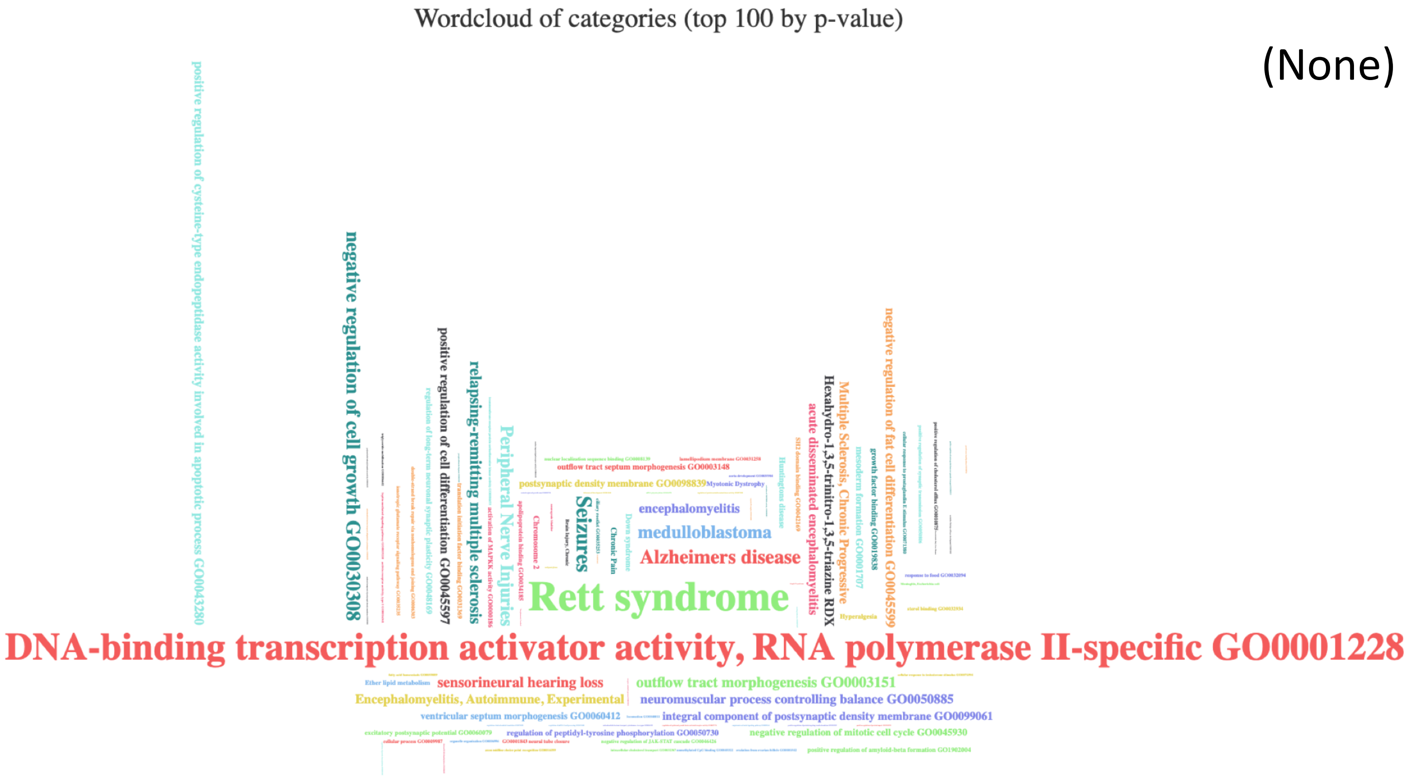

F2 IRR

(None)

Suppl Fig 6 – Behavioral vs miRNA – TP2

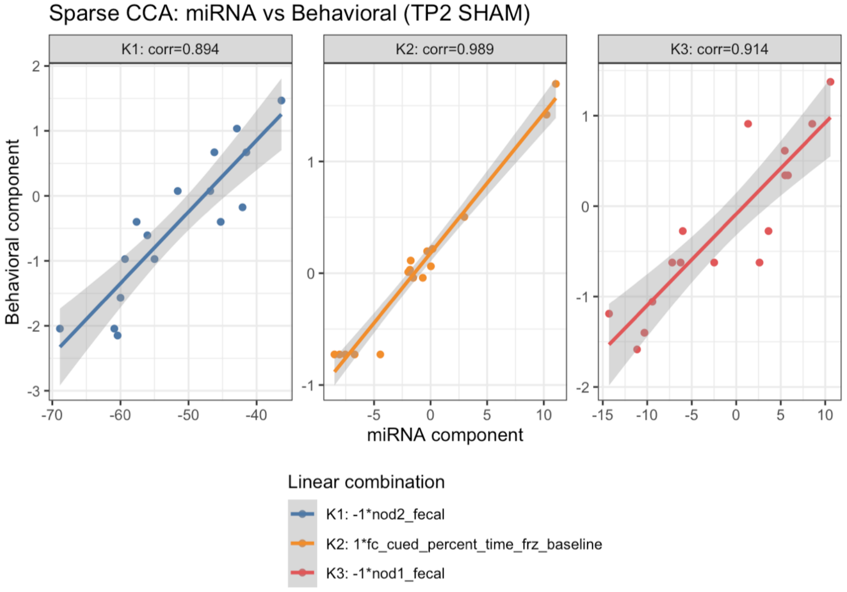

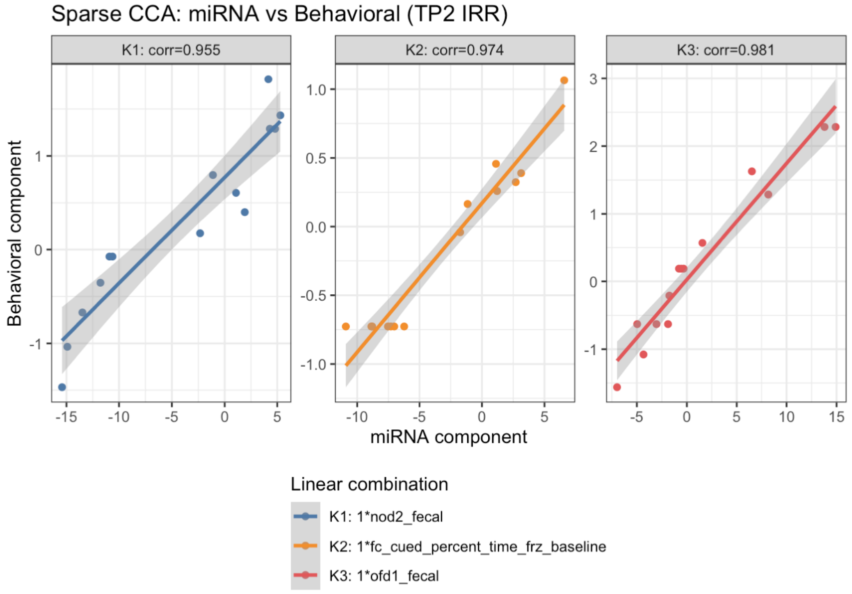

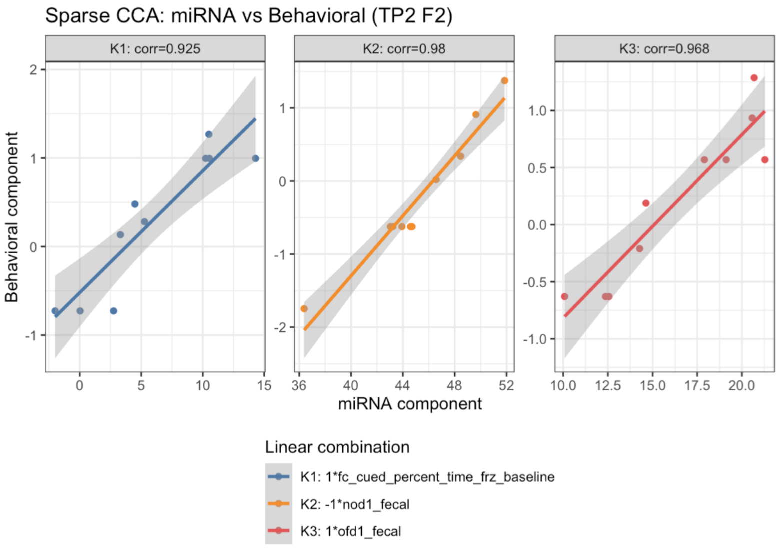

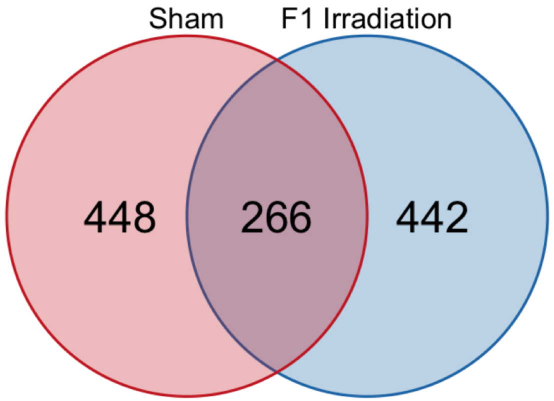

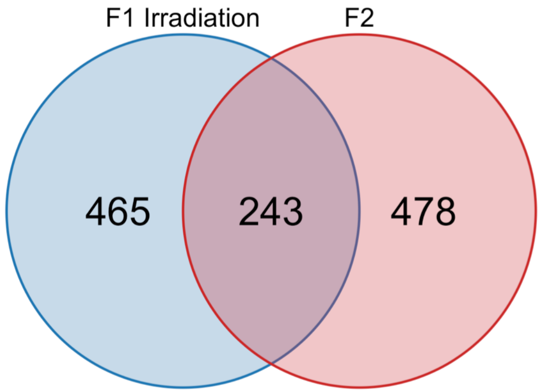

Suppl Fig 7 - Behavioral vs miRNA – TP3

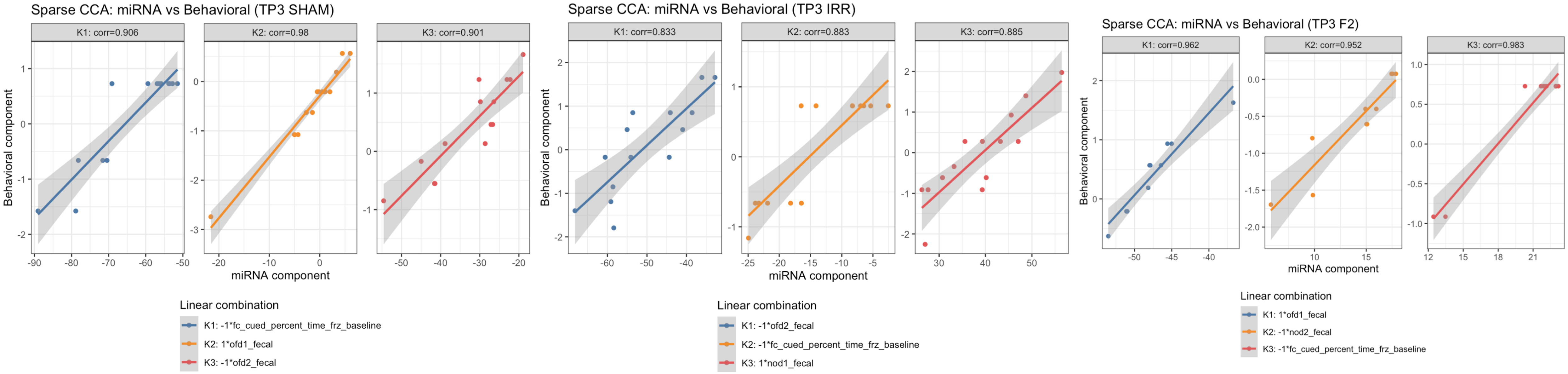

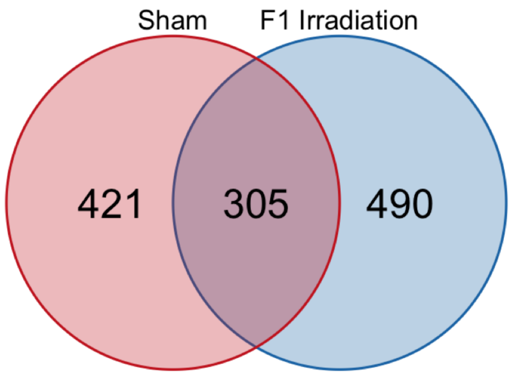

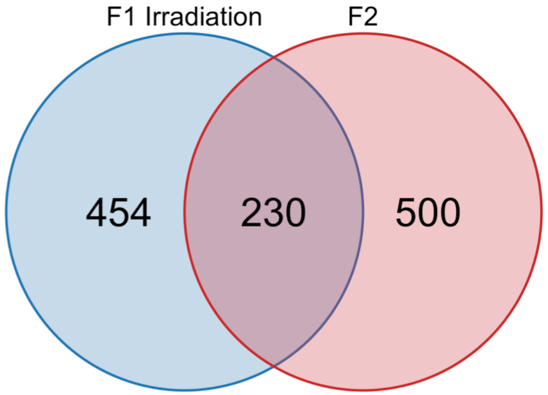

Suppl Fig 8 Behavioral vs miRNA – TP1

F1 Sham

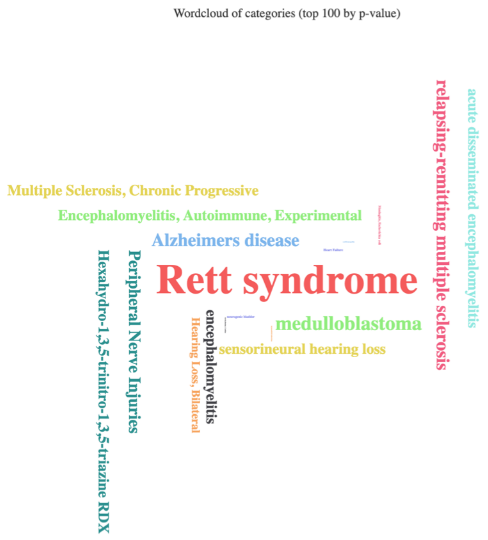

F1 IRR

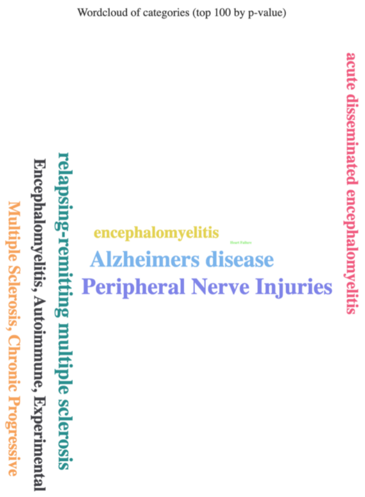

F2 IRR

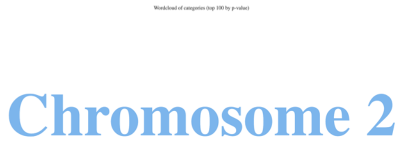

Suppl Fig 9 – Behavioral vs miRNA – TP2

F1 Sham

(None)

F1 IRR

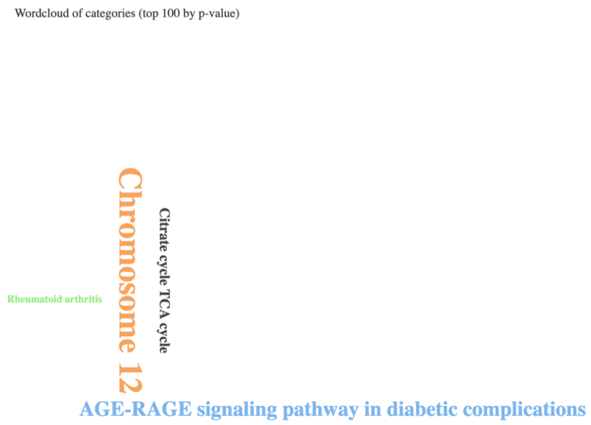

F2 IRR

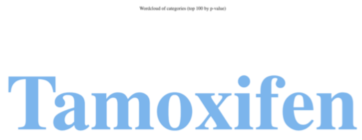

Suppl Fig 10 - Behavioral vs miRNA – TP3

F1 Sham

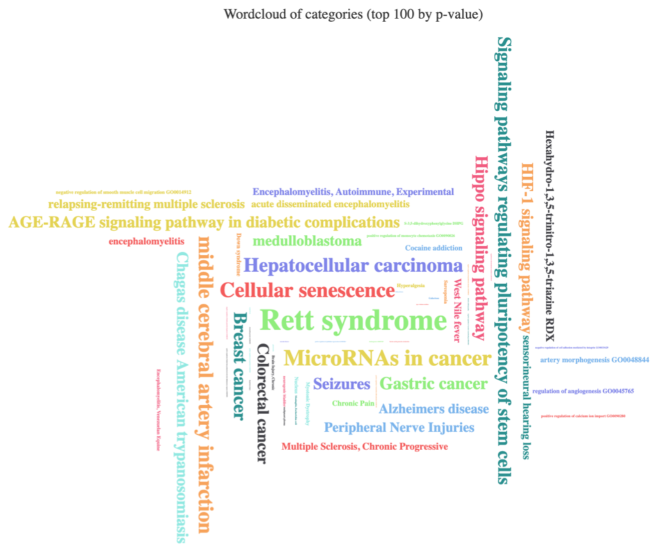

F1 IRR

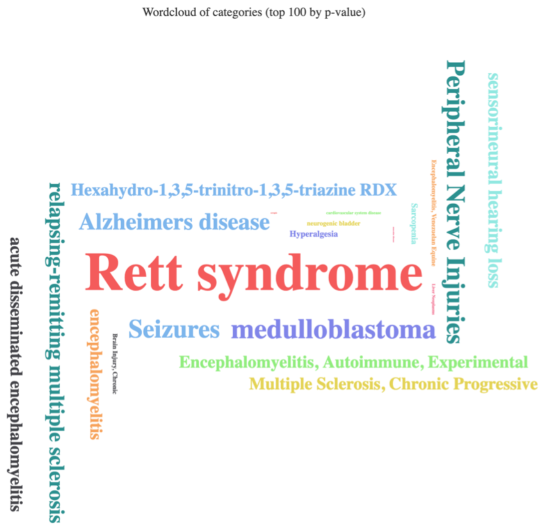

F2 IRR

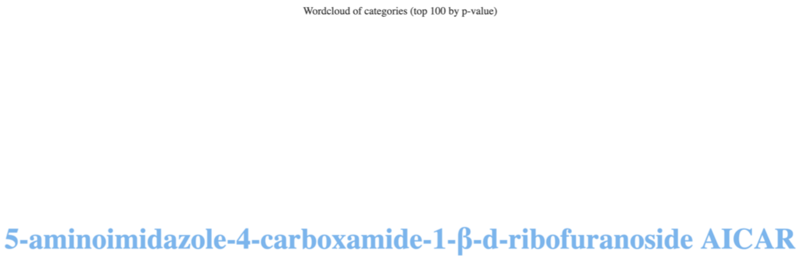

Suppl Fig 11 Lipidomics vs miRNA – TP1

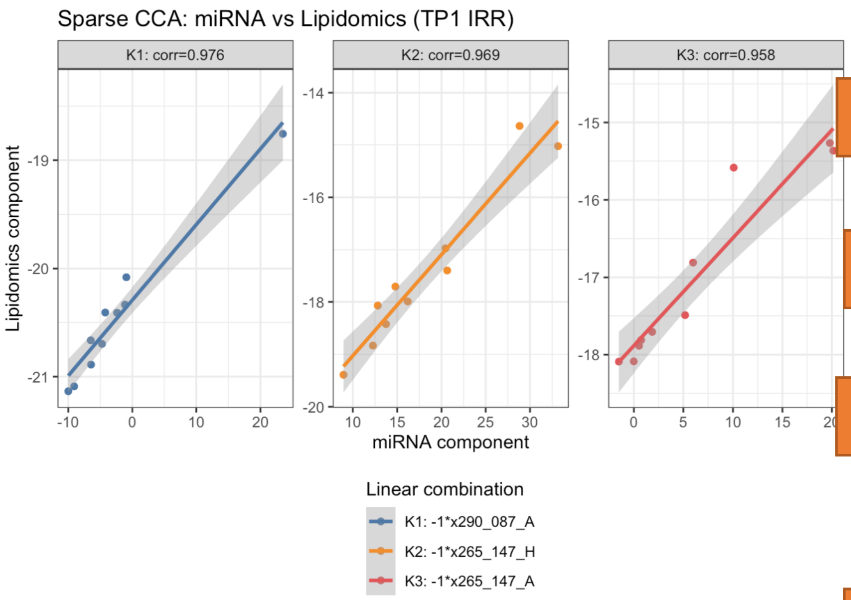

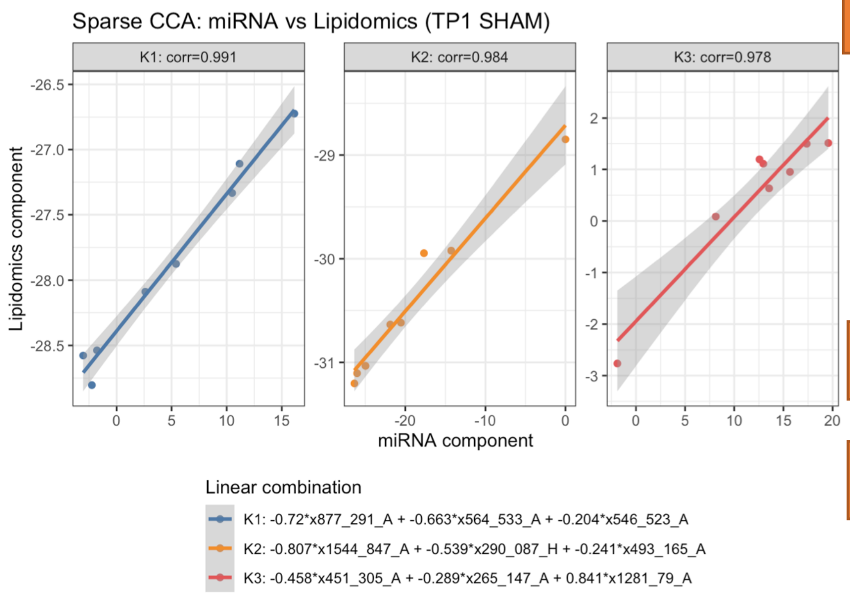

LIPIDS

TP1 IRR

K1 — 290.087 A = fatty acid which can be of multiple carbon length and/or number of double bonds

K2 — 265.147 H = Micropine (sphingoid base analogue)

K3 — 265.147 A = Micropine (sphingoid base analogue)

TP1 SHAM

K1 — 877.291 A = no identification,
564.533 A = three biological possibilities: a large 34-35 carbon oxidized unsaturated fatty acid, an oxidized diacylglycerol or a branched fatty acid esters of hydroxy fatty acids (FAHFA 18:1 18:0, Delta of 0.0212) are endogenous lipids found in adipose tissue and serum that correlate with insulin sensitivity and are reduced in insulin-resistant humans
546.523 A = Most-likely a large 34-35 carbon oxidized unsaturated fatty acid or an oxidized diacylglycerol

K2 — 1544.847 A = ganglioside SB1a, 290.087 H = fatty acid which can be of multiple carbon length and/or number of double bonds, 493.164 A = no ID

K3 — 451.305 A = matched mass 451.2699, Delta 0.0351, lysophosphatidylethanolamine (16:0) or Lysophosphatidylcholine (O-14:1) delta 0.0013. LPC is a signal of mitochondrial stress. Both compounds are derived from their precursors by the enzymatic action of phospholipase A2 , 265.147A = Micropine (sphingoid base analogue) , 1281.790 A = No ID

miRNA enrichment

TP1 IRR

K1

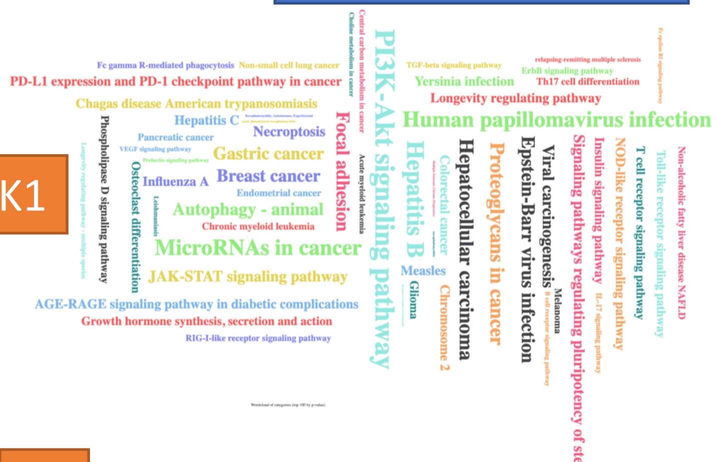

K2

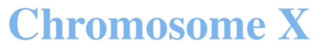

K3 (None)

TP1 SHAM

K1 (None)

K2

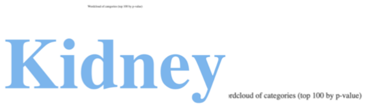

K3

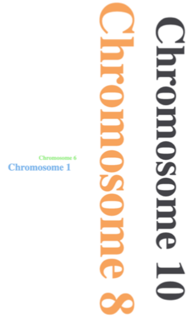

Suppl Fig 12 Lipidomics vs miRNA – TP2

miRNA enrichment

LIPIDS

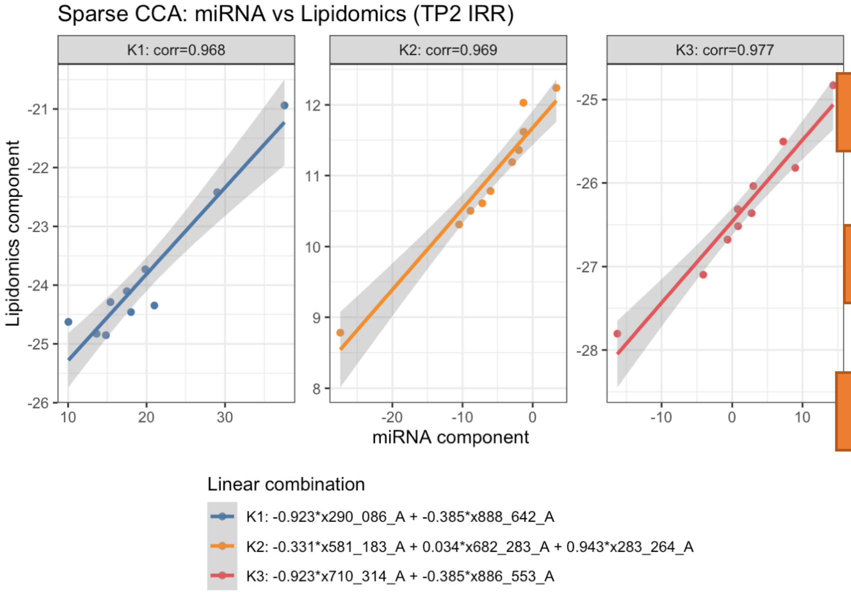

K1

290.086 A = fatty acid which can be of multiple carbon length and/or number of double bonds , 888.642 A = Isobars for unsaturated Phosphatidylinositol with 38 carbons in its tail or an unsaturated triacylglycerol (TG) with 55 carbons composing its three fatty acid tails.

K2

581.183 A = no ID , 682.283 A = no ID, 283.264 A = n-acylethanolamine (15:1), (induction of inflammation, precursor of eicosanoids or Delta 0.0235, sphingolipid (m18:1), sphingolipid base analog

K3

710.314 A = isobars corresponding to either an oxidized Phosphatidylglycerol or oxidized Phosphatidylinositol, 886.553 A = Most hits are for an unsaturated Phosphatidylinositol with 38 carbons in its tail

K1

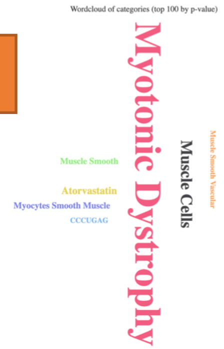

K2

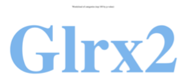

K3

(None)

K1

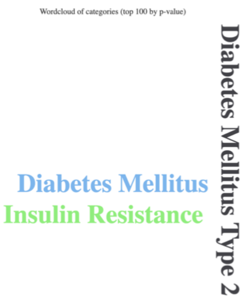

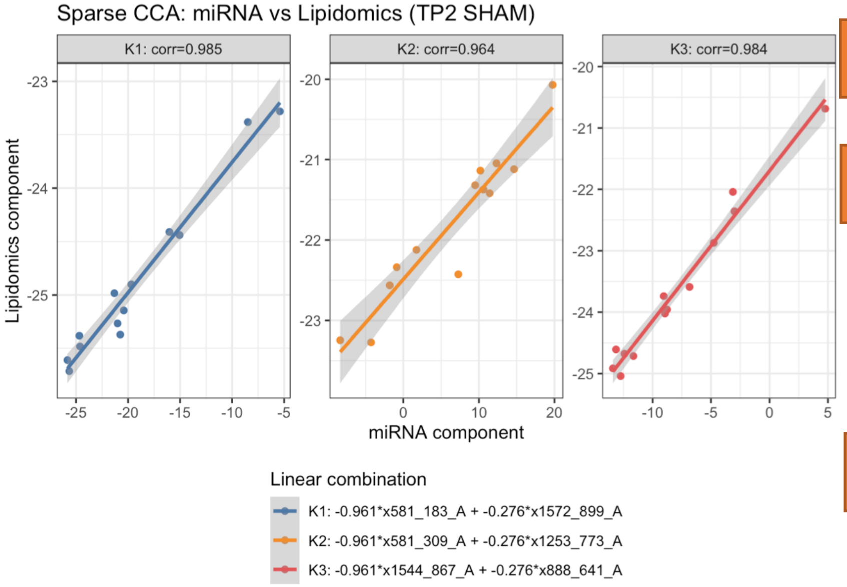

K1

581.183 A = no ID, 1572.899 A = several possible isomers of a sulfated globoside

K2

581.309 A = either lysophosphatidylethanolamine (26:6) delta 0.0391 or Lysophosphatidylserine (22:0) delta 0.0603. Both compounds are derived from their precursors by the enzymatic action of phospholipase A2. The lysophospholipids lead to increase eicosanoid production (leukotrienes and prostaglandins) , 1253.773 A = cytidine diphosphate lipid (CDP-1)

K2

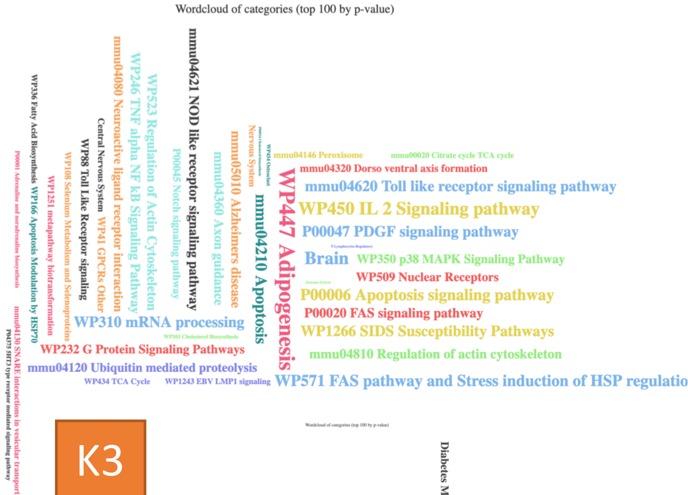

K3

1544.867 A = possibly Ganglioside SB1a (t18:0/26:0), matched mass1544.83069, another sulfated globoside.
888.641 A = Isobars for unsaturated Phosphatidylinositol with 38 carbons in its tail or an unsaturated triacylglycerol (TG) with 55 carbons composing its three fatty acid tails.

K3

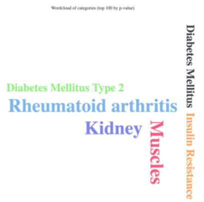

Suppl Fig 13 Lipidomics vs miRNA – TP3

LIPIDS

miRNA enrichment

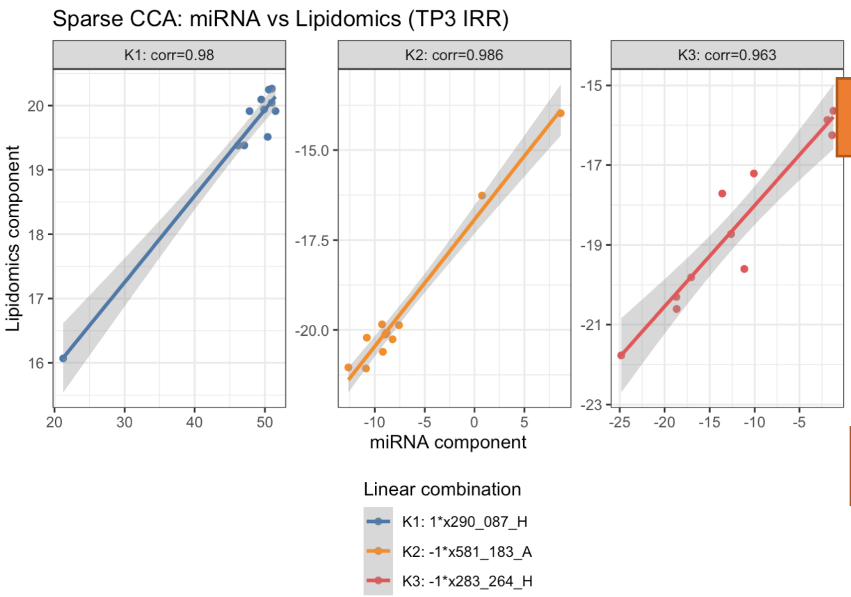

K1

290.087 H = a fatty acid which can be of multiple carbon length and/or number of double bonds

K2

581.183 A = no ID

K3

283.264 H = n-acylethanolamine (15:1), (induction of inflammation, precursor of eicosanoids or Delta 0.0235, sphingolipid (m18:1), sphingolipid base analog

K1

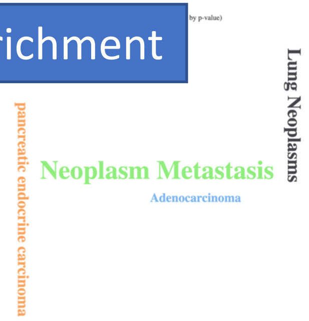

K2

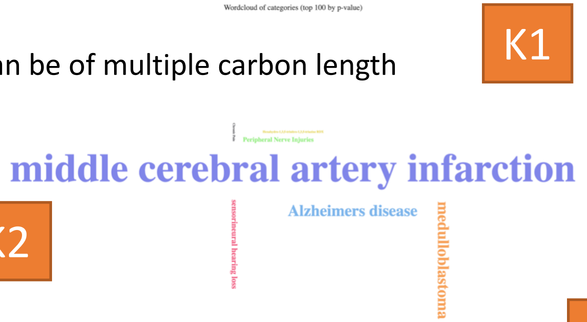

K3

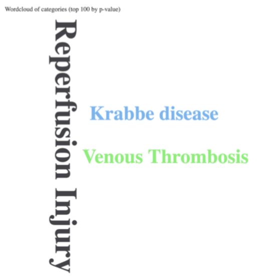

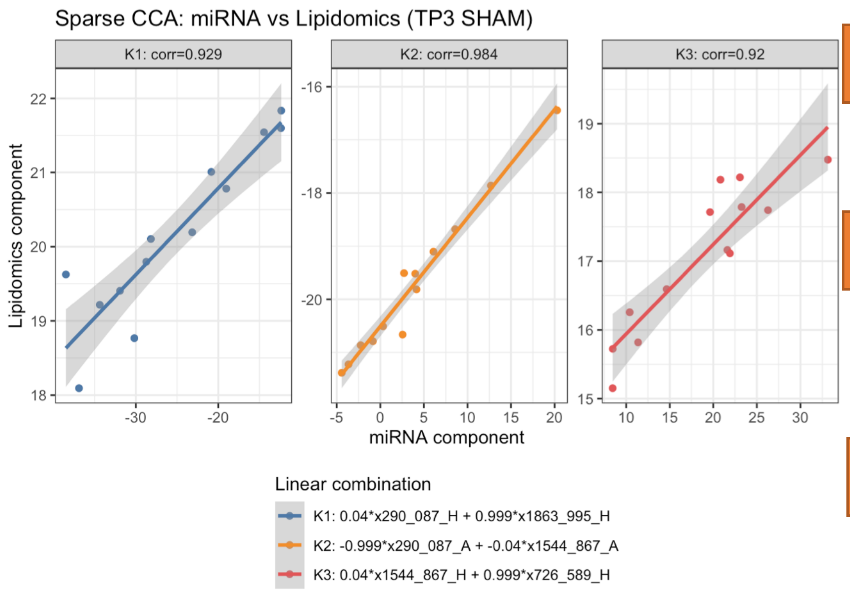

K1

290.087 H = fatty acid which can be of multiple carbon length and/or number of double bonds.
1863.995 H = Ganglioside Fuc-GM1 (NeuGC)

K2

290.087 H = = fatty acid which can be of multiple carbon length and/or number of double bonds
1544.867 A = possibly Ganglioside SB1a (t18:0/26:0), matched mass1544.83069, another sulfated globoside.

K3

1544.867 A = possibly Ganglioside SB1a (t18:0/26:0), matched mass1544.83069, another sulfated globoside.
726.589 H = isobars corresponding to either sphingomyelin (d18:2/18:1, delta 0.0214) which makes up the myelin sheath around neurons, diacylglycerol or phosphatidic acid

K1

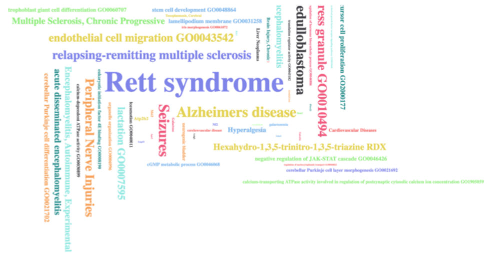

K2

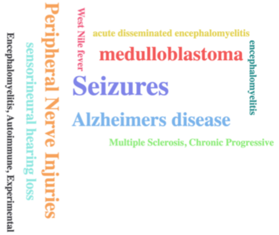

K3

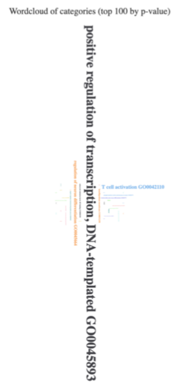

Suppl Fig 14 Cognitive vs lipidomics – TP1

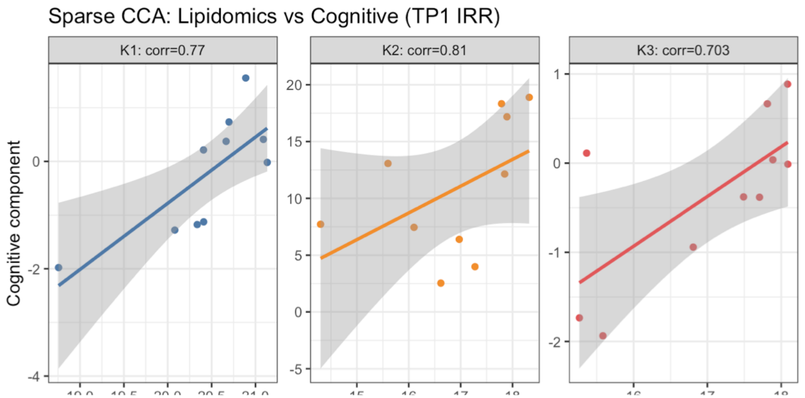

K	form_out	form_lipid	corr
V1	K1: -0.959*bin_2_preference_obj_2* + -0.243tone + -0.139*context_minute4* + -0.033context_minute5	1 x290_087_A	0.7697754
V2	K2: -0.972*train_tone1* + 0.054train_isi3 + 0.068*train_tone5* + 0.077train_tone4 + 0.204*train_isi4	1 x290_086_H	0.8096856
V3	K3: -0.974*bin_1_preference_obj_2* + -0.076train_tone2 + -0.009*train_tone5* + 0.021context_minute1 + 0.122*context_minute4* + 0.173train_tone1	1 x265_147_A	0.7025606

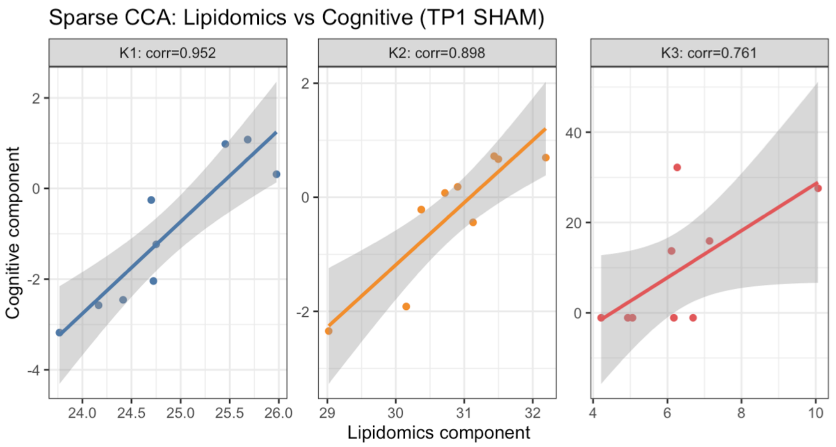

K	form_out	form_lipid	corr
V1	K1: -0.959*bin_2_preference_obj_2* + -0.243tone + -0.139*context_minute4* + -0.033context_minute5	1 x290_087_A	0.7697754
V2	K2: -0.972*train_tone1* + 0.054train_isi3 + 0.068*train_tone5* + 0.077train_tone4 + 0.204*train_isi4	1 x290_086_H	0.8096856
V3	K3: -0.974*bin_1_preference_obj_2* + -0.076train_tone2 + -0.009*train_tone5* + 0.021context_minute1 + 0.122*context_minute4* + 0.173train_tone1	1 x265_147_A	0.7025606

LIPIDS

K1 290.087 A = a fatty acid which can be of multiple carbon length and/or number of double bonds

K2 290.086 H = a fatty acid which can be of multiple carbon length and/or number of double bonds

K3 265.147 A = Micropine (sphingoid base analogue)

K1

451.305 A = lysophosphatidylethanolamine (16:0) or Lysophosphatidylcholine (O-14:1) delta 0.0013. LPC is a signal of mitochondrial stress. Both compounds are derived from their precursors by the enzymatic action of phospholipase A2

1281.79 A = no ID

1544.847 A = possibly Ganglioside SB1a (t18:0/26:0)

290.087 H = a fatty acid which can be of multiple carbon length and/or number of double bonds

K2

1544.847 A = possibly Ganglioside SB1a (t18:0/26:0)

419.255 A = N-Arachidonoyl Taurine (NAT (18:0)), an arachidonyl aminoacid, N-Arachidonoyl Taurine is increased after the administration of cannabinoid agonists

886.546 A = Most hits are for an unsaturated Phosphatidylinositol with 38 carbons in its tail

K3

493.165 A = no ID, 581.180 A = no ID

708.574 A = Several isobaric possibilities. The largest number of hits corresponds to a Phosphatidylglycerol (PG) of various carbon chain lengths totaling 31 carbons, next would be a diacylglycerol (20:0/22:0 or 21:0/21:0)

1544.849 A = possibly Ganglioside SB1a (t18:0/26:0)

Suppl Fig 15 Cognitive vs lipidomics – TP2

Sparse CCA: Lipidomics vs Cognitive (TP2 IRR)

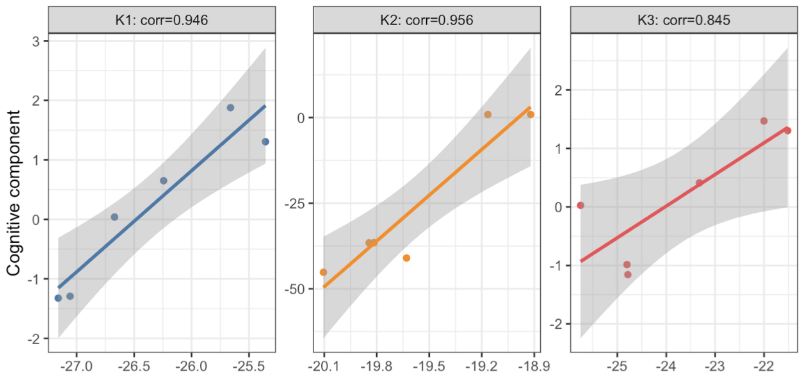

K	form_out	form_lipid	corr
V1	K1: 0.164context_minute5 + 0.259context_minute3 + 0.952*context_total	-0.385 x291_091_A + -0.923 x581_184_A	0.9456560
V2	K2: -0.853bin_2_preference_obj_2 + -0.522preference_obj_2	-0.971 x290_088_A + -0.153 x581_183_A + 0.184 x886_553_A	0.9557277
V3	K3: -0.227bin_3_preference_obj_2 + 0.193train_tone3 + 0.955*train_isi2	-0.385 x1544_867_A + -0.923 x835_283_A	0.8446590

Sparse CCA: Lipidomics vs Cognitive (TP2 SHAM)

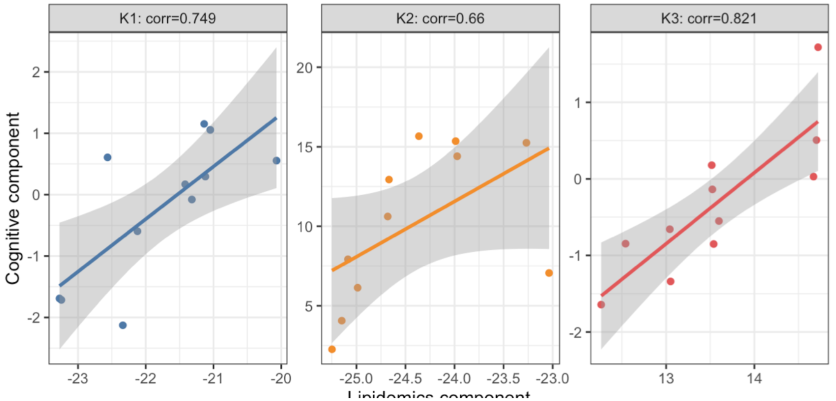

K	form_out	form_lipid	corr
V1	K1: 0.067context_minute2 + 0.39context_minute3 + 0.919*context_total	-0.276 x1253_773_A + -0.961 x581_309_A	0.7493977
V2	K2: 0.191train_isi4 + 0.23context_minute1 + 0.954*context_minute2	-0.276 x291_091_A + -0.961 x581_183_A	0.6596294
V3	K3: 0.094context_minute4 + 0.146tone + 0.165context_minute5 + 0.971train_tone3	0.961 x290_087_A + -0.276 x419_256_A	0.8207900

LIPIDS

K1

291.091 A = no ID
581.184 A = no ID

K2

290.088 H = a fatty acid which can be of multiple carbon length and/or number of double bonds
581.183 A = no ID

K3

1544.867 A = possibly Ganglioside SB1a (t18:0/26:0)
835.283 A = CDP-1-hexanoyl-2-(6Z,9Z,12Zoctadecatrienoyl)-sn-glycerol CP1 (cytodine diphosphate lipid), I cannot find any biological activity

K1

1253.773 A = cytidine diphosphate lipid (CDP-1)
581.309 A = either lysophosphatidylethanolamine (26:6) delta 0.0391 or Lysophosphatidylserine (22:0) delta 0.0603. Both compounds are derived from their precursors by the enzymatic action of phospholipase A2. The lysophospholipids lead to increase eicosanoid production (leukotrienes and prostaglandins)

K2

291.091 A = no ID
581.183 A = no ID

K3

290.087 A = a fatty acid which can be of multiple carbon length and/or number of double bonds
419.256 A = either an acyl carnitine (18:4) matched mass 419.3036, delta 0.0486 or an acyl taurine (20:0) matched mass of 419.3069, delta 0.0519

Suppl Fig 16 Cognitive vs lipidomics – TP3

Sparse CCA: Lipidomics vs Cognitive (TP3 IRR)

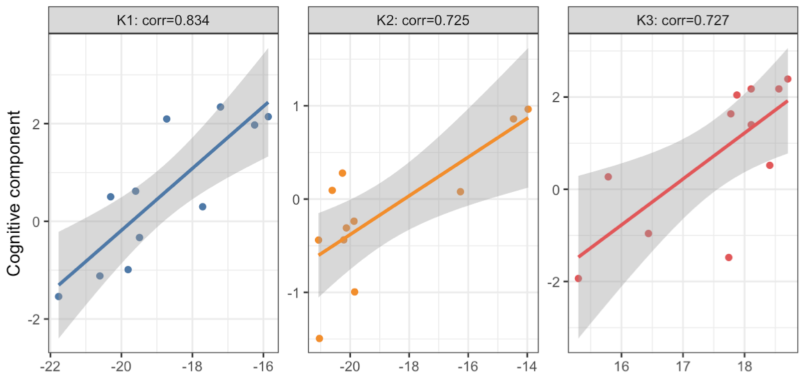

K	form_out	form_lipid	corr
V1	K1: -0.896context_minute5 + -0.442context_total + -0.036*context_minute3	-1 x283_264_H	0.8338296
V2	K2: -0.081train_tone3 + -0.021train_isi1 + 0.035bin_1_preference_obj_2 + 0.283bin_2_preference_obj_2 + 0.955*time_with_objects	-1 x581_183_A	0.7248504
V3	K3: -0.879context_minute1 + -0.476context_total + -0.019*context_minute4	1 x888_641_H	0.7268940

Sparse CCA: Lipidomics vs Cognitive (TP3 SHAM)

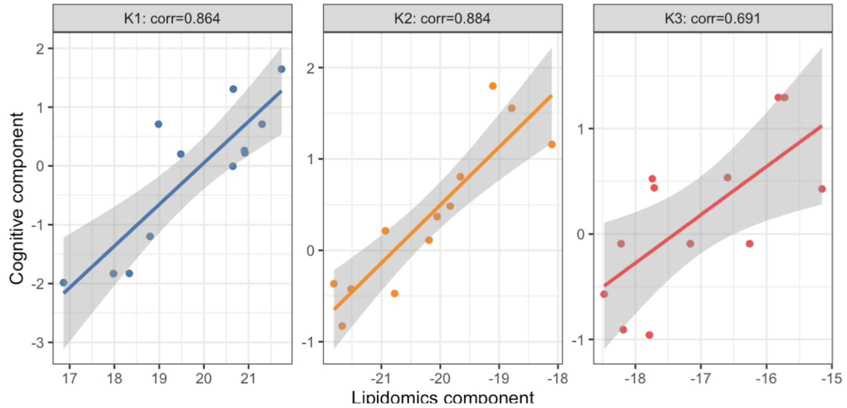

K	form_out	form_lipid	corr
V1	K1: 0.156context_minute2 + 0.268train_isi3 + 0.951*context_minute1	0.04 x290_087_A + 0.999 x581_183_A	0.8644827
V2	K2: -0.853train_isi1 + -0.522train_isi2	-0.04 x1604_89_H + -0.999 x1863_995_H	0.8844297
V3	K3: -0.853train_tone1 + 0.522bin_2_preference_obj_2	-0.04 x1544_867_H + -0.999 x726_589_H	0.6908343

LIPIDS

K1 283.264 H = Delta0.0129, n-acylethanolamine (15:1), (induction of inflammation, precursor of eicosanoids or Delta 0.0235, sphingolipid (m18:1), sphingolipid base analog

K2 581.183 A = no ID

K3 888.641 H = Isobars for unsaturated Phosphatidylinositol with 38 carbons in its tail or an unsaturated triacylglycerol (TG) with 55 carbons composing its three fatty acid tails.

K1 290.087 A = a fatty acid which can be of multiple carbon length and/or number of double bonds
581.183 A = no ID

K2 1604.89 H = several possible isomers of a sulfated globoside
1863.995 H = Ganglioside Fuc-GM1 (NeuGC)

K3 1544.867 H = possibly Ganglioside SB1a (t18:0/26:0)
726.589 H = isobars corresponding to either sphingomyelin (d18:2/18:1, delta 0.0214) which makes up the myelin sheath around neurons, diacylglycerol or phosphatidic acid

Suppl Fig 17 Behavioral vs lipidomics – TP1

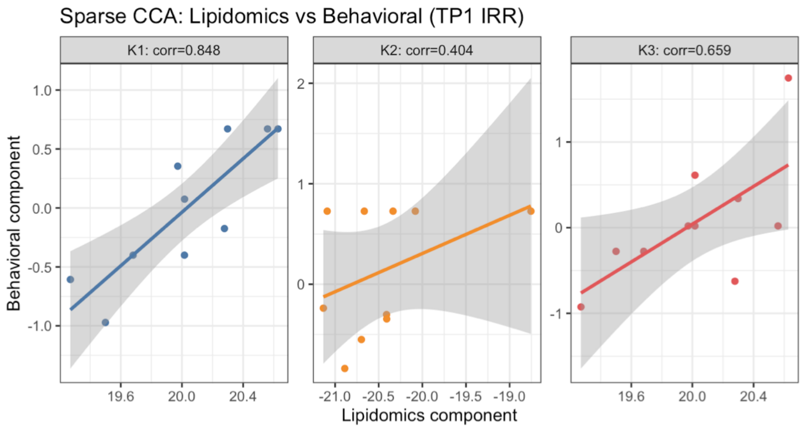

K	form_out	form_lipid	corr
V1	K1: -1*nod2_fecal	1 x290_087_H	0.8482406
V2	K2: -1*fc_cued_percent_time_frz_baseline	-1 x290_087_A	0.4035642
V3	K3: -1*nod1_fecal	1 x290_087_H	0.6593617

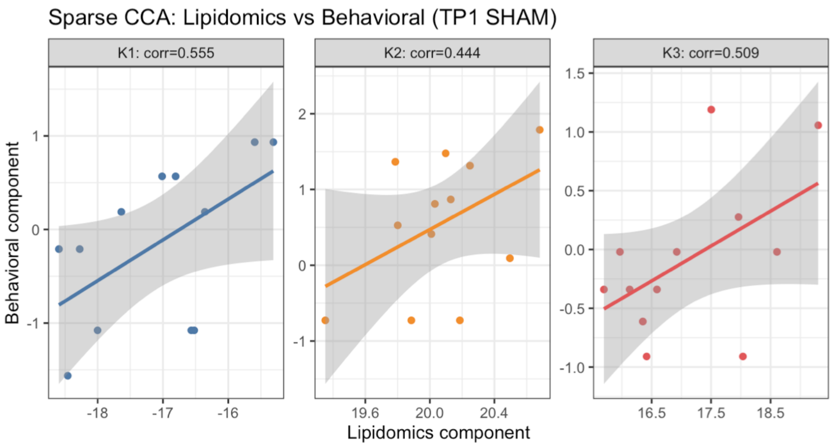

K	form_out	form_lipid	corr
V1	K1: 1*ofd1_fecal	-1 x290_086_H	0.5554043
V2	K2: 1*fc_cued_percent_time_frz_baseline	1 x290_087_H	0.4440140
V3	K3: 1*nod1_fecal	1 x265_147_A	0.5085519

LIPIDS

K1 290.087 H = a fatty acid which can be of multiple carbon length and/or number of double bonds

K2 290.087 A = a fatty acid which can be of multiple carbon length and/or number of double bonds

K3 290.087 H = a fatty acid which can be of multiple carbon length and/or number of double bonds

K1 290.086 H= a fatty acid which can be of multiple carbon length and/or number of double bonds

K2 290.087 H = a fatty acid which can be of multiple carbon length and/or number of double bonds

K3 265.147 A = Delta0.0572, Micropine (sphingoid base analogue)

Suppl Fig 18 Behavioral vs lipidomics – TP2

Sparse CCA: Lipidomics vs Behavioral (TP2 IRR)

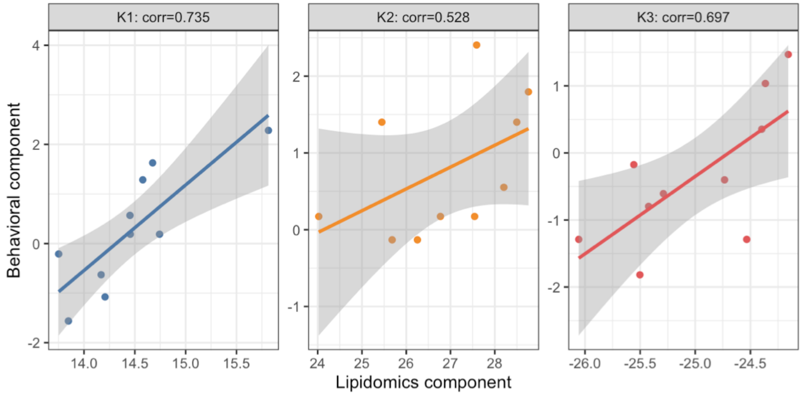

K	form_out	form_lipid	corr
V1	K1: 1*ofd1_fecal	-0.152 x1544_867_A + 0.971 x1863_996_A + -0.185 x888_642_A	0.7354057
V2	K2: 1*ofd2_fecal	0.385 x1544_87_A + 0.923 x2127_061_A	0.5280724
V3	K3: -1*nod2_fecal	0.0159 x581_184_A + -0.933 x682_283_A + -0.358 x710_314_A	0.6965808

Sparse CCA: Lipidomics vs Behavioral (TP2 SHAM)

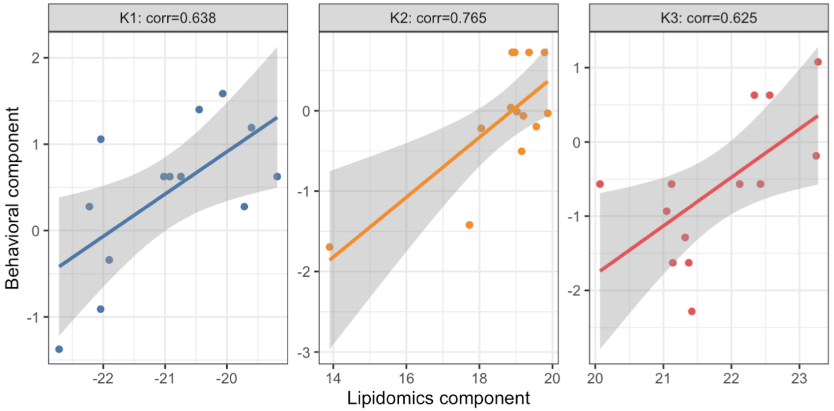

K	form_out	form_lipid	corr
V1	K1: 1*nod1_fecal	-0.978 x1253_773_A + -0.0592 x1544_867_A + -0.2 x581_309_A	0.6383476
V2	K2: -1*fc_cued_percent_time_frz_baseline	-0.157 x888_641_A + 0.0975 x1544_867_H + 0.983 x581_183_H	0.7651704
V3	K3: -1*ofd1_fecal	0.276 x1253_773_A + 0.961 x581_309_A	0.6251693

LIPIDS

K1

1544.867 A = possibly Ganglioside SB1a (t18:0/26:0)
1863.996 A = Ganglioside Fuc-GM1 (NeuGC)

K2

1544.870 A = possibly Ganglioside SB1a (t18:0/26:0)
2127.061 A = Matched mass, 2127.16016 Ganglioside GD1a(NeuGc/NeuGc)(t18:0/36:6(18Z,21Z,24Z,27Z,30Z,33Z), brain ganglioside and it shows the correct mouse sialyation of NeuGc.

K3

581.184 A = no ID
682.283 A = phosphatidylglycerophosphate (PGP). PGP is a precursor of cardiolipins in the mitochondria.
710.314 A = isobars corresponding to either an oxidized Phosphatidylglycerol or oxidized Phosphatidylinositol

K1

1253.773 A = cytidine diphosphate lipid (CDP-1)
1544.867 A = possibly Ganglioside SB1a (t18:0/26:0)
581.309 A = either lysophosphatidylethanolamine (26:6) delta 0.0391 or Lysophosphatidylserine (22:0) delta 0.0603. Both compounds are derived from their precursors by the enzymatic action of phospholipase A2. The lysophospholipids lead to increase eicosanoid production (leukotrienes and prostaglandins)

K2

888.641 A = Isobars for unsaturated Phosphatidylinositol with 38 carbons in its tail or an unsaturated triacylglycerol (TG) with 55 carbons composing its three fatty acid tails.
1544.867 H = possibly Ganglioside SB1a (t18:0/26:0)
581.183 H = no ID

K3

1253.773 A = cytidine diphosphate lipid (CDP-1)
581.309 A = either lysophosphatidylethanolamine (26:6) delta 0.0391 or Lysophosphatidylserine (22:0) delta 0.0603. Both compounds are derived from their precursors by the enzymatic action of phospholipase A2. The lysophospholipids lead to increase eicosanoid production (leukotrienes and prostaglandins)

Suppl Fig 19 Behavioral vs lipidomics – TP3

Sparse CCA: Lipidomics vs Behavioral (TP3 IRR)

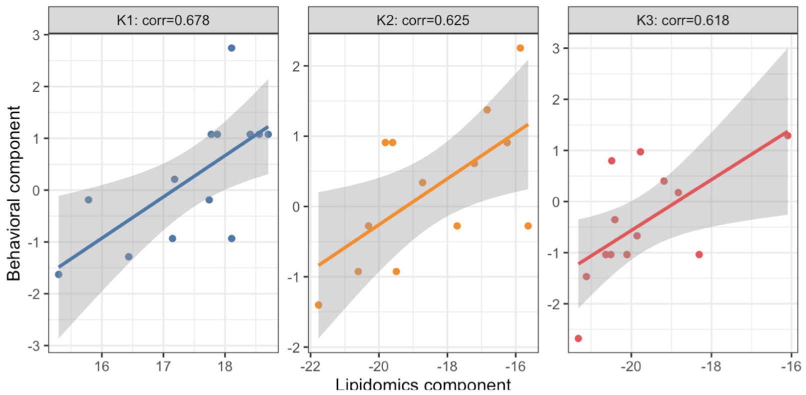

K	form_out	form_lipid	corr
V1	K1: -1*ofd1_fecal	1 x888_641_H	0.6780741
V2	K2: -1*nod1_fecal	-1 x283_264_H	0.6246149
V3	K3: 1*nod2_fecal	-1 x581_183_H	0.6177454

Sparse CCA: Lipidomics vs Behavioral (TP3 SHAM)

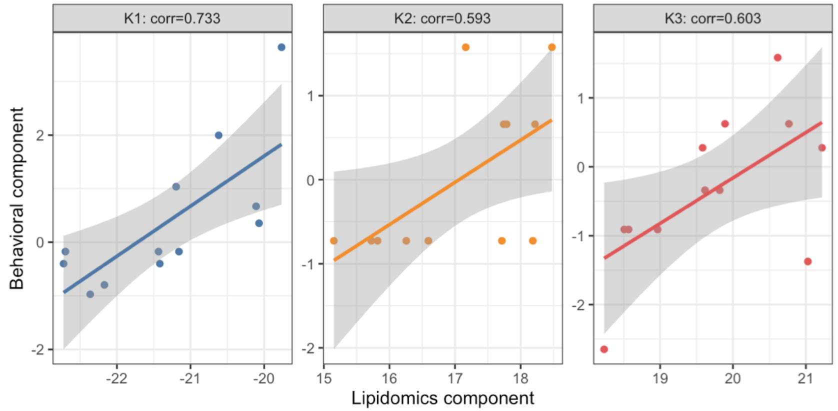

K	form_out	form_lipid	corr
V1	K1: -1*nod2_fecal	-0.04 x1544_867_A + -0.999 x1835_964_A	0.7329386
V2	K2: 1*fc_cued_percent_time_frz_baseline	0.04 x1544_867_H + 0.999 x726_589_H	0.5932461
V3	K3: 1*nod1_fecal	0.999 x1835_964_A + -0.04 x1863_995_H	0.6027099

LIPIDS

K1 888.641 H = Isobars for unsaturated Phosphatidylinositol with 38 carbons in its tail or an unsaturated triacylglycerol (TG) with 55 carbons composing its three fatty acid tails.

K2 283.264 H = Delta0.0129, n-acylethanolamine (15:1), (induction of inflammation, precursor of eicosanoids or Delta 0.0235, sphingolipid (m18:1), sphingolipid base analog

K3 581.183 H = no ID

K1 1544.867 A = possibly Ganglioside SB1a (t18:0/26:0)
1835.964 A =Ganglioside Fuc-GM1 (NeuGC)

K2 1544.867 H = possibly Ganglioside SB1a (t18:0/26:0)
726.589 H = isobars corresponding to either sphingomyelin (d18:2/18:1, delta 0.0214) which makes up the myelin sheath around neurons, diacylglycerol or phosphatidic acid

K3 1835.964 A = Ganglioside Fuc-GM1 (NeuGC)
1863.995 H =Ganglioside Fuc-GM1 (NeuGC)
