# Supplementary material for: Associations between lipids in selected brain regions, plasma miRNA, and behavioral and cognitive measures following 28Si ion irradiation
Source: Sci Rep. 2021 Jul 21;11:14899. doi: 10.1038/s41598-021-93869-3 (PMC8295277; doi:10.1038/s41598-021-93869-3)
Supplement: Supplementary file 3 — Supplementary Information 3. [file 41598_2021_93869_MOESM3_ESM.docx]

**The result with the whole dataset (heatmap_wholedataset.pdf)**

The 6 month-old group shows a higher value in ‘NOD2.Fecal’ and ‘context_minute5’ than the other groups but lower ‘Baseline’ score. The 12 month-old mice recorded a lower score at the task of ‘Bin.Preference.Obj2’ and spent less ‘time with objects’. On the other hand, the mice group shows relatively higher lipidomics level at Normalized.MAP2.Cortex than the other groups.

Compared to BalbC, the strain C3H has very low values at ‘OFD1.Fecal’, ‘context_minute4’, ‘Tone’, ‘context_minute5’, and ‘OFD2.Fecal’. But, they spent more ‘time with objects’. The ‘context_minute2’ of male mice is slightly lower than that of the female group. The mice group after the irradiation has a higher value in ‘Preference.Obj.2’.

OFD1(2).Fecal and NOD1(2).Fecal has a positive coefficient with mmu-miR-107-5p, mmu-miR-1188-5p, mmu-miR-431-5p, mmu-miR-412-3p, and mmu-miR-5112 that their function is associated with ‘fructose 6-phosphate metabolic process’, ‘monosaccharide binding’, ‘fructose 1’,’6-bisphosphate metabolic process’, ‘Glycolysis Gluconeogenesis’, and ‘positive regulation of T-helper 1 cell cytokine production’.

The miRNAs, mmu-miR-3106-5p, mmu-miR-1940, mmu-miR-3090-3p, mmu-miR-381-5p, and mmu-miR-217-5p has positive coefficient with ‘Tone’, ‘train_tone3’, and ‘train_tone4’. Their target genes are Tmem88b and Mrpl35. The gene ontology is annotated by ‘cholesterol homeostasis’ and ‘cellular response to low-density lipoprotein particle stimulus’, and ‘negative regulation of reactive oxygen species biosynthetic process’

Bin.2(3).Preference.Obj.2 and Preference.Obj.2 forms a positive coefficient with mmu-miR-290a-3p, mmu-miR-3475-5p, mmu-miR-3065-5p, and mmu-miR-376c-5p. Their gene ontology is associated with ‘positive regulation of NMDA glutamate receptor activity’, ‘ATPase activator activity’, ‘structural constituent of muscle’, and ‘positive regulation of cell migration involved in sprouting angiogenesis’.

Context_minute1(2,4,5) and context_total has positive coefficient with mmu-miR-374c-3p

Mmu-let-7a-2-3p, mmu-miR-34b-5p, mmu-miR-3113-5p, mmu-miR-379-3p, and mmu-miR-219c-3p. Both mmu-miR-34b-5p and mmu-miR-3113-5p are annotated with ‘Sec61 translocon complex’, ‘desensitization of G protein-coupled receptor signaling pathway’,’epithelial cell proliferation involved in renal tubule morphogenesis’, and ‘negative regulation of multicellular organism growth’. Their target gene is 4930402H24Rik. On the other hand, the miRNAs (mmu-miR-1971, mmu-miR-141-5p, mmu-miR-200a-5p, mmu-miR-212-5p, mmu-miR-2861, mmu-miR-147-3p, mmu-miR-182-5p, and mmu-miR-212-5p) has a negative coefficient. Among those, mmu-miR-1971; mmu-miR-212-5p, and mmu-miR-2861 have target genes, Kremen2 and Mcmdc2. Their gene ontology is associated with ‘oogenesis’, and ‘late meiotic recombination nodule assembly‘

For the lipidomic level in ‘Normalized.CD68.Cortex’, it has negative coefficients with mmu-miR-294-3p, mmu-miR-3086-3p, mmu-miR-3060-3p, mmu-miR-3108-5p, mmu-miR-544-3p, mmu-miR-1928, and mmu-miR-194-2-3p (they are known to target *Glrx2*, *Phf14*, *Ube2n* and associated with 'protein disulfide oxidoreductase activity','negative regulation of platelet-derived growth factor receptor-alpha signaling pathway', 'positive regulation of histone modification', and 'inactivation of MAPK activity' in gene ontology enrichment analysis) whereas a positive coefficient with mmu-miR-28b and mmu-miR-429-5p,mmu-miR-488-5p, and mmu-miR-3089-5p (their target genes include *Tars2* and *Slc27a* and the gene ontology is significantly associated with ‘aminoacyl-tRNA editing activity’, ‘threonine-tRNA ligase activity’,’threonyl-tRNA aminoacylation’,’L-lysine import across plasma’, and ‘high-affinity L-ornithine transmembrane transporter’).

The male group has a lower lipidomic at ‘Normalized.CD68.Cortex’ and 12 month-old mice have a higher lipidomic level at ‘Normalized.MAP2.Cortex’.

Next, we repeated the same Lasso regression experiment with the subset of mice group individually after grouping them by their biological condition (the breed age, strain, sex, and radiation) respectively.

**The breed age dependency (subgroup_diff.pdf:page4)**

The mouse strain C3H showed lower Tone and fecal measurements at each age group. The 12 month-old male mice group has a lower value in ‘train_tone4’ and ‘Preference.Obj.2’. Three miRNA, miR-467e-3p, miR-3085-3p, and mmu.miR.1197.5p is highly proportional to ‘Preference.Obj.2’ in the 12 month-old groups.

**The strain dependency (subgroup_diff.pdf:page1)**

In C3H, either ‘context_minute3’ or ‘context_total’ is highly proportional to the mmu-miR-467d-3p and mmu-miR-466e-5p. The overexpression is associated with ‘visual perception’ in gene ontology. In BalbC, ‘context_minute’ is inversely correlated with the mmu-miR-466o-3p or mmu-miR-511-3p. In the brain lipidomics, BalbC shows some meaningful association. The 12-month-old mic group has a higher lipidomic level in ‘Normalized MAP2 Cortex’ and ‘Normalized.BDNF.Cortex’. Interestingly, mmu-miR-466o-3p forms a larger negative coefficient with the response variable, ‘Normalized.CD68.Hypothalamus’. It suggests that as the over-represented mmu-miR-466o-3p in BalbC mice group decreases the ‘CD68.Hypothalamus’ or ‘MAP2.Cortex’ lipidomic level which results in a lower metric in ‘context_minute1’.

**The sex dependency (subgroup_diff.pdf:page2)**

The 12 month-old mice have a lower value in ‘Bin.Preference.Obj.2’ compared to younger mice and, among those, female mice show higher ‘MAP2/BDNF.Cortex’ lipidomic level. Overall, female C3H mice except 6 month-old mice show a lower value in ‘OFD.Fecal’ or ‘NOD.Fecal’.

Both mmu-miR-34a-3p and mmu-miR-3073a-3p in male group are proportional to the ‘train_tone2’. Enrichment analysis results suggest that the over-represented miRNAs are associated with 'inflammatory response to up-regulated antigenic stimulus', 'glucose homeostasis', and 'cytoplasmic vesicle'.
