# Supplementary material for: Associations between lipids in selected brain regions, plasma miRNA, and behavioral and cognitive measures following 28Si ion irradiation
Source: Sci Rep. 2021 Jul 21;11:14899. doi: 10.1038/s41598-021-93869-3 (PMC8295277; doi:10.1038/s41598-021-93869-3)
Supplement: Supplementary file 4 — Supplementary Information 4. [file 41598_2021_93869_MOESM4_ESM.docx]

**Supplemental Figure Legends.**

Suppl. Figure 1. *Effects and trends towards significant effects of ^28^Si ion irradiation on behavioral and cognitive measures of C3H and BALB/c mice at the three time points.* **A.** At TP1, irradiated BALB/c mice spent more time exploring the two objects during the object recognition test than sham-irradiated BALB/c mice. No radiation effect was seen on this measure in C3H mice. *t* = 2.753, **p* = 0.0156. **B.** At TP1, there was a trend towards less freezing during the baseline period (prior to the first tone) during fear conditioning training in irradiated than sham-irradiated BALB/c mice. *t* = 1.806, ^#^*p* = 0.0925. **C.** At TP 1, there was a trend towards increased freezing of C3H mice during the second interval between tone-shock pairings. *t* = 1.825, ^#^*p* = 0.894. **D.** At TP2, there was a trend towards reduced freezing during the tone in the cued fear memory test in irradiated than sham-irradiated C3H mice. *t* = 1.870, ^#^*p* = 0.0825. **E.** At TP2, there was a trend towards reduced freezing of irradiated than sham-irradiated C3H mice during the first tone of fear conditioning training. *t* = 2.006, ^#^*p* = 0.0661. **F.** At TP3, irradiated C3H mice deposited more fecal boli in the first day of open field testing than sham-irradiated C3H mice. *t* = 4.316, **p* = 0.0007. **G.** At Tp3, there was a trend towards irradiated C3H mice depositing more fecal boli on the second day of open field testing than sham-irradiated C3H mice. *t* = 1.983, ^#^*p* = 0.0673. **H.** At TP3, as at TP1, irradiated BALB/c mice spent more time exploring the two objects during the object recognition test than sham-irradiated BALB/c mice. *t* =2.536, **p* = 0.0248. **I.** At TP3, there was a trend towards increased freezing during the contextual fear memory test in irradiated than sham-irradiated BALB/c mice. *t* =1.790, ^#^*p* = 0.0950.

Suppl. Figure 2. **A.** Effects of ^28^Si ion irradiation on BDNF levels in the amygdala of BALB/c mice at TP1. *t* = 2.543, **p* = 0.0234 versus sham-irradiated BALB/c. **B.** Effects of ^28^Si ion irradiation on BDNF levels in the hypothalamus of BALB/c mice at TP1. *t* = 2.444, **p* = 0.0309 versus sham-irradiated BALB/c. **C.** Effects of ^28^Si ion irradiation on CD68 levels in the amygdala of BALB/c mice at TP2. *t* = 2.718, **p* = 0.0167 versus sham-irradiated BALB/c. **D.** No effects of ^28^Si ion irradiation on CD68 levels in the hypothalamus at TP2.

Suppl. Figure 3. *Correlations between plasma miRNAs and cognitive measures at TP2.* Results from sCCA analyses of plasma miRNAs and cognitive measures at time point 2. Three analyses are shown: sham mice (panel A), F1 irradiated mice (panel B), F2 irradiated mice (panel C). The top 3 canonical vector components (linear combinations within each data set denoted K1, K2, K3) with the strongest correlations are detected and presented in the three panels of each analyses. For each component, the explicit linear combinations of cognitive measures are shown in the legend. Pearson’s correlation for each component is shown above each panel. The scatter plots show the linear combination for miRNA on the x-axis and the linear combination of cognitive component in the Y axis, along with the best fit linear line and confidence band (grey shaded area). Venn diagrams show the number and overlap of miRNAs represented collectively across the three canonical components within each irradiation/generation analysis.

Suppl. Figure 4. *Correlations between plasma miRNAs and cognitive measures at TP3.* Results from sCCA analyses of plasma miRNAs and cognitive measures at time point 3. Three analyses are shown: sham mice (panel A), F1 irradiated mice (panel B), F2 irradiated mice (panel C). The top 3 canonical vector components (linear combinations within each data set denoted K1, K2, K3) with the strongest correlations are detected and presented in the three panels of each analyses. For each component, the explicit linear combinations of cognitive measures are shown in the legend. Pearson’s correlation for each component is shown above each panel. The scatter plots show the linear combination for miRNA on the x-axis and the linear combination of cognitive component in the Y axis, along with the best fit linear line and confidence band (grey shaded area). Venn diagrams show the number and overlap of miRNAs represented collectively across the three canonical components within each irradiation/generation analysis.

Suppl. Figure 5. *miRNA pathways over-represented in sCCA correlations with cognitive measures at TP1.*

Over-represented pathways found via miEAA for sets of miRNAs found to be correlated with cognitive measures in sCCA analyses for time point 1 within F1 sham mice (panel A), F1 irradiated mice (panel B), and F2 irradiated mice (panel C). Pathways in larger font represent larger number of miRNAs contained in the pathway are represented in the results. Complete results shown in Suppl. Table 1.

Suppl. Figure 6. *miRNA pathways over-represented in sCCA correlations with cognitive measures at TP2.* Over-represented pathways found via miEAA for sets of miRNAs found to be correlated with cognitive measures in sCCA analyses for time point 2 within F1 sham mice (panel A), F1 irradiated mice (panel B), and F2 irradiated mice (panel C). Pathways in larger font represent larger number of miRNAs contained in the pathway are represented in the results. Complete results shown in Suppl. Table 1.

Suppl. Figure 7. *miRNA pathways over-represented in sCCA correlations with cognitive measures at TP3.* Over-represented pathways found via miEAA for sets of miRNAs found to be correlated with cognitive measures in sCCA analyses for time point 3 within F1 sham mice (panel A), F1 irradiated mice (panel B), and F2 irradiated mice (panel C). Pathways in larger font represent larger number of miRNAs contained in the pathway are represented in the results. Complete results shown in Suppl. Table 1.

Suppl. Figure 8. *Correlations between plasma miRNAs and behavioral measures at TP2.* Results from sCCA analyses of plasma miRNAs and behavioral measures at time point 2. Three analyses are shown: sham mice (panel A), F1 irradiated mice (panel B), F2 irradiated mice (panel C). The top 3 canonical vector components (linear combinations within each data set denoted K1, K2, K3) with the strongest correlations are detected and presented in the three panels of each analyses. For each component, the explicit linear combinations of behavioral measures are shown in the legend. Pearson’s correlation for each component is shown above each panel. The scatter plots show the linear combination for miRNA on the x-axis and the linear combination of behavioral component in the Y axis, along with the best fit linear line and confidence band (grey shaded area). Venn diagrams show the number and overlap of miRNAs represented collectively across the three canonical components within each irradiation/generation analysis.

Suppl. Figure 9. *Correlations between plasma miRNAs and behavioral measures at TP3.* Results from sCCA analyses of plasma miRNAs and behavioral measures at time point 3. Three analyses are shown: sham mice (panel A), F1 irradiated mice (panel B), F2 irradiated mice (panel C). The top 3 canonical vector components (linear combinations within each data set denoted K1, K2, K3) with the strongest correlations are detected and presented in the three panels of each analyses. For each component, the explicit linear combinations of behavioral measures are shown in the legend. Pearson’s correlation for each component is shown above each panel. The scatter plots show the linear combination for miRNA on the x-axis and the linear combination of behavioral component in the Y axis, along with the best fit linear line and confidence band (grey shaded area). Venn diagrams show the number and overlap of miRNAs represented collectively across the three canonical components within each irradiation/generation analysis.

Suppl. Figure 10. *miRNA pathways over-represented in sCCA correlations with behavioral measures at TP1.* Over-represented pathways found via miEAA for sets of miRNAs found to be correlated with behavioral measures in sCCA analyses for time point 1 within F1 sham mice (panel A), F1 irradiated mice (panel B), and F2 irradiated mice (panel C). Pathways in larger font represent larger number of miRNAs contained in the pathway are represented in the results. Complete results shown in Suppl. Table 1.

Suppl. Figure 11. *miRNA pathways over-represented in sCCA correlations with behavioral measures at TP2.* Over-represented pathways found via miEAA for sets of miRNAs found to be correlated with behavioral measures in sCCA analyses for time point 2 within F1 sham mice (panel A), F1 irradiated mice (panel B), and F2 irradiated mice (panel C). Pathways in larger font represent larger number of miRNAs contained in the pathway are represented in the results. Complete results shown in Suppl. Table 1.

Suppl. Figure 12. *miRNA pathways over-represented in sCCA correlations with behavioral measures at TP3.* Over-represented pathways found via miEAA for sets of miRNAs found to be correlated with behavioral measures in sCCA analyses for time point 3 within F1 sham mice (panel A), F1 irradiated mice (panel B), and F2 irradiated mice (panel C). Pathways in larger font represent larger number of miRNAs contained in the pathway are represented in the results. Complete results shown in Suppl. Table 1.

Suppl. Figure 13. *Correlations between plasma miRNAs and lipids at TP1.* Results from sCCA analyses of plasma miRNAs and lipidomics at time point 1. Two analyses are shown: F1 irradiated mice (top panel), F1 sham mice (bottom panel) .The top 3 canonical vector components (linear combinations within each data set denoted K1, K2, K3) with the strongest correlations are detected and presented. For each canonical vector component, the lipid mass is described and annotated. Then, in the third column, the miRNA pathways over-represented in the miRNA found to be correlated with lipids in each canonical component are shown.

Suppl. Figure 14. *Correlations between plasma miRNAs and lipids at TP2.* Results from sCCA analyses of plasma miRNAs and lipidomics at time point 2. Two analyses are shown: F1 irradiated mice (top panel), F1 sham mice (bottom panel). The top 3 canonical vector components (linear combinations within each data set denoted K1, K2, K3) with the strongest correlations are detected and presented. For each canonical vector component, the lipid mass is described and annotated. Then, in the third column, the miRNA pathways over-represented in the miRNA found to be correlated with lipids in each canonical component are shown.

Suppl. Figure 15. *Correlations between plasma miRNAs and lipids at TP3.* Results from sCCA analyses of plasma miRNAs and lipidomics at time point 3. Two analyses are shown: F1 irradiated mice (top panel), F1 sham mice (bottom panel) .The top 3 canonical vector components (linear combinations within each data set denoted K1, K2, K3) with the strongest correlations are detected and presented. For each canonical vector component, the lipid mass is described and annotated. Then, in the third column, the miRNA pathways over-represented in the miRNA found to be correlated with lipids in each canonical component are shown.

Suppl. Figure 16. *Correlations between cognitive measures and lipids at TP1.* Results from sCCA analyses of cognitive measures and lipidomics at time point 1. Two analyses are shown: F1 irradiated mice (top panel), F1 sham mice (bottom panel). The top 3 canonical vector components (linear combinations within each data set denoted K1, K2, K3) with the strongest correlations are detected and presented. For each canonical vector component, the lipid masses are described and annotated.

Suppl. Figure 17. *Correlations between cognitive measures and lipids at TP2.* Results from sCCA analyses of cognitive measures and lipidomics at time point 2. Two analyses are shown: F1 irradiated mice (top panel), F1 sham mice (bottom panel). The top 3 canonical vector components (linear combinations within each data set denoted K1, K2, K3) with the strongest correlations are detected and presented. For each canonical vector component, the lipid masses are described and annotated.

Suppl. Figure 18. *Correlations between cognitive measures and lipids at TP3.* Results from sCCA analyses of cognitive measures and lipidomics at time point 3. Two analyses are shown: F1 irradiated mice (top panel), F1 sham mice (bottom panel). The top 3 canonical vector components (linear combinations within each data set denoted K1, K2, K3) with the strongest correlations are detected and presented. For each canonical vector component, the lipid masses are described and annotated.

Suppl. Figure 19. *Correlations between behavioral measures and lipids at TP1.* Results from sCCA analyses of behavioral measures and lipidomics at time point 1. Two analyses are shown: F1 irradiated mice (top panel), F1 sham mice (bottom panel). The top 3 canonical vector components (linear combinations within each data set denoted K1, K2, K3) with the strongest correlations are detected and presented. For each canonical vector component, the lipid masses are described and annotated.

Suppl. Figure 20. *Correlations between behavioral measures and lipids at TP2.* Results from sCCA analyses of behavioral measures and lipidomics at time point 2. Two analyses are shown: F1 irradiated mice (top panel), F1 sham mice (bottom panel). The top 3 canonical vector components (linear combinations within each data set denoted K1, K2, K3) with the strongest correlations are detected and presented. For each canonical vector component, the lipid masses are described and annotated.

Suppl. Figure 21. *Correlations between behavioral measures and lipids at TP3.* Results from sCCA analyses of behavioral measures and lipidomics at time point 3. Two analyses are shown: F1 irradiated mice (top panel), F1 sham mice (bottom panel). The top 3 canonical vector components (linear combinations within each data set denoted K1, K2, K3) with the strongest correlations are detected and presented. For each canonical vector component, the lipid masses are described and annotated.

**Suppl. Figure 22.** *Heatmap of lasso coefficients describing associations between miRNA and condition to behavioral measure outcomes, stratified by irradiation status..*

Heatmap of magnitude of lasso regression coefficients with behavioral metrics as outcomes, run separately for irradiation and sham mice. Each row corresponds to a behavior outcome, with the independent predictors of miRNA, sex (sexM denotes male vs female), strain (strainC3H), and time point (time_pointTP3, time_pointTP2 compared to TP1 reference group). Darker red denotes strong positive associations and darker blue denotes strong negative associations. In subtitle, “coeff” is the Lasso coefficient and the range of coefficient magnitudes; MSE denotes range of mean square error from cross-validation with 100 repeats.

**Supplementary Table Legends**

Supplementary Table 1. *Results for over-representation analyses of miRNAs correlated to cognitive measures, behavioral measures, and lipidomics.*

Each tab represents the over-presented pathways found in the set of miRNAs found to be correlated to another data set (behavioral measures = “beh”, cognitive measures = “cog”, lipidomics = “lipid”) with sCCA. The miRNAs from all 3 canonical vector components were collapsed into one list for enrichment analyses. Each time point and irradiation group and generation are presented separately within individual tabs (in the names of the tabs “tp1”, “tp2”, “tp3” represent time point 1, 2, 3; “irr_f1”, “irr_f2”, and “sham” represent F1 and F2 irradiated mice, and F1 sham mice.). Within each tab, the columns represent the pathway “category” (database of pathways or diseases queried), “subcategory” denoting the pathway or disease found, “enrichment” denoting over- or under-representation, the p-value of the pathway enrichment test, the adjusted p-value (FDR) and q-value (FDR), the “expected” number of miRNAs observed in a category, and the “observed” number of miRNAs oversved in a category. Lastly, each miRNA in the pathway/subcategoory detected in the sCCA components is shown following the miEAA enrichment results.

Supplementary Table 2a. Lasso regression coefficients from input variables of miRNAs expression abundance and four categorical variables (sex, mice strain, mice time points, and irradiation or not) and the response variable, mice behavioral measures.

Supplementary Table 2b. Lasso regression coefficients from input variables, miRNAs expression abundance and four categorical variables (sex, mice strain, mice time points, and irradiation or not) and the response variable, plasma lipidomics levels.

Supplementary Table 3. Lasso regression coefficients from input variables, miRNAs expression abundance and four categorical variables (sex, mice strain, mice time points, and irradiation or not) and the response variable, either hypothalamus (lasso_coeff_miRNA_vs_lipidH) and amygdala (lasso_coeff_miRNA_vs_lipidA)
