# Supplementary material for: Associations between lipids in selected brain regions, plasma miRNA, and behavioral and cognitive measures following 28Si ion irradiation
Source: Sci Rep. 2021 Jul 21;11:14899. doi: 10.1038/s41598-021-93869-3 (PMC8295277; doi:10.1038/s41598-021-93869-3)
Supplement: Supplementary file 9 — Supplementary Information 9. [file 41598_2021_93869_MOESM9_ESM.pdf]

INGENUITY
PATHWAY ANALYSIS

Analysis Name: x451_305_A - 2020-11-20 05:01 PM
Analysis Creation Date: 2020-11-20
Build version: exported
Content version: 57662101 (Release Date: 2020-09-15)

Experiment Metadata

Name	Value

Analysis Settings

Top Canonical Pathways

Top Upstream Regulators

Top Diseases and Bio Functions

Diseases and Disorders

(c) 2000-2020 QIAGEN. All rights reserved.

Name	p-value range	# Molecules
**Organismal Injury and Abnormalities**	4.71E-02 - 6.20E-21	54
**Reproductive System Disease**	2.77E-02 - 6.20E-21	38
**Psychological Disorders**	1.57E-02 - 1.89E-17	26
**Neurological Disease**	3.01E-02 - 4.09E-17	31
**Cancer**	4.63E-02 - 4.70E-16	45

Molecular and Cellular Functions

Name	p-value range	# Molecules
**Cellular Development**	4.51E-02 - 3.67E-07	36
**Cellular Growth and Proliferation**	4.51E-02 - 3.67E-07	31
**Cell Cycle**	4.61E-02 - 3.78E-07	12
**Cellular Movement**	4.61E-02 - 1.86E-05	20
**Cell Death and Survival**	4.24E-02 - 3.15E-04	18

Physiological System Development and Function

Name	p-value range	# Molecules
**Embryonic Development**	4.61E-02 - 3.78E-07	10
**Digestive System Development and Function**	5.22E-07 - 5.22E-07	5
**Hepatic System Development and Function**	5.22E-07 - 5.22E-07	5
**Organ Development**	4.51E-02 - 5.22E-07	7
**Connective Tissue Development and Function**	3.81E-02 - 3.30E-06	12

(c) 2000-2020 QIAGEN. All rights reserved.

INGENUITY PATHWAY ANALYSIS

Top Tox Functions

Cardiotoxicity

Name	p-value range	# Molecules
Cardiac Dilation	3.49E-06 - 3.49E-06	9
Cardiac Enlargement	1.61E-01 - 3.49E-06	10
Cardiac Regeneration	3.81E-02 - 3.81E-02	1
Cardiac Proliferation	4.51E-02 - 4.35E-02	2
Congenital Heart Anomaly	7.74E-02 - 7.74E-02	1

Hepatotoxicity

Name	p-value range	# Molecules
Liver Inflammation/Hepatitis	5.22E-07 - 5.22E-07	5
Liver Cirrhosis	7.07E-04 - 5.99E-05	7
Liver Fibrosis	1.51E-01 - 7.07E-04	5
Liver Steatosis	7.64E-03 - 7.64E-03	3
Liver Hyperplasia/Hyperproliferation	4.11E-01 - 8.29E-03	21

Nephrotoxicity

(c) 2000-2020 QIAGEN. All rights reserved.

INGENUITY PATHWAY ANALYSIS

Name	p-value range	# Molecules
**Glomerular Injury**	9.79E-13 - 9.79E-13	10
**Renal Inflammation**	9.79E-13 - 9.79E-13	10
**Renal Nephritis**	9.79E-13 - 9.79E-13	10
**Renal Necrosis/Cell Death**	5.69E-01 - 2.19E-01	2

Top Regulator Effect Networks

Top Networks

ID	Associated Network Functions	Score
**1**	Organismal Injury and Abnormalities, Reproductive System Disease, Psychological Disorders	26
**2**	Connective Tissue Disorders, Inflammatory Disease, Organismal Injury and Abnormalities	26
**3**	Cell Cycle, Embryonic Development, Cellular Development	20
**4**	Skeletal and Muscular System Development and Function, Tissue Morphology, Inflammatory Disease	20

(c) 2000-2020 QIAGEN. All rights reserved.

INGENUITY PATHWAY ANALYSIS

5	Neurological Disease, Organismal Injury and Abnormalities, Psychological Disorders	18

Top Tox Lists

Name	p-value	Overlap
Renal Ischemia-Reperfusion Injury MicroRNA Biomarker Panel (Mouse)	2.20E-02	12.5 % 1/8
Decreases Transmembrane Potential of Mitochondria and Mitochondrial Membrane	3.08E-01	0.8 % 1/132

Top My Lists

Top My Pathways

Top Analysis-Ready Molecules

(c) 2000-2020 QIAGEN. All rights reserved.

INGENUITY PATHWAY ANALYSIS
