# Supplementary material for: Associations between lipids in selected brain regions, plasma miRNA, and behavioral and cognitive measures following 28Si ion irradiation
Source: Sci Rep. 2021 Jul 21;11:14899. doi: 10.1038/s41598-021-93869-3 (PMC8295277; doi:10.1038/s41598-021-93869-3)
Supplement: Supplementary file 13 — Supplementary Information 13. [file 41598_2021_93869_MOESM13_ESM.pdf]

INGENUITY
PATHWAY ANALYSIS

Analysis Name: x888_641_A - 2020-11-22 06:38 PM
Analysis Creation Date: 2020-11-22
Build version: exported
Content version: 57662101 (Release Date: 2020-09-15)

Experiment Metadata

Name	Value

Analysis Settings

Reference set: Ingenuity Knowledge Base (Genes Only)
Relationship to include: Direct and Indirect
Includes Endogenous Chemicals
Optional Analyses: My Pathways My List

Filter Summary:
Consider only molecules and/or relationships where
(species = Human OR Rat OR Mouse OR Uncategorized) AND
(confidence = Experimentally Observed) AND
(tissues/cell lines = Skeletal Muscle OR Cytotoxic T cells OR Other Cervical cancer cell line OR RXF-393 OR HCC-2998 OR Cornea OR Other Monocyte-derived dendritic cells OR Olfactory Bulb OR SNB-75 OR A498 OR Trigeminal Ganglion OR NIH/3T3 cells OR Placenta OR Granulocytes not otherwise specified OR T lymphocytes not otherwise specified OR Sertoli cells OR CD34+ cells OR Pancreas OR T47-D OR

(c) 2000-2020 QIAGEN. All rights reserved.

U266 OR Brainstem OR Naive helper T cells OR Other Macrophage Cancer Cell Lines OR Large Intestine OR Medulla Oblongata OR KM-12 OR Bone marrow-derived macrophages OR Other Endothelial cells OR Lymph node OR MDA-MB-231 OR Monocyte-derived dendritic cells not otherwise specified OR Thymocytes OR LNCaP cells OR Melanocytes OR U87MG OR Hep3B OR Prostate Cancer Cell Lines not otherwise specified OR Caudate Nucleus OR Other Immune cell lines OR Other Osteosarcoma Cell Lines OR HEL OR Nervous System not otherwise specified OR Embryonic stem cells OR Fibroblasts OR NCI-H522 OR Splenocytes OR Central memory cytotoxic T cells OR Other Organ Systems OR Stromal cells OR Lens OR 786-0 OR Chondrocytes OR Vd2 Gamma-delta T cells OR Neutrophils OR Other Cells OR U937 OR Other Leukemia Cell Lines OR Caco2 cells OR IGROV1 OR Striatum OR Corpus Callosum OR Cells not otherwise specified OR Teratocarcinoma Cell Lines not otherwise specified OR Amygdala OR HMC-1 OR Ovarian Cancer Cell Lines not otherwise specified OR Granulosa cells OR Other Dendritic cells OR HCT-15 OR White Matter OR Hepatocytes OR Ventricular Zone OR Granule cells OR Hematopoietic progenitor cells OR SK-MEL-5 OR Forestomach OR A2780 OR Organ Systems not otherwise specified OR Plasma cells OR BT-474 OR Other Memory T lymphocytes OR Spinal Cord OR UO-31 OR Bone marrow-derived dendritic cells OR Brain OR Th17 cells OR MALME-3M OR P19 OR Other Bone marrow cells OR Activated CD56bright NK cells OR SR OR Other Nervous System OR Other Neuroblastoma Cell Lines OR Breast Cancer Cell Lines not otherwise specified OR SK-OV-3 OR B lymphocytes not otherwise specified OR Dendritic cells not otherwise specified OR MG-63 OR HOP-92 OR Other NK cells OR J774 OR Other Lung Cancer Cell Lines OR Smooth muscle cells not otherwise specified OR Other Neurons OR EKVX OR Other Pancreatic Cancer Cell Lines OR MDA-MB-435 OR CAKI-1 OR H460 OR Neurons not otherwise specified OR MOLT-4 OR Stomach OR Thalamus OR Melanoma Cell Lines not otherwise specified OR COLO205 OR Small Intestine OR 293 cells OR Peritoneal macrophages OR MEF cells OR HuH7 OR RBL-2H3 OR Swiss 3T3 cells OR Lymphocytes not otherwise specified OR Monocytes not otherwise specified OR Microvascular endothelial cells OR Macrophage Cancer Cell Lines not otherwise specified OR BT-549 OR LOX IMVI OR MDA-MB-468 OR Other Hepatoma Cell Lines OR Epithelial cells not otherwise specified OR Granule Cell Layer OR Peripheral blood leukocytes not otherwise specified OR Esophagus OR Other Lymphocytes OR Other Myeloma Cell Lines OR Salivary Gland OR Osteosarcoma Cell Lines not otherwise specified OR NCI-ADR-RES OR HCT-116 OR Pancreatic Cancer Cell Lines not otherwise specified OR Other CNS Cell Lines OR SK-MEL-2 OR CD56bright NK cells OR Uterus OR Vd1 Gamma-delta T cells OR Bone marrow cells not otherwise specified OR Jurkat OR U251 OR Trachea OR HL-60 OR OVCAR-4 OR Th2 cells OR Testis OR Cortical neurons OR Beta islet cells OR Other Smooth muscle cells OR OVCAR-8 OR Prostate Gland OR Leukemia Cell Lines not otherwise specified OR Cardiomyocytes OR Peripheral blood monocytes OR ACHN OR WEHI-231 OR Pro-B lymphocytes OR Crypt OR CNS Cell Lines not otherwise specified OR Activated Vd1 Gamma-delta T cells OR THP-1 OR CD4+ T-lymphocytes OR DU-145 OR Other Breast Cancer Cell Lines OR Skin OR Other Colon Cancer Cell Lines OR Heart OR HT29 OR M14 OR NCI-H226 OR Other Pheochromocytoma cell lines OR SF-268 OR Thyroid Gland OR TK-10 OR Other T lymphocytes OR Memory B cells OR MDA-N OR SF-539 OR Pyramidal neurons OR Effector memory RA+ cytotoxic T cells OR Cartilage Tissue OR Dermis OR Sciatic Nerve OR NK cells not otherwise specified OR BDCA-1+ dendritic cells OR Myeloid dendritic cells

(c) 2000-2020 QIAGEN. All rights reserved.

OR Other Peripheral blood leukocytes OR PBMCs OR Substantia Nigra OR HepG2 OR Epidermis OR Cos-7 cells OR Calvaria OR Other Cell Line OR Parietal Lobe OR Activated Vd2 Gamma-delta T cells OR Pituitary Gland OR Other Melanoma Cell Lines OR Kidney Cancer Cell Lines not otherwise specified OR Other Ovarian Cancer Cell Lines OR Th1 cells OR Choroid Plexus OR Oocytes OR Natural T-regulatory cells OR Cerebellum OR Microglia OR HS 578T OR RAW 264.7 OR SF-295 OR Stem cells not otherwise specified OR Kidney OR Purkinje cells OR OVCAR-5 OR Adrenal Gland OR Langerhans cells OR Megakaryocytes OR Min6 OR SN12C OR Other Prostate Cancer Cell Lines OR RKO OR RPMI-8266 OR BDCA-3+ dendritic cells OR Tissues and Primary Cells not otherwise specified OR Vascular smooth muscle cells OR Gray Matter OR Astrocytes OR A375 OR Cerebral Cortex OR Cerebral Ventricles OR SW-620 OR Eosinophils OR OVCAR-3 OR PANC-1 OR Lung Cancer Cell Lines not otherwise specified OR Other Lymphoma Cell Lines OR NCI-H23 OR Intraepithelial T lymphocytes OR Pheochromocytoma cell lines not otherwise specified OR Liver OR SW-480 OR Dorsal Root Ganglion OR Mammary Gland OR J-774A.1 OR Macrophages not otherwise specified OR SK-N-SH OR Bladder OR Mononuclear leukocytes not otherwise specified OR SK-MEL-28 OR Immature monocyte-derived dendritic cells OR Other Granulocytes OR Monocyte-derived macrophage OR Activated CD56dim NK cells OR Mesenchymal stem cells OR U2OS OR UACC-257 OR PC-3 OR Ovary OR MDA-MB-361 OR Adipocytes OR Putamen OR Adipose OR Effector memory cytotoxic T cells OR MCF7 OR Blood platelets OR Other Tissues and Primary Cells OR Hippocampus OR Peripheral blood lymphocytes OR K-562 OR Other Teratocarcinoma Cell Lines OR Cell Line not otherwise specified OR UACC-62 OR Kidney cell lines not otherwise specified OR Mast cells OR Other Fibroblast cell lines OR Effector memory helper T cells OR Effector T cells OR Lung OR INS-1 OR Activated helper T cells OR CD56dim NK cells OR Other Monocytes OR Colon Cancer Cell Lines not otherwise specified OR Myeloma Cell Lines not otherwise specified OR Other Kidney cell lines OR HUVEC cells OR Other Immune cells OR Pre-B lymphocytes OR Hypothalamus OR Keratinocytes OR Other B lymphocytes OR HeLa OR Immune cell lines not otherwise specified OR Thymus OR HOP-62 OR Other Mononuclear leukocytes OR Other Stem cells OR Other Macrophages OR NT2/D1 OR Murine NKT cells OR 3T3-L1 cells OR Cervical cancer cell line not otherwise specified OR Memory T lymphocytes not otherwise specified OR Naive B cells OR Central memory helper T cells OR Spleen OR Subventricular Zone OR Mature monocyte-derived dendritic cells OR A549-ATCC OR Fibroblast cell lines not otherwise specified OR Other Epithelial cells OR NB4 OR PC-12 cells OR CCRF-CEM OR Other Kidney Cancer Cell Lines OR Endothelial cells not otherwise specified OR Nucleus Accumbens OR Osteoblasts OR Lymphoma Cell Lines not otherwise specified OR Plasmacytoid dendritic cells OR BA/F3 OR Smooth Muscle OR Immune cells not otherwise specified OR Hepatoma Cell Lines not otherwise specified OR Retina OR Neuroblastoma Cell Lines not otherwise specified OR NCI-H332M) AND

(mol. types = biologic drug OR canonical pathway OR chemical - endogenous mammalian OR chemical - endogenous non-mammalian OR chemical - kinase inhibitor OR chemical - other OR chemical - protease inhibitor OR chemical drug OR chemical reagent OR chemical toxicant OR complex OR cytokine OR disease OR enzyme OR function OR G-protein coupled receptor OR group OR growth factor OR ion channel OR kinase OR ligand-dependent nuclear receptor OR mature microRNA OR microRNA OR other OR peptidase OR phosphatase OR transcription

(c) 2000-2020 QIAGEN. All rights reserved.

regulator OR translation regulator OR transmembrane receptor OR transporter) AND (data sources = An Open Access Database of Genome-wide Association Results OR BIND OR BioGRID OR Catalogue Of Somatic Mutations In Cancer (COSMIC) OR Chemical Carcinogenesis Research Information System (CCRIS) OR ClinicalTrials.gov OR ClinVar OR Cognia OR DIP OR DrugBank OR Gene Ontology (GO) OR GVK Biosciences OR Hazardous Substances Data Bank (HSDB) OR HumanCyc OR Ingenuity Expert Findings OR Ingenuity ExpertAssist Findings OR IntAct OR Interactome studies OR MIPS OR miRBase OR miRecords OR Mouse Genome Database (MGD) OR Obesity Gene Map Database OR Online Mendelian Inheritance in Man (OMIM) OR TarBase OR TargetScan Human)

Top Canonical Pathways

Top Upstream Regulators

Top Diseases and Bio Functions

Diseases and Disorders

Name	p-value range	# Molecules
**Organismal Injury and Abnormalities**	4.89E-02 - 1.14E-22	43
**Reproductive System Disease**	4.48E-02 - 1.14E-22	25
**Inflammatory Disease**	1.59E-02 - 2.32E-15	18
**Inflammatory Response**	3.82E-02 - 2.32E-15	21
**Renal and Urological Disease**	4.01E-02 - 2.32E-15	14

(c) 2000-2020 QIAGEN. All rights reserved.

INGENUITY PATHWAY ANALYSIS

Molecular and Cellular Functions

Name	p-value range	# Molecules
Cellular Development	4.70E-02 - 2.28E-04	19
Cellular Growth and Proliferation	3.60E-02 - 2.28E-04	17
Cellular Movement	4.92E-02 - 2.30E-04	14
Cell Cycle	3.38E-02 - 1.74E-03	3
Cell Death and Survival	4.92E-02 - 9.12E-03	3

Physiological System Development and Function

Name	p-value range	# Molecules
Digestive System Development and Function	7.56E-03 - 7.56E-03	2
Hepatic System Development and Function	7.56E-03 - 7.56E-03	2
Organ Development	3.60E-02 - 7.56E-03	3
Hematological System Development and Function	2.71E-02 - 1.14E-02	2
Lymphoid Tissue Structure and Development	1.14E-02 - 1.14E-02	1

Top Tox Functions

Cardiotoxicity

Name	p-value range	# Molecules
Cardiac Regeneration	3.16E-02 - 3.16E-02	1

(c) 2000-2020 QIAGEN. All rights reserved.

INGENUITY PATHWAY ANALYSIS

**Cardiac Proliferation**	3.60E-02 - 3.60E-02	1
**Congenital Heart Anomaly**	6.43E-02 - 6.43E-02	1
**Cardiac Fibrosis**	1.23E-01 - 1.23E-01	2
**Cardiac Dilation**	2.69E-01 - 2.69E-01	2

Hepatotoxicity

Name	p-value range	# Molecules
**Liver Steatosis**	4.47E-03 - 4.47E-03	3
**Liver Inflammation/Hepatitis**	7.56E-03 - 7.56E-03	2
**Liver Cirrhosis**	1.16E-01 - 4.89E-02	3
**Hepatocellular carcinoma**	5.13E-01 - 5.57E-02	5
**Liver Hyperplasia/Hyperproliferation**	1.00E00 - 5.57E-02	9

Nephrotoxicity

Name	p-value range	# Molecules
**Glomerular Injury**	3.28E-02 - 2.32E-15	13
**Renal Inflammation**	2.32E-15 - 2.32E-15	11
**Renal Nephritis**	2.32E-15 - 2.32E-15	11
**Renal Fibrosis**	3.28E-02 - 3.28E-02	2

Top Regulator Effect Networks

(c) 2000-2020 QIAGEN. All rights reserved.

INGENUITY PATHWAY ANALYSIS

Top Networks

ID	Associated Network Functions	Score
**1**	Organismal Injury and Abnormalities, Reproductive System Disease, Gene Expression	24
**2**	Endocrine System Disorders, Gastrointestinal Disease, Metabolic Disease	22
**3**	Organismal Injury and Abnormalities, Reproductive System Disease, Skeletal and Muscular Disorders	20
**4**	Glomerular Injury, Inflammatory Disease, Inflammatory Response	18
**5**	Gene Expression, Developmental Disorder, Hereditary Disorder	18

Top Tox Lists

Name	p-value	Overlap
**Renal Ischemia-Reperfusion Injury MicroRNA Biomarker Panel (Mouse)**	6.27E-07	37.5 % 3/8

(c) 2000-2020 QIAGEN. All rights reserved.

Top My Lists

Top My Pathways

Top Analysis-Ready Molecules

(c) 2000-2020 QIAGEN. All rights reserved.
